# Immunogenicity and safety of a quadrivalent plant-derived virus like particle influenza vaccine candidate—Two randomized Phase II clinical trials in 18 to 49 and ≥50 years old adults

**DOI:** 10.1371/journal.pone.0216533

**Published:** 2019-06-05

**Authors:** Stéphane Pillet, Julie Couillard, Sonia Trépanier, Jean-François Poulin, Bader Yassine-Diab, Bruno Guy, Brian J. Ward, Nathalie Landry

**Affiliations:** 1 Medicago Inc., Québec City, Québec, Canada; 2 Research Institute, McGill University Health Centre, Montreal, Québec, Canada; 3 Caprion Biosciences Inc., Montréal, Québec, Canada; 4 Expert/Consultant–Microbiology and Vaccinology, Lyon, France; Public Health England, UNITED KINGDOM

## Abstract

**Background:**

New influenza vaccines eliciting more effective protection are needed, particularly for the elderly who paid a large and disproportional toll of hospitalization and dead during seasonal influenza epidemics. Low (≤15 μg/strain) doses of a new plant-derived virus-like-particle (VLP) vaccine candidate have been shown to induce humoral and cellular responses against both homologous and heterologous strains in healthy adults 18–64 years of age. The two clinical trials reported here addressed the safety and immunogenicity of higher doses (≥15 μg/strain) of quadrivalent VLP candidate vaccine on 18–49 years old and ≥50 years old subjects. We also investigated the impact of alum on the immunogenicity induced by lower doses of the vaccine candidate.

**Method:**

In the first Phase II trial reported here (NCT02233816), 18–49 year old subjects received 15, 30 or 60 μg/strain of a hemagglutinin-bearing quadrivalent virus-like particle (QVLP) vaccine or placebo. In the second trial (NCT02236052), ≥50 years old subjects received QVLP as above or placebo with additional groups receiving 7.5 or 15 μg/strain with alum. Along with safety recording, the humoral and cell-mediated immune responses were analyzed.

**Results:**

Local and systemic side-effects were similar to those reported previously. The QVLP vaccine induced significant homologous and heterologous antibody responses at the two higher doses, the addition of alum having little-to-no effect. Serologic outcomes tended to be lower in ≥50 years old subjects previously vaccinated. The candidate vaccine also consistently elicited both homologous and heterologous antigen-specific CD4^+^ T cells characterized by their production of interferon-gamma (IFN-γ), interleukine-2 (IL-2) and/or tumor-necrosis factor alpha (TNF-α) upon *ex vivo* antigenic restimulation.

**Conclusion:**

Overall, the 30 μg dose produced the most consistent humoral and cellular responses in both 18–49 and ≥50 years old subjects, strongly supporting the clinical development of this candidate vaccine. That particular dose was chosen to test in the ongoing Phase III clinical trial.

## Introduction

Influenza A viruses are a major public health threat and seasonal epidemics account for more than 200,000 hospitalizations and 30,000 deaths annually in USA only [1]. Older adults are particularly vulnerable to acute respiratory illness, especially influenza which contributes disproportionately to this burden [1–3]. Influenza is the most common cause of viral pneumonia and associated complications leading to frailty and loss of autonomy in older adults [4, 5], and in this regard aging of the population in the coming decades is becoming one of the greatest demographic and public health challenges facing industrialized countries. Vaccination currently represents the most effective intervention against influenza and its associated complications in adults and elderly [6, 7]. Unfortunately, antibody (Ab) responses and the protection elicited by available vaccines tend to be lower in older as compared to younger adults [8, 9]. These relatively poor responses in older adults are multifactorial with contributions from underlying medical conditions, a lifetime of prior exposures to influenza antigens through vaccination and natural infection [10], low-level chronic inflammation (*i*.*e*.: so-called ‘inflammaging’, [11]) and general immunosenescence [12, 13]. It is possible that the design of the influenza vaccines themselves also contributes to limited vaccine efficacy in the elderly. All of the commercially-available influenza vaccines have been developed to optimally induce strain-specific Ab response and hemagglutination inhibition (HI) titers in particular [14]. This focus on antibodies may have had negative effects on influenza vaccine development in general [15], including particularly damaging effects on the development of vaccines for the elderly who can derive significant benefit from vaccination despite making little-to-no Ab response [16–18]. It is likely that cell-mediated immunity (CMI) is important at all ages but becomes pivotal in protecting the elderly [7, 18, 19]. Candidate vaccines that can induce both Ab and cellular responses might therefore provide better protection, especially in older people. Plant-derived virus-like-particles (VLP) vaccines are produced by transient transfection of *Nicotiana benthamiana* and take the form of 80–120 nm enveloped vesicles studded with wild-type hemagglutinin (HA) trimers [20]. These vaccines appear to have intrinsic adjuvant-like activity [21] and are handled by both murine and human antigen presenting cells in a fashion similar to intact virus [22]. Supporting a potential benefit in the older population, we have recently shown that even very old Balb/c mice (20–24 months of age) are better protected from H1N1 influenza challenge after vaccination with plant-derived VLP than with a standard split virion vaccine despite low or even absent Ab titers, but in presence of substantial cellular responses [23]. We have previously shown that 15 μg, 9 μg and even 3 μg of a quadrivalent plant-derived influenza VLP vaccine candidate (QVLP) can induce strong humoral and cellular responses against both homologous and heterologous strains in healthy adults 18–64 years of age [24]. Herein, we report on the safety and the impact of higher doses of QVLP (15–60 μg/strain) as well as the inclusion of Alum as an adjuvant on the humoral and cellular responses in both young (18–49 years old, Adults) and older (≥50 years old, OA≥50) adults in two different Phase II clinical trials.

## Material and methods

### Production of plant-derived HA VLP influenza vaccine

The QVLP vaccine was produced in *N*. *benthamiana* using the *Agrobacterium* infiltration-based HA0 transient expression platform as previously described [25, 26]. The HA proteins in the VLP were based on human sequences of A/California/07/2009 H1N1 (H1/Cal), A/Victoria/361/11 H3N2 (H3/Vic), B/Brisbane/60/08 (B/Bris, Victoria lineage) and B/Massachusetts/02/2012 (B/Mass, Yamagata lineage) influenza strains according to the recommendations of the World Health Organization (WHO) for Northern hemisphere vaccines in 2013–2014. The drug substances for each strain were combined into a quadrivalent drug product and the final doses were based on HA content per strain.

### Study design, procedures and ethics

The two Phase II studies were approved by each of the sites’ Ethics Review Boards as well as the Health Products and Food Branch of Health Canada, and were carried out in accordance with the Declaration of Helsinki and the principles of Good Clinical Practices and approved by the different institutional ethic committee (*i*.*e*. Aspire IRB, McGill University Health Center REB, the Comité d'Éthique de la Recherche du CHU de Québec and IRB service). Written informed consent was obtained from all study participants. Each dose of QVLP was administered intramuscularly (IM) in the deltoid muscle for a volume of 0.5 mL for all dose levels, with the exception of the 60 μg per strain doses, for which the volume of injection was 1.0 mL.

#### Demographics and subjects

Subjects in general good health were selected by the Investigator based upon the medical history, a physical examination and a panel of screening clinical laboratory tests. Subjects were excluded if they had received any vaccine within 30 days pre-vaccination or any adjuvanted or investigational influenza vaccine within one year pre-vaccination. Pregnant women were excluded and female subjects of childbearing potential were required to use an acceptable method of contraception from one month prior to vaccination until at least 60 days post-vaccination. Subject demographics and baseline characteristics for the Safety Analysis Set are summarized in S1 and S2 Tables. For both studies, all dose groups including Placebo, had slightly more women (≥53.3%) than men; the number of women and men were relatively similar across the vaccine groups. More than half of the subjects were white or Caucasian. The mean age of subjects ranged from 33 to 36 years and was comparable across all groups for Adults and from 63 to 64 years for OA≥50. The mean body mass index (BMI) ranged from 25kg/m^2^ to 27 kg/m^2^ and was also comparable across all groups in both studies. Overall, no statistically significant differences in demographics and baseline characteristics were found between the different groups (ANOVA and Fisher’s exact test).

#### Study design and procedures

The complete protocols of the two studies are provided as Supplemental Material. The authors confirm that all ongoing and related trials for this drug/intervention are registered.

**Fig 1:**
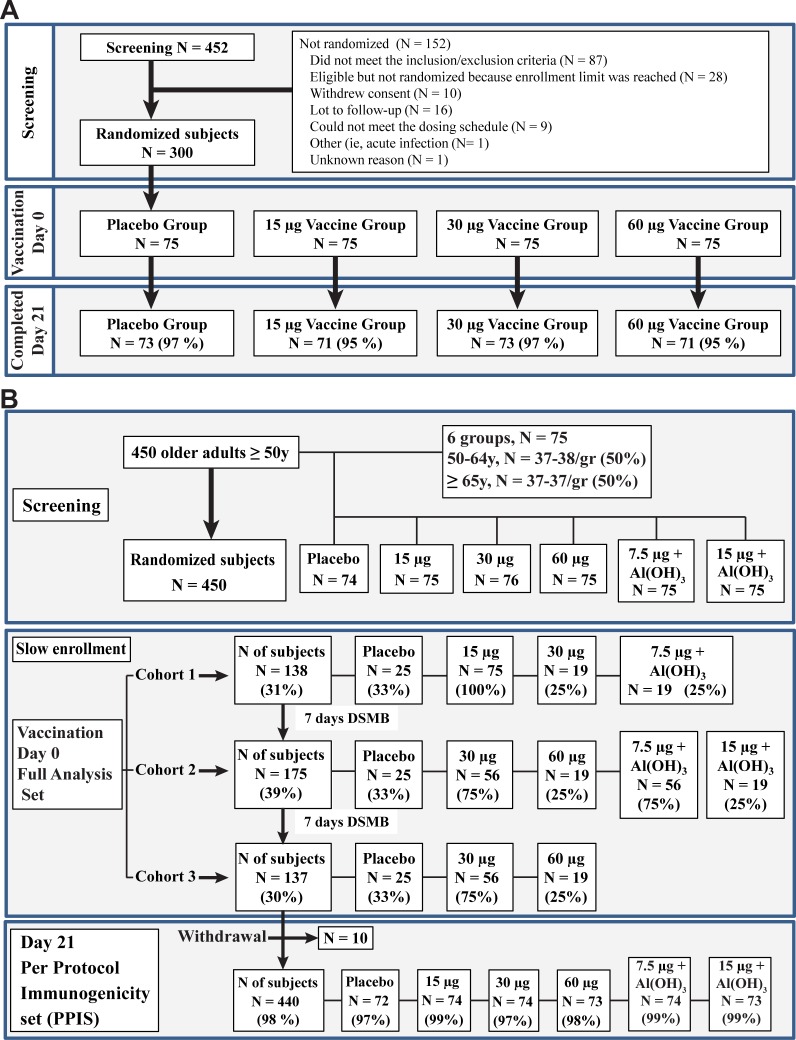
Disposition from screening to day 21 visit in (A) Adults (18-49y) and (B) Older Adults (≥50y).

The Adults study was an observer-blind, placebo-controlled, dose-ranging clinical trial (NCT02233816) involving 300 healthy males and females 18 to 49 years of age. Subjects were randomized in a 1:1:1:1 ratio into four treatment groups that received one IM injection in the deltoid muscle of 15, 30 or 60 μg HA equivalent per strain and a placebo (saline), respectively named QVLP15, QVLP30, QVLP60 and Placebo groups. The study was conducted at two sites in the United States between August 2014 and May 2016. Fig 1A provides a summary of the numbers of subjects vaccinated in each cohort and in each treatment group. All 300 subjects who were randomized received their scheduled dose of QVLP or Placebo and were included in the Safety Analysis Set. The Day 21 consisted of 271 subjects (90.3%) with no major protocol violations. The large majority of subjects (>93.3% in each vaccine group) had not received an influenza vaccine in the 24 months prior to receiving the study vaccine.

The OA≥50 study was an observer-blind, placebo-controlled, dose-ranging clinical trial (NCT02236052) involving a total of 450 eligible males and females 50 years of age and older. Subjects were randomized in a 1:1:1:1:1:1 ratio into six treatment groups. Three received one IM injection in the deltoid muscle of 15, 30 or 60 μg HA equivalent per strain (QVLP15, QVLP30, QVLP60 respectively). Additional groups received one IM injection with 7.5 or 15 μg VLP HA equivalent per strain mixed with 0.5 mg Alhydrogel (Alum; Brenntag Canada Inc, Toronto, ON) immediately prior to injection (QVLP7.5+Alum, QVLP15+Alum respectively) or Placebo (saline). Fig 1B provides a summary of the numbers of subjects vaccinated in each cohort and in each treatment group. The study was conducted at three sites in Canada between July 2014 and June 2015. Since more than 60% of the Canadians ≥50 years of age receive seasonal vaccination and prior exposure could affect the humoral response, we assessed the impact of vaccination in the two years before this study on the serum antibody responses. Following collection of the immunogenicity sample 21 days after injection (D21) a large proportion of OA≥50 subjects (85.6%, overall) elected to receive the offered commercial influenza vaccine (Influvac).

### Safety and reactogenicity assessments

Subjects were observed for 30 minutes after vaccination for immediate reactions and were provided with diary cards to record any solicited local or systemic reactions that occurred after vaccination. Solicited local reactions (pain, swelling, and redness/erythema at the injection site) and solicited systemic reactions (fever [≥38°C], headache, muscle aches, joint aches, fatigue, chills, malaise, swelling in the axilla or neck) were recorded for seven days following vaccination. Unsolicited adverse events (AEs) were collected from the day of injection (D0) through D21 after the injection. Serious adverse events (SAEs), new onset of chronic diseases (NOCDs), and AEs leading to study withdrawal were collected throughout the study. Any solicited local or systemic reactions that persisted beyond D7, were also recorded as AEs, with an AE onset defined as 168 hours (seven days) post-vaccination. Each solicited local and systemic reaction and unsolicited AE, SAE, and NOCD was graded for intensity (mild, moderate, severe, or potentially life threatening). Site investigators evaluated the solicited systemic reactions and unsolicited AEs, SAEs, and NOCDs for causality (definitely not related, probably not related, possibly related, probably related or definitely related). All solicited local reactions were assigned a causality of ‘definitely related’. *P*-values for the difference between each pair of treatment groups and for overall difference among treatment groups were calculated using Fisher’s exact test or Chi-square test.

### Immunogenicity

#### Antibody response

For both studies, serum samples were collected on D0 and D21 for HI and microneutralization (MN) assays. The HI assays were performed as previously described according to the WHO recommendation [27, 28]. The homologous H1N1, H3N2, B/Brisbane (Victoria lineage) and B/Massachusetts (Yamagata lineage) antigens were obtained from the National Institute for Biological Standards and Control (NIBSC, London, UK). Cross-reactive responses were tested using A/Brisbane/59/2007 H1N1 (H1/Bris), A/Uruguay/716/2007 H3N2 (H3/Urug), B/Malaysia/2506/2004 (B/Mal) and B/Florida/4/2006 (B/Flo) antigens (NIBSC). Serum titers are expressed as the reciprocal of the highest dilution that showed complete inhibition of hemagglutination. Sera with no detectable HI inhibition were assigned a value of 4 for statistical purposes. The geometric mean titers (GMT) at D21 or geometric mean fold rise (GMFR) with their 95% CIs were derived from the ANCOVA least squares means results per treatment. The serum antibody endpoints were assessed using the *per protocol* data set. *P*-value for treatment comparisons were based on ANCOVA on log10 transformed data at D21 or fold rise. The seroconversion rate (SCR) and the seroprotection rate (percentage of subjects achieving a HI titer ≥40, SPR) were defined according to regulatory criteria [29]. The *P*-values for the difference in SCR and SPR between treatment groups, par of treatments were calculated using Fisher’s exact test. The MN titers against the homologous strains A/Cal, A/Vic, B/Bris and B/Mass were measured in serum at D0 and D21 according to the WHO guideline for the serologic diagnosis of influenza [27]. The MN titers are expressed as the reciprocal of the highest dilution that showed complete neutralization of input virus. Sera that tested negative at a 1:10 dilution were assigned a titer of 5 for statistical analysis.

#### Cell-mediated immune response

The T cell response was assessed in 10–12 subjects/group at D0 and D21 as previously described [24, 30]. Briefly, PBMC were stimulated *ex vivo* with 2.5 μg/mL of homologous VLP (*i*.*e*.: A/Cal, A/Vic, B/Bris or B/Mass). In order to assess the heterologous response, PBMC were stimulated with 2.5 μg/ml of peptide pools (GenScript, Piscataway, NJ) consisting of 15mer peptides overlapping by 11 amino acids spanning the complete HA sequences of H1/Bris, H3/Urug, B/Mal and B/Flo. The markers and the antibodies used for the flow cytometry analysis are detailed in S3 Table. The data acquisition was performed on BD LSRII flow cytometer (Becton Dickinson, Franklin Lakes, NJ). Approximately 2.5x10^5^ viable lymphocytes were acquired for each sample and data were analyzed using FlowJo v9.7 (Tree Star, OR), Pestle v1.7 and SPICE v5.2 (Mario Roederer, Vaccine Research Centre, National Institutes of Health, USA, available at http://exon.niaid.nih.gov/spice). Data were first background subtracted (*i*.*e*.: Antigen stimulated response minus the response in unstimulated cells at each time-point). The net change in each parameter attributable to vaccination was calculated as D21 minus D0. Intracellular cytokine data are presented as the percentage of T cells positive for individual cytokines (*i*.*e*.: Sum IFN-γ, Sum IL-2 and Sum TNF-α with or without another cytokine) as poly-functional T cells (Sum Poly, *i*.*e*. cells synthesizing ≥2 cytokines) and as total responsive cells (Total Response, *i*.*e*. cells synthesizing ≥1 cytokine). The changes between D0 and D21 were assessed by a Wilcoxon matched-pairs signed rank test. For comparisons of T cell frequencies between QVLP and Placebo groups for each influenza strain, the Kruskal-Wallis test was used, followed by Dunn’s multiple comparison post-hoc analysis. The number of subjects included in the CMI analysis did not allow the impact of previous vaccination on the CMI parameters to be addressed.

### Statistical analysis

All calculations were performed using SAS version 9.1 (SAS Institute, Cary, NC) or GraphPad Prism Software (version 6.03, GraphPad Software, La Jolla, CA). A *P* value ≤0.05 was considered as significant.

## Results

### Safety and reactogenicity

In Adults, the incidence of solicited local and systemic reactions through D7 was 28.0%, 36.0%, 49.3% and 65.3% in the Placebo, QVLP15, QVLP30 and QVLP60 groups, respectively (Table 1). Overall none of solicited local or systemic reactions were severe or potentially life-threatening. The most commonly reported solicited local reaction through D7 was pain at injection site (Table 1). The incidence rates of pain at injection site were significantly higher in all QVLP groups compared to Placebo (*P*≤0.0003). The incidence of swelling at injection site was also significantly higher in the QVLP30 and the QVLP60 groups compared to Placebo (*P* = 0.028). All local symptoms were considered vaccine-related. The majority of the solicited local symptoms were mild in severity and either resolved on the same day or the day after immunization. The most commonly reported solicited systemic reactions through D7 were headache, malaise, and fatigue (Table 1). The incidence of headache in the Placebo was significantly higher than in QVLP15 (*P* = 0.027). The incidence of fatigue in QVLP15 was significantly higher than in Placebo (*P* = 0.033). Most solicited systemic reactions were of short duration and were mild in severity. The majority of unsolicited TEAEs were considered by the investigator as mild in severity. These TEAEs resolved with medication and were considered not vaccine-related. No SAEs or NOCDs considered to be related to the VLP vaccine were reported during the study.

**Table 1:** Incidence of local and systemic solicited signs and symptoms through day 7 in Adults (18-49y).

Solicited local and systemic reactions reported through day 7 (Safety Analysis Set)				
	Number of subjects (%)			
	Placebo n = 75	15 μg VLP Vaccine n = 75	30 μg VLP Vaccine n = 75	60 μg VLP Vaccine n = 75
**Local Reactions**				
Erythema at injection site	0	1 (1.3)	1 (1.3)	1 (1.3)
Swelling at injection site	0	1 (1.3)	6 (8.0)^**a**^	6 (8.0)^**a**^
Pain at injection site	5 (6.7)	23 (30.7)^**a**^	33 (44.0)^**a**^	44 (58.7)^**a**^
**Systemic Reactions**				
Fever	1 (1.3)	2 (2.7)	3 (4.0)	2 (2.7)
Fatigue	1 (1.3)	8 (10.7)^**a**^	2 (2.7)	4 (5.3)
Headache	18 (24.0)	7 (9.3)^**b**^	10 (13.3)	10 (13.3)
Muscle aches	2 (2.7)	3 (4.0)	3 (4.0)	5 (6.7)
Malaise	4 (5.3)	6 (8.0)	3 (4.0)	9 (12.0)
Joint aches	1 (1.3)	0	2 (2.7)	2 (2.7)
Chills	0	1 (1.3)	0	5 (6.7)
Swelling in the axilla	0	0	1 (1.3)	1 (1.3)
Swelling in the neck	1 (1.3)	0	2 (2.7)	3 (4.0)

^**a**^ Significantly higher than Placebo.

^**b**^ Significantly lower than Placebo.

In OA≥50, between D0 and D7, a total of 271 (71.2%) subjects who received the QVLP reported at least one solicited local or systemic reaction. Most reactions were mild and of short duration. A small proportion reported moderate reactions and only one QVLP30 subject reported a severe reaction (fatigue considered severe for less than one day). Injection site pain was the most frequently reported local reaction (Table 2) and the numbers of subjects reporting pain were significantly higher in all QVLP groups compared to the Placebo (P<0.0001). Swelling at the injection site was significantly higher than Placebo in QVLP30, QVLP60 and QVLP15+Alum. Muscles aches was significantly higher than Placebo in QVLP groups with the exception of QVLP60. Local injection site reactions (*i*.*e*.: pain, swelling) were generally less frequent at the lower QVLP doses compared to the higher dose levels. Pain or swelling in QVLP15 were significantly less frequent than in QVLP60 (Table 2, *P* = 0.0046 and 0.0484 respectively). QVLP15+Alum subjects also reported significantly more muscle aches than QVLP60 (*P* = 0.0287). The most commonly reported unsolicited AEs within 21 days following vaccination (≥2% of subjects in the combined QVLP groups) were nasopharyngitis, arthralgia, oropharyngeal pain, rhinorrhea, and headache. Only one of these events was observed for >3.5% of subjects (nasopharyngitis; 5.9% of subjects in the combined VLP groups) and all occurred at similar frequencies in the QVLP groups and Placebo. One severe AE considered by the site investigator to be related to the study medication was reported during the study (gout; QVLP15 group); this event started 48 days after vaccination and resolved with treatment within 11 days. No SAEs or NOCDs considered to be related to the VLP vaccine were reported during the study.

**Table 2:** Incidence of local and systemic solicited signs and symptoms through day 7 in older adults (≥50y).

Solicited local and systemic reactions reported through day 7 (Safety Analysis Set)						
	Number of Subjects (%)					
	Placebo (N = 75)	15 μg VLP Vaccine (N = 75)	30 μg VLP Vaccine (N = 75)	60 μg VLP Vaccine (N = 74)	7.5 μg VLP + Al(OH)_3_ (N = 76)	15 μg VLP + Al(OH)_3_ (N = 75)
**Local Reactions**						
Erythema at injection site	0	0	0	3 (4.1)	1 (1.3)	0
Swelling at injection site	0	1 (1.3)	7 (9.3)^**a**^	12 (16.2)^**a**^^**b**^	5 (6.6)^**a**^	6 (8.0)^**a**^^**b**^
Pain at injection site	6 (8.0)	35 (46.7)^**a**^	46 (61.3)^**a**^	52 (70.3)^**a**^^**b**^	48 (63.2)^**a**^	48 (64.0)^**a**^
**Systemic Reactions**						
Fever	0	0	1 (1.3)	0	0	0
Fatigue	9 (12.0)	11 (14.7)	10 (13.3)	14 (18.9)	11 (14.5)	6 (8.0)
Headache	15 (20.0)	8 (10.7)	14 (18.7)	15 (20.3)	12 (15.8)	18 (24.0)
Muscle aches	1 (1.3)	11 (14.7)	12 (16.0)	5 (6.8)	10 (13.2)	15 (20.0)^**c**^
Malaise	4 (5.3)	2 (2.7)	7 (9.3)	7 (9.5)	6 (7.9)	0
Joint aches	4 (5.3)	5 (6.7)	5 (6.7)	3 (4.1)	3 (3.9)	4 (5.3)
Chills	1 (1.3)	3 (4.0)	2 (2.7)	3 (4.1)	4 (5.3)	0
Swelling in the axilla	0	0	0	0	1 (1.3)	1 (1.3)
Swelling in the neck	1 (1.3)	2 (2.7)	0	1 (1.4)	1 (1.3)	0

^**a**^ Significantly higher than Placebo.

^**b**^ Significantly higher than QVLP15

^**c**^ Significantly higher than QVLP60

### The antibody response

#### Homologous strains

**Fig 2:**
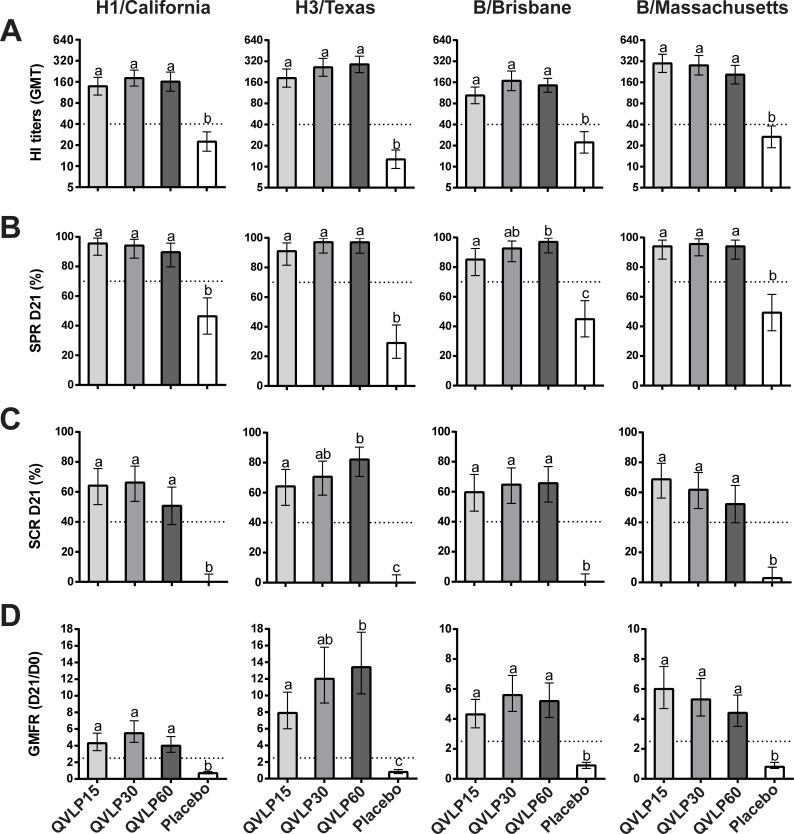
Serum antibody response (HI titers) against the four homologous strains 21 days after vaccination in adults (18-49y). (**A**) Geometric mean titers (GMT ± 95% CI), (**B**) Percent of seroprotection rate (SPR ± 95% CI), (**C**) Percent of seroconversion rate (SCR ± 95% CI) and (**D**) Geometric mean fold increase ratio (GMFR ± 95% CI). Histograms not connected by same letter are significantly different (*P*≤0.05, pair-wise comparison Tukey-Kramer test). The dotted line marks the values of the CHMP criteria.

In Adults, all the QVLP groups displayed significantly higher HI titers, SPR, SCR and GMFR than Placebo for the four homologous strains (Fig 2). No significant differences between the doses were observed except between QVLP15 and QVLP60 for the SCR, the GMFR against H3/Vic and the SPR against B/Bris. All the CHMP criteria, defined as SCR ≥40%, SPR ≥70%, GMFR ≥2.5 [29], were met in the QVLP30 group and no significant differences between QVLP30 and QVLP60 were observed.

**Fig 3:**
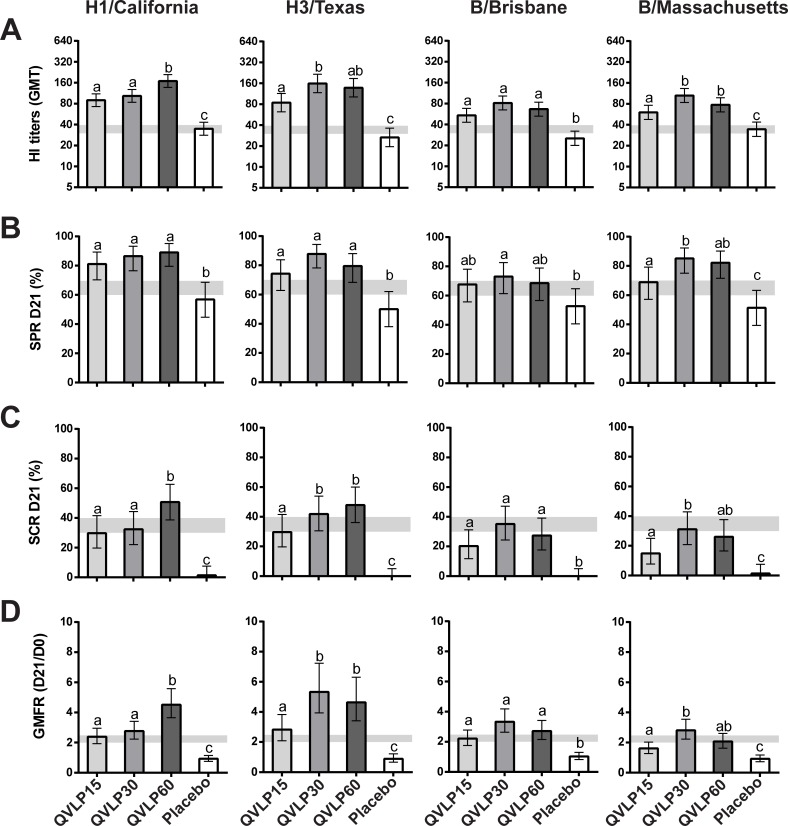
Serum antibody response (HI titers) against the four homologous strains 21 days after vaccination in older adults (≥50y). (**A**) Geometric mean titers (GMT ± 95% CI), (**B**) Percent of seroprotection rate (SPR ± 95% CI), (**C**) Percent of seroconversion rate (SCR ± 95% CI) and (**D**) Geometric mean fold increase ratio (GMFR ± 95% CI). Histograms not connected by same letter are significantly different (*P*≤0.05, pair-wise comparison Tukey-Kramer test). The gray zone marks the values of the CHMP criteria (the upper limit marks the values for adults ≥50y to 64y and the lower limit for adults ≥65y).

In OA≥50, we measured significantly higher HI titers, SPR, SCR and GMFR than Placebo for the four homologous strains with the exception of SPR against B/Bris in QVLP15 and QVLP60 (Fig 3). The addition of alum adjuvant did not significantly improve the Ab response (S1 Fig). There were no significant increase related to the addition of alum in GMTs, SCRs, SPRs and GMFRs for any of the homologous strains between the QVLP15+Alum and the QVLP15 groups. Additionally no beneficial effects were observed between alum adjuvanted groups and the QVLP30. Interestingly, the QVLP7.5+Alum sometimes elicited slightly stronger HI responses for 3 of the 4 homologous strains (all except H1/Cal). The OA≥50 trial was not designed to meet the historical CHMP criteria (*i*.*e*. the number of subjects/group was too low) but rather to determine the best dose to pursue further Phase II studies. Nonetheless, the QVLP30 group met those criteria for the homologous strains with the exception of the SCR which is likely attributable to the impact of prior vaccination.

**Fig 4:**
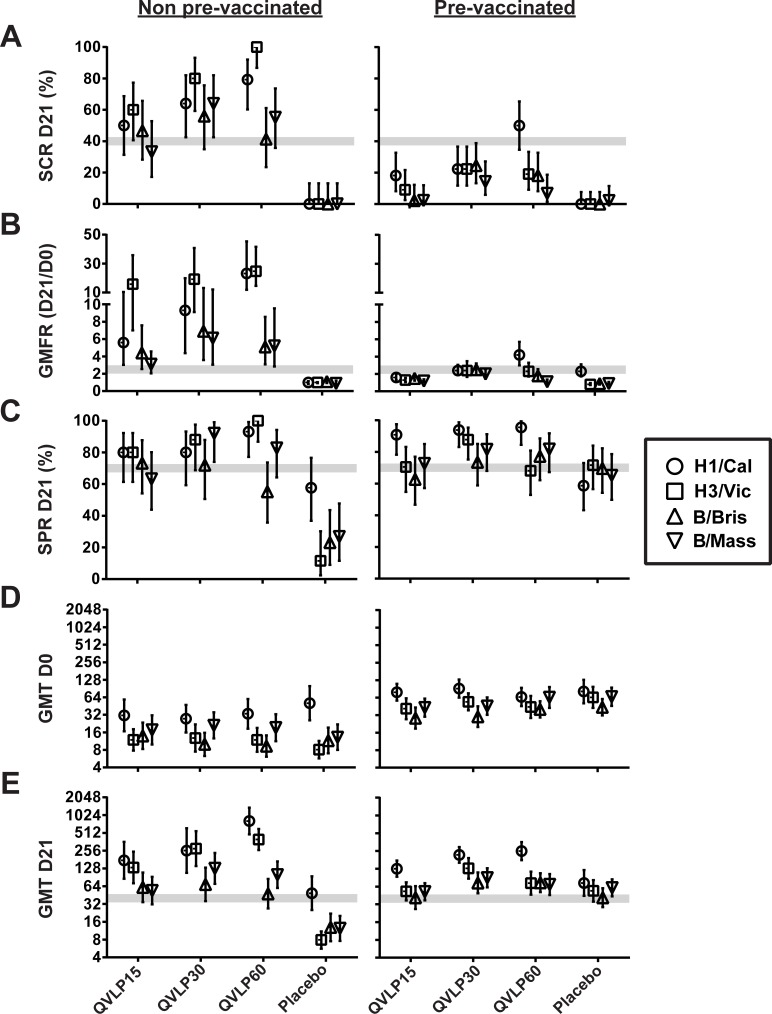
Impact of previous vaccination on serum antibody response (HI titers) against the four homologous influenza strains in older adults (≥50y). (**A**) Percent of seroconversion rate (SCR ± 95% CI), (**B**) geometric mean fold increase ratio (GMFR ± 95% CI) between D0 and D21, (**C**) percent of seroprotection rate (SPR ± 95% CI) at D21, (**D**) geometric mean titer (GMT ± 95% CI) at D0, (**E**) geometric mean titer (GMT ± 95% CI) at D21. The gray zone marks the values of the CHMP criteria (the upper limit marks the values for adults ≥50y to 64y and the lower limit for adults ≥65y).

The impact of prior vaccination in OA≥50 was also investigated. The majority of the OA≥50 subjects had received at least one licensed influenza vaccine during the 2 years prior to study enrolment and these subjects were evenly distributed across groups (60–70.7%; S2 Table). The pre-vaccination status (pre-vaccinated vs none pre-vaccinated) strongly influenced the SCR and the GMFR of the QVLP vaccinated subjects with no impact on the Placebo (Fig 4A and 4B). In contrast, the SPR at D21 was unaffected except for the Placebo group reaching 70% in the pre-vaccinated but remained <40% (except against H1/Cal) in the non-prevaccinated subjects (Fig 4C). These observations most likely resulted from higher pre-existing HI titers at D0 and relatively weaker D21 responses in the pre-vaccinated subjects (Fig 4D and 4E).

#### Heterologous strains

**Fig 5:**
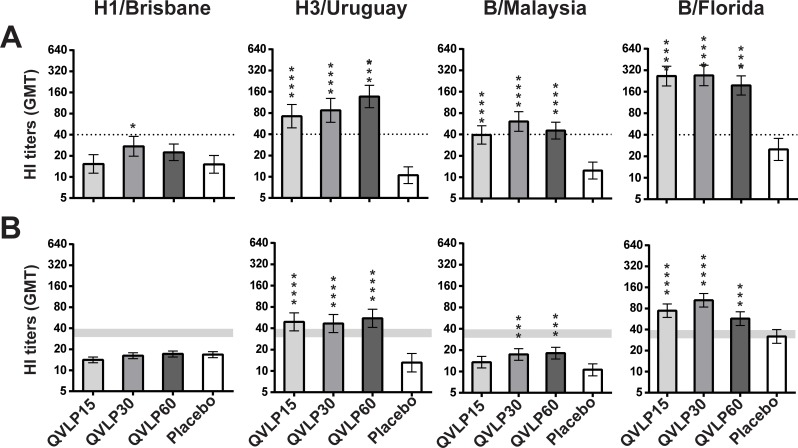
Serum antibody response (HI titers) against the four homologous strains 21 days after vaccination. Geometric mean titer (GMT ± 95% CI) in (**A**) adult (18-49y) and (**B**) older adults (≥50y). Significant differences between vaccinated groups and Placebo are indicated (*P≤0.05, **P≤0.01, ***P≤0.001 pair-wise comparison Tukey-Kramer test). The dotted line or the gray zone (upper limit mark the values for adults ≥50y to 64y, lower limit for adults ≥65y) mark the values of the CHMP criteria.

In Adults, significant increases of HI titers compared to Placebo were observed in QVLP30 for the four heterologous strains (Fig 5A). The other dose level groups showed significant increases for the two B lineage and H3N2 strains.

In OA≥50, there was little to no impact on HI titers for H1/Bris in any group but significant cross-reactivity was seen for the heterologous H3N2 (H3/Urug) and B viruses (B/Mal and B/Flo). The most consistent responses were seen in the QVLP30 and QVLP60 groups and there were no significant differences between these groups (Fig 5B). Again, there was no significant advantage to the addition of alum (S2 Fig).

### The cell-mediated response

#### Homologous response

**Fig 6:**
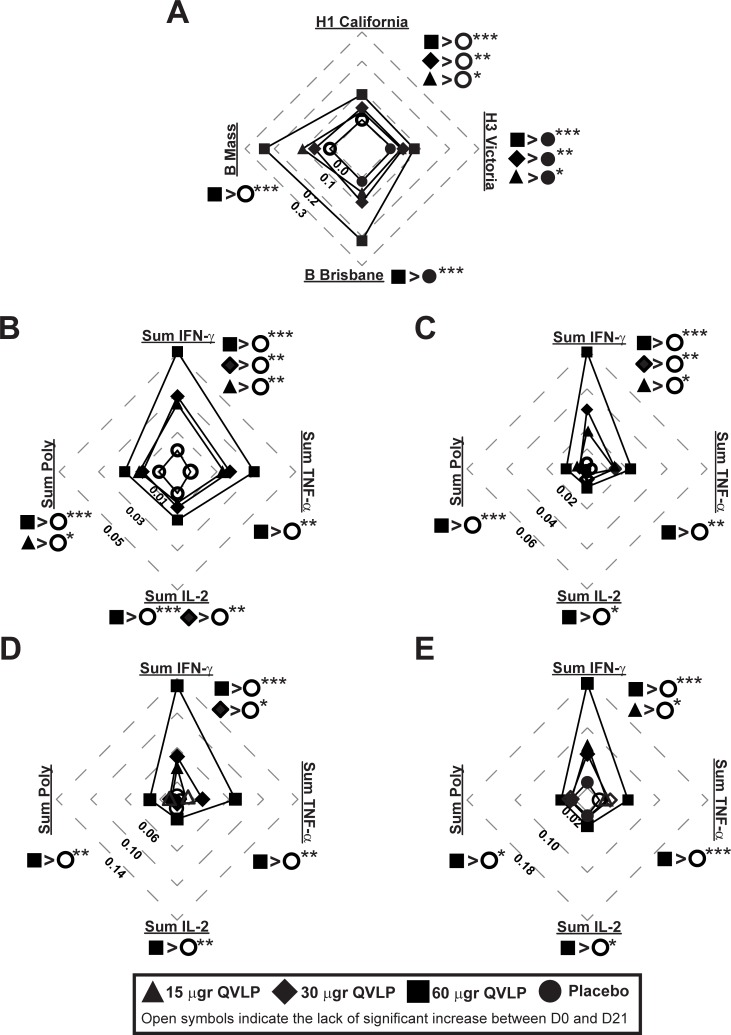
CD4 T cell-mediated immune (% CD4 T cells) against homologous strains in adults (18-49y). Median net changes (D21-D0) are represented. Plain symbols represent significant (*P*≤0.05, Wilcoxon matched-pairs signed rank) increase between D21 and D0 in opposition to open symbols indicating no significant increase of HA-specific CD4^+^ T cells ratio 21 days after vaccination. (**A**) Total response (i.e. the percentage of HA-specific CD4^+^ T cells secreting at least one of the three cytokines IFN-γ, TNF-α, IL-2) after *ex vivo* stimulation with the four VLP include in the vaccine. (**B-E**) Percentage of HA-specific CD4^+^ T cells secreting IFN-γ (Sum IFN-γ), TNF-α (Sum TNF-α), IL-2 (Sum IL-2) or at least two of these three cytokines (Sum poly) after *ex vivo* stimulation with (**B**) H1/Cal VLP, **(C**) H3/Vic VLP, (**D**) B/Bris VLP, (**E**) B/Mass VLP. Significant differences of the median net changes (D21-D0) are reported on each radar graphs (*P≤0.05, **P≤0.01, ***P≤0.01, Kruskal-Wallis test followed by Dunn’s multiple comparisons test).

In Adults, the immunization with QVLP significantly increased the frequencies of HA-specific CD4^+^ T cells producing at least one cytokine (Total Response) between D0 and D21 for the four homologous strains at all tested doses (Fig 6A, filled symbols). The vaccine-induced (D21-D0) HA-specific CD4^+^ Total Response against H1/Cal and H3/Vic were significantly higher in QVLP vaccinated subjects than in Placebo regardless the dose (Fig 6A). The D21-D0 HA-specific CD4^+^ Total Responses were generally higher against the B strains, and QVLP60 induced a significantly higher B strain HA-specific CD4^+^ Total Response than Placebo (Fig 6A). However, the D21-D0 HA-specific CD4^+^ Total Responses in QVLP30 and QVLP60 were not significantly different.

The HA-specific CD4^+^ Sum Total, Sum IFN-γ, Sum TNF-α, Sum IL-2 and Sum Poly significantly increased from baseline (D0) in QVLP30 and QVLP60 21 days after immunization for all the homologous strains (Fig 6B–6E, filled symbols) with the exception of B/Mass-specific Sum TFN-α^+^CD4^+^ T cells in QVLP30 (Fig 6E, open symbols). Compared to Placebo, the QVLP candidate vaccine induced a significant increase of H1 and H3-specific Sum IFN-γ^+^ CD4^+^ T cells at all tested doses and QVLP60 induced a significant increase of Sum IFN-γ, Sum TNF-α, Sum IL-2 and Sum Poly CD4^+^ T cells for the four homologous strains (Fig 6B–6E). Although the sum of each cytokine and Sum Poly responses were generally higher in QVLP60, no significant differences were observed between QVLP60 and QVLP30.

**Fig 7:**
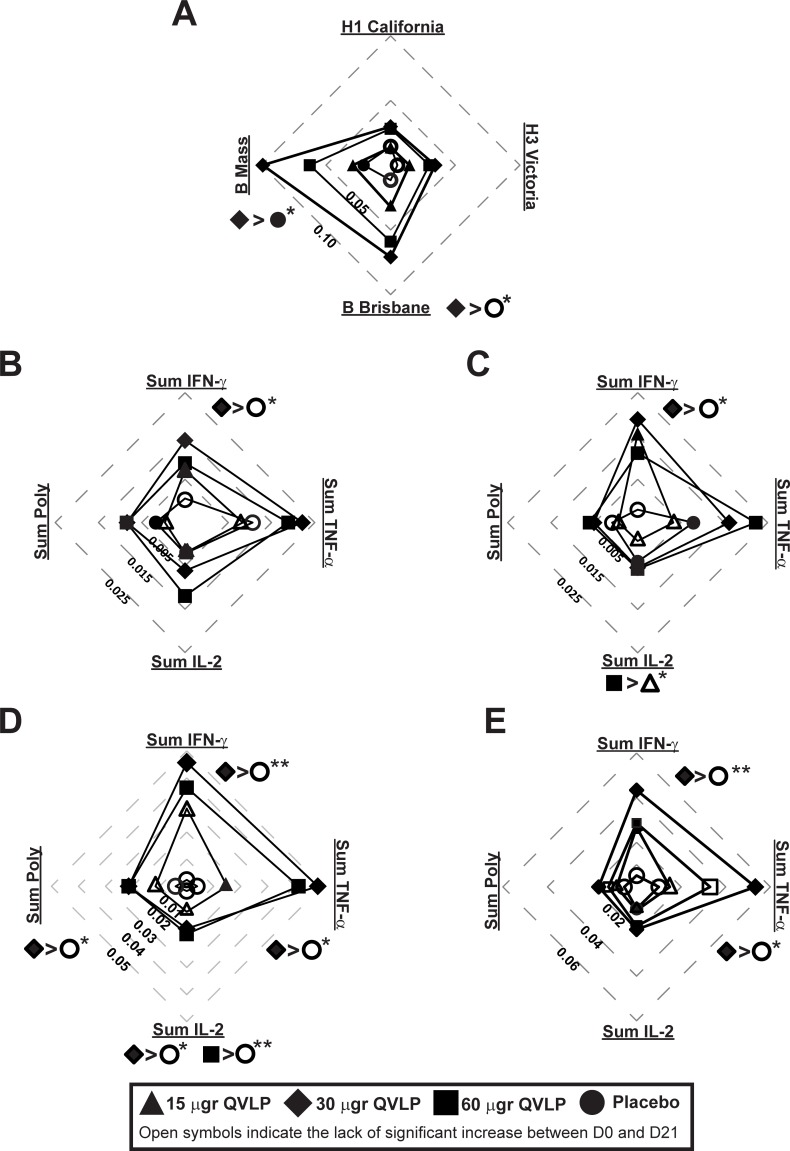
CD4 T cell-mediated immune (% CD4 T cells) against homologous strains in older adults (≥50y). Median net changes (D21-D0) are represented. Plain symbols represent significant (*P*≤0.05, Wilcoxon matched-pairs signed rank) increase between D21 and D0 in opposition to open symbols indicating no significant increase of HA-specific CD4^+^ T cells ratio 21 days after vaccination. (**A**) Total response (i.e. the percentage of HA-specific CD4^+^ T cells secreting at least one of the three cytokines IFN-γ, TNF-α, IL-2) after *ex vivo* stimulation with the four VLP include in the vaccine. (**B-E**) Percentage of HA-specific CD4^+^ T cells secreting IFN-γ (Sum IFN-γ), TNF-α (Sum TNF-α), IL-2 (Sum IL-2) or at least two of these three cytokines (Sum poly) after *ex vivo* stimulation with (**B**) H1/Cal VLP, **(C**) H3/Vic VLP, (**D**) B/Bris VLP, (**E**) B/Mass VLP. Significant differences of the median net changes (D21-D0) are reported on each radar graphs (*P≤0.05, **P≤0.01, ***P≤0.01, Kruskal-Wallis test followed by Dunn’s multiple comparisons test).

In OA≥50, we also observed a significant increase of the HA-specific CD4^+^ Total Responses between D0 and D21 for the four homologous strains at all vaccine doses (Fig 7A, filled symbols). However, only the vaccine-induced HA-specific CD4^+^ Total Response against the B strains in QVLP30 were significantly higher than Placebo (Fig 7A). In contrast to Adults, the higher response was achieved in QVLP30.

The HA-specific CD4^+^ Sum Total, Sum IFN-γ, Sum TNF-α, Sum IL-2 and Sum Poly significantly increased from baseline (D0) in QVLP30 and QVLP60 21 days after immunization (Fig 7B–7E, filled symbols) for all the homologous strains with the exception of B/Mass-specific Sum TFN-α^+^ and Sum Poly CD4^+^ T cells in QVLP60 (Fig 7E, open symbols). As compared to Placebo, QVLP30 elicited a higher Sum IFN-γ^+^ CD4^+^ T cells for the four homologous strains (Fig 7B–7E). That particular group also displayed higher Sum Poly CD4^+^ T cells for B/Bris (Fig 7D) and Sum TNF-α^+^ for the two B strains (Fig 7D–7E) compared to Placebo. We also noted a significantly higher ratio of B/Bris-specific Sum IL-2 CD4^+^ T cells after vaccination with QVLP30 and QVLP60 compared to Placebo (Fig 7D). Whereas the response in Adults was skewed toward IFN-γ production, the response in OA≥50 tended to be dominated by TNF-α^+^ CD4 T cells (Fig 7B–7E).

#### Heterologous response

**Fig 8:**
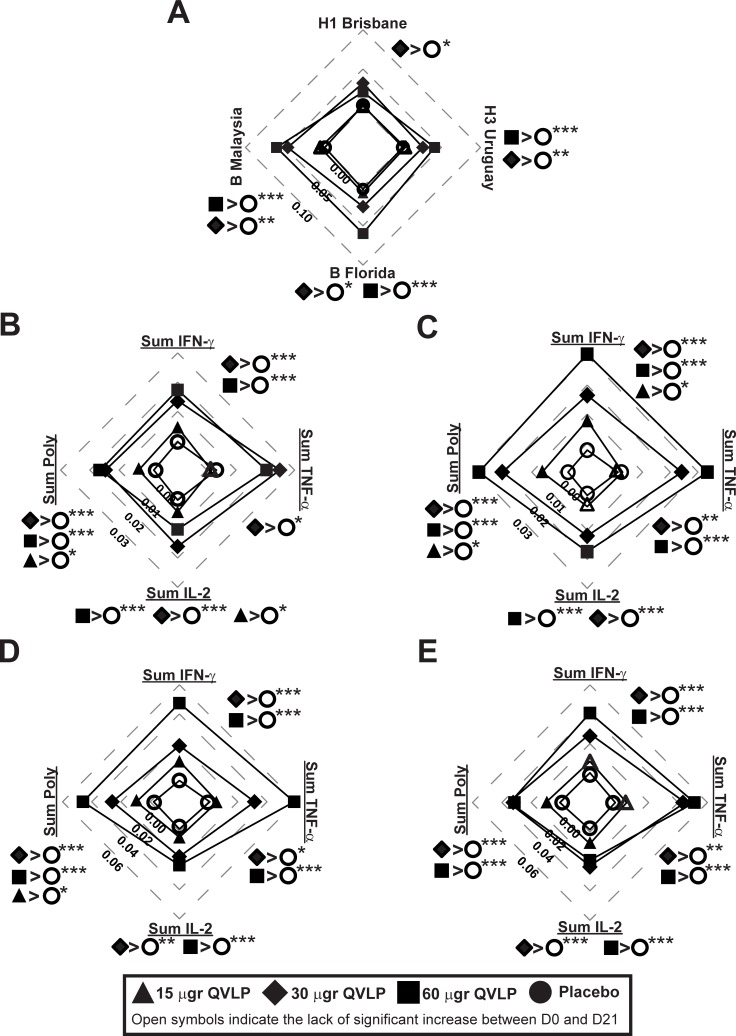
CD4 T cell-mediated immune (% CD4 T cells) against heterologous strains in adults (18-49y). Median net changes (D21-D0) are represented. Plain symbols represent significant (*P*≤0.05, Wilcoxon matched-pairs signed rank) increase between D21 and D0 in opposition to open symbols indicating no significant increase of HA-specific CD4^+^ T cells ratio 21 days after vaccination. (**A**) Total response (i.e. the percentage of HA-specific CD4^+^ T cells secreting at least one of the three cytokines IFN-γ, TNF-α, IL-2) after *ex vivo* stimulation with the peptide pools of 15mer peptides overlapping by 11 amino acids spanning the complete HA sequences of four heterologous strains. (**B-E**) Percentage of HA-specific CD4^+^ T cells secreting IFN-γ (Sum IFN-γ), TNF-α (Sum TNF-α), IL-2 (Sum IL-2) or at least two of these three cytokines (Sum poly) after *ex vivo* stimulation with (**B**) H1/Bris peptide pool, **(C**) H3/Urug peptide pool, (**D**) B/Flo peptide pool, (**E**) B/Malaysia peptide pool. Significant differences of the median net changes (D21-D0) are reported on each radar graphs (*P≤0.05, **P≤0.01, ***P≤0.01, Kruskal-Wallis test followed by Dunn’s multiple comparisons test).

In Adults, QVLP30 and QVLP60 induced a significant increase of the HA-specific CD4^+^ T cell Total Responses between D0 and D21 (Fig 8A, filled symbols). In those two groups, the vaccine-induced (D21-D0) Total Responses were also significantly higher than Placebo with the exception of the QVLP60 against H1/Bris (Fig 8A).

QVLP30 and VLP60 significantly increased HA-specific Sum IFN-γ, Sum TNF-α, Sum IL-2 and Sum Poly CD4^+^ T cells between D0 and D21 for the four strains (Fig 8B–8E, filled symbols) but only QVLP30 induced significantly higher Sum IFN-γ, Sum TNF-α, Sum IL-2 and Sum Poly than Placebo (Fig 8B–8E), QVLP60 failing to promote significantly higher H1/Bris-specific vaccine-induced Sum TFN-α CD4^+^ T cells than Placebo (Fig 8B). Interestingly, the 15 μg dose of QVLP was sufficient enough to significantly increase the HA-specific CD4^+^ polyfunctional T cells (Sum Poly) against H1/Bris, H3/Urug and B/Flo (Fig 8B–8D).

**Fig 9:**
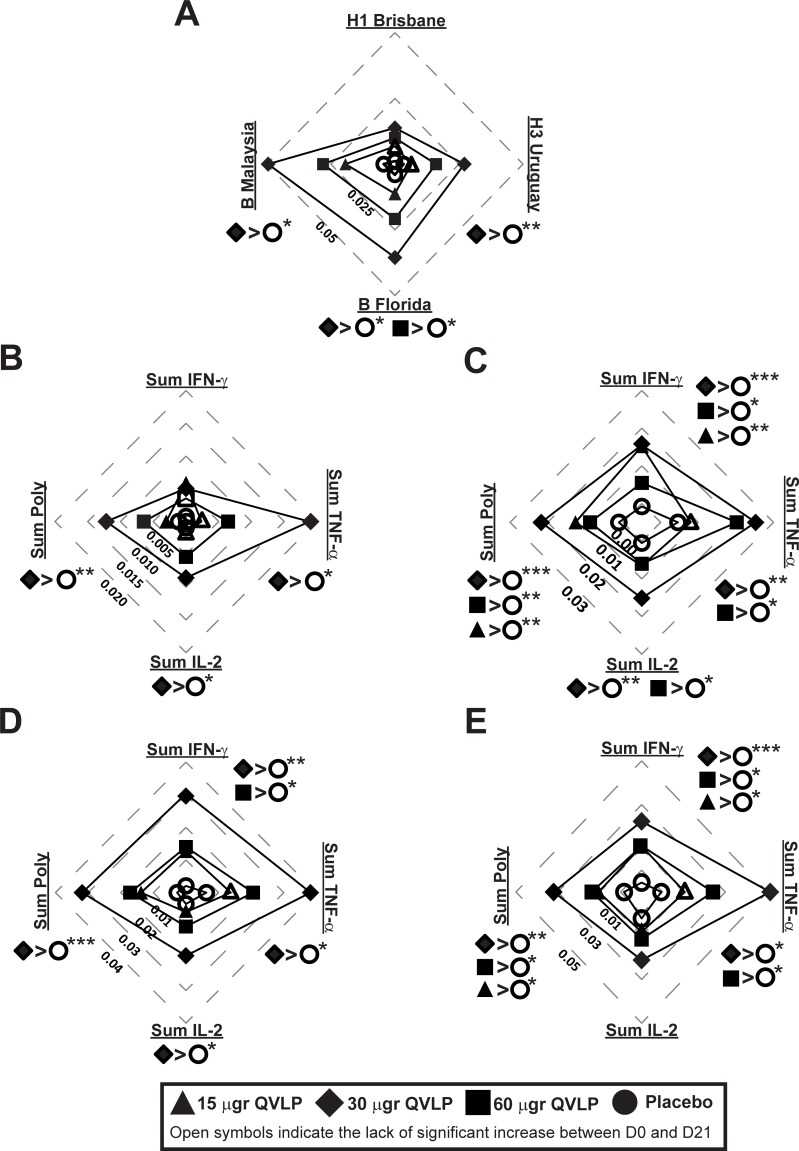
CD4 T cell-mediated immune (% CD4 T cells) against heterologous strains in older adults (≥50y). Median net changes (D21-D0) are represented. Plain symbols represent significant (*P*≤0.05, Wilcoxon matched-pairs signed rank) increase between D21 and D0 in opposition to open symbols indicating no significant increase of HA-specific CD4^+^ T cells ratio 21 days after vaccination. (**A**) Total response (i.e. the percentage of HA-specific CD4^+^ T cells secreting at least one of the three cytokines IFN-γ, TNF-α, IL-2) after *ex vivo* stimulation with the peptide pools of 15mer peptides overlapping by 11 amino acids spanning the complete HA sequences of four heterologous strains. (**B-E**) Percentage of HA-specific CD4^+^ T cells secreting IFN-γ (Sum IFN-γ), TNF-α (Sum TNF-α), IL-2 (Sum IL-2) or at least two of these three cytokines (Sum poly) after *ex vivo* stimulation with (**B**) H1/Bris peptide pool, **(C**) H3/Urug peptide pool, (**D**) B/Flo peptide pool, (**E**) B/Malaysia peptide pool. Significant differences of the median net changes (D21-D0) are reported on each radar graphs (*P≤0.05, **P≤0.01, ***P≤0.01, Kruskal-Wallis test followed by Dunn’s multiple comparisons test).

In OA≥50, QVLP30 and QVLP60 induced a significant increase of the HA-specific CD4^+^ T cell Total Responses between D0 and D21 (Fig 9A, filled symbols). However, only VLP30 has a significant higher vaccine-induced Total Responses against H3/Urug, B/Florida and B/Malaysia as compared to Placebo (Fig 9A).

QVLP30 and VLP60 significantly increased HA-specific Sum IFN-γ, Sum TNF-α, Sum IL-2 and Sum Poly CD4^+^ T cells between D0 and D21 for the four strains (Fig 9B–9E, filled symbols), with the only exception of H1/Bris-specific Sum IFN-γ CD4^+^ T cells in QVLP60 (Fig 9B, open square). As with the homologous H3/Vic, QVLP30 and QVLP60 significantly increased the H3/Urug-specific Sum IFN-γ, Sum TNF-α, Sum IL-2 and Sum Poly CD4^+^ T response as compared to Placebo (Fig 9C). Although QVLP30 dose tended to elicit the strongest responses in OA≥50 while responses in Adults followed a more dose-responding pattern, none of these differences reached statistical significance.

## Discussion

Previous preclinical and *in vitro* studies demonstrated several substantial advantages of the plant-derived influenza VLP vaccines. First, they have all of the advantages of nanoparticulate vaccines in terms of antigen protection, presentation and efficient delivery [20, 31]. We have recently shown that plant-derived VLPs bearing influenza H5 injected into the footpad of a mouse can be found in ~10% of the dendritic cells of the draining lymph node in ~10 minutes [32]. They cluster and activate human immune cells through HA-sialic acid interactions to elicit powerful innate responses in human peripheral blood mononuclear cells within minutes *in vitro* [21]. Once bound to an antigen-presenting cell (APC), they recapitulate most of the early steps of viral entry into endosome, fusion with endosome membranes and intracellular trafficking [22]. The results of the two Phase II studies described herein–the first in healthy adults 18–49 years of age and the second in healthy older adults ≥50 years of age–confirm the potential of the plant-derived QVLP vaccine candidate as an effective tool against seasonal influenza. Although these studies did not include comparator vaccines, safety and reactogenicity profiles of the QVLP were comparable to those reported for commercially-available inactivated influenza vaccines [33–35]. Local effects were mostly mild and transient, with no SAEs. Although the plant origin of QVLP raises the possibility of allergic-type responses, subjects with seasonal allergies were not excluded from these studies and no subject reported worsening of allergy symptoms after vaccination. Furthermore, we have previously shown that individuals exposed to plant-derived VLP vaccines do not produce IgE that target immunopathologic plant glycan motifs [36]. Although concerns about influenza vaccination in egg-allergic subjects has been down-graded in recent years [37, 38], the plant origin of the QLVP vaccine candidate eliminates the egg-associated risk even in those with anaphylaxis. These results confirmed the safety observations in previous trials performed with similar monovalent and multivalent plant-derived VLP candidates [24, 30, 39]. The serologic responses against H1/Cal measured in Adults were very similar to what was previously observed in a Phase I-II trial over a lower dose range [24]. However, both the HI and MN results were more consistent at the higher doses. While high-dose formulations of split-virion and recombinant HA vaccines have been shown to induce higher HI titers in older adults, the gains in efficacy for the elderly have been relatively moderate [8, 40, 41]. Unlike these other vaccines, there was no obvious dose-response with the plant-derived QVLP above the 15 μg/strain dose. In both studies, the greatest response for both the homologous and heterologous strains tested was often seen in the QVLP30 group. Aluminum adjuvants generally have their greatest impact on Ab responses in unprimed populations but older subjects have a lifetime of experience with both natural influenza infections and prior vaccinations [42, 43]. Accordingly, the addition of Alum had no effect on either HI or MN titers in the OA≥50 study. Furthermore, aluminum adjuvants are thought to work at least in part through activation of the inflammasome [44] and elderly subjects are often in a state of chronic, low level inflammation (so-called inflammaging, [45]). Although a number of oil-in-water adjuvants (eg: MF59, ASO3) have been shown to increase antibody titers and cross-reactivity of the antibody response to influenza vaccines across the age range [46, 47] the plant-derived QVLP elicited strong and cross-reactive antibody responses without an adjuvant in both studies. While antibodies constitute the first line of defense against most viruses, engagement of multiple components of the immune system is probably necessary to achieve optimal vaccine-induced protection. Unfortunately, inactivated influenza vaccines are generally poor CMI inducers [48–50]. In contrast, the QVLP candidate vaccine induced significant poly-functional CD4^+^ T cell responses against homologous and heterologous strains in both the Adult and OA≥50 studies. CMI is critical for recovery from and memory against virtually all viral pathogens and natural influenza induces strong CD4^+^ and CD8^+^ T cell responses [51–53]. CD4^+^ T cells are important to support both B and CD8^+^ T cell function in the lung [54] and have been proposed as potential correlates of vaccine protection against influenza [55–57]. Indeed, pre-existing influenza-specific CD4^+^ T cells were recently shown to protect against symptomatic illness in both H3N2 and H1N1 human challenge studies [58]. Interestingly, the frequencies of HA-specific CD4+ T cells elicited by the quadrivalent VLP vaccine in this study were comparable to the levels associated with reduction of clinical signs of infection in children and adults [58–60]. Although not measured in these studies, vaccine-induced CD4^+^ follicular T cells have recently been suggested as good markers of long-term antibody response [61, 62]. While the overall serologic response was similar between the QVLP15 and QVLP30/QVLP60 groups, the CD4^+^ response was significantly improved at the higher QVLP doses, particularly in the OA≥50. Preclinical studies in both ferrets and mice with these plant-derived VLP vaccines have shown their ability to protect both young and old animals, even in absence of any substantial antibody responses [23, 28]. In the younger adults, the CD4^+^ T cell response was dominated to some extent by IFN-γ^+^ cells while the responses in the older adults appeared to be skewed towards TNF-α^+^ cells. As noted above, the trend towards TNF-α production in the older subjects may be a reflection of inflammaging [11, 45, 63]. Although we recently demonstrated that human antigen-presenting cells present HA epitopes on MHC class I after *in vitro* exposure to H1-VLP [64], we did not measure significant changes in Ag-specific CD8^+^ T cells in the Adult nor the OA≥50 studies. However, the timing of sampling (*i*.*e*. 21 days after vaccination) was not optimal for measuring CD8^+^ T cells. Potential CD8^+^ T activation at earlier time point is currently under investigation. Given the number of immunologic parameters measured, the identification of an optimal QVLP dose across the adult age-range is challenging. While most of the homologous and heterologous antibody responses peaked or plateaued at the 30 μg dose, many of the cellular outcomes assessed were slightly higher and/or more consistent at the highest dose (60 μg/strain) in both the Adult and OA≥50 studies. It has already been reported that the optimal vaccine dose for inducing antibodies is not necessarily the same as that for cellular responses, CMI being favored (level and affinity) by lower doses [65, 66]. Based on the data from these two trials, 30 μg of plant-derived VLP influenza vaccine appears to be the best compromise for inducing both strong antibody and cellular responses across all the adult age range. In conclusion, the results obtained in these two Phase II studies support the further development of the seasonal plant-derived QVLP influenza candidate vaccine towards large-scale efficacy studies using a 30 μg dose. Indeed, the results of our first phase III efficacy study in healthy adults (18–64 years of age) will be available in the coming months (NCT03301051). We are hopeful that the ability of the plant-derived QVLP vaccine to elicit not only strong antibody responses but also poly-functional and cross-reactive CD4^+^ T cells will result in a substantial improvement in protection. Additionally the fact that Medicago's QVLP vaccine is based on human HA sequences will be particularly interesting given the global difficulties experienced in 2017–18 with mismatch between the egg-adapted antigens in most of the commercial vaccines and the circulating strains [67, 68].

## Supporting information

S1 Table Subject demographics and baseline characteristics (Safety population) in adults (18-49y). SD: Standard deviation; Min.: Minimum; Max.: Maximum; Am Indian: American Indian or Alaskan Native; Black: Black or African American; Hawaiian: Native Hawaiian or other Pacific Islander. BMI: Body mass index. Placebo is the pooled results of subjects in all cohorts who received the placebo.
Note: Percentages are based on the number of subjects in the Safety Analysis set, with non-missing data within treatment group. Screening data was used to generate this table. Age is calculated as the closest integer result of (Date of Study Day 0—Date of Birth)/365.25; BMI is calculated as Weight (kg)/[Height (m)]^2^.
^a^ P-value for the difference of the number of subjects between treatment groups and two levels of the demographic variable by Fisher’s exact test or Chi-Square test. ^b^ P-value for the difference of the number of subjects between treatment groups and white vs other races by Fisher’s exact test. ^c^ P-value for the difference between treatment groups from an analysis of variance with treatment group of factor. ^d^ P-value for the difference of the number of subjects between treatment groups and influenza immunized vs not immunized by Fisher’s exact test.
^e^ Influenza immunizations received within 24 months prior to the administration of study vaccine.
(DOCX)

S2 Table Subject demographic and baseline characteristics (Safety population) in older adults (>50y). SD: Standard deviation; Min.: Minimum; Max.: Maximum; Am Indian: American Indian or Alaskan Native; Black: Black or African American; Hawaiian: Native Hawaiian or other Pacific Islander. BMI: Body mass index. Placebo is the pooled results of subjects in all cohorts who received the placebo.
Note: Percentages are based on the number of subjects in the Safety Analysis set, with non-missing data within treatment group. Screening data was used to generate this table. Age is calculated as the closest integer result of (Date of Study Day 0—Date of Birth)/365.25; BMI is calculated as Weight (kg)/[Height (m)^2].
^a^ P-value for the difference of the number of subjects among treatment groups by Fisher’s exact test. ^b^ P-value for the difference of the number of subjects among treatment groups and white vs other races by Fisher’s exact test. ^c^ P-value for the difference among treatment groups from an analysis of variance with treatment group as factor. ^d^ P-value for the difference of the number of subjects among treatment groups and influenza immunized vs not immunized by Fisher’s exact test. ^e^ Influenza immunizations received within 24 months prior to the administration of study vaccine.
(DOCX)

S3 Table Immunological markers used for the flow cytometry analysis. (DOCX)

S4 Table CD4 T cell-mediated immune against homologous strains after immunization with adjuvanted QVLP in older adults (≥50y). Median net changes (D21-D0) of HA-specific CD4 T cells (% of CD4) after *ex vivo* stimulation with VLP. Bold values represent significant (*P*≤0.05, Wilcoxon matched-pairs signed rank) increase between D21 and D0.
(DOCX)

S1 Fig Serum antibody response (HI titer) against the four homologous strains 21 days after immunization with adjuvanted QVLP in older adults (≥50y). Responses elicited by 7.5 and 15 μg of the QVLP vaccine adjuvanted with Alum were compared with the unadjuvanted dose of 15 μg QVLP and Placebo. (**A**) Geometric mean titers (GMT ± 95% CI), (**B**) Percent of seroprotection rate (SPR ± 95% CI), (**C**) Percent of seroconversion rate (SCR ± 95% CI) and (**D**) Geometric mean fold increase ratio (GMFR ± 95% CI). Histograms not connected by same letter are significantly different (*P*≤0.05, pair-wise comparison Tukey-Kramer test). The gray zone marks the values of the CHMP criteria (upper limit mark the values for adults ≥50y to 64y, lower limit for adults ≥65y).
(EPS)

S2 Fig Serum antibody response (HI titer) against the four homologous strains 21 days after immunization with adjuvanted QVLP in older adults (≥50y). Responses elicited by 7.5 and 15 μg of the QVLP vaccine adjuvanted with Alum were compared with the unadjuvanted dose of 15 μg QVLP and Placebo (geometric mean titers, GMT ± 95% CI). Histograms not connected by same letter are significantly different (*P*≤0.05, pair-wise comparison Tukey-Kramer test). The gray zone marks the values of the CHMP criteria (upper limit mark the values for adults ≥50y to 64y, lower limit for adults ≥65y).
(EPS)

S1 File Protocol NCT02233816. Clinical study: Immunogenicity, safety, and tolerability of a plant-derived seasonal VLP quadrivalent influenza vaccine in adults.
(PDF)

S2 File Protocol NCT02236052. Clinical study: Immunogenicity, safety, and tolerability of a Plant-derived seasonal VLP quadrivalent influenza vaccine in elderly.
(PDF)

S3 File CONSORT checklist. (DOC)
