# Supplementary material for: Immunogenicity and safety of a quadrivalent plant-derived virus like particle influenza vaccine candidate—Two randomized Phase II clinical trials in 18 to 49 and ≥50 years old adults
Source: PLoS One. 2019 Jun 5;14(6):e0216533. doi: 10.1371/journal.pone.0216533 (PMC6550445; doi:10.1371/journal.pone.0216533)
Supplement: S1 Table — SD: Standard deviation; Min.: Minimum; Max.: Maximum; Am Indian: American Indian or Alaskan Native; Black: Black or African American; Hawaiian: Native Hawaiian or other Pacific Islander. BMI: Body mass index. Placebo is the pooled results of subjects in all cohorts who received the placebo. Note: Percentages are based on the number of subjects in the Safety Analysis set, with non-missing data within treatment group. Screening data was used to generate this table. Age is calculated as the closest integer result of (Date of Study Day 0—Date of Birth)/365.25; BMI is calculated as Weight (kg)/[Height (m)]2. a P-value for the difference of the number of subjects between treatment groups and two levels of the demographic variable by Fisher’s exact test or Chi-Square test. b P-value for the difference of the number of subjects between treatment groups and white vs other races by Fisher’s exact test. c P-value for the difference between treatment groups from an analysis of variance with treatment group of factor. d P-value for the difference of the number of subjects between treatment groups and influenza immunized vs not immunized by Fisher’s exact test. e Influenza immunizations received within 24 months prior to the administration of study vaccine. (DOCX) [file pone.0216533.s001.docx]

**S1 Table**: **Subject demographics and baseline characteristics (Safety Population) in adults (18-49y).**

| **Category** | | **Characteristic** | **15 µg** | **30 µg** | **60 µg** | **Placebo** | **P-Value** |
| --- | --- | --- | --- | --- | --- | --- | --- |
|  |  |  | **(N = 75)** | **(N = 75)** | **(N = 75)** | **(N = 75)** |  |
| **Age (years)** | | **Mean** | **33.9** | **32.5** | **34.5** | **35.8** | **0.1601 ^c^** |
|  |  | **SD** | **9.00** | **8.93** | **8.73** | **9.38** |  |
|  |  | **Min., Max.** | **19, 49** | **18, 49** | **19, 49** | **19, 49** |  |
| **Gender, n (%)** | | **Female** | **42 (56.0)** | **44 (58.7)** | **40 (53.3)** | **43 (57.3)** | **0.9383 ^a^** |
|  |  | **Male** | **33 (44.0)** | **31 (41.3)** | **35 (46.7)** | **32 (42.7)** |  |
| **Ethnicity**  **n (%)** | | **Not Hispanic or Latino** | **26 (34.7)** | **20 (26.7)** | **14 (18.7)** | **14 (18.7)** | **0.0765 ^a^** |
|  |  | **Hispanic or Latino** | **49 (65.3)** | **55 (73.3)** | **61 (81.3)** | **61 (81.3)** |  |
| **Race, n (%)** | | **White** | **51 (68.0)** | **56 (74.7)** | **59 (78.7)** | **64 (85.3)** | **0.0853 ^b^** |
|  |  | **Black** | **24 (32.0)** | **18 (24.0)** | **16 (21.3)** | **11 (14.7)** |  |
|  |  | **Asian** | **0** | **0** | **0** | **0** |  |
|  |  | **Am Indian** | **0** | **0** | **0** | **0** |  |
|  |  | **Hawaiian** | **0** | **0** | **0** | **0** |  |
|  | | **Other** | **0** | **1 (1.3)** | **0** | **0** |  |
| **BMI (kg/m^2^ )** | **Mean** | **26.3** | **24.9** | **25.6** | **26.1** | **0.0834 ^c^** |  |
|  | **SD** | **4.05** | **3.48** | **3.62** | **3.48** |  |  |
|  | **Min., Max.** | **18, 32** | **18, 32** | **19, 32** | **18, 32** |  |  |
| **Influenza Immunization History^e^** | **Yes, n (%)** | **5 (6.7)** | **3 (4.0)** | **3 (4.0)** | **2 (2.7)** | **0.7550 ^d^** |  |
|  | **No, n (%)** | **70 (93.3)** | **72 (96.0)** | **72 (96.0)** | **73 (97.3)** |  |  |

SD: Standard deviation; Min.: Minimum; Max.: Maximum; Am Indian: American Indian or Alaskan Native; Black: Black or African American; Hawaiian: Native Hawaiian or other Pacific Islander.

BMI: Body mass index. Placebo is the pooled results of subjects in all cohorts who received the placebo.

Note: Percentages are based on the number of subjects in the Safety Analysis set, with non-missing data within treatment group. Screening data was used to generate this table.

Note: Age is calculated as the closest integer result of (Date of Study Day 0 - Date of Birth)/365.25; BMI is calculated as Weight (kg)/[Height (m)]^2^.

^a^ P-value for the difference of the number of subjects between treatment groups and two levels of the demographic variable by Fisher’s exact test or Chi-Square test. ^b^ P-value for the difference of the number of subjects between treatment groups and white vs other races by Fisher’s exact test. ^c^ P-value for the difference between treatment groups from an analysis of variance with treatment group of factor. ^d^ P-value for the difference of the number of subjects between treatment groups and influenza immunized vs not immunized by Fisher’s exact test. ^e^ Influenza immunizations received within 24 months prior to the administration of study vaccine.
