# Supplementary material for: Immunogenicity and safety of a quadrivalent plant-derived virus like particle influenza vaccine candidate—Two randomized Phase II clinical trials in 18 to 49 and ≥50 years old adults
Source: PLoS One. 2019 Jun 5;14(6):e0216533. doi: 10.1371/journal.pone.0216533 (PMC6550445; doi:10.1371/journal.pone.0216533)
Supplement: S2 Table — SD: Standard deviation; Min.: Minimum; Max.: Maximum; Am Indian: American Indian or Alaskan Native; Black: Black or African American; Hawaiian: Native Hawaiian or other Pacific Islander. BMI: Body mass index. Placebo is the pooled results of subjects in all cohorts who received the placebo. Note: Percentages are based on the number of subjects in the Safety Analysis set, with non-missing data within treatment group. Screening data was used to generate this table. Age is calculated as the closest integer result of (Date of Study Day 0—Date of Birth)/365.25; BMI is calculated as Weight (kg)/[Height (m)^2]. a P-value for the difference of the number of subjects among treatment groups by Fisher’s exact test. b P-value for the difference of the number of subjects among treatment groups and white vs other races by Fisher’s exact test. c P-value for the difference among treatment groups from an analysis of variance with treatment group as factor. d P-value for the difference of the number of subjects among treatment groups and influenza immunized vs not immunized by Fisher’s exact test. e Influenza immunizations received within 24 months prior to the administration of study vaccine. (DOCX) [file pone.0216533.s002.docx]

**S2 Table**: **Subject demographics and baseline characteristics (Safety Population) in older adults (>50y).**

| **Category** | | **Characteristic** | **15 µg** | **30 µg** | **60 µg** | **7.5 µg  + Al(OH)_3_** | **15 µg  + Al(OH)_3_** | **Placebo** | **P-Value** |
| --- | --- | --- | --- | --- | --- | --- | --- | --- | --- |
|  |  |  | **(N = 75)** | **(N = 75)** | **(N = 74)** | **(N = 76)** | **(N = 75)** | **(N = 75)** |  |
| **Age (years)** | | **Mean** | **64.2** | **63.2** | **63.9** | **64.4** | **63.5** | **63.4** | **0.9486 ^c^** |
|  |  | **SD** | **7.53** | **8.17** | **8.04** | **9.04** | **8.67** | **9.46** |  |
|  |  | **Min., Max.** | **51, 79** | **50, 85** | **50, 83** | **50, 85** | **50, 81** | **50, 87** |  |
| **Gender, n (%)** | | **Female** | **41 (54.7)** | **45 (60.0)** | **42 (56.8)** | **56 (73.7)** | **47 (62.7)** | **51 (68.0)** | **0.1392 ^a^** |
|  |  | **Male** | **34 (45.3)** | **30 (40.0)** | **32 (43.2)** | **20 (26.3)** | **28 (37.3)** | **24 (32.0)** |  |
| **Ethnicity**  **n (%)** | | **Not Hispanic or Latino** | **74 (98.7)** | **75 (100)** | **73 (98.6)** | **76 (100)** | **74 (98.7)** | **75 (100)** | **0.6063 ^a^** |
|  |  | **Hispanic or Latino** | **1 (1.3)** | **0** | **1 (1.4)** | **0** | **1 (1.3)** | **0** |  |
| **Race, n (%)** | | **White** | **75 (100)** | **74 (98.7)** | **72 (97.3)** | **76 (100)** | **75 (100)** | **74 (98.7)** | **0.2170 ^b^** |
|  |  | **Black** | **0** | **1 (1.3)** | **1 (1.4)** | **0** | **0** | **1 (1.3)** |  |
|  |  | **Asian** | **0** | **0** | **1 (1.4)** | **0** | **0** | **0** |  |
|  |  | **Am Indian** | **0** | **0** | **0** | **0** | **0** | **0** |  |
|  |  | **Hawaiian** | **0** | **0** | **0** | **0** | **0** | **0** |  |
| **BMI (kg/m^2^ )** | **Mean** | **26.6** | **26.3** | **26.6** | **25.7** | **26.0** | **25.7** | **0.2794 ^c^** |  |
|  | **SD** | **2.91** | **3.26** | **3.18** | **3.25** | **3.22** | **3.35** |  |  |
|  | **Min., Max.** | **19, 32** | **19, 32** | **20, 32** | **19, 32** | **19, 32** | **18, 32** |  |  |
| **Influenza Immunization History^e^** | **Yes, n (%)** | **45 (60.0)** | **49 (65.3)** | **45 (60.8)** | **51 (67.1)** | **53 (70.7)** | **49 (65.3)** | **0.7630 ^d^** |  |
|  | **No, n (%)** | **30 (40.0)** | **26 (34.7)** | **29 (39.2)** | **25 (32.9)** | **22 (29.3)** | **26 (34.7)** |  |  |

SD: Standard deviation; Min.: Minimum; Max.: Maximum; Am Indian: American Indian or Alaskan Native; Black: Black or African American; Hawaiian: Native Hawaiian or other Pacific Islander.

BMI: Body mass index. Placebo is the pooled results of subjects in all cohorts who received the placebo.

Note: Percentages are based on the number of subjects in the Safety Analysis set, with non-missing data within treatment group. Screening data was used to generate this table.

Note: Age is calculated as the closest integer result of (Date of Study Day 0 - Date of Birth)/365.25; BMI is calculated as Weight (kg)/[Height (m)^2].

^a^ P-value for the difference of the number of subjects among treatment groups by Fisher’s exact test. ^b^ P-value for the difference of the number of subjects among treatment groups and white vs other races by Fisher’s exact test.

^c^ P-value for the difference among treatment groups from an analysis of variance with treatment group as factor.

^d^ P-value for the difference of the number of subjects among treatment groups and influenza immunized vs not immunized by Fisher’s exact test. ^e^ Influenza immunizations received within 24 months prior to the administration of study vaccine.
