# Supplementary material for: Immunogenicity and safety of a quadrivalent plant-derived virus like particle influenza vaccine candidate—Two randomized Phase II clinical trials in 18 to 49 and ≥50 years old adults
Source: PLoS One. 2019 Jun 5;14(6):e0216533. doi: 10.1371/journal.pone.0216533 (PMC6550445; doi:10.1371/journal.pone.0216533)
Supplement: S3 Table — (DOCX) [file pone.0216533.s003.docx]

**S3 Table**: **Immunological markers used for the flow cytometry analysis.**

| **Markers** | **Fluorochromes** | **(Clones) Manufacturers** |
| --- | --- | --- |
| Live/dead  CD14 | V500  V500 | Invitrogen  (M5E2) BD Biosciences |
| CD3 | V450 | (SP34-2) BD Horizon |
| CD4 | Phycoerythrin-TexasRed | (SFCI2T4D11) Beckman Coulter |
| CD8 | BV650 | (RPA-T8) eBiosciences |
| IL-2 | Alexa 700 | (MQ1-17H12) BioLegend |
| IFN-γ | Phycoerythrin-Cyanine7 | (B27) BD Biosciences |
| TNF-α | Allophycocyanin | (6401.1111) BD Biosciences |
