# Supplementary material for: Immunogenicity and safety of a quadrivalent plant-derived virus like particle influenza vaccine candidate—Two randomized Phase II clinical trials in 18 to 49 and ≥50 years old adults
Source: PLoS One. 2019 Jun 5;14(6):e0216533. doi: 10.1371/journal.pone.0216533 (PMC6550445; doi:10.1371/journal.pone.0216533)
Supplement: S4 Table — Median net changes (D21-D0) of HA-specific CD4 T cells (% of CD4) after ex vivo stimulation with VLP. Bold values represent significant (P≤0.05, Wilcoxon matched-pairs signed rank) increase between D21 and D0. (DOCX) [file pone.0216533.s004.docx]

**S4 Table**: **CD4 T cell-mediated immune against homologous strains after immunization with adjuvanted QVLP in older adults (≥50y).**

| H1N1 A/California/07/2009 | | | | | |
| --- | --- | --- | --- | --- | --- |
|  | | QVLP 7.5 μg+Alum | QVLP 15 μg+Alum | QVLP 15 μg | Placebo |
| Sum Total Response | | **0.0289** | **0.0252** | **0.0137** | 0.0144 |
| Sum IFN-γ | | **0.0144** | **0.0098** | **0.0071** | 0.0006**^a^** |
| Sum TNF-α | | **0.0148** | **0.0164** | 0.0083 | 0.0106 |
| Sum IL-2 | | **0.0126** | **0.0069** | **0.0020^a^** | **0.0021** |
| Sum Poly | | **0.0105** | **0.0063** | -0.0004**^a^** | **0.0017** |
| H3N2 A/Victoria/361/11 | | | | | |
|  | QVLP 7.5 μg+Alum | | QVLP 15 μg+Alum | QVLP 15 μg | Placebo |
| Sum Total Response | **0.0289** | | **0.0281** | **0.0146** | 0.0053 |
| Sum IFN-γ | **0.0126** | | **0.0034** | **0.0155** | -0.0020 |
| Sum TNF-α | 0.0189 | | **0.0257** | 0.0032 | **0.0079** |
| Sum IL-2 | 0.0009 | | **0.0052** | -0.0008**^b^** | **0.0040** |
| Sum Poly | 0.0005 | | 0.0060 | -0.0006 | 0.0009 |
| B/Brisbane/60/08 | | | | | |
|  | QVLP 7.5 μg+Alum | | QVLP 15 μg+Alum | QVLP 15 μg | Placebo |
| Sum Total Response | **0.0623** | | **0.1129** | **0.0323** | 0.0116**^b^** |
| Sum IFN-γ | **0.0315** | | **0.0398** | **0.0287** | 0.0024**^b^** |
| Sum TNF-α | **0.0346** | | **0.0764** | **0.0149** | 0.0039**^b^** |
| Sum IL-2 | **0.0186** | | **0.0221** | **0.0094** | 0.0017**^b^** |
| Sum Poly | **0.0155** | | **0.0165** | **0.0116** | 0.0041 |
| B/Massachusetts/02/2012 | | | | | |
|  | QVLP 7.5 μg+Alum | | QVLP 15 μg+Alum | QVLP 15 μg | Placebo |
| Sum Total Response | **0.0691** | | **0.1208** | **0.0268** | **0.0051^b^** |
| Sum IFN-γ | **0.0541** | | **0.0635** | **0.0149** | 0.0100**^a,b^** |
| Sum TNF-α | **0.0314** | | **0.0728** | **0.0105** | 0.0098 |
| Sum IL-2 | **0.0186** | | **0.0215** | **0.0090** | **0.0055** |
| Sum Poly | **0.0239** | | **0.0267** | **0.0292** | 0.0208 |

Median net changes (D21-D0) of HA-specific CD4 T cells (% of CD4) after *ex vivo* stimulation with VLP. Bold values represent significant (*P*<0.05, Wilcoxon matched-pairs signed rank) increase between D21 and D0.**^a^** Significantly lower than 7.5 μg+Alum (P<0.05, Kruskal-Wallis test followed by Dunn’s multiple comparisons test). **^b^** Significantly lower than 15 μg+Alum (P<0.05, Kruskal-Wallis test followed by Dunn’s multiple comparisons test).
