# Supplementary material for: Immunogenicity and safety of a quadrivalent plant-derived virus like particle influenza vaccine candidate—Two randomized Phase II clinical trials in 18 to 49 and ≥50 years old adults
Source: PLoS One. 2019 Jun 5;14(6):e0216533. doi: 10.1371/journal.pone.0216533 (PMC6550445; doi:10.1371/journal.pone.0216533)
Supplement: S1 File — Clinical study: Immunogenicity, safety, and tolerability of a plant-derived seasonal VLP quadrivalent influenza vaccine in adults. (PDF) [file pone.0216533.s007.pdf]

IMMUNOGENICITY, SAFETY, AND TOLERABILITY OF A PLANT-DERIVED SEASONAL VLP QUADRIVALENT INFLUENZA VACCINE IN ADULTS

**PROTOCOL NUMBER:**	CP-Q13VLP-007
**INVESTIGATIONAL PRODUCT:**	Seasonal VLP quadrivalent (H1N1, H3N2, B/Brisbane and B/Massachusetts) vaccine
**DEVELOPMENT PHASE:**	Phase 2a
**SPONSOR:**	Medicago R&D Inc.<br>1020, route de l'Eglise, bureau 600<br>Sainte-Foy, Quebec, Canada, G1V 3V9<br>Tel.: 1.418.658.9393
**PRINCIPAL INVESTIGATORS (PIs):**	Eric Sheldon, MD<br>Miami Research Associates (MRA)<br>6141 Sunset Drive, Suite 301<br>South Miami, Florida, USA, 33143<br>Office: 1.305.598.3125<br>Fax: 1.305.595.9239
	David J. Seiden, MD<br>Broward Research Group (BRG).<br>7261 Sheridan Street, Suite 210<br>Hollywood, Florida, USA, 33020<br>Office: 1.954.374.1150<br>Fax: 1.954.322.1633
**SPONSOR RESPONSIBLE MEDICAL OFFICER:**	Dr. Brian J. Ward<br>Research Institute of the MUHC<br>Montreal General Hospital<br>1650 Cedar Avenue, Room L10-509<br>Montreal, Quebec, Canada, H3G 1A4<br>Pager: 1.514.921.6953
**CRO (Data Management, Biostatistics and Medical Writing):**	INC Research<br>720 King Street West, 7th Floor<br>Toronto, Ontario, Canada, M5V 2T3<br>Tel.: 1.416.963.9338<br>Fax 1.416.963.9732
**PROTOCOL DATE AND VERSION:**	June 17th, 2014 – Final Version 1.0

Information in this protocol is confidential and should not be disclosed, other than to those directly involved in the execution or the ethical review of the study, without written authorization from Medicago R&D Inc. (Herein after known as "Medicago"), and its affiliates. This study will be conducted in accordance with applicable Good Clinical Practice (GCP) guidelines, the United States Code of Federal Regulations (CFR), and International Conference on Harmonization (ICH) guidelines.

TABLE OF CONTENTS

TABLE OF CONTENTS **2**

LIST OF TABLES **5**

LIST OF FIGURES **5**

PROTOCOL AGREEMENT **6**

PROTOCOL SIGNATURE PAGE **7**

SYNOPSIS **8**

LIST OF ABBREVIATIONS **16**

1. INTRODUCTION **17**

1.1. BACKGROUND 17
1.2. BACKGROUND OF THE INVESTIGATIONAL PRODUCT 20
1.3. CLINICAL STUDIES 20
1.3.1 Safety *20*
1.3.2 Antibody response *21*
1.3.3 Results for Plant Glycans *24*
1.4. PRE-CLINICAL STUDIES 30

2. TRIAL OBJECTIVES AND ENDPOINTS **30**

2.1 PRIMARY OBJECTIVES 30
2.1.1 Primary Endpoints *31*
2.2 SECONDARY OBJECTIVES 32
2.2.1 Secondary endpoints and measures *32*
2.3 EXPLORATORY OBJECTIVES 33
2.3.1 Exploratory Endpoints and measures *34*

3. INVESTIGATOR AND TRIAL CENTER **36**

4. ETHICS APPROVAL **37**

4.1 ETHICAL CONDUCT OF THE STUDY 37

5. INVESTIGATIONAL PLAN **37**

5.1 DESCRIPTION OF THE OVERALL TRIAL DESIGN AND PLAN 37
5.1.1 Inclusion Criteria *39*
5.1.2 Exclusion Criteria *40*
5.2 STUDY VISIT PROCEDURES 42
5.2.1 Screening Procedures (up to 30 days prior to study vaccination) *42*
5.2.2 Dosing Visit (Day 0) *43*
5.2.3 Days 1 and 8 following vaccination phone Contact. acceptable interval for Day 1: +1Day; for day 8: -1day/+2 Days. *47*
5.2.4 Day 3 Blood collection (acceptable interval -1 day/+2 days) *48*
5.2.5 Day 21 Visit blood collection (acceptable interval: -2 days/+ 3 days) *49*
5.2.6 Final Visit-Day 201 (acceptable interval is ±14 days) *50*
5.3 SELECTION OF THE STUDY POPULATION 51
5.3.1 Recruitment Procedures *51*
5.3.2 Subject Information and Consent *51*
5.3.3 Temporary Contraindications *51*
5.3.4 Removal of Subjects from Treatment or Assessment *52*

5.4 MODIFICATION OF THE STUDY AND PROTOCOL 54
5.5 INTERRUPTION OF THE STUDY 54

6. TREATMENTS 54

6.1 VACCINES ADMINISTERED 54
6.2 IDENTITY OF INVESTIGATIONAL PRODUCT 54
6.2.1 Study vaccine composition 55
6.2.2 Preparation and Administration of study vaccine 55
6.2.3 Precautions for Use 56
6.2.4 Dose Selection and Timing 56
6.3 IDENTITY AND COMPOSITION OF THE CONTROL PRODUCT 57
6.3.1 Preparation and administration 57
6.3.2 Precautions for use 57
6.4 PRODUCT LOGISTICS 58
6.4.1 Labeling and Packaging 58
6.4.2 Storage and Shipment Conditions 58
6.4.3 Product Accountability 59
6.4.4 Replacement Doses 59
6.4.5 Return of Unused Products 59
6.5 RANDOMIZATION/ALLOCATION PROCEDURES 59
6.6 BLINDING AND CODE BREAKING PROCEDURES 60
6.7 CONCOMITANT THERAPY 60

7. SPECIMENS AND CLINICAL SUPPLIES 61

7.1 MANAGEMENT OF SAMPLES 61
7.1.1 Sample Collection 62
7.1.2 Sample Preparation 62
7.1.3 Sample Storage and Shipment 63
7.2 CLINICAL SUPPLIES 64

8. SERIOUS ADVERSE EVENTS (SAES) AND ADVERSE EVENTS (AES) 64

8.1 REPORTING OF SERIOUS ADVERSE EVENTS (SAEs) 64
8.1.1 Adverse Events 65
8.1.2 Expectedness of an Adverse Drug Reaction 66
8.1.3 Initial SAE Reporting by the Investigator 66
8.1.4 Follow-up Reporting by the PI 67
8.1.5 Reporting of SAEs Occurring after Trial Termination 67
8.1.6 Causal Relationship 67
8.1.7 Reporting of SAEs to Health authorities and IRB 68
8.1.8 Seven (7)-Day Safety Monitoring 68

9. DATA COLLECTION AND MANAGEMENT 70

9.1 DATA COLLECTION, CRF COMPLETION 70
9.2 DATA MANAGEMENT 71

10. STATISTICAL METHODS AND DETERMINATION OF SAMPLE SIZE 71

10.1 GENERAL CONSIDERATIONS 71
10.1.1 Analysis of Primary Safety endpoints 71
10.1.2 Analysis of Primary immunogenicity Endpoints 72
10.1.3 Analysis of Secondary Immunogenicity Endpoints 72
10.1.4 Exploratory Immunogenicity endpoints 73
10.2 ANALYSIS POPULATIONS 73

10.2.1 Definition of Population 73
10.3 DETERMINATION OF SAMPLE SIZE 74
10.4 HANDLING OF MISSING DATA 74

11. ETHICAL AND LEGAL ISSUES AND PI/SPONSOR RESPONSIBILITIES 74

11.1 ETHICAL CONDUCT OF THE TRIAL / GOOD CLINICAL PRACTICE (GCP) 74
11.2 SOURCE DOCUMENTS AND SOURCE DATA 75
11.3 CONFIDENTIALITY OF DATA AND ACCESS TO SUBJECT RECORDS 75
11.4 MONITORING, AUDITING AND ARCHIVING 75
11.4.1 Monitoring 75
11.4.2 Audits and Inspections 76
11.4.3 Archiving 76
11.5 FINANCIAL CONTRACT AND INSURANCE COVERAGE 76
11.6 STIPENDS FOR PARTICIPATION 76

12. REFERENCES 76

LIST OF TABLES

Table 1: Study visit procedures

Table 2: Incidence of systemic and local reactions within 7 days after the vaccination[1]

Table 3: Serum Hemagglutination-Inhibition Antibody Titers (Full set analysis)

Table 4: Serum Neutralizing Antibody Titers (non-audited data)

Table 5: IgE responses to plant glycans according to the bromelain assay

Table 6: Sub-populations of memory T-cell based on the expression of cell-surface markers

Table 7: Sites and Responsible Key Personnel

Table 8: Dose levels to be used in this Phase IIa trial (Immunization on Day 0)

Table 9: Severity grade to be used for Local and Systemic Symptoms and other AEs

LIST OF FIGURES

Figure 1: Relative distribution of the CD4 T cell functional response to ex vivo stimulation with each of the 4 VLPs included in the quadrivalent VLP vaccine.

Figure 2: Details of the functional response of the CD4 T-cell based on the expression of the 4 markers (CD107a, IFN-γ, IL-2 and TNF-α) after *ex vivo* stimulation with each of the 4 VLPs included in the quadrivalent VLP vaccine 21 days after immunization.

Figure 3: Details of the functional response of the TM CD4 T-cells based on the expression of the 4 markers (CD107a, IFN-γ, IL-2 and TNF-α) after *ex vivo* stimulation with each of the 4 VLPs included in the Q-VLP vaccine 21 days after immunization.

Figure 4: Percentage of total cross-reactive CD4 (left) and CD8 (right) T-cell to B/Massachusetts HA protein peptide pool.

Figure 5: Details of the functional response of CD4 (A) and TM CD4 T-cells based on the expression of the 4 markers (CD107a, IFN-γ, IL-2 and TNF-α) after *ex vivo* stimulation with B/Massachusetts HA protein peptide pool 21 days after immunization.

Figure 6: Proposed study schedule with approximate enrolment dates.

PROTOCOL AGREEMENT

I have read this Medicago Protocol No. CP-Q13VLP-007

Title: Immunogenicity, Safety, and Tolerability of a Plant-Derived Seasonal VLP Quadrivalent Influenza Vaccine in Adults

I have fully discussed the objectives of this trial and the contents of this protocol with the Sponsor's representative.

I understand that the information in this protocol is confidential and should not be disclosed, other than to those directly involved in the execution or the ethical review of the study, without written authorization from Medicago R&D Inc. It is, however, permissible to provide information to a subject in order to obtain consent once Investigational Review Board (IRB) approval is obtained.

I agree to conduct this trial according to this protocol and to comply with its requirements, subject to ethical and safety considerations and guidelines, and to conduct the trial in accordance with International Conference on Harmonization (ICH) guidelines. Good Clinical Practice (GCP), USA Investigational New Drug (IND) regulations, and other applicable regulatory requirements.

I understand that the Sponsor may decide to suspend or prematurely terminate this study at any time for whatever reason and that such a decision will be communicated to me in writing. Conversely, should I decide to withdraw from execution of the trial, I will communicate my intention immediately in writing to the Sponsor.

Investigator Name and Address

Eric Sheldon, M.D.
Miami Research Associates (MRA)
6141 Sunset Drive, Suite 301, South Miami, Florida, USA, 33143

Signature Date

David J.Seiden, MD
Broward Research Group (BRG).
7261 Sheridan Street, Suite 210, Hollywood, Florida, USA, 33020

Signature Date

PROTOCOL SIGNATURE PAGE

PROTOCOL TITLE:
Immunogenicity, Safety, and Tolerability of a Plant-Derived Seasonal VLP Quadrivalent Influenza Vaccine in Adults

PROTOCOL NUMBER: CP-Q13VLP-007
PROTOCOL DATE: June 17th , 2014

SIGNATURES:
Protocol Author:

Isabelle-A. Vandandaigue, CRA, Medicago	Date

Protocol Reviewed by:

Eric Sheldon, MD, Principal Investigator, MRA	Date
David J.Seiden, MD, Principal Investigator, BRG	Date
Cindy Lui, Biostatistician	Date
Brian J. Ward, MD, Sponsor Responsible, Medical Officer	Date
Sonia Trepanier, Senior Director, Clinical Studies	Date

Protocol Authorized by:

Nathalie Landry, Vice-president, Product Development	Date
Irene Clement, Vice-president, Regulatory Affairs	Date

SYNOPSIS

COMPANY:	MEDICAGO R & D INC.
INVESTIGATIONAL PRODUCT/ DRUG PRODUCT:	PLANT-DERIVED SEASONAL VIRUS-LIKE PARTICLES (VLP) QUADRIVALENT INFLUENZA VACCINE (WILL BE REFERRED TO SEASONAL VLP INFLUENZA VACCINE)
ACTIVE SUBSTANCE(S):	MIX OF RECOMBINANT H1, H3, AND 2B PROTEINS [HEMAGGLUTININ (HA)] EXPRESSED AS VLPS FOR THE 4 INFLUENZA STRAINS RECOMMENDED BY WHO FOR VACCINATION IN THE NORTHERN HEMISPHERE IN 2013-2014 AS FOLLOWS:<br>• The H1 VLP Influenza vaccine contains an HA protein for which the sequence is derived from the A/California/7/2009 H1N1 virus.<br>• The H3 VLP Influenza vaccine contains an HA protein for which the sequence is derived from the wild-type A/Victoria/361/2011 H3N2 virus.<br>• The B VLP Influenza vaccine contains an HA protein for which the sequence is derived from the:<br>-B/Massachusetts/2/2012 virus (Yamagata lineage) and<br>-B/Brisbane/60/2008 virus (Victoria lineage).
TITLE OF THE TRIAL:	Immunogenicity, Safety, and Tolerability of a Plant-Derived Seasonal VLP quadrivalent Influenza Vaccine (Seasonal VLP Influenza Vaccine) in Healthy Adults
BRIEF SUMMARY:	This is a multiple sites (at least 2 clinical sites) phase 2a, randomized, observer-blind, dose ranging, placebo-controlled clinical study to evaluate the immunogenicity, safety, and tolerability of a single intramuscular injection of plant-based Seasonal VLP quadrivalent Influenza Vaccine administered to healthy adults. A total of three hundred (300) subjects will be randomized in four (4) groups of 75 subjects. This study will be a cohort staggering (slow enrolment) for 3 dose levels (15 µg, 30 µg, and 60 µg VLP per strain) and a placebo-controlled group (1:1:1:1 ratio).<br>The 300 subjects (male and female subjects, 18 to 49 years of age) will be divided in 3 different cohorts (Refer to Section 6.2.4 for details on composition of each cohort). The 7-day safety data after the immunization will be collected by the clinical staff and will be reviewed by the Data and Safety Monitoring Board (DSMB). The DSMB members will determine if the clinical sites are allowed to continue immunization of the next cohort.<br>Three (3) and 21 days after immunization, key safety (Day 3) and immunogenicity (Day 21) data will be collected and analyzed. All subjects will be followed for safety until Day 201 visit (6-month follow up).
DEVELOPMENT PHASE:	Phase 2a
INVESTIGATORS AND TRIAL CENTER:	Eric Sheldon, MD,<br>Miami Research Associates (MRA)<br>6141 Sunset Drive, Suite 301<br>South Miami, FL 33143,USA<br>Office: 1.305.598.3125<br><br>David J.Seiden<br>Broward Research Group (BRG).<br>7261 Sheridan Street, Suite 210<br>Hollywood, Florida, USA, 33020<br>Office: 1.954.374.1150
EXPECTED START DATE:	August 2014
EXPECTED COMPLETION DATE:	September (October) 2014: for immunization (Day 0) and blood sampling at Day 21.<br>January 2015: report including results up to Day 21<br>April (May) 2015: final follow-up safety visit expected.
PLANNED TRIAL PERIOD:	Total planned trial period: Approximately 8 months, from screening procedures to Day 201 visit.

<table>
<tr><td>PRIMARY OBJECTIVE:</td><td>The primary objective of this study is to assess the immunogenicity, safety and tolerability of one dose of a Plant-Derived Seasonal VLP quadrivalent Influenza Vaccine given at 3 dose levels: 15 µg, 30 µg, and 60 µg/strain compared to a placebo.</td></tr>
<tr><td>PRIMARY ENDPOINTS:</td><td><u>Immunogenicity</u>
For the HI test against the homologous influenza vaccine strains:
Geometric mean titers (GMTs) of Hemagglutination Inhibition (HI) antibody for H1N1, H3N2, and B (B/Bris and B/Mass) vaccine strains on Days 0 and 21. Follow-up serology samples for GMTs will be collected at Day 201. GMTs will be analyzed as follows:
Seroconversion factor or geometric mean fold rise (GMFR): is the geometric mean of the ratio of GMTs (Day 21/Day 0).
Seroconversion rate: the proportion of subjects in a given treatment group with either a ≥4-fold increase in reciprocal HI titers between Day 0 and Day 21 or a rise of undetectable HI titer (i.e. <10) pre-vaccination (Day 0) to an HI titer of ≥40 at Day 21 post-vaccination.
Seroprotection rate: the proportion of subjects in a given treatment group attaining a reciprocal HI titer of ≥40 at Day 21 post-vaccination (the percentage of vaccine recipients with a serum HI titer of at least 1:40 following vaccination).
<u>Safety and Tolerability</u>
Percentage, intensity, and relationship of immediate complaints (30 minutes post-vaccination).
Percentage, intensity, and relationship to vaccination of solicited local and systemic signs and symptoms (for 7 days following study vaccine).
Percentage, intensity, and relationship of unsolicited local and systemic signs and symptoms (for 21 days following study vaccine)
Occurrences of all adverse events (AEs)/serious adverse events (SAEs).
Occurrences of new onset of a chronic disease (NOCD)
The number and percentage of subjects with normal and abnormal urine, haematological, and biochemical values at Screening, Days 3, and 201.</td></tr>
<tr><td>STATISTICAL METHODS FOR PRIMARY ENDPOINTS:</td><td><u>Immunogenicity</u>
Immunogenicity will be evaluated by the immune response induced in subjects as measured by the serum HI antibody titers at specified timepoints. Immunogenicity endpoints (including GMTs) will be compared between vaccine dose groups and also between each vaccine dose group to placebo.

Seroconversion and seroprotection rates will be compared using Fisher's exact tests at Day 21. The difference and the 95 % CI on the difference between the proportions of seroconverted or seroprotected subjects in each dose group compared to placebo will be assessed using Fisher's exact method. The lower bound of the 95% confidence limit for the percentage subjects achieving seroconversion or seroprotection will be compared to CBER's criteria. Refer to Section 2.1 for details on CPMP and CBER's criteria and to section 10.3 for determination of sample size.

<u>Safety and Tolerability</u>
Safety and tolerability will be evaluated through reported AEs, history/symptom-directed physical examination findings, clinical laboratory results, and vital signs. Safety endpoints will be compared (each vaccine dose level versus placebo and also between vaccine dose groups) using Fisher's exact tests.</td></tr>
</table>

**SECONDARY OBJECTIVES:**	The secondary objectives are as follow:<br>• To assess the reactivity of antibodies induced by one dose of seasonal VLP quadrivalent influenza vaccine at the three dose levels 15 µg, 30 µg, and 60 µg/strain compared to the placebo, as measured by HI antibody titers for the homologous influenza strains. This will be assessed using measures of percentage of subjects with a detectable HI antibody response (≥lower limit of quantification; ≥LOQ) at Days 0, 21, and 201.<br>• To assess the reactivity of antibodies induced by one dose of VLP quadrivalent influenza vaccine at the three dose levels 15 µg, 30 µg, and 60 µg/strain, compared to the placebo, as measured by microneutralisation (MN) antibody titers for the homologous influenza strains. This will be assessed using measures of GMFR, seroconversion rate, and of percentage of subjects with a detectable MN antibody response (≥LOQ) at Days 0, 21, and 201.<br>• To assess the cross-reactivity of antibodies induced by one dose of VLP quadrivalent influenza vaccine at the three dose levels 15 µg, 30 µg, and 60 µg/strain, compared to the placebo, as measured by HI and MN antibody titers for heterologous influenza strains. This will be assessed using measures of GMFR, seroconversion rate, and seroprotection rate (for HI only) and of the percentage of subjects with a detectable HI or MN antibody response (≥ LOQ) at Days 0, 21, and 201.
**SECONDARY ENDPOINTS:**	For the HI test (against the homologous influenza vaccine strains):<br>GMTs of HI antibody for H1N1, H3N2, and B (B/Bris and B/Mass) vaccine strains on Days 0 and 21. Follow-up serology samples for GMTs will be collected at Day 201. GMTs will be analyzed as follows:<br>• The percentage of subjects with a detectable HI antibody response (≥LOQ) at Days 0, 21, and 201 against the H1N1, H3N2, and B (B/Bris and B/Mass) vaccine strains.<br>For the HI test (against heterologous influenza vaccine strains):<br>GMTs of HI antibody for heterologous influenza strains on Days 0 and 21. Follow-up serology samples for GMTs will be collected at Day 201. GMTs will be analyzed as follows:<br>• Seroconversion factor or GMFR: is the geometric mean of the ratio of GMTs (Day 21/Day 0).<br>• Seroconversion rate: the proportion of subjects in a given treatment group with either a ≥4-fold increase in reciprocal HI titers between Day 0 and Day 21 or a rise of undetectable HI titer (i.e. <10) pre-vaccination (Day 0) to an HI titer of ≥40 at Day 21 post-vaccination.<br>• Seroprotection rate: the proportion of subjects in a given treatment group attaining a reciprocal HI titer of ≥40 at Day 21 post-vaccination (the percentage of vaccine recipients with a serum HI titer of at least 1:40 following vaccination).<br>• The percentage of subjects with a detectable HI antibody response (≥LOQ) at Days 0, 21, and 201 against other influenza vaccine strains.

**SECONDARY ENDPOINTS (CONT'D):**	For the MN test (against the homologous and heterologous influenza strains):<br>GMTs of MN antibody for homologous and heterologous H1N1, H3N2, and B (B/Bris and B/Mass) strains on Days 0 and 21. Follow-up serology samples for GMTs will be collected at Day 201. GMTs will be analyzed as follows:<br>• Seroconversion factor or GMFR: is the geometric mean of the ratio of GMTs (Day 21/Day 0).<br>• Seroconversion rate: the proportion of subjects in a given treatment group with either a ≥4-fold increase in reciprocal MN titers between Day 0 and Day 21 or a rise of undetectable MN titer (i.e. <10) pre-vaccination (Day 0) to an MN titer of ≥40 at Day 21 post-vaccination.<br>The percentage of subjects with a detectable MN antibody response (≥LOQ) at Days 0, 21, and 201.
**STATISTICAL METHODS FOR SECONDARY ENDPOINTS:**	Seroconversion factor or GMFR will be compared between active dose groups and also between each active dose group to placebo using analysis of variance (ANOVA) methods. A multi-stage test (i.e., Dunnett's test and Tukey's correction) will be used to evaluate differences among the treatment groups.<br>Seroconversion rates and seroprotection rates (for HI only) will be compared (i.e., each vaccine dose level versus placebo and also between active dose groups) using Fisher's exact tests at Day 21. The difference and the 95% CI on the difference between the proportions of seroconverted or seroprotected subjects in each dose group compared to placebo will be calculated using Fisher's exact method.
**EXPLORATORY ENDPOINTS:**	Cell-mediated immune (CMI) response in PBMCs (in 15 subjects per treatment group, for a total of 60 subjects)<br>• To characterize the T cell response induced by vaccination (Days 0, 21, and 201).<br>Humoral response<br>• To measure the increase in sera IgE antibodies directed to plant-specific glycans (timeframe: Days 0, 21, and 201).
**STATISTICAL METHODS FOR EXPLORATORY ENDPOINTS**	When applicable for Exploratory endpoints and immunogenicity endpoints will be compared (each vaccine dose level versus placebo and between each vaccine group) using analysis of variance methods for continuous endpoints and Fishers' exact tests or chi-square tests for categorical endpoints. Non-parametric methods will be utilized if the continuous data does not meet normality assumptions.
**ADMINISTRATION AND DURATION OF TREATMENT:**	One intra-muscular (IM) injection on Day 0 of the Seasonal VLP quadrivalent Vaccine or placebo into the deltoid muscle of the non-dominant arm (preferably). The volume of injection is 0.5 mL for all dose levels with the exception of the 60 µg per strain (240 µg total) for which the volume of injection will be 1.0 mL.
**INVESTIGATIONAL PRODUCT, DOSE, LOT NUMBERS:**	Seasonal VLP quadrivalent Influenza vaccine, a Plant-Derived candidate Influenza Vaccine administered by IM injection of 15 µg, 30 µg, or 60 µg, of each strain.<br>The four influenza recombinant HA proteins of the VLP quadrivalent vaccine are based on the following human influenza strains:<br>• A/California/7/2009 (H1N1)<br>• A/Victoria/361/2011 (H3N2)<br>• B/Brisbane/60/2008 (B/Bris)<br>• B/Massachusetts/2/2012 (B/Mass)<br>The name in parenthesis will be used in the protocol to simplify the text.

**INVESTIGATIONAL PRODUCT, DOSE, LOT NUMBERS (CONT'D):**	The Seasonal VLP Influenza Vaccine lot numbers are defined below:<br>Vaccine at concentration of 30 µg/mL:<br>- Lot number: AAHAW01-140016-011<br>Vaccine at concentration of 60 µg/mL:<br>Lot number: AAHAW01-140014-011
**CONTROL PRODUCT (PLACEBO):**	Phosphate buffered saline (PBS; Placebo):100 mM phosphate buffer + 150 mM sodium chloride + 0.01% Tween 80.<br>Lot number: PPPXX01-130005-013
**NUMBER OF SUBJECTS AND TREATMENT GROUPS**	Total planned subjects: 300 subjects randomized into 4 groups in a 1:1:1:1 ratio:<br>Group 1: 75 subjects administered (IM) 15 µg per strain of the Seasonal VLP vaccine<br>Group 2: 75 subjects administered (IM) 30 µg per strain of the Seasonal VLP vaccine<br>Group 3: 75 subjects administered (IM) 60 µg per strain of the Seasonal VLP Vaccine<br>Group 4: 75 subjects administered (IM) placebo (PBS)
**SAFETY ASSESSMENTS**	Clinical safety assessments will include vital signs (including oral temperature), safety clinical laboratory testing (blood chemistry and haematology testing and urinalysis at screening and days 3 and 201), and history/symptom-directed physical examinations.<br>In addition, a phone contact/memory aid (e.g. diary card) follow-up to collect solicited local and systemic symptoms and unsolicited AEs, SAEs, visits to healthcare providers, hospitalizations and any new medications taken or changes in concomitant medication will be performed at Days 1 and 8 following vaccination. Between Day 21 and Day 201 visits, any AEs/SAEs experienced during the study will be recorded on subjects' memory aid.
**INCLUSION CRITERIA:**	1) Male and female adults, 18 to 49 years of age, inclusive.<br>2) Body mass index (BMI) of ≥18 and ≤32.<br>3) Give his/her consent to participate in this study (by signing the informed consent form [ICF]). In the opinion of the Investigator, competence and willingness to provide written, informed consent for participation after reading the ICF. The subject must have adequate opportunity to discuss the study with an Investigator or qualified designee.<br>4) Healthy as judged by the Investigator or designee and determined by complete general physical examination, vital signs, clinical laboratory tests, and medical history conducted no more than 30 days prior to study vaccine administration. Subjects with a pre-existing chronic disease will be allowed to participate if the disease is stable and, according to the Investigator's judgment, the condition is unlikely to confound the results of the study or pose additional risk to the subject by participating in the study. Stable disease is generally defined as no new onset of exacerbation of pre-existing chronic disease 6 months prior to immunization. Based on the Investigator's judgment, a subject with more recent stabilization of a disease could also be eligible.<br>5) Comprehension of the study requirements, expressed availability for the required study period, ability to attend scheduled visits, accessible by phone on a consistent basis.<br>6) If female, have a negative serum pregnancy test result at screening and negative urine pregnancy test on Day 0 prior to immunization.

**INCLUSION CRITERIA (CONT'D):**	7) Female of childbearing potential must use an effective method of contraception for 1 month prior to immunization and agrees to continue employing adequate birth control measures for at least 60 days post-immunization. Moreover, she must have no plan to become pregnant for at least 2 months post-immunization. Abstinent subjects should be asked what method(s) they would use, should their circumstances change, and subjects without a well-defined plan should be excluded.<br>The following relationship or methods of contraception are considered to be effectives:<br>• Hormonal contraceptives (e.g., injectable, topical [patch], estrogenic vaginal ring, etc.);<br>• Intra-uterine device (IUD) with or without hormonal release;<br>• Male partner using a condom plus spermicide or sterilized partner (at least 1 year prior to immunization);<br>• Credible history of abstinence (self-reported);<br>• Heterosexual abstinence at least 60 days post-immunization;<br>• Female partner.<br>8) Non-childbearing females defines as:<br>• Surgically-sterile (defined as bilateral tubal ligation or hysterectomy performed more than 1 month prior to immunization);<br>• Post-menopausal (absence of menses for 24 consecutive months and age consistent with natural cessation of ovulation).
**EXCLUSION CRITERIA:**	1) According to Investigator's opinion, presence of significant acute or chronic, uncontrolled medical or neuropsychiatric illness. "Uncontrolled" is defined as:<br>• Requiring a new medical or surgical treatment within one month prior to study vaccine administration;<br>• Requiring a change in medication dosage in one month prior to study vaccine administration due to uncontrolled symptoms or drug toxicity (elective dosage adjustments in stable subjects are acceptable).<br>2) Any medical or neuropsychiatric condition or any history of excessive alcohol use or drug abuse which, in the Investigator's opinion, would render the subject unable to provide informed consent or unable to provide valid safety observations and reporting.<br>3) Any autoimmune disease or any confirmed or suspected immunosuppressive condition or immunodeficiency including history of human immunodeficiency virus (HIV) infection, Hepatitis B or C, or the presence of lymphoproliferative disease.<br>4) Administration of any vaccine (including any other influenza vaccine) within 30 days prior to study enrolment or planned administration within the period from the vaccination up to blood sampling at Day 21 or within 30 days prior to blood sampling at Day 201. Immunization on an emergency basis will be evaluated case-by-case by the Investigator.<br>5) Administration of any adjuvanted or investigational influenza vaccine within 1 year prior to the study enrolment or planned administration prior to the end of this study (Day 201). Administration of any 'standard', not adjuvanted influenza vaccine (e.g.: live attenuated TIV/QIV vaccine IN or split TIV/QIV vaccine by either intra-dermal or IM route) prior to the 30 day exclusion period mentioned above would be acceptable.

**EXCLUSION CRITERIA (CONT'D):**	6) Use of any investigational or non-registered product within 30 days prior to study enrolment or planned use during the study period. Subjects may not participate in any other investigational or marketed drug study while participating in this study (approximately 8 months).<br>7) Treatment with systemic glucocorticoids at a dose exceeding 10 mg of prednisone per day, or equivalent for more than 7 consecutive days or for 10 or more days in total, within one month of study vaccine administration, any other cytotoxic or immunosuppressant drug, or any immunoglobulin preparation within 3 months of vaccination. Low doses of nasal or inhaled glucocorticoids are allowed.<br>8) Any significant disorder of coagulation or treatment with warfarin derivatives or heparin. Persons receiving prophylactic anti-platelet medications (e.g., low-dose aspirin [no more than 325 mg/day]), and without a clinically apparent bleeding tendency are eligible. Subjects treated with new generation drugs that will not increase risk of intramuscular bleeding (such as clopidogrel) are also eligible.<br>9) History of allergy to any of the constituents of the VLP quadrivalent (including H1N1, H3N2, B/Bris, and B/Wis) study vaccine, or to the phosphate buffered saline (PBS; used as placebo), or tobacco allergy.<br>10) History of anaphylactic allergic reactions to any food, medication or bee sting.<br>11) Any history of asthma (eg: status asthmaticus, hospitalization for asthma control) or recurrent asthma episodes requiring medical attention in the last 3 years (≥ 1 episode/year)<br>12) Continuous use of anti-histamines in the last 4 weeks prior to immunization or use of anti-histamines 48 hours prior to study immunization.<br>13) Have a rash, dermatological condition, tattoos, muscle mass or any other abnormalities at injection site which may interfere with injection site reaction rating.<br>14) Have received a blood transfusion within 90 days prior to study vaccination.<br>15) If female, have a positive or doubtful pregnancy test result prior to immunization or lactating females.<br>16) Vital sign abnormalities: systolic blood pressure ≥150 mmHg and/or diastolic blood pressure ≥90mmHg, heart rate ≤40 beats/min and ≥100 beats/min. Although a vital signs measurement is out of the acceptable ranges, a subject may be included in the study based on Investigator's judgment. Presence of any febrile illness (including oral temperature (OT) ≥38.0˚C within 24 hours prior to immunization). Such subjects may be re-evaluated for enrolment after resolution of illness.<br>17) Cancer or treatment for cancer within 3 years of study vaccine administration. Persons with a history of cancer who are disease-free without treatment for 3 years or more are eligible. Persons with treated and uncomplicated basal cell carcinoma of the skin are eligible. Person with non-treated, non-disseminated local prostate cancer are eligible.<br>Identified as an Investigator or employee of the Investigator or clinical site with direct involvement in the proposed study, or identified as an immediate family member (i.e., parent, spouse, natural or adopted child) of the Investigator or employee with direct involvement in the proposed study.

Table 1: Study visit procedures

Day of visit	Screen Visit	Day 0	Day 1	Day 3	Day 8	Day 21	Monthly[11] calls	Day 201[12]
Visit type	Enrol.	Vaccine/ Immuno	☎ Safety	Collect Safety	☎ Safety	Safety/ Immuno	☎ Safety	Final Safety/ Immuno
Visit allowed time window	Day -30 to Day -1		+1 day	-1/+2 days	-1/+2 days	- 2/+3 days	±7 days	±14 days
Informed consent (ICF)	X							
Inclusion/Exclusion Criteria and Eligibility	X	X						
Eligibility assessment		X		X		X		
Demographics	X							
Complete Physical Examination History/symptom-directed Physical Examination[1]	X	X		X		X		X
Medical & medication history (within 3 months prior to immunization) & check for changes at each following visit or call	X	X[9]	X	X	X	X	X	X
Body measurements (Height, weight, BMI)[2]	X	X						
Vital Signs (BP, HR, RR, OT)[3]	X	X		X		X		X
Clinical Laboratory Samples (Chemistry, Haematology, and Urinalysis)	X			X				X
Serology(HIV, Hepatitis B & C)	X							
Pregnancy test[4]	X	X				X		
Randomization		X						
Serology for HI, MN titers[5]		X				X		X
Cellular Immune Response (for PBMC preparation)[6]		X				X		X
Vaccine administration		X						
Safety Observation (for at least 30-minutes post-vaccination)		X						
Memory Aid (MA or diary card) Instructions[7]		X				X		
Collection of Solicited and systemic signs and symptoms[8]		X	X		X			
Occurrence of AEs/SAEs, NOCDs/Meds		X[10]	X	X	X	X	X	X
Study conclusion								X

[1] Complete physical examination (PE) to be done at the Screening and history/symptom-directed PEs at Day 0, 3, 21, and 201 (PE will not be routinely performed, unless deemed to be necessary by the Investigator). If subject complains of arm and/or shoulder pain, a direct examination of the lymph node for swelling (neck and axilla) will be done.
[2] During Day 0 visit procedures, only weight will be measured while the height will be obtained from that measured at initial screening visit.
[3] Blood pressure (BP), heart rate (HR), respiratory rate (RR; not done at screening), and oral temperature (OT) will be measured at screening, prior and after immunization on Day 0, and at Day 3, 21, and 201.
[4] To be performed for all female subjects; at screening it will be in sera and at Days 0 (before immunization) and Day 21 in urine.
[5] Blood sampling will be performed prior to immunization on Day 0.
[6] Blood sampling for CMI response analysis (PBMC) in 15 subjects per treatment group, for a total of 60 subjects.
[7] This includes training on measurement device and use of the thermometer at Day 0, instructions for OT and solicited and systemic signs and symptoms collection from Day 0 (evening) up to Day 7 (evening). Emergency information and instructions will also be given. A MA will be given on Day 0 and Day 21 (to cover Day 0 to Day 21 and Day 21 to Day 201 periods, respectively) and could be requested to bring their MA to clinical site visits and have it available during each phone contact, as reference.
[8] During the phone contact, subjects will be reminded to record any information on their MA (including their next visit and/or phone contact).
[9] Record any changes in medical history and medication intakes since screening and confirm subjects' eligibility.
[10] Record the occurrence of any AEs/SAEs prior and post-immunization (during the 30-minute observation period).
[11] Subjects should be reached once a month with no more than 45 days between phone contacts (use Day 21 date as starting reference).
[12] Unscheduled visits could occur during the study (based on Investigator's request or for repeat measurements purpose).

LIST OF ABBREVIATIONS

AE	Adverse Event
ALT	Alaninetransferase
ANOVA	Analysis of variance
AST/SGOT	Aspartatetransferas
BMI	Body Mass Index
BP	Blood Pressure
CBER	Center for Biologics Evaluation and Research
CCD	Cross-reactive carbohydrate determinant
CI	Confidence Interval
CMI	Cell-mediated immune response
CPMP	Committee for Proprietary Medicinal Products
CRF	Case Report Form
CRO	Clinical Research Organization
DNA	Deoxyribonucleic acid
DSMB	Data Safety Monitoring Board
FAS	Full Analysis Set
FDA	Food and Drug Administration
GCP	Good Clinical Practice
GGT	Gamma glutamyltransferase
GLP	Good Laboratory Practice
GMFR	Geometric Mean Fold Rise
GMP	Good Manufacturing Practice
GMT	Geometric Mean Titre
H/HA	Hemagglutinin
HI	Hemagglutination Inhibition
HIV	Human immunodeficiency virus
HR	Heart Rate
IB	Investigational brochure
ICF	Informed Consent Form
ICH	International Conference on Harmonization
IgE	Immunoglobulin E
IND	Investigational New Drug
IM	Intramuscular
IRB	Investigational Review Board
LOQ	Limit of Quantification
MA	Memory Aid

MCH	Mean cell haemoglobin
MCHC	Mean cell haemoglobin concentration
MCV	Mean cell volume
MedDRA	Medical Dictionary for Drug Regulatory Affairs
MN	Microneutralization
MRA	Miami Research Associates
N/NA	Neuraminidase
NOCD	New onset of a chronic disease
OT	Oral temperature
OTC	Over-the-Counter
PBMCs	Peripheral Blood Mononuclear Cells
PBS	Phosphate buffered saline
PI	Principal Investigator
PP	Per Protocol
QC	Quality control
QA	Quality assurance
RNA	Ribonucleic acid
RR	Respiratory Rate
SAE	Serious Adverse Event
SAP	Statistical Analysis Plan
SAS®	Statistical Analysis System®
URTI	Upper Respiratory Tract Infection
VLP	Virus-like particle
WBC	White blood cells
WHO	World Health Organization

1. INTRODUCTION

1.1. Background

Seasonal influenza is a potentially serious infection associated with a wide range of clinical outcomes across the entire human lifespan. The influenza virus genome is an 8-segment single-stranded RNA with high potential for in situ recombination and mutations for host adaptation. Two segments code for the hemagglutinin (H) and neuraminidase (N) antigens that play an essential role for virus infectivity. At present, 17 H and 9 N subtypes are known in bird populations and some subtypes routinely circulate in humans such as the H1N1, H3N2 and B influenza viruses. When a new subtype acquires the capacity for human-to-human transmission, this can give rise to a major pandemic like the one caused by an H1N1 strain in 1918 referred to as the Spanish flu. Antibodies binding to the HA molecule can prevent or modify infection and are the basis on which influenza vaccines are formulated every year.

The main strategy for prevention and control of seasonal and pandemic influenza is still vaccination. Since 1960, annual influenza vaccination has been recommended in the USA for individuals with chronic disease, those aged 65 years or older and (more recently) pregnant women. In 2012, the Advisory Committee on Immunization Practices issued the first recommendation of national universal seasonal influenza vaccination[1].

Despite continuing advances in understanding of the immune response to natural infection and immunization, the disease remains a major cause of morbidity and mortality worldwide. In the USA alone, influenza is responsible for approximately 36,000 deaths per year and the World Health Organization estimates that influenza causes 3-5 million cases of severe illness and 250,000- 500,000 deaths per year[2]. Influenza is also responsible for a massive economic burden, with a total cost for each winter influenza season evaluated at over 87 billion dollars[3] in the United States alone.

Licensure of influenza vaccines is based either on efficacy studies or on surrogate correlates of protection. The latter are primarily based on their ability to induce hemagglutination-inhibition (HI) titers. The current criteria used by the FDA for accelerated approval (using surrogate endpoints) of an influenza vaccine are the following:

For adults < 65 years of age and for the pediatric population:

- The lower bound of the two-sided 95% CI for the percent of subjects achieving seroconversion for HI antibody should meet or exceed 40%.
- The lower bound of the two-sided 95% CI for the percent of subjects achieving an HI antibody titer > 1:40 should meet or exceed 70%.

For adults ≥ 65 years of age:

- The lower bound of the two-sided 95% CI for the percent of subjects achieving seroconversion for HI antibody should meet or exceed 30%.
- The lower bound of the two-sided 95% CI for the percent of subjects achieving an HI antibody titer >1:40 should meet or exceed 60%.

Despite the existence of these surrogate correlates of protection, recent reviews of vaccine efficacy studies suggest that the overall efficacy of licensed trivalent vaccines is highly variable and depends, among other factors, on the 'match' between circulating strains and the strains used for vaccine production. One such analysis produced efficacy estimates from 43-89% in well-matched seasons and from 38-85% in poorly matched seasons (<25% of isolates matched the vaccine strains)[4], suggesting that there is still great room for improvement.

For the 2012-2013 season, vaccine efficacy numbers are particularly discouraging. When broken down by age group, the vaccine overall efficacy against influenza A and B viruses ranged from 27% in people 65 and older to 64% in children (aged 6 months to 17 years old). When specific strains and age groups were examined (eg: H3N2 in the elderly), efficacy estimates could be as low as 9% (95% CI: -84% to 55%)[5].

Medicago's plant-derived VLP Quadrivalent Influenza vaccines may be able to address several of the limitations of currently licensed vaccines. First, the HA proteins in each monovalent VLP is based on the genetic sequence of circulating human influenza viruses selected by the WHO. In contrast, influenza strains grown in embryonic eggs or tissue culture tend to mutate for optimal

growth in these substrates. Some of these mutations can occur in the critical HA protein and may even involve changes in cellular receptor tropism (eg: adaptation to avian 2-3α sialic acid receptor rather than the human 2-6α sialic acid receptors). The extent of mutations in the HA protein is strain dependent and can vary widely from year to year. For example, the H1N1 virus strain currently used by egg-based vaccine manufacturers (reassortant X-179A H1N1 strain) contains 4 amino acid mutations in the globular domain of the HA protein. Similarly, when grown in eggs, most influenza B strains contain at least one amino acid mutation that affects HA glycosylation compared to human isolates[6]. In some cases, these mutations change the affinity of antibodies directed against human strains. Although such cross-species selection pressure can theoretically be avoided with the use of mammalian cell culture instead of embryonic eggs (eg: Novartis' newly-licensed TIV - Flucelvax™) unpredictable mutations can arise with living virus in any artificial culture system. Furthermore, any living virus needs to be inactivated by chemicals and then split by a detergent before injection into humans, processes that can also influence antigenicity.

There are no mutations in the HA molecule produced with the plant-based manufacturing system since this platform does not rely on the growth of living viruses. Medicago's technology is based on the production of recombinant HA proteins that self-assemble into Virus-Like Particles (VLPs). These nanoparticules mimic the shape of the influenza virus and are efficiently recognized by the immune system that has evolved to recognize pathogens of such dimension.

Medicago has accumulated considerable pre-clinical and clinical data showing the extent of the immune response induced by such VLP vaccines. First, preclinical studies show that 1-2 doses of H5 or H7 VLP vaccine can protect animals from disease and death despite low to undetectable HI antibodies at some doses. Challenge studies in both ferret and mouse models further demonstrate that these VLP vaccines can even provide some degree of heterosubtypic protection. For example, mice immunized with a single dose of an H5 VLP vaccine are largely protected from lethal H2N2 challenge. These results strongly suggest that protection against influenza can be mediated by immune effectors other than HI antibodies and that plant-derived VLP vaccine have the potential to induce cross-reactive protection.

Medicago's clinical trial program has revealed that plant-made H5 and H1 VLP vaccines also induce long-lasting and cross-reactive T cell responses which could be pivotal for protection against both matched and diverse influenza strains. After almost half a century of focusing on humoral antibody responses to influenza, there is increasing interest in revisiting the role of T cell immunity after both influenza infection and vaccination[7-8]. In particular, T cell memory may be important for understanding natural infection and vaccine-induced immunity in the elderly who often derive significant benefit from vaccination despite little evidence of a humoral response[9]. Recent evidence suggests that cross-reactive T cells elicited by infection and vaccination contribute to cross-strain protection in both animal models and humans. These observations have led to the introduction of adjuvants that can boost the humoral and cell-mediated responses induced by traditional egg-derived split vaccines[10].

Our results to date suggest that plant-derived VLP vaccines induce strong T cell responses even in the absence of adjuvants. Although the primary end-points of the proposed phase 1-2 clinical trial of Medicago's seasonal VLP Quadrivalent vaccine will be safety and the induction of HI antibodies, secondary and exploratory end-points will include more detailed antibody responses (eg: microneutralization (MN) and antibodies against conserved epitopes of the stem region) as

well as T cell responses. The latter will be measured using PBMCs collected from 15 subjects per group at days 0, 21 and 201. T cell cross-reactivity will be evaluated by stimulating the PBMCs with various VLPs and peptide pools.

The data collected in these studies will help define the quality of the immune response induced by the plant-made vaccine, establish potential competitive advantages, support the design of a subsequent clinical trial.

1.2. Background of the Investigational Product

Please refer to Section 2.2 and 3.0 of the Investigator Brochure.

1.3. Clinical Studies

In 2013, Medicago completed a Phase 1-2 study in the US (IND 15716) for its quadrivalent VLP influenza vaccine candidate. The current quadrivalent VLP vaccine is produced by a similar process, thus the results for the Phase 1-2 study provide supportive safety and immunogenicity data for the quadrivalent formulation. In addition, potential reactions to plant glycans were investigated in this trial and the results are presented in 1.3.3.

1.3.1 SAFETY

Overall results from the phase 1-2 clinical study showed that the VLP Quadrivalent Influenza vaccine was well tolerated with an incidence of solicited signs and symptoms and an adverse event (AE) profile that did not represent a notably greater safety risk than that of the placebo (see Table 2). A few cases of fever over 38ºC have been reported and local and systemic reactions were mostly mild and of short duration. Neither SAEs nor allergic reactions were reported up to Day 21. No safety issues were noted with respect to physiological measurements. The 6 month safety follow up data will be available in July 2014.

Table 2: Incidence of systemic and local reactions within 7 days after the vaccination[1]

GROUP	PLACEBO	3 µg/strain VLP QUADRIVALENT VACCINE	9 µg/strain VLP QUADRIVALENT VACCINE	15µg/strain VLP QUADRIVALENT VACCINE
	N=30	N=30	N=30	N=30
SUBJECTS WITH AT LEAST ONE SOLICITED LOCAL OR SYSTEMIC REACTIONS				
	11(36.7)	14(46.7)	14(46.7)	18(60.0)
SYSTEMIC				
Fever	1(3.3)	0	2(6.7)	1(3.3)
Fatigue	5(16.7)	1(3.3)	4(13.3)	5(16.7)
Headaches	7(23.3)	4(13.3)	4(13.3)	7(23.3)
Muscle aches	3(10.0)	0	4(13.3)	5(16.7)
Feelings of general discomfort or uneasiness	4(13.3)	1(3.3)	3(10.0)	4(13.3)
Joint aches	2(6.7)	0	0	4(13.3)
Chills	1(3.3)	1(3.3)	2(6.7)	2(6.7)

Swelling in the Axilla	0	0	1(3.3)	0
Swelling in the Neck	1(3.3)	0	0	1(3.3)
Swelling in the Groin	0	0	0	0
LOCAL				
Redness	0	0	0	0
Swelling	2(6.7)	0	2(6.7)	2(6.7)
Pain	4(13.3)	11(36.7)	11(36.7)	15(50.0)

[1] Data are number of subjects reporting adverse reactions within 7 days of vaccination. Percentages are based on number of patients in the study population.

1.3.2 ANTIBODY RESPONSE

The antibody response against the four strains included in the vaccines is shown below. Table 3 shows the HI antibody results and Table 4 shows the serum neutralizing antibody results.

Briefly, 21 days after vaccination all tested dosages induced a detectable antibody response against the 4 strains that is statistically different from the placebo group. CHMP criteria were met at all tested dose for the A influenza strains and with dosages of 9 and 15 micrograms. There was no statistical difference in GMT, seroconversion and seroprotection rates between the 3, 9 or 15 microgram doses for the H1N1 and H3N2 influenza strains. However, for the B/Brisbane strain (Victoria lineage), the 15 microgram dose induced a significant higher antibody response compared to the 3 microgram dose (p=0.0188) and a higher seroconversion rate (p=0.0083). For the B\Wisconsin strain, the 15 microgram dose induced a higher antibody level and seroprotection rates than the 3 microgram dose (p values of 0.0394 and 0.0257). For the B\Wisconsin strain, the seroprotection rate of the 9 microgram dose was lower than the 15 microgram dose (p value of 0.0271). Based on these results, the next trial will evaluate dosages of 15, 30 and 60 micrograms of the VLP Quadrivalent vaccine. The vaccine will be evaluated in groups of 75 subjects per arm in order to evaluate immunogenicity according to CBER's criteria. At time of issue of this IB, cross-reactive HI antibody results were not available.

Table 3: Serum Hemagglutination-Inhibition Antibody Titers (Full set analysis)

**Strain**	A/California/07/2009, A/Victoria/361/2011, B/Wisconsin/1/2010 (Yamagata lineage), B/Brisbane/60/2008 (Victoria lineage)			
**Dose**	3µg/Strain (Total HA: 12µg)	9µg/Strain (Total HA: 36µg)	15µg/Strain (Total HA: 60µg)	Placebo
**N**	29	30	27	30
**Age (years)**	18-49 years	18-49 years	18-49 years	18-49 years
**Reference**	Results from Clinical Trial : CP-Q12VLP-004			
**H1N1**	**A/California/07/2009 WT**			
**GMT D21**	72.7 (44.4-119.0)	68.5 (40.7-115.0)	102.0 (57.7-180.4)	14.8 (9.5-23.0)
**Seroprotection D0**	34.5% (17.9-54.3)	30.0% (14.7-49.4)	48.1% (28.7-68.1)	26.7% (12.3-45.9)
**Seroprotection D21**	79.3% (60.3-92.0)	73.3% (54.1-87.7)	81.5% (61.9-93.7)	33.3% (17.3-52.8)
**Seroconversion D21**	41.4% (23.5-61.1)	50.0% (31.3-68.7)	40.7% (22.4-61.2)	0.0% (0.0-11.6)
**GMI (D21/D0)**	3.7 (2.6-5.2)	4.3 (3.1-6.0)	4.1 (2.9-5.8)	1.0 (0.7-1.4)
**H3N2**	**A/Texas/50/2012 (NIBSC #13/162)**			
**GMT D21**	91.8 (54.5-154.7)	129.9 (82.8-203.9)	128.7 (70.2-236.0)	14.5 (9.5-22.0)
**Seroprotection D0**	27.6% (12.7-47.2)	33.3% (17.3-52.8)	48.1% (28.7-68.1)	23.3% (9.9-42.3)
**Seroprotection D21**	79.3% (60.3-92.0)	90.0% (73.5-97.9)	81.5% (61.9-93.7)	23.3% (9.9-42.3)
**Seroconversion D21**	48.3% (29.4-67.5)	60.0% (40.6-77.3)	44.4% (25.5-64.7)	0.0% (0.0-11.6)
**GMI (D21/D0)**	4.8 (3.2-7.3)	7.4 (4.9-11.0)	5.8 (3.8-8.8)	0.9 (0.6-1.4)
**B (Victoria lineage)**	**B/Brisbane/60/2008 (NYMC BX-35) (NIBSC#10/106)**			
**GMT D21**	38.6 (25.4-58.6)	49.8 (32.6-76.1)	82.1 (50.4-133.6)	19.7 (12.1-32.1)
**Seroprotection D0**	34.5% (17.9-54.3)	16.7% (5.6-34.7)	40.7% (22.4-61.2)	30.0% (14.7-49.4)
**Seroprotection D21**	65.5% (45.7-82.1)	70.0% (50.6-85.3)	85.2% (66.3-95.8)	30.0% (14.7-49.4)
**Seroconversion D21**	13.8% (3.9-31.7)	43.3% (25.5-62.6)	48.1% (28.7-68.1)	0.0% (0.0-11.6)
**GMI (D21/D0)**	2.0 (1.5-2.7)	3.5 (2.6-4.7)	4.0 (3.0-5.4)	1.1 (0.8-1.5)
**B (Yamagata lineage)**	**B/Wisconsin/1/2010 (NIBSC #12/110)**			
**GMT D21**	64.5 (36.8-113.0)	74.5 (48.4-114.7)	136.3 (86.8-214.2)	22.4 (13.9-36.0)
**Seroprotection D0**	48.3% (29.5-67.5)	20.0% (7.7-38.6)	48.1% (28.7-68.1)	36.7% (19.9-56.1)
**Seroprotection D21**	72.4% (52.8-87.3)	73.3% (54.1-87.7)	96.3% (81.0-99.9)	43.3% (25.5-62.6)
**Seroconversion D21**	24.1% (10.3-43.5)	53.3% (34.3-71.7)	51.9% (31.9-71.3)	10.0% (2.1-26.5)
**GMI (D21/D0)**	2.6 (1.9-3.7)	5.2 (3.7-7.3)	5.5 (3.9-7.9)	1.2 (0.9-1.7)

The results shown in Table 4 confirm the trends seen with HI antibody results. In general seroconversion rates with the neutralizing antibody test are slightly higher for A influenza strains and comparable for B influenza strains. For this assay, one cross-reactivity result is available. Interestingly the VLP Quadrivalent vaccine induced 33.3% seroconversion rate against the newly selected B-Yamagata strain (B\Massachussets/2/2012) for the 2014-2015. These results suggest some level of cross-protection against heterosubtypic influenza strains.

Table 4: Serum Neutralizing Antibody Titers (non-audited data)

**Strain**	A/California/07/2009, A/Victoria/361/2011, B/Wisconsin/1/2010 (Yamagata lineage),B/Brisbane/60/2008 (Victoria lineage)			
**Dose**	3µg/Strain (Total HA: 12µg)	9µg/Strain (Total HA: 36µg)	15µg/Strain (Total HA: 60µg)	Placebo
**N**	29	30	27	30
**Age (years)**	18-49 years	18-49 years	18-49 years	18-49 years
**Reference**	Results from Clinical Trial : CP-Q12VLP-004			
**MN titers H1N1**	**A/California/07/2009**			
**GMT D0**	23.9 (13.2-43.2)	23.8 (14.4-39.2)	31.7 (15.9-63.1)	16.2 (9.9-26.7)
**GMT D21**	93.4 (53.7-162.5)	103.1 (56.4-188.6)	153.9 (86.4-274.1)	16.2 (9.9-26.5)
**Seroconversion D21**	44.8% (26.5-64.3)	46.7% (28.3-65.7)	55.6% (35.3-74.5)	0.0% (0.0-11.6)
**GMI (D21/D0)**	3.9 (2.3-6.6)	4.3 (2.7-6.9)	4.9 (2.8-8.3)	1.0 (0.9-1.1)
**MN titers H3N2**	**A/Texas/50/212**			
**GMT D0**	73.6 (42.4-127.7)	66.5 (39.6-111.6)	103.4 (59.1-181.0)	59.2 (36.4-96.5)
**GMT D21**	436.4 (286.3-665.7)	544.4 (370.8-799.4)	570.2 (395.9-821.1)	62.0 (37.9-101.5)
**Seroconversion D21**	48.3% (29.5-67.5)	70.0% (50.6-85.3)	63.0% (42.4-80.6)	0.0% (0.0-11.6)
**GMI (D21/D0)**	5.9 (3.4-10.3)	8.2 (5.1-13.1)	5.5 (3.5-8.8)	1.0 (1.0-1.2)
**MN titers B (Victoria lineage)**	**B/Brisbane/60/2008**			
**GMT D0**	12.5 (8.8-17.8)	10.8 (7.3-16.0)	15.3 (10.2-22.9)	13 (8.2-20.7)
**GMT D21**	24.5 (16.1-37.2)	31.0 (20.4-47.0)	57.3 (35.2-93.2)	13.0 (8.1-20.9)
**Seroconversion D21**	20.7% (8.0-39.7)	26.7% (12.3-45.9)	40.7% (22.4-61.2)	0.0% (0.0-11.6)
**GMI (D21/D0)**	2.0 (1.4-2.7)	2.9 (2.1-3.9)	3.8 (2.3-6.0)	1.0 (0.9-1.1)
**MN titers B (Yamagata lineage)**	**B/Wisconsin/1/2010**			
**GMT D0**	27.3 (17.0-43.9)	17.6 (10.9-28.4)	29.4 (18.6-46.4)	28.3 (19.7-40.7)
**GMT D21**	69.3 (40.3-119.2)	71.3 (49.6-102.3)	107.5 (65.5-176.4)	23.0 (14.9-35.4)
**Seroconversion D21**	34.5% (17.9-54.3)	53.3% (34.3-71.7)	44.4% (25.5-64.7)	0.0% (0.0-11.6)
**GMI (D21/D0)**	2.5 (1.8-3.6)	4.0 (2.9-5.7)	3.7 (2.3-5.9)	0.8 (0.6-1.0)

MN titers B (newly recommended Yamagata lineage for 2014-2015 season)	B/Massachusetts/2/2012			
**GMT D0**	14.6 (9.8-21.8)	9.8 (6.3-15.1)	18.5 (11.2-30.7)	13.2 (8.9-19.4)
**GMT D21**	27.9 (16.6-47.0)	30.6 (19.7-47.4)	62.6 (38.7-101.5)	12.7 (8.7-18.6)
**Seroconversion D21**	13.8% (3.9-31.7)	26.7% (12.3-45.9)	33.3% (16.5-54.0)	0.0% (0.0-11.6)
**GMI (D21/D0)**	1.9 (1.4-2.5)	3.1 (2.3-4.3)	3.4 (2.1-5.4)	1.0 (0.9-1.0)

1.3.3 RESULTS FOR PLANT GLYCANS

The serum at Day 0, 21 were analyzed for the presence of IgE to plant-specific β(1-2) xylose and α(1-3)fucose carbohydrate residues according to the bromelain test (ImmunoCap, Thermo Scientific). This test is used to identify subjects with potentially clinically-relevant allergies to plant glycans. The level of IgEs to bromelain (glycoprotein extracted from pineapple) are classified into five grades (0 to 4) corresponding to symptom levels. As shown in Table 5, vaccination with the quadrivalent VLP vaccine did not increase the IgE level of any subject 21 days post-dose, confirming the general safety of plant glycans found in the plant-made vaccine. By comparison, one subject in the placebo group showed an increase in anti-bromelain glycan. This is most probably due to natural exposure to allergens.

Table 5: IgE responses to plant glycans according to the bromelain assay

CLINICAL TRIAL	GROUP	NUMBER OF SUBJECTS WITH IGES ≥GRADE 1 TO BROMELAIN AT SCREENING	NUMBER OF SUBJECTS THAT SHOWED AN IGE INCREASE 1 MONTH AFTER VACCINATION
PHASE 1-2 WITH VLP QUADRIVALENT VACCINE (ONE DOSE)	3 µg/strain	0/29	0/29
	9 µg/strain	0/30	0/30
	15 µg/strain	1/27	0/27
	Placebo	0/30	1/30

Medicago is not expecting that the dosages proposed in the phase 2A study will have any impact regarding development of allergy symptoms and IgEs levels but this will of course, be part of the investigation for the VLP quadrivalent product.

1.3.4 Results for CMI

In order to better characterize the CMI generated by the plant-derived quadrivalent VLP vaccine, the peripheral blood mononuclear cells (PBMC) were collected from 10 subjects randomly chosen in the different treatment groups. Unfortunately, some samples were not properly processed and

cannot be used in the analysis. The technical problem was fixed but due to these issues we had to restrict the analysis to 6 Placebo subjects and 9 subjects who received 15 µg/strain of the quadrivalent VLP.

Homotypic immunity

A first analysis revealed qualitative changes in the response between patients who received 15 µg/strain quadrivalent VLP vaccine and Placebo (Figure 1). $CD4^+$ cells from vaccinated patients stimulated *ex vivo* with each of the 4 VLP included in the quadrivalent VLP vaccine exhibited a greater diversity in the polyfunctional response with a higher proportion of $CD4^+$ positive for at least 2 out of the 4 functional markers, namely IFN-γ, IL-2, TNF-α and CD107a as compared to Placebo, 21 days after immunization (Figure 1).

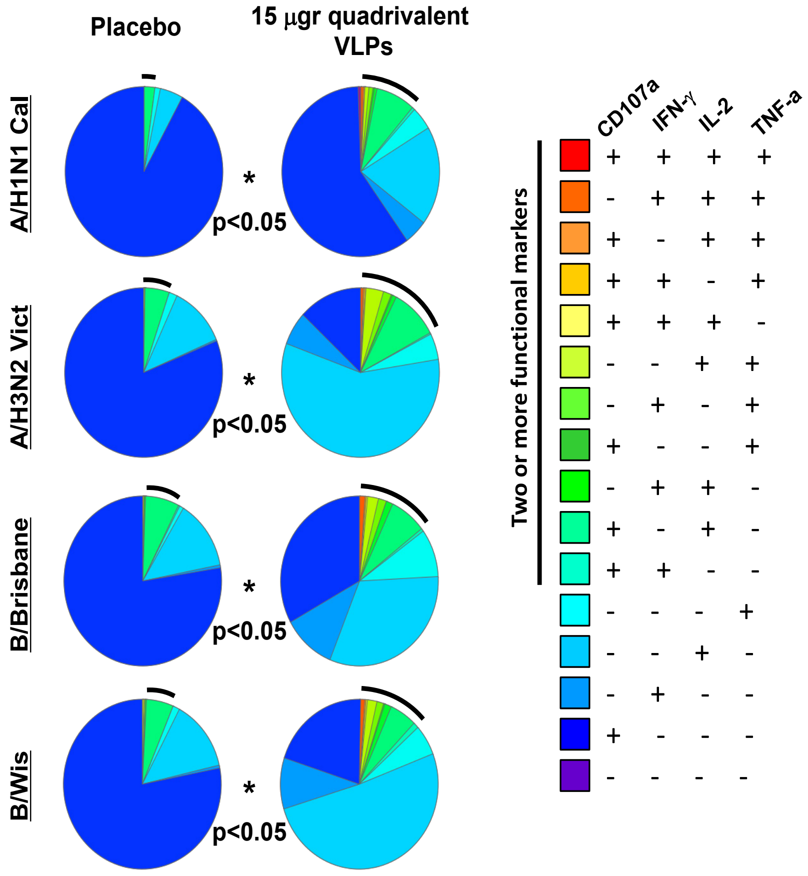

Figure 1: Relative distribution of the CD4 T cell functional response to ex vivo stimulation with each of the 4 VLPs included in the quadrivalent VLP vaccine. All the functional signature based on the expression of the 4 functional markers (CD107a, IFN-γ, IL-2 and TNF-α) were color-ranked from the more poly-functional (quadruple positive = red) to the quadruple negative (purple). The proportion of CD4 T cell expressing two or more functional markers are highlighted in black.

* $p<0.05$ indicates significant differences between the distribution of the response in Placebo and quadrivalent VLP vaccine groups (permutation analysis, SPICE v5.3 http://exon.niaid.nih.gov/spice/)

The presence of polyfunctional T cells has been associated with protection and better clinical outcome in several infections[11-13].

We also observed a recurrent increase of particular functional signatures in PBMCs from patients who received the quadrivalent VLP vaccine after *ex vivo* stimulation with each of the 4 VLPs included in the vaccine, 21 days after the immunization. All these functional signatures involved IFN-γ, alone or with other functional maker(s) (Figure 2).

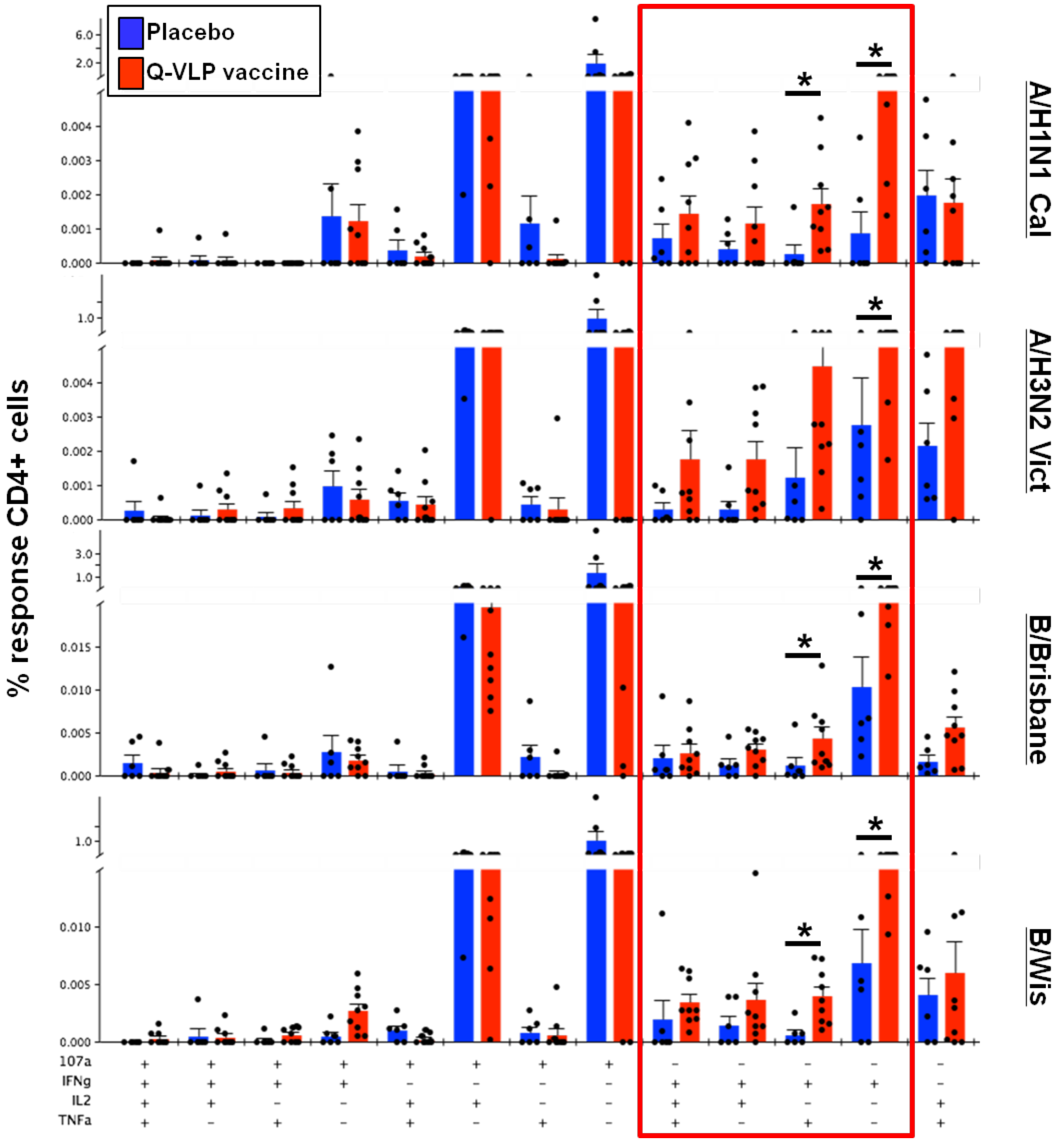

Figure 2: Details of the functional response of the CD4 T-cell based on the expression of the 4 markers (CD107a, IFN-γ, IL-2 and TNF-α) after *ex vivo* stimulation with each of the 4 VLPs included in the quadrivalent VLP vaccine 21 days after immunization. Histograms represent the percentage (mean ± SEM) of positive cells for each functional signature detailed on the bottom of the bar graphs. * Indicate significant ($p<0.05$) differences between Placebo and quadrivalent VLP vaccine (Wilcoxon signed rank test, SPICE v5.3 http://exon.niaid.nih.gov/spice/).

The presence of IFN-γ indicates an effector phenotype. Effector cells have been associated with the clearance of virus and the effector memory cells are seen as one of the most desirable population to be elicited by vaccination[11].

The heterogenicity of the T-cell memory response has been extensively investigated over the last 15 years however remaining largely unexplained. Basically, memory T-cell can be defined as Central Memory (CM), Transitional memory (TM) and Effector Memory (EM) based on the expression of CD45RA, CCR7 and CD27 at the surface of the T-cell (Table 6)[12].

Table 6: Sub-populations of memory T-cell based on the expression of cell-surface markers

	CD45RA	CCR7	CD27
CENTRAL MEMORY	-	+	+
TRANSITIONAL MEMORY	-	-	+
EFFECTOR MEMORY	-	-	-

We observed an increase of the same functional signatures in patients who received the quadrivalent VLP vaccine when analysis was restricted to the CD4+ TM cells ($CD45RA^-/CCR7^-/CD27^+$) and a significant difference was observed in CD4+ TM IFN-γ monovalent cells for each of the 4 VLPs included in the quadrivalent VLP vaccine after *ex vivo* stimulation of the PBMCs 21 days after immunization (Figure 3). The absence of statistically significant difference in some of the 3 other functional signatures involving IFN-γ highlighted in the Figure 3 is likely due to low statistical power resulting from the reduced number of samples.

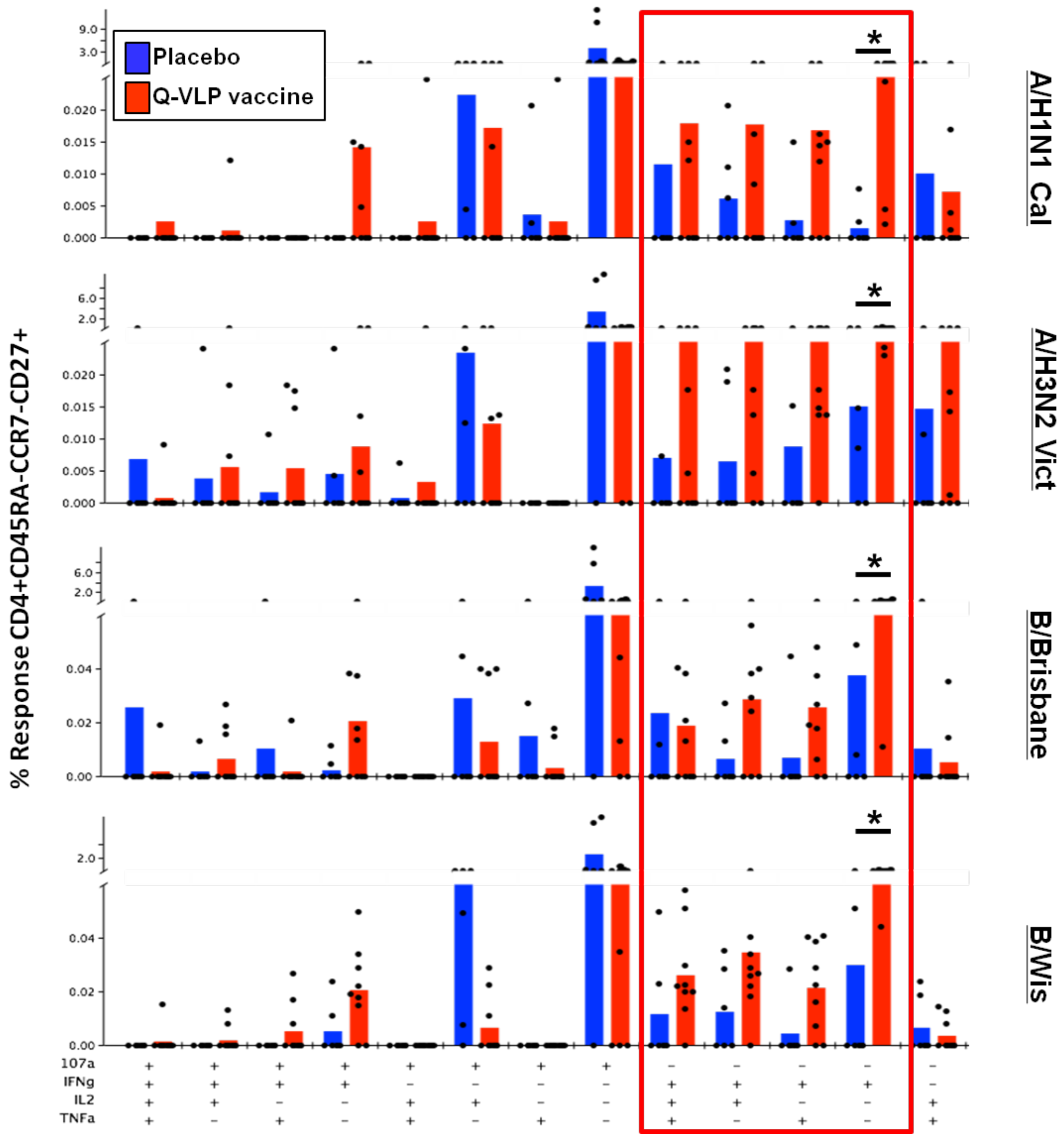

Figure 3: Details of the functional response of the TM CD4 T-cells based on the expression of the 4 markers (CD107a, IFN-γ, IL-2 and TNF-α) after *ex vivo* stimulation with each of the 4 VLPs included in the Q-VLP vaccine 21 days after immunization.

Histograms represent the percentage (mean) of positive cells for each functional signature detailed below the bar graphs. * Indicate significant ($p<0.05$) difference between Placebo and Q-VLP vaccine (Wilcoxon signed rank test, SPICE v5.3 http://exon.niaid.nih.gov/spice/).

Heterotypic immunity

The PBMCs were also stimulated *ex vivo* with a peptide pool consisting in 15-mer peptides overlapping by 11 amino acids and covering the entire sequence of B/Massachusetts HA protein to test for potential heterotypic CIM induced by the quadrivalent VLP vaccine.

Twenty-one days after immunization, patients who received the quadrivalent VLP vaccine had significantly more cross-reactive $CD4^+$ T-cells to B/Massachusetts HA peptide pool than Placebo (Figure 4). A similar increase, although not statistically significant, was observed in $CD8^+$ T-cells.

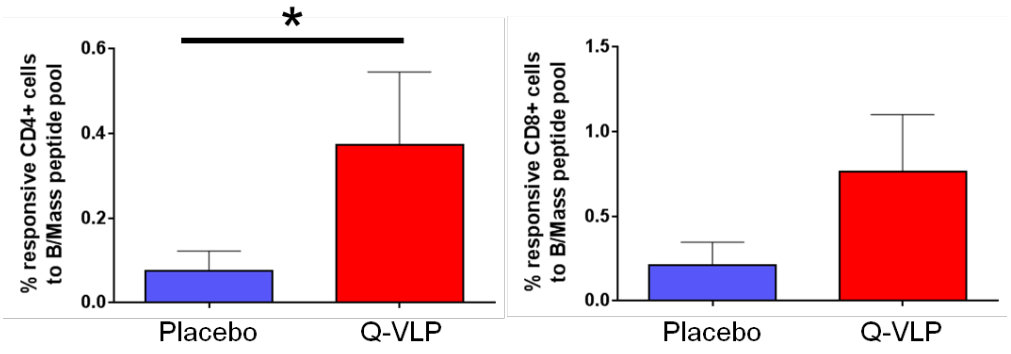

Figure 4: Percentage of total cross-reactive CD4 (left) and CD8 (right) T-cell to B/Massachusetts HA protein peptide pool.
Histograms represent the percentage (mean ± SEM) of responsive $CD4^+$ or $CD8^+$ T-cells defined as a T-cell expressing at least one of the four functional markers after *ex vivo* stimulation with the peptide pool. * Indicate significant ($p<0.05$) difference between Placebo and quadrivalent VLP vaccine (Mann Whitney test, GraphPad Prism v6.03).

The detailed analysis of the response revealed that vaccination with the quadrivalent VLP vaccine elicited a significant increase of the highly poly-functional $CD107a^-$/IFN-γ^+/IL-2^+/TNF-α^+ CD4 T-cells (Figure 5A). We also observed an increase, although non statistically significant ($p=0.06$) of the proportion of the quadruple positive $CD107a^+$/IFN-γ^+/IL-2^+/TNF-α^+ CD4 T-cells in patients who received the quadrivalent VLP as compared to the Placebo, 21 days after immunization.

A similar pattern was observed in TM CD4 T-cells which reflect the establishment of a population of cross-reactive transitional memory T-cells in patients vaccinated with the plant-derived quadrivalent VLP (Figure 5B).

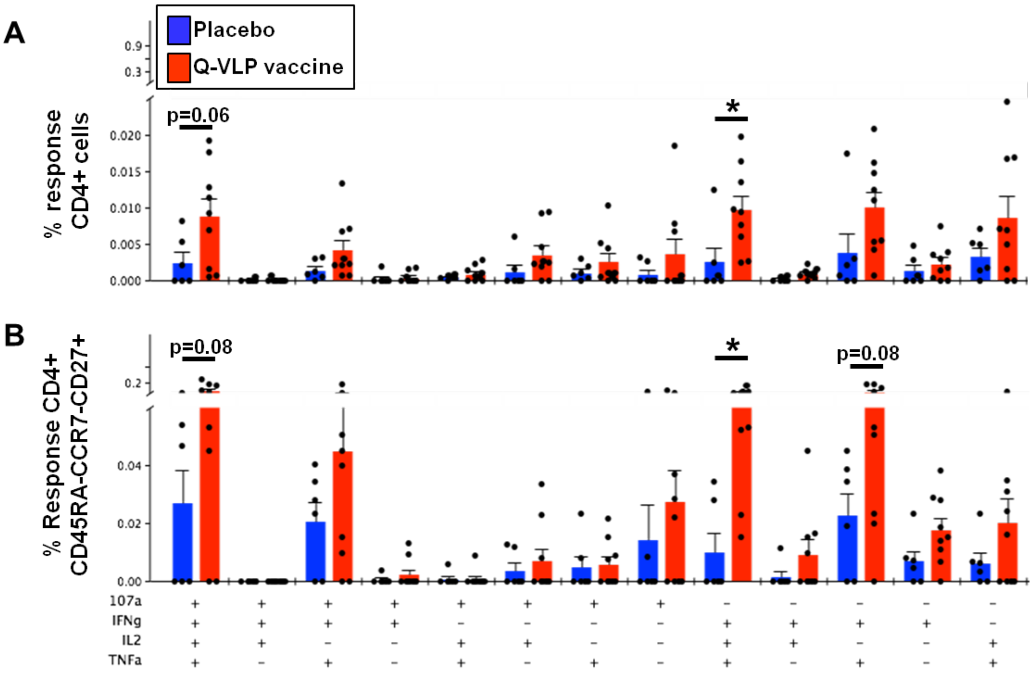

Figure 5: Details of the functional response of CD4 (A) and TM CD4 T-cells based on the expression of the 4 markers (CD107a, IFN-γ, IL-2 and TNF-α) after *ex vivo* stimulation with B/Massachusetts HA protein peptide pool 21 days after immunization.

Histograms represent the percentage (mean ± SEM) of positive cells for each functional signature detailed below the bar graphs. * Indicate significant ($p<0.05$) differences between Placebo and quadrivalent VLP vaccine (Wilcoxon signed rank test, SPICE v5.3 http://exon.niaid.nih.gov/spice/).

As stated above, the presence of poly-functional cells, and especially poly-functional effector memory cells, has been associated with better protection and clinical outcome in several infections.

1.4. Pre-Clinical Studies

Please refer to Section 4.0 of the Investigator Brochure.

2. TRIAL OBJECTIVES AND ENDPOINTS

2.1 Primary Objectives

The primary objective of this study is to assess the immunogenicity, safety, and tolerability of one dose of a Plant-Derived Seasonal VLP quadrivalent Influenza vaccine given at 3 dose levels: 15 µg, 30 µg, or 60 µg of each strain of the seasonal VLP vaccine compared to a placebo (saline).

One primary objective of this study is to measure the capacity of the VLP quadrivalent vaccine to induce specific antibodies against the H1N1, H3N2, and the B/Brisbane and B/Massachusetts virus strains contained in the vaccine. The European CPMP has established criteria on which

seasonal influenza vaccines obtain licensure in a number of countries. These criteria are based on the level of antibodies induced and the percentage of subjects achieving a target antibody titer measured by the HI test. The HI test will measure the level of antibodies for the A/California/7/2009/ H1N1, A/Victoria/361/2011 H3N2, B/Massachusetts/2/2012 (Yamagata lineage) and B/Brisbane/60/2008 (Victoria lineage) vaccine strains. As outlined in the next section, the Center for Biologics Evaluation and Research (CBER) also has criteria very similar to the CPMP, for evaluation of antibody levels, that are required for licensure in the US and these are outlined in the next section.

2.1.1 PRIMARY ENDPOINTS

As primary endpoint, immunogenicity will be evaluated primarily by measuring the serum HI response induced in subjects. Serum HI responses will be described in terms of Geometric mean titers (GMTs) of hemagglutination inhibition (HI) antibody on Day 0 and Day 21. Follow-up serology samples for GMTs will be collected at Day 201.

In accordance with the most recent serological criteria for influenza vaccine immunogenicity in FDA Guidelines[14], the three following criteria (seroconversion rate, seroprotection rate and seroconversion factor or GMFR (Geometric Mean Fold Rise)) are considered in the immunogenicity assessment of the seasonal quadrivalent influenza VLP vaccine:

- Seroconversion rate: the proportion of subjects in a given treatment group with either a ≥4-fold increase in reciprocal HI titers between Day 0 and Day 21; or a rise of undetectable HI titer (i.e. <10) pre-vaccination, (Day 0) to an HI titer of ≥40 at Day 21 post-vaccination. **The lower bound of the two-sided 95% confidence interval (CI) for the percent of subjects achieving seroconversion for HI antibody should meet or exceed 40%.**
- Seroprotection rate: the proportion of subjects in a given treatment group attaining a reciprocal HI titer of ≥40 at 21 days post-vaccination (the percentage of vaccine recipients with a serum HI titer of at least 1:40 following vaccination). **The lower bound of the two-sided 95% CI for the percent of subjects achieving an HI antibody titer >1:40 should meet or exceed 70%.**
- Seroconversion factor or GMFR is the geometric mean of the ratio of GMTs (Day 21/Day 0). Mean geometric increase >2.5.

As another primary endpoint, safety and tolerability will also be assessed, including solicited local events as follows: erythema (redness), swelling, and pain at the injection site. The following systemic symptoms will also be solicited: headache, fever, muscle aches, joint aches, fatigue, chills, malaise, swelling in the neck and axilla. The causal relationship of the solicited local and systemic reactions will be graded as: mild (1), moderate (2), severe (3) or potentially life threatening (4). The occurrence of any AEs/SAEs, history/symptom-directed physical examination findings, clinical laboratory results, and vital signs (including oral temperature [OT]) will be assessed. The signs and symptoms will be measured and reported by each study subject from the time of study vaccine administration up to seven days post-vaccination (from Day 0–Day 1 and from Day 2–Day 7).

- Percentage, intensity, and relationship of immediate complaints (30 minutes post-vaccination);
- Percentage, intensity, and relationship to vaccination of solicited local (erythema, swelling, and pain at the injection site) and systemic signs and symptoms (headache, muscle aches, joint aches, fatigue, chills, malaise, and swelling in the neck and axilla) for 7 days following the study vaccine;
- Percentage, intensity, and relationship of unsolicited local and systemic signs and symptoms for 21 days following study vaccine;
- Occurrences of all AEs/SAEs;
- Occurrences of new onset of a chronic disease (NOCD);
- The number and percentage of subjects with normal and abnormal urine, haematological, and biochemical values at Screening, Days 3 and 201.

2.2 Secondary Objectives

The secondary objectives of this study include:

- The assessment of the reactivity of antibodies induced by one dose of seasonal VLP quadrivalent influenza vaccine at the three dose levels 15 µg, 30 µg, and 60 µg of each strain compared to the placebo, as measured by HI antibody titers for the homologous influenza strains, described in terms of Geometric mean titers (GMTs) of hemagglutination inhibition (HI) antibody on Day 0 and Day 21. Follow-up serology samples for GMTs will be taken at Day 201. This will be assessed using measures of percentage of subjects with a detectable HI antibody response (≥lower limit of quantification; ≥LOQ) at Days 0, 21, and 201.
- The assessment of the reactivity of antibodies induced by one dose of VLP quadrivalent influenza vaccine at the three dose levels 15 µg, 30 µg, and 60 µg of each strain, compared to the placebo, as measured by microneutralisation (MN) antibody titers for the homologous influenza strains. This will be assessed using measures of GMFR, seroconversion rate, and of the percentage of subjects with a detectable MN antibody response (≥LOQ) at Days 0, 21, and 201, against the homologous strains.
- The assessment of the cross-reactivity of antibodies induced by one dose of VLP quadrivalent influenza vaccine the three dose levels 15 µg, 30 µg, and 60 µg of each strain, compared to the placebo, as measured by HI and MN antibody titers for heterologous influenza strains. This will be assessed using measures of GMFR, seroconversion rate, seroprotection rate (for HI only), and of percentage of subjects with a detectable HI or MN antibody response (≥LOQ) at Days 0, 21, and 201

2.2.1 SECONDARY ENDPOINTS AND MEASURES

For the HI test against the homologous influenza vaccine strains:

As mentioned in primary objectives, immunogenicity will be evaluated by measuring the serum antibody response induced in subjects, using the HI test against the vaccine strains. A secondary endpoint from this analysis will be the measurement of the percentage of

subjects with a detectable HI antibody response (≥LOQ) at Days 0, 21, and 201, against the homologous strains.

For the HI test against the heterologous influenza strains:

GMTs of HI antibody for heterologous influenza strains on Day 0 and Day 21. Follow-up serology samples for GMTs will be collected at Day 201.

GMTs will be analyzed as follows:

- Seroconversion factor or GMFR: is the geometric mean of the ratio of GMTs (Day 21/Day 0).
- Seroconversion rate: the proportion of subjects in a given treatment group with either a ≥ 4-fold increase in reciprocal HI titers between Day 0 and Day 21; or a rise of undetectable HI titer (i.e. <10) pre-vaccination, (Day 0) to an HI titer of ≥40 at Day 21 post-vaccination.
- Seroprotection rate: the proportion of subjects in a given treatment group attaining a reciprocal HI titer of ≥40 at 21 days post-vaccination (the percentage of vaccine recipients with a serum HI titer of at least 1:40 following vaccination).
- The percentage of subjects with a detectable HI antibody response (≥LOQ) at Days 0, 21, and 201.

For the MN test against homologous and heterologous influenza strains:

GMTs of MN antibody for homologous and heterologous H1N1, H3N2, and B (B/Bris and B/Mass) strains on Days 0 and 21. Follow-up serology samples for GMTs will be collected at Day 201. GMTs will be analyzed as follows:

- Seroconversion factor or GMFR: is the geometric mean of the ratio of GMTs (Day 21/Day 0).
- Seroconversion rate: the proportion of subjects in a given treatment group with either a ≥ 4-fold increase in reciprocal MN titers between Day 0 and Day 21; or a rise of undetectable MN titer (i.e. <10) pre-vaccination, (Day 0) to an MN titer of ≥40 at Day 21 post-vaccination.
- The percentage of subjects with a detectable MN antibody response (≥LOQ) at Days 0, 21, and 201.

2.3 Exploratory Objectives

The exploratory objectives of this study are as follows:

- To evaluate cellular responses induced by one dose of a Seasonal VLP quadrivalent Influenza Vaccine given at dose levels of 15 µg, 30 µg, and 60 µg of each strain compared to the placebo. T cell responses will be assessed at Days 0, 21 and 201 in a subset of subjects.
- Since the HA protein of the Plant-Derived VLP quadrivalent contains plant-specific glycans, there is at least a theoretically concern that these glycans might trigger an allergic response. In clinical studies with Medicago's H5 VLP, H1 VLP and VLP quadrivalent vaccines to date, no allergic reactions have occurred and there has been no long-term induction of IgE to plant-specific sugars as measured in the bromelain

assay. An exploratory objective of this VLP quadrivalent vaccine study will be to monitor for IgE antibodies directed against plant glycans induced by vaccination (timeframe: Days 0, 21, and 201).

2.3.1 EXPLORATORY ENDPOINTS AND MEASURES

Cell-mediated immune response (CMI):

PBMCs will be isolated to assess the CMI induced by the quadrivalent vaccine. The role of cellular immunity in influenza is currently the subject of considerable research. In particular, some cellular responses appear to be good correlates of protection in the elderly as well as conferring cross-reactivity against other viruses. A vaccine that can induce protection against drifted strains would clearly be a significant advantage for seasonal use when vaccines are typically produced months in advance of their use.

Activation of CD4+ and CD8+ T cells of the adaptive immunity is also important in protection against viral infection and is not assessed by the measurement of specific antibodies. Cellular immune response analysis with PBMC collected in our previous clinical study revealed that the H5 VLP vaccine induced long lasting (6 months) polyfunctional CD4+ cells. Polyfunctional memory T cells are widely considered to have better effector function than monofunctional[10]. Additional analysis revealed that these CD4+ T cells are also cross-reactive towards other influenza strains. During the phase 1 trial conducted with the non-adjuvanted H1 VLP made for the A/California/7/09 H1N1 strain (NCT01302990), it was demonstrated that the Plant-Derived H1 VLP induced a higher percentage of polyfunctional CD4+ T cells than the comparator vaccine (Fluzone® trivalent vaccine form Sanofi USA) and that only the Plant-Derived vaccine induced T cells cross-reactive for other influenza strains. These features are important differentiation factors for the Plant-Derived vaccines that are of high importance for seasonal vaccine and also in the context of a pandemic. The quality of the T cell response induced by the Plant-Derived vaccine is thought to play an important role in the protection against heterologous influenza strains when antibody levels are low. Medicago plans to continue investigating the nature of both humoral and cellular immune responses induced by the Plant-Derived VLP quadrivalent in the proposed clinical trial.

Blood samples for PBMCs isolation will be taken from 15 subjects enrolled per treatment group on Days 0, 21, and 201.

CMI assay could include: Flow cytometry panel to characterize subset cell populations, including CD3, CD4, CD8, CD45RA, CD27 and CD16/CD56 and viability markers (Aqua) as well as functional markers including IL-2, TNFα and IFNγ and cytokines in combination with the cell surface expression of CD107a (lysosomal-associated membrane protein-1, or LAMP-1), granzyme B and perforin (clone B-D48) as markers of T cell degranulation and cytolytic activity.

Antibody to plant-specific glycans:

The possible induction of antibodies to plant-specific sugars found on the VLP vaccine will be assessed using the ImmunoCAP technology that measures IgE specific to the cross-reactive carbohydrate determinant (CCD) MUXF3 present on bromelain (ImmunoCAP: Pharmacia&Upjohn, Uppsala, Sweden). Bromelain contains only the

MUXF3 carbohydrate epitope. This CCD is found on many plant allergens and can trigger histamine release by mast cells and basophils *in vitro*[15-17]. Results of this assay are reported in kUA/l with an interpretation of potential clinical impact. Grade 0 is considered to be ‘negative’, Grades 1 and 2 are ‘low-’ and ‘moderate positive’ respectively while Grades 3-6 define varying degrees of ‘high’ and ‘very high’ positive. Values higher than 2 have been associated with clinically-relevant allergy. Data will be analyzed at Days 0, 21, and 201.

3. INVESTIGATOR AND TRIAL CENTER

Table 7: Sites and Responsible Key Personnel

Sponsor	Clinical Sites	DSMB	Other key roles	
**Medicago R & D Inc.**<br>1020, route de l'Eglise,<br>bureau 600<br>Sainte-Foy, Quebec,<br>Canada, G1V 3V9<br>Office: 1.418.658.9393,<br>Fax: 1.418.658.6699<br><br>**Senior Director-Clinical Studies**<br>Sonia Trepanier<br>Tel.: 1.418.658.9393 ext. 137<br>Mobile: 1.418.655.7158<br>trepaniers@medicago.com<br><br>**Sponsor Contact Manager-Clinical studies**<br>Sebastien Soucy<br>Tel.: 1.418.658.9393 ext. 122<br>Mobile: 1.418.446.2032<br>soucys@medicago.com<br><br>**Sponsor Medical Officer**<br>Dr. Brian J. Ward, M.D.<br>Research Institute of the MUHC<br>Montreal General Hospital<br>1650 Cedar Avenue,<br>Room L10-509<br>Montreal, Quebec,<br>Canada, H3G 1A4<br>Tel.: 1.514.934.1934 ext. 42810<br>After hours:<br>Mobile: 1.514.952.6583<br>Pager: 1.514.921.6953<br>brian.ward@mcgill.ca	**Sites and PIs**<br>Miami Research Associates (MRA)<br>6141 Sunset Drive, Suite 301<br>South Miami, Florida,<br>USA, 33143<br>PI:<br>Eric Sheldon, MD<br>Tel.: 1.305.598.3125<br>Fax: 1.305.595.9239<br>esheldon@miamiresearch.com<br><br>David J. Seiden, MD<br>Broward Research Group (BRG).<br>7261 Sheridan Street, Suite 210<br>Hollywood, Florida, USA, 33020<br>Office: 1.954.374.1150<br>Fax: 1.954.322.1633<br>dseiden@browardresearch.com	**Member of DSMB**<br>Eric Sheldon, MD<br>MRA<br>esheldon@miamiresearch.com<br><br>Brian J. Ward, M.D.<br>Research Institute of the MUHC<br>brian.ward@mcgill.ca<br><br>David J. Seiden, MD<br>BRG<br>dseiden@browardresearch.com<br><br>Scott A. Halperin, M.D.<br>IWK Health Centre,<br>Dalhousie University,<br>5850/5980 University Ave<br>Halifax, Nova Scotia,<br>Canada, B3K 6R8<br>Tel.: 1.902.470.8141<br>Fax: 1.902.470.7232<br>scott.halperin@dal.ca	**Safety laboratory**<br>MRA Clinical Research Lab (for chemistry, haematology, and urinalysis testing)<br>6280 Sunset Drive, Suite 610, St. Miami, Florida,<br>USA, 33143<br>Tel.: 1.305.722.6921<br>Fax: 1.305.722.6946<br><br>Quest Diagnostics Clinical Laboratories (for serology)<br>10200 Commerce Parkway<br>Miramar, Florida, USA,<br>33025-3938<br>Tel.: 1.954.378.5000<br>Fax: 1.305.595.9239<br><br>**Serological Laboratory (HI titer analysis)**<br>Medicago R&D Inc.<br>1020, route de l'Eglise,<br>bureau 600<br>Sainte-Foy, Quebec,<br>Canada, G1V 3V9<br>Michele Dargis, Director, Analytical Development<br>Tel.: 1.418.658.9393ext. 150<br>Fax: 1.418.658.6699<br>dargism@medicago.com	**Data Management, Statistical Analysis, and Medical Report writing**<br>INC Research Early stage<br>720 King Street West, 7th Fl<br>Toronto, Ontario,<br>Canada, M5V 2T3<br>Laura Melo, B.Sc. (Hons), Project Data Manager<br>Tel.: 416.963.5602 ext.: 327<br>Fax 416.963.9732<br>laura.melo@incresearch.com<br><br>**Analytical Laboratory (PBMC analysis)**<br>ImmuneCarta Services<br>201 Avenue President-Kennedy,<br>Suite PK-3850<br>Montreal, Quebec,<br>Canada, H2X 3Y7<br>Jean-François Poulin, Ph.D.,<br>Principal Scientist<br>Tel.: 1.514.335.8350 #54945<br>Fax: 1.514.333.0542<br>jpoulin@immunecarta.com<br><br>**(anti-glycan antibodies)**<br>Laboratoires Bio-Medic<br>4535, Wilfrid-Hamel, #140,<br>Quebec, Canada, G1P 2J7<br>Melanie Lessard, TM, RT<br>Directrice, Operations et RH<br>Tél. : 1-866-624-3322 # 235<br>Fax: 418.780.3031<br>melanielessard@labbiomedic.com

4. ETHICS APPROVAL

4.1 Ethical Conduct of the Study

The Institutional Review Board (IRB) for this study will be Aspire IRB, 11491 Woodside Avenue, Santee, California, USA, 92071 (email@aspire-irb.com).

The study will be conducted in accordance with the ethical principles that have their origins in the most recent update of the Declaration of Helsinki, in the International Conference on Harmonization (ICH) Guideline E6, in the GCP rules, and in the applicable USA regulatory requirements.

5. INVESTIGATIONAL PLAN

5.1 Description of the Overall Trial Design and Plan

This will be a multiple centres (at least 2 clinical sites) Phase II, randomized, observer-blind, dose ranging, placebo-controlled clinical study in adult subjects. A total of three hundred (300) subjects will be randomized in four (4) groups of 75 subjects. This study will be a cohort staggering (slow enrolment) for 3 dose levels (15 µg, 30 µg, and 60 µg VLP per strain) and a placebo-controlled group (ratio 1:1:1:1).

The 300 subjects (male and female subjects, 18 to 49 years of age) will be divided in 3 different cohorts. A 7-day safety data after the immunization will be collected by the clinical staff and will be reviewed by the Data and Safety Monitoring Board (DSMB). The DSMB members will determine if the clinical sites are allowed to continue immunization of the next cohort.

The proposed study schedule is presented in the Figure 6.

Figure 6: Proposed study schedule with approximate enrolment dates.
(dates may be earlier)

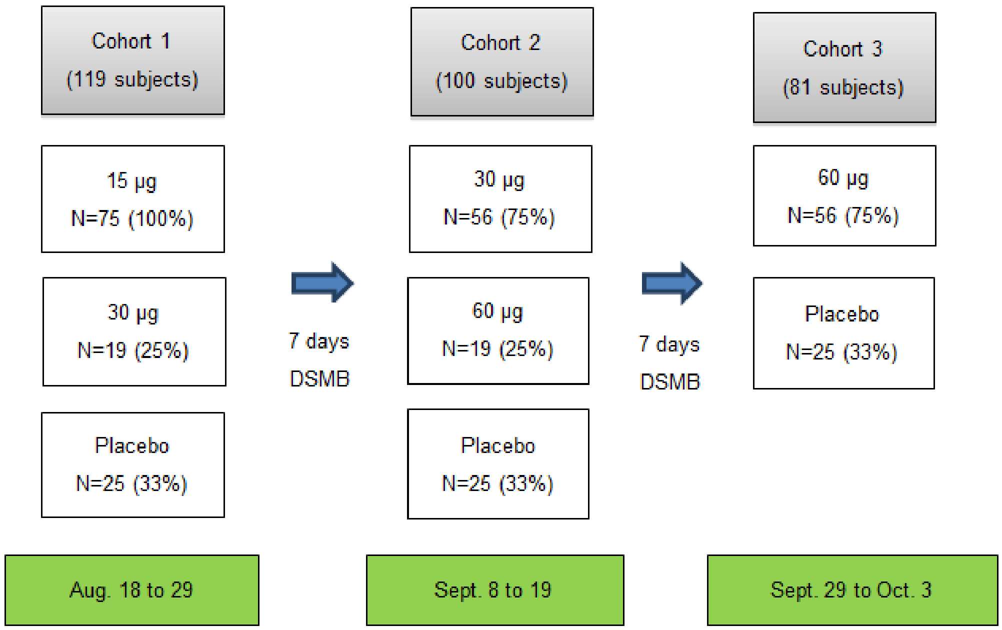

The subjects will be screened up to 30 days prior to study immunization and will demonstrate a satisfactory baseline medical assessment by general physical examination, biochemical, haematological, and serological analysis (stable health status with no exclusionary medical or psychiatric conditions). A serum pregnancy test will also be performed at screening for all females, regardless of their childbearing potential status. The screening data must be reviewed by the Investigator to determine subjects' eligibility prior to randomization.

On Day 0, just prior to immunization procedures, a urine pregnancy test (on all females) and vital signs measurements will be performed. In addition, blood samples for the assessment of the immune response (HI/MN) and for the isolation and analysis of PBMCs (only from 15 subjects per treatment group) will be collected. After confirmation that subjects is still eligible to continue in the study, the subject will be randomized and then immunized with the study vaccine.

The subjects will be administered either one of the 3 dose levels (15 µg, 30 µg, or 60 µg per strain) or the placebo. All subjects (regardless of treatment group) will receive the test article intramuscularly. Dose levels to be used in this study are presented below in Table 8.

Table 8: Dose levels to be used in this Phase IIa trial (Immunization on Day 0)

Test Material	***Treatment Group (Grp)***	***Dose Level***	***No. of Subjects***
VLP quadrivalent Influenza vaccine	Grp1	15 µg per strain	75
VLP quadrivalent Influenza vaccine	Grp 2	30 µg per strain	75
VLP quadrivalent Influenza vaccine	Grp3	60 µg per strain	75
Placebo	Grp 4	Not applicable; 100mM phosphate buffer + 150 mM NaCl + 0.01% Tween 80	75

Thereafter, subjects will be observed for at least 30 minutes after immunization for any signs and symptoms of local and systemic intolerance to the study vaccine. Any unusual signs or symptoms reported during the initial 30-minute of observation period will be promptly monitored closely. Vital signs will be measured within 10 minutes after the 30-minutes observation period and hourly thereafter, if deemed necessary by the Investigator. During the 30-minute observation period, subjects will be given a Memory Aid (MA; or diary card), a measuring device, and a digital thermometer and will be instructed on their use. Solicited local and systemic symptoms, as well as any unsolicited AEs and any concomitant medication intakes will be recorded by subjects in their MA for the 21 days following the administration of the study vaccine (i.e. from Day 0 post-vaccination through Day 21 visit). Subjects could be asked to bring their MA with them to subsequent visits and/or for phone contacts.

Subjects will be contacted by clinical staff on Day 1 and Day 8 (i.e. approximately 24 hours and 7 days post-vaccination, respectively) to collect data on any solicited or unsolicited events and to remind them to continue completing the MA up to the Day 21 visit.

Subjects will be required to return to the clinical site on Day 3 and on Day 21. Subjects will be asked about the occurrence of any AEs and any medication intakes; all such safety data will be recorded in the source documents. Blood chemistry and haematology testing, a urinalysis, and a urine pregnancy test (on all females) will be performed at Day 3 visit. Blood samples for the assessment of the immune response (HI/MN) and for the isolation and analysis of PBMCs (only from 15 subjects per treatment group) will also be collected at Day 21 visit. During the Day 21 visit, subjects will be provided a second MA and will be instructed to record any AEs and any concomitant medication intakes occurring from Day 21 to Day 201.

Following the Day 21 visit until the time of the final visit at Day 201, monthly phone calls to each subject will be performed in order to collect safety data such as new AEs/SAEs, NOCDs, new medications for AE/SAE and/or NOCD, subjects' hospitalizations, or unscheduled visit to the Investigator's sites.

Subjects will be required to return to the clinical site for the final visit on Day 201. Subjects will be asked about the occurrence of any AEs and any medication intakes; all such safety data will be recorded in the source data. Blood chemistry and haematology testing, a urinalysis, and blood samples for the assessment of the immune response (HI/MN) and for the isolation and analysis of PBMCs (only from 15 subjects per treatment group) will also be collected. Subjects could be requested to bring their MA with them upon their return to the clinic at the final visit (Day 201). At the time of this visit, all safety data will be recorded in source data.

The duration of the study is approximately 7-8 months (from screening procedures up to Day 201 visit).

5.1.1 INCLUSION CRITERIA

The subjects enrolled in this study must meet all of the following inclusion criteria:

1) Male and female adults, 18 to 49 years of age, inclusive.
2) Body mass index (BMI) of ≥18 and ≤32.
3) Give his/her consent to participate in this study (by signing the informed consent form [ICF]). In the opinion of the Investigator, competence and willingness to provide written, informed consent for participation after reading the ICF. The subject must have adequate opportunity to discuss the study with an Investigator or qualified designee.
4) Healthy as judged by the Investigator or designee and determined by complete general physical examination, vital signs, clinical laboratory tests, and medical history conducted no more than 30 days prior to study vaccine administration. Subjects with a pre-existing chronic disease will be allowed to participate if the disease is stable and, according to the Investigator's judgment, the condition is unlikely to confound the results of the study or pose additional risk to the subject by participating in the study. Stable disease is generally defined as no new onset of exacerbation of pre-existing chronic disease 6 months prior to immunization. Based on the Investigator's judgment, a subject with more recent stabilization of a disease could also be eligible.

5) Comprehension of the study requirements, expressed availability for the required study period, ability to attend scheduled visits, accessible by phone on a consistent basis.
6) If female, have a negative serum pregnancy test result at screening and negative urine pregnancy test on Day 0 prior to immunization.
7) Female of childbearing potential must use an effective method of contraception for 1 month prior to immunization and agrees to continue employing adequate birth control measures for at least 60 days post-immunization. Moreover, she must have no plan to become pregnant for at least 2 months post-immunization. Abstinent subjects should be asked what method(s) they would use, should their circumstances change, and subjects without a well-defined plan should be excluded.

   The following relationship or methods of contraception are considered to be effectives:
   - Hormonal contraceptives (e.g., injectable, topical [patch], estrogenic vaginal ring, etc.);
   - Intra-uterine device (IUD) with or without hormonal release;
   - Male partner using a condom plus spermicide or sterilized partner (at least 1 year prior to immunization);
   - Credible history of abstinence (self-reported);
   - Heterosexual abstinence at least 60 days post-immunization;
   - Female partner.
8) Non-childbearing females defines as:
   - Surgically-sterile (defined as bilateral tubal ligation or hysterectomy performed more than 1 month prior to immunization);
   - Post-menopausal (absence of menses for 24 consecutive months and age consistent with natural cessation of ovulation).

5.1.2 EXCLUSION CRITERIA

The subjects enrolled in this study must not meet any one of the following exclusion criterion:

1) According to Investigator's opinion, presence of significant acute or chronic, uncontrolled medical or neuropsychiatric illness. "Uncontrolled" is defined as:
   - Requiring a new medical or surgical treatment within one month prior to study vaccine administration;
   - Requiring a change in medication dosage in one month prior to study vaccine administration due to uncontrolled symptoms or drug toxicity (elective dosage adjustments in stable subjects are acceptable).
2) Any medical or neuropsychiatric condition or any history of excessive alcohol use or drug abuse which, in the Investigator's opinion, would render the subject unable to provide informed consent or unable to provide valid safety observations and reporting.

3) Any autoimmune disease or any confirmed or suspected immunosuppressive condition or immunodeficiency including history of human immunodeficiency virus (HIV) infection, Hepatitis B or C, or the presence of lymphoproliferative disease.
4) Administration of any vaccine (including any other influenza vaccine) within 30 days prior to study enrolment or planned administration within the period from the vaccination up to blood sampling at Day 21 or within 30 days prior to blood sampling at Day 201. Immunization on an emergency basis will be evaluated case-by-case by the Investigator.
5) Administration of any adjuvanted or investigational influenza vaccine within 1 year prior to the study enrolment or planned administration prior to the end of this study (Day 201). Administration of any 'standard', not adjuvanted influenza vaccine (e.g.: live attenuated TIV/QIV vaccine IN or split TIV/QIV vaccine by either intra-dermal or IM route) prior to the 30 day exclusion period mentioned above would be acceptable.
6) Use of any investigational or non-registered product within 30 days prior to study enrolment or planned use during the study period. Subjects may not participate in any other investigational or marketed drug study while participating in this study (approximately 8 months).
7) Treatment with systemic glucocorticoids at a dose exceeding 10 mg of prednisone per day, or equivalent for more than 7 consecutive days or for 10 or more days in total, within one month of study vaccine administration, any other cytotoxic or immunosuppressant drug, or any immunoglobulin preparation within 3 months of vaccination. Low doses of nasal or inhaled glucocorticoids are allowed.
8) Any significant disorder of coagulation or treatment with warfarin derivatives or heparin. Persons receiving prophylactic anti-platelet medications (e.g., low-dose aspirin [no more than 325 mg/day]), and without a clinically apparent bleeding tendency are eligible. Subjects treated with new generation drugs that will not increase risk of intramuscular bleeding (such as clopidogrel) are also eligible.
9) History of allergy to any of the constituents of the VLP quadrivalent (including H1N1, H3N2, B/Bris, and B/Wis) study vaccine, or to the phosphate buffered saline (PBS; used as placebo), or tobacco allergy.
10) History of anaphylactic allergic reactions to any food, medication or bee sting.
11) Any history of asthma (eg: status asthmaticus, hospitalization for asthma control) or recurrent asthma episodes requiring medical attention in the last 3 years (≥ 1 episode/year).
12) Continuous use of anti-histamines in the last 4 weeks prior to immunization or use of anti-histamines 48 hours prior to study immunization.
13) Have a rash, dermatological condition, tattoos, muscle mass or any other abnormalities at injection site which may interfere with injection site reaction rating.
14) Have received a blood transfusion within 90 days prior to study vaccination.

15) If female, have a positive or doubtful pregnancy test result prior to immunization or lactating females.

16) Vital sign abnormalities: systolic blood pressure ≥150 mmHg and/or diastolic blood pressure ≥90mmHg, heart rate ≤40 beats/min and ≥100 beats/min. Although a vital signs measurement is out of the acceptable ranges, a subject may be included in the study based on Investigator's judgment. Presence of any febrile illness (including oral temperature (OT) ≥38.0°C within 24 hours prior to immunization). Such subjects may be re-evaluated for enrolment after resolution of illness.

17) Cancer or treatment for cancer within 3 years of study vaccine administration. Persons with a history of cancer who are disease-free without treatment for 3 years or more are eligible. Persons with treated and uncomplicated basal cell carcinoma of the skin are eligible. Person with non-treated, non-disseminated local prostate cancer are eligible.

18) Identified as an Investigator or employee of the Investigator or clinical site with direct involvement in the proposed study, or identified as an immediate family member (i.e., parent, spouse, natural or adopted child) of the Investigator or employee with direct involvement in the proposed study.

5.2 Study Visit Procedures

5.2.1 Screening Procedures (up to 30 days prior to study vaccination)

The following procedures will be performed either at screening or in combination with Day 0 but prior to immunization:

- Review and Signature of the Informed Consent Form (ICF). The Investigator or his/her designee, will fully inform the subject of the nature and scope of the study, potential risks and benefits of participation, the study procedures involved and will answer to all questions prior to requesting the subject's signature on the ICF. Subject's consent must be obtained prior to performing any study-related procedures. Source documentation of the ICF being signed prior to any study-related procedures being performed is necessary and a copy of the ICF must be provided to the subject.
- Collect and review demographics (gender, date of birth, age, race, and ethnicity) and body measurements (BMI, weight, and height) data.

  Note: BMI is to be calculated as follows:

  Metric: weight (kg)/height (m)^2
  Non-metric: weight (lb)/height (in)^2 X 703.

  BMI will be rounded to nearest whole number using the standard convention:

  0.1-0.4 round down and 0.5-0.9 round up.

- Collect and review medical history. The medical history should record significant problems active at the time of screening and within the prior year. Problems that have been clinically inactive within the prior year, but which might alter the subject's current or future medical management, should also be noted (e.g., known mitral valve prolapse or history of seizure disorder).
- Review and record current and previous (up to 30 days prior immunization day) medication use.
- Record influenza immunizations received within 24 months prior to the administration of study vaccine and record any adverse reactions following these immunizations.
- Perform vital signs measurements including resting blood pressure (BP), heart rate (HR) and oral temperature (OT should be collected in ºC format; the measurement should be collect not following a recent hot or cold beverage or following smoking). BP will be taken after the subject has been in a seated position for at least 3 minutes. BP should be taken by cuff (manual or automated are acceptable, although the same method should be used throughout the study). BP may be repeated one time following a 5-minute resting period in a seated position, if judged necessary. All values are to be recorded in the source data including the reason for repeating. Inclusion of subjects in regards to out-of-range BP and HR measurement will be based on Investigator's judgment.
- Perform a complete physical examination of the major body systems, including lymphadenopathy (a directed exam for lymph node swelling of the neck and axilla). This examination should be performed by the Investigator (or a Sub-Investigator designated by the Investigator).
- Collect blood samples (approximately 20 mL) for biochemistry, haematology, and serology (screen for HIV and Hepatitis B and Hepatitis C testing) analysis.
- Collect a urine sample for urinalysis (using dipstick).
- Perform serum pregnancy testing for all females.
- Review of inclusion and exclusion criteria and determine subject's eligibility to participate to this study.
- Subjects will be encouraged not to take any over-the-counter (OTC) antipyretics (such as acetaminophen, aspirin, paracetamol, naproxen, or ibuprofen) from 48 hours prior to immunization visit (Day 0).

5.2.2 DOSING VISIT (DAY 0)

Emergency equipment must be available on site and appropriate treatment must be instituted as soon as possible in the event of anaphylaxis or any other immediate hypersensitivity reaction. The Investigator (or a Sub-Investigator designated by the Investigator) will be on site for vaccine administration and remain for at least 30 minutes (and more if deemed necessary) after administration of the vaccine to the last subject. The Investigator (or a Sub-Investigator designated by the Investigator) will be on call for the remainder of the study. If necessary, a physician should be immediately available at the clinical site to administer treatment or to apply procedures for any immediate AEs/SAEs.

The following procedures will be performed at the dosing visit prior to immunization (Day 0; immunization day):

- Record updates in medical history and medications and confirm that the subject continues to meet all inclusion criteria and none of the exclusion criterion since screening.
- Perform a lymphadenopathy examination only if subject complains of arm and/or shoulder pain (a directed exam for lymph node swelling of the neck and axilla). In addition, a history/symptom-directed physical examination of the major body systems could also be performed based on Investigator's opinion. These examinations should be performed by the Investigator (or a Sub-Investigator designated by the Investigator).
- Measurement of vital signs (including BP, HR, OT, and respiratory rate [RR]). BP will be taken after the subject has been in a seated position for at least 3 minutes. OT will be collected using a digital thermometer (in ºC). Inclusion of subjects in regards of BP and HR or pulse results will be based on Investigator's judgment.
- Review BMI. For this visit, only the weight will be measured (height will be obtained from result in the screening visit). Refer to Section 5.2.1, bullet point 2, for formula to calculate BMI.
- Collect a urine sample for all female subjects and perform urine pregnancy testing. No study vaccine must be administered until a negative result is obtained and documented.
- Once eligibility is confirmed, the subject will be randomized to a treatment group or placebo (refer to Section 6.5). The assigned randomization number will be documented on source data.
- Collect blood samples (serology, approximate 10 mL) for HI/MN titer assessment.
- Collect blood samples (approximately 40 mL) on 15 subjects per treatment group for cellular immune assays (PBMC).
- Once all pre-vaccination procedures are completed, the study vaccine will be administered intramuscularly (IM) into the deltoid muscle of the non-dominant arm (if possible) using a 25 gauge needle of sufficient length to reach the substance of the muscle based on the subject's weight/BMI (at least 1 inch or 2.5 cm or longer; for subjects with BMI over than 30, the use of a 1.5 inch or 3.8 cm is recommended). In order to prevent confounding reaction around immunization site, whenever possible blood samples will not be collected from the same arm as the one used for immunization. The arms for blood collection and immunization will be documented.

5.2.2.1 DAY 0, MINIMUM OF 30 MINUTES POST-DOSE OBSERVATION

The following procedures will be performed at the dosing visit after immunization (Day 0; immunization day):

- Subjects will remain in the clinic for at least 30 minutes post-vaccination. The observation period will include an assessment of solicited local and systemic reactions. Any unusual signs or symptoms reported during the initial 30 minutes of observation will prompt continued close monitoring. Based on their condition, subjects may be asked to remain in the clinic for more than 30 minutes after the immunization (reason will be recorded in source data). All data (including assessment of solicited local signs and systemic symptoms) will be recorded in the source document during and after the post-observation period, when applicable. Refer to Section 8.1.1 for Assessment of AE and/or local and systemic reactions.
- During the observation period, subjects will be provided a Memory Aid (MA, or diary card), a measurement device template (in mm) for measuring solicited local reactions, and an oral digital thermometer for recording daily temperature (in ºC). Clinical sites will be responsible to purchase thermometers (in ºC) and measurement devices (in mm) for subjects. The clinic staff will also provide to subjects training on how to use adequately these tools. The subjects will be:
    - Instructed on how to collect their OT with the digital thermometer (in ºC) supplied to them for purposes of the study. From the evening of Day 0 to Day 7 following vaccination, the subject's OT will be taken at approximately the same time each evening and the findings will be recorded in the MA. Subjects will be asked to take their temperature at any other time if they feel feverish and to record the highest temperature of the day in the MA. In which case, they are allowed to take OTC antipyretics (e.g., acetaminophen, aspirin, paracetamol, naproxen, or ibuprofen) and will be advised to increase the frequency of their temperature measurements to approximately every four (4) hours, until no longer febrile (fever defined as a temperature of ≥38.0ºC). Medication intake will be documented in MA and transcribed in source document.
    - Instructed on how to measure any local intolerance symptoms including erythema (redness) and swelling diameter at the injection site using the measurement template supplied to them for purposes of the study. Subjects will be also requested to evaluate pain at the injection site. Local tolerance will be assessed every day starting the evening of Day 0 up to the evening of Day 7 (post-immunization) and recorded on the MA.
    - Instructed to grade, on a daily basis from the evening of Day 0 through the evening of Day 7 following vaccination, each of the solicited systemic symptoms.

The severity of local and systemic symptoms or any AEs will be graded according to the FDA Guidance for industry[18]. Table 9 below presents different severity grades to be used in this study.

Table 9: Severity grade to be used for Local and Systemic Symptoms and other AEs

**Symptoms**	**Severity**				
	**None**	**Grade 1 (Mild)**	**Grade 2 (Moderate)**	**Grade 3 (Severe)**	**Grade 4 (Potential life-threatening)**
**Injection site reactions (local reactions)**					
**Erythema (redness)**	Less than 25 mm	Between 25mm and 50 mm	Between 51 and 100 mm	More than 100 mm	Necrosis or exfoliative dermatitis
**Swelling**	Less than 25 mm	Between 25mm and 50 mm and does not interfere with activity	Between 51 and 100 mm or interfere with activity	More than 100 mm or prevents daily activity	Necrosis
**Pain**	None	Does not interfere with activity	Repeated use of non-narcotic pain reliever >24 hrs or interferes with activity	Any use of narcotic pain reliever or prevents daily activity	Results in a visit to emergency room (ER) or hospitalization
**Solicited systemic reaction of other symptoms**					
**Fever (ºC)**	<38.0ºC	≥38.0 and ≤38.4ºC	≥38.5 and ≤38.9ºC	≥ 39.0 and ≤40.0 ºC	>40.0ºC
**Headache**	None	No interference with activity	Repeated use of non-narcotic pain reliever >24 hours or some interferes with activity	Significant; any use of narcotic pain reliever or prevents daily activity	Results in a visit to emergency room (ER) or hospitalization
**Fatigue or Myalgia**	None	No interference with activity	Some interference with activity	Significant; prevents daily activity	Results in a visit to emergency room (ER) or hospitalization
**Nausea/ Vomiting**	None	No interference with activity or 1-2 episodes/24 hrs	Some interference with activity or >2 episodes/24 hrs	Prevents daily activity, requires outpatient IV hydration	Results in a visit to emergency room (ER) or hospitalization for hypotensive shock
**Diarrhea**	None	2-3 loose stools/ <400gms/24 hrs	4-5 stools or 400-800gms/24 hrs	6 or more watery stools or >800gms /24 hrs or requires outpatient IV hydration	Results in a visit to emergency room (ER) or hospitalization
**Other illness (than listed above)**	None	No interference with activity	Some interference with activity not requiring medical intervention	Prevents daily activity and requires medical intervention	Results in a visit to emergency room (ER) or hospitalization

- Instructed to examine swelling in the neck and axilla and record any feeling and/or swelling in their MA.
- Requested to record their individual data in their MA, as described above.
- Requested to use their MA to support their recall in providing information during the scheduled phone interview conducted on Days 1 and 8 following vaccination.

  - Advised that they will be asked about the occurrence of any symptoms or events requiring medical attention and the use of concomitant medication during the 21-day post-vaccination period as well as throughout the 6-month follow-up period.
  - Informed that they could be requested to bring their MA to each clinic visit. The staff would review the MA entries and grade entries with the subject.
  - Advised that if they experience unsolicited AEs and/or solicited local and systemic reactions of greater than Grade 2 (moderate), they will have to report it promptly (as soon as possible) to the clinical site staff (refer to MA for emergency contact). The Investigator should be informed within 48 hours after the knowledge by the clinical site and may request the subject come to the clinic for evaluation.
  - Advised on emergency contact information and instructions for contacting study personnel. Subjects will be advised to immediately contact the Investigator (or his designee), in the event of a SAE or medical emergency. Subjects will be provided with a phone contact number and be instructed to call if any reaction to vaccination is significant or of concern.
  - Advised to notify their health care professional(s) (e.g., primary care physician) that they are participating in a clinical research study of an influenza vaccine.
- Measurement of vital signs (including BP, HR, RR, and OT). BP will be taken after the 30-minute observation period with the subject in a seated position for at least 3 minutes. Any out-of-range measurements will be assessed by the Investigator (or Sub-Investigator) and any further action will be determined upon his medical decision.
- Provide appointments (date and time) for the next planned visits to the clinical site (Days 3 and 21) and also for the Day 8 phone contact.
- Subjects will be released from the clinical site once all post-vaccination procedures are completed and the subjects have a stable condition.

5.2.3 DAYS 1 AND 8 FOLLOWING VACCINATION PHONE CONTACT. ACCEPTABLE INTERVAL FOR DAY 1: +1DAY; FOR DAY 8: -1DAY/+2 DAYS.

The following procedures will be performed during the phone contacts:

- Subjects will be asked to use their MA to support their recall in providing information during the scheduled phone interview conducted on Days 1 and 8 following vaccination.

  Unsolicited AEs and/or solicited local and systemic reactions of greater than Grade 2 (moderate), they will have to report it promptly (as soon as possible) to the clinical site staff (refer to MA for emergency contact). The Investigator should be informed within 48 hours after the knowledge by the clinical site and may request the subject come to the clinic for evaluation. The Investigator will document review of these events on the source document.

If any of the solicited local or systemic symptoms persist beyond Day 7, these will also be recorded as an AE. In this case, the AE start will be set as 168 hours (7 days) post-vaccination. The subject will be requested to note when the AEs resolved and report this information to the Investigator or clinic staff at the next visit to the clinical site or contact.

- Subjects will also be asked about any difficulties in completing the MA, any change in health, any visits to health care facilities, and/or medical practitioners and use of any concomitant medications.
- Subjects will be reminded of their next appointment (date and time) for the clinical visit and/or the next phone contact.
- Especially at the Day 1 phone contact, subjects will again be instructed to take and record their OT on a daily basis (approximately the same time each evening) during the 7-day safety period and the findings will be recorded in the MA. Subjects will be asked to take their temperature at any other time if they feel feverish (defined as a temperature of ≥38.0ºC) and to record the highest temperature of the day in the MA. In which case, they are allowed take OTC antipyretics (e.g., acetaminophen, aspirin, paracetamol, naproxen, or ibuprofen) and will be advised to increase the frequency of their temperature measurements to approximately every four (4) hours, until no longer febrile.

NOTE: As previously mentioned in this protocol, a maximum of -1 day to +2 days from scheduled visit to the clinic is allowed for the phone contact on Day 8. The subjects will be instructed to perform procedures (refer to Section 5.2.2.1) approximately at the same time each evening from the evening of Day 0 up to the evening of Day 7 (post-immunization). However, the subjects could be reached prior the Day 7 evening, due to the -1 day window. If a subject is reached on Day 7, the phone contact should be performed at least 168 hours after the subject's immunization. Moreover, any solicited or unsolicited signs and symptoms happening on Day 7 after the safety phone call should be recorded on the MA for that day. During the subsequent visit to the clinical site, the subject will be questioned in regard to such events which could have occurred since last contact.

In case the subject cannot be reached via phone, he could be contacted by text message or via email (if these contacts are available). However, the phone should be the initial and preferred way of communication.

5.2.4 DAY 3 BLOOD COLLECTION (ACCEPTABLE INTERVAL -1 DAY/+2 DAYS)

The following procedures will be performed during the safety visit on Day 3:

- Record updates on any change in subjects' health or complaints of lymphadenopathy or any significant visits to health care facilities and/or medical practitioners and use of any concomitant medications and confirm continued eligibility since last visit. Subjects could be asked to bring their MA for that visit.

- Perform a lymphadenopathy examination only if subject complains of arm and/or shoulder pain (a directed exam for lymph node swelling of the neck and axilla). In addition, a history/symptom-directed physical examination of the major body systems could also be performed based on Investigator's opinion. These examinations should be performed by the Investigator (or a Sub-Investigator designated by the Investigator).
- Measure vital signs (including BP, HR, OT, and RR). BP will be taken after the subject has been in a seated position for 3 minutes. OT will be collected using a digital thermometer (in ºC format).
- Perform urinalysis (dipstick) on all subjects.
- Collect blood samples (approximately 15 mL) for biochemistry and haematology.

  Any out of acceptable range clinical laboratory values considered to be a clinically significant change from baseline by the Investigator, will be repeated upon Investigator's request.
- Remind to subjects date and time of their Day 8 phone call and the Day 21 visit.

5.2.5 DAY 21 VISIT BLOOD COLLECTION (ACCEPTABLE INTERVAL: -2 DAYS/+ 3 DAYS)

The following procedures will be performed during the Day 21 visit:

- Record updates on any change in subjects' health or complaints of lymphadenopathy or any significant visits to health care facilities and/or medical practitioners and use of any concomitant medications and confirm that the subject continues to meet all inclusion criteria and none of the exclusion criterion since last visit.
- Perform a lymphadenopathy examination only if subject complains of arm and/or shoulder pain (a directed exam for lymph node swelling of the neck and axilla). In addition, a history/symptom-directed physical examination of the major body systems could also be performed based on Investigator's opinion. These examinations should be performed by the Investigator (or a Sub-Investigator designated by the Investigator).
- Measure vital signs (including BP, HR, OT, and RR). BP will be taken after the subject has been in a seated position for 3 minutes. OT will be collected using a digital thermometer (in ºC format).
- Perform urine pregnancy testing on all females.
- Collect blood samples (serology, approximate 10 mL) for HI/MN titer assessment.
- Collect blood samples (approximately 40 mL) on 15 subjects per treatment group for cellular immune assays (PBMC).
- Provide a MA to subjects, for collection of safety data from Day 21 to Day 201. Subjects will also be provided with a date and time to return to the clinic for the Day 201 follow-up visit.

- Perform monthly phone calls to collect AEs/SAEs and NOCDs following the Day 21 visit until the final visit at Day 201. Subjects should be reached once a month with no more than 45 days between phone calls (use Day 21 date as starting reference). Subjects will be asked about any change in their health, any visits to health care facilities including hospitalizations or emergency room visits and/or medical practitioners, and use of any concomitant medications. The 6-month (180 days) follow up period will start after the Day 21 visit and will, therefore, be up to Day 201. In case the subject cannot be reached via phone, he could be contacted by text message or via email (if these contacts are available). However, the phone should be the initial and preferred way of communication.

NOTE: As previously mentioned in this protocol, a maximum of -2 days to +3 days from scheduled visit to the clinic is allowed for the Day 21 visit and ±14 days are allowed for the Day 201 visit. All effort will be made to have the subjects returned on the planned date for Day 21 activities. If for any reason the visit at Day 21 is done before or after this exact planned date, subsequent visit (Day 201) will be adjusted accordingly.

5.2.6 FINAL VISIT-DAY 201 (ACCEPTABLE INTERVAL IS ±14 DAYS)

The following procedures will be performed during the final visit on Day 201:

- Record updates on any change in subjects' health, complaints of lymphadenopathy, or any significant visits to health care facilities and/or medical practitioners and use of any concomitant medications and confirm that the subject continues to meet all inclusion criteria and none of the exclusion criterion since last visit. Subjects could be asked to bring their MA for that visit.
- Perform a lymphadenopathy examination only if subject complains of arm and/or shoulder pain (a directed exam for lymph node swelling of the neck and axilla). In addition, a history/symptom-directed physical examination of the major body systems could also be performed based on Investigator's opinion. These examinations should be performed by the Investigator (or a Sub-Investigator designated by the Investigator).
- Measure vital signs (including BP, HR, OT, and RR). BP will be taken after the subject has been in a seated position for 3 minutes. OT will be collected using a digital thermometer (in ºC format).
- Collect blood samples (approximately 15 mL) for biochemistry and haematology.

  Any out of acceptable range clinical laboratory values considered to be a clinically significant change from baseline by the Investigator, will be repeated upon Investigator's request.
- Perform urinalysis (dipstick) on all subjects.
- Collect blood samples (serology, approximate 10 mL) for HI/MN titer assessment to evaluate antibody persistence for approximately 6 months following vaccination.
- Collect blood samples (approximately 40 mL) on 15 subjects per treatment group for cellular immune assays (PBMC).

5.3 Selection of the Study Population

5.3.1 RECRUITMENT PROCEDURES

Subjects enrolled in this study will be healthy subjects, aged between 18 to 49 years and will be volunteer members of the community at large. Following IRB approval potential subjects could be contacted and invited to participate in this study, if they meet all inclusion criteria and none of the exclusion criterion.

5.3.2 SUBJECT INFORMATION AND CONSENT

Voluntary written informed consent must be obtained from each subject prior to performing any study-related procedures (including screening procedures). Each subject should be given both verbal and written information describing the nature and duration of the clinical study. The informed consent process should take place under conditions where the subject has adequate time to consider the risks and benefits associated with his/her participation in the study. Subjects should not be screened until they have signed an approved ICF written in a language that is understandable to them.

The IRB-approved ICF should be signed and dated by the subject and the Investigator (or their nominated designee) who conducted the informed consent discussion. The Investigator is responsible for assuring the appropriate content of the ICF and that an informed consent is obtained from each subject in accordance to all applicable regulations and guidelines. The original signed ICF should be retained in the Investigator's files. Each subject should receive a copy of the signed and dated written ICF along with any other written information provided to the subject.

The Investigator should maintain a log of all subjects who sign the ICF and indicate if the subject was randomized and received study vaccine or, if not, the reason why. The subject's medical records (source data) should also document that the ICF was signed and dated prior to any study-related screening procedures being performed.

5.3.3 TEMPORARY CONTRAINDICATIONS

The following conditions constitute contraindications to administration of study vaccine:

- Temperature ≥38.0ºC within 24 hours prior to randomization.
- Acute cold symptoms such as minor upper respiratory tract infection (URTI) symptoms that typically resolve in 48-72 hours prior to randomization.

The following AEs constitute absolute contraindications to administration of study vaccine; if any of these AEs occur during the study, the subject may continue other study procedures at the discretion of the investigator, with the approbation of the Sponsor. The subject must be followed until resolution of the event, as with any AE:

- Any confirmed or suspected immunosuppressive or immunodeficient condition, including HIV infection.

- Discovery of any health condition that, in the investigator's opinion, places the subject at increased risk from receipt of investigational product; or discovery of a change in the subject's status which renders him/her unable to comply with protocol-mandated safety follow-up.
- Pregnancy: Subjects who become pregnant during the study will be followed for safety. The Investigator, or his/her designee, will collect pregnancy information on any subject who becomes pregnant while participating in this study. The Investigator (or his/her designee) will record pregnancy information on the Pregnancy Report Form (for the template of the form, refer to study manual procedures) and submit it to the protocol safety contact (refer to Section 3, under Sponsor contact) within 24 hours of learning of a subject's pregnancy. The subject will be followed to determine the outcome of the pregnancy. At the end of the pregnancy, whether that will be full-term or prematurely, information on the status of the mother and child will be forwarded to the protocol safety contact. Generally, follow-up will be no longer than 6 to 8 weeks following the estimated delivery date. While pregnancy itself is not considered an AE/SAE, any pregnancy complication or elective termination of a pregnancy for medical reasons will be recorded as an AE or a SAE, and will be followed. A spontaneous abortion is always considered to be a SAE and will be reported. Furthermore, any SAE occurring as a result of a post-study pregnancy and considered reasonably related in time to receipt of the investigational product by the investigator, will be reported to the protocol safety contact. While the Investigator is not obligated to actively seek this information from former study subjects, he/she may learn of a pregnancy through spontaneous reporting. Information on pregnancies identified during the screening phase/prior to vaccine administration does not need to be collected; this information need not be communicated to the Sponsor.
- Acute disease at the time of randomization or vaccination. Acute disease is defined as the presence of a moderate or severe illness with or without fever. All vaccines can be administered to persons with a minor illness such as diarrhea or mild URTI with or without low-grade fever.

5.3.4 REMOVAL OF SUBJECTS FROM TREATMENT OR ASSESSMENT

Subjects will be advised that they are free to withdraw from the study at any time without prejudice to their future medical care by the physician or the institution. Subjects who withdraw or are withdrawn from the study after immunization will not be replaced. The primary reason for withdrawal must be recorded in source data and captured in the CRF.

5.3.4.1 CONDITIONS FOR WITHDRAWAL

Every reasonable effort should be made to ensure that each subject complies with the protocol and completes all study visits. However, a subject may withdraw or be withdrawn from participation if:

- The subject withdraws consent,
- The subject displays non-compliance to the terms of their participation in the clinical study (based on Investigator's opinion);

- Safety reasons as judged by Data Safety Monitoring Board (DSMB), the Investigator and/or Medicago;
- Medicago or FDA terminates the clinical study.

In case of withdrawal due to a SAE, a subject will not be replaced. Drop-outs will be subjects who leave the study earlier than planned for whatever reason; drop-outs will not be replaced. Drop-outs who received the study medication will be asked to return to the clinical site for a final assessment. The procedures performed for the final assessment will comprise those of safety assessment done for Day 201. All drop-outs must be reported to Medicago. The reason for drop-out should be documented in the subjects' records and in the appropriate section of the CRF. The CRF must be completed up to and including the time of drop-out/final assessment.

5.3.4.2 LOST TO FOLLOW-UP PROCEDURES

Every attempt will be made to contact study subjects who are lost to follow-up. At least 3 contacts will be attempted and recorded in the source document. As a last resort, one registered letter requesting contact with the site will be sent to any subject with whom the clinic staff no longer has contact. Such contact will be documented in the subject's source documents.

5.3.4.3 SCREEN FAILURES

Screen failures are subjects that have signed the study-specific ICF and are not eligible for enrolment (subjects who were not randomized), due to failure on one or more inclusion or exclusion criteria or because the subject withdrew consent prior to randomization.

Eligible/not treated subjects are subjects that have signed the ICF and are eligible for enrolment, but were either not randomized or randomized and not treated.

Tracking of both classes of screen failures will be carried out within the study records maintained within the participating clinical sites. Neither class of subjects will receive a safety follow-up.

5.3.4.4 FOLLOW-UP OF DISCONTINUATIONS

Subjects who receive a study vaccine will be followed for safety until the end of study (Day 201).

5.3.4.5 MEDICAL HISTORY

The medical history should record significant problems active at the time of screening and within the prior year. Problems that have been clinically inactive within the prior year, but which might alter the subject's current or future medical management, should also be noted (e.g., known mitral valve prolapsed or history of seizure disorder).

5.4 Modification of the Study and Protocol

Modifications to the protocol should be made as an amendment and should be approved both by Medicago and the IRB prior to being implemented unless the amendment is made to eliminate an immediate hazard to the clinical study subjects. Medicago shall be responsible for notifying the USA regulatory authority, FDA, of any amendments to the protocol.

The Investigator is responsible for notifying the IRB of all protocol amendments and ensuring that the IRB has approved any amendment.

Note that administrative changes (e.g., change in monitor phone number) may be implemented without prior IRB approval; however, the Investigator must notify the IRB of any administrative change and ensure that IRB acknowledges receipt of the administrative changes.

5.5 Interruption of the Study

The Investigator (in consultation with the Sponsor), the Sponsor, or Regulatory Authorities may interrupt or terminate this study, for any reason judged valid. The Investigator will immediately, on discontinuance of the clinical study at the clinical site, inform both the clinical study subjects and the IRB responsible for the study of the discontinuance, provide them with reasons for the discontinuance and advise them in writing of any potential risks to the health of the clinical study subjects or other persons. It is the Sponsor's responsibility to report discontinuance of the study to the regulatory agencies, within the appropriate timeframe and providing them with the reasons for the discontinuance, and advise them in writing of any potential risks to the health of clinical study subjects or other persons. The Sponsor must then inform the Investigator that the appropriate notifications were made.

6. TREATMENTS

6.1 Vaccines Administered

The subjects will receive either one of the 3 doses of the study vaccine (i.e. 15 µg, 30 µg, or 60 µg of each strain of the seasonal VLP Influenza Vaccine) or a placebo as an intramuscular injection.

6.2 Identity of Investigational Product

The study vaccine is a VLP quadrivalent seasonal influenza vaccine composed of recombinant H1, H3, and 2 B proteins [hemagglutinins (HA)] from the 4 influenza strains recommended by WHO for vaccination in the Northern hemisphere in 2013-2014, assembled into VLPs and produced in a plant-based (*Nicotiana benthamiana*) transient expression system. The transfer vector used to insert the plasmids (containing the constructs for proteins of interest) into the plant for expression is a bacterium, *Agrobacterium tumefaciens*. The HA protein sequences utilized in the expression constructs were all obtained from viruses isolated from humans, not from egg or cell grown viruses.

Transient expression is a protein expression technology which does not require or use the stable integration process of a foreign gene into the host genome. Transient expression can be achieved by various means, but generally implies the use of a "transfer vector," bacterial, viral, or purely mechanical, which mediates the passage of genetic information in the form of a polynucleotide (in this example, a strand of DNA) into a host cell. Once in the cell, the genetic information is

generally used directly as a template for transcription into mRNA molecules that are subsequently translated into a protein by the host cellular machinery. For further information refer to the Investigator Brochure (IB).

6.2.1 STUDY VACCINE COMPOSITION

The seasonal VLP Influenza vaccine is a clear liquid suspension consisting of a mix of 4 VLPs each bearing the HA of an approximate molecular weight of 72 kDa of one of the following virus strains A/California/7/2009 [H1N1], wild-type A/Victoria/361/2011 [H3N2], B/Brisbane/60/2008 (Victoria lineage) [B/Bris], or B/Massachusetts/2/2012 (Yamagata lineage) [B/Mass]), in a PBS (100 mM PO_4, 150 mM NaCl, 0.01%Tween 80; pH 7-7.6).

The seasonal VLP Influenza Vaccine will be contained in a 2 mL borosilicate vial (type 1). The Hemagglutinin protein concentration in the vaccine vials provided will be 30 µg/mL in a volume of 0.7 mL per vaccine vial or 60 µg/mL in a volume of 1.2 mL. According to the randomization scheme, 0.5 mL from the 30 µg/mL, 0.5 mL from the 60 µg/mL, or 1.0 mL from the 60 µg/mL vaccine vials will be administered to the subjects for final dosages of 15 µg, 30 µg, or 60 µg per strain, respectively (corresponding to 60 µg, 120 µg, or 240 µg in total HA of seasonal VLP vaccine).

6.2.2 PREPARATION AND ADMINISTRATION OF STUDY VACCINE

Study vaccine or placebo will be prepared by 2 staff member at the clinical site as described in the “Preparation of Investigational Study Vaccine/placebo” section of the Study Procedures Manual. Prepared study vaccine (or placebo) will subsequently be administered to subjects by an unblinded staff member.

> NOTE: The personnel responsible for drug preparation can also perform the vaccination (if certified and qualified to do it) as this person would already be unblinded. The unblinded staff members must not be involved in the evaluation of any AEs or reactogenicity evaluation of the subjects following immunization.

Vials with a vaccine concentration of 30 µg/mL per strain (volume of 0.7 mL per vial) or at a concentration of 60 µg/mL per strain (volume of 1.2 mL per vial) will be used in a clean environment in an aseptic manner for preparation for injection as follows:

- For the administration of the 15 µg vaccine dose, the vaccine vials that contain a volume of 0.7 mL of vaccine at concentrations of 30 µg/mL per strain will be used to withdraw 0.5 mL (15 µg per strain of vaccine) to be administered to subjects allocated to the low dose.
- For the administration of the 30 µg vaccine dose, the vaccine vials that contain a volume of 1.2 mL of vaccine at concentrations of 60 µg/mL per strain will be used to withdraw 0.5 mL (30 µg per strain of vaccine) to be administered to subjects allocated to the middle dose.
- For the administration of the 60 µg vaccine dose, the vaccine vials that contain a volume of 1.2 mL of vaccine at concentrations of 60 µg/mL per strain will be used to withdraw 1.0 mL (60 µg per strain of vaccine) to be administered to subjects allocated to the high dose.

The needle used for the injection should be of sufficient length to reach the substance of the muscle. According to randomization scheme, subjects will receive 0.5 mL or 1.0 mL (depending on which vaccine strength to be administered) of one of the 3 doses of the seasonal VLP influenza vaccine, intramuscularly in the deltoid muscle, preferably in the non-dominant arm. Whenever possible, the injection will be given in the opposite arm from which blood samples are drawn.

Elastomer stoppers should never be removed from any vial. After withdrawal of the sufficient volume of formulated vaccine to deliver 0.5 mL or 1.0 mL, the remaining volume of formulated vaccine in each vial will be retained. The 0.5 mL or 1.0 mL vaccine dose administered as well as remaining vaccine volume in vial will be recorded in the Study Drug Administration Record form (refer to Study Procedures Manual) by the clinical site, which will be separate from the study medication record for drug preparation. After drug accountability monitoring is completed, the Investigator (or designee) will return all study vaccines/placebo vials (used and unused vials) in accordance with instructions provided by the Sponsor (refer to Section 6.4.5 for details).

Some variations in the method for vaccine preparation may be used upon agreement by Sponsor but in any case, all vaccine preparation will be documented in appropriate form (refer to Study Procedures Manual). Also, further specific information relating to vaccine or placebo preparation, storage, and shipment will be presented in the Study Procedures Manual.

6.2.3 PRECAUTIONS FOR USE

The study vaccine should be stored at 2ºC to 8ºC but should be at room temperature before administration (should not be administered cold).

<u>Do not freeze</u> vaccine as this destroys activity. Do not use vaccine that has been frozen.

DO NOT SHAKE the vaccine preparations. Do not vortex vaccine.

Study vaccine must not be administered intravenously.

6.2.4 DOSE SELECTION AND TIMING

This study will be a dose escalation, cohort staggering (slow enrolment) for the 3 dose levels (15 µg, 30 µg, and 60 µg VLP per strain) with a placebo-controlled group and will be divided in 3 cohorts.

<u>Cohort 1</u>: Approximately 119 subjects will be randomized and dosed as follows:

- 75 subjects with the 15 µg dose (100% of this treatment group);
- 19 subjects with the 30 µg dose (25% of this treatment group);
- 25 subjects with a placebo (33% of placebo group).

The 7-day safety data after the immunization will be collected by the clinical staff and will be reviewed by the Data and Safety Monitoring Board (DSMB). The DSMB consisting of the PIs, the Sponsor's Medical Officer and 1 external medical advisor; the members will determine if the clinical sites are allowed to continue with the immunization of the second cohort.

Cohort 2: Approximately 100 subjects will be randomized and dosed as follows:

- 56 subjects with the 30 µg dose (75% of this treatment group);
- 19 subjects with the 60 µg dose (25% of this treatment group);
- 25 subjects with a placebo (33% of placebo group).

The 7-day safety data after the immunization will be collected by the clinical staff and reviewed by the DSMB prior to allowing immunization of the third cohort.

Cohort 3: Approximately 81 subjects will be randomized and dosed as follows:

- 56 subjects with the 36 µg dose (75% of this treatment group);
- 25 subjects with a placebo (33% of placebo group).

The Seasonal VLP Influenza Vaccine lot numbers are defined below:

Vaccine concentration of 30µg/mL: Lot number: AAHAW01-140016-011

Vaccine concentration of 60µg/mL: Lot number: AAHAW01-140014-011

6.3 Identity and Composition of the Control Product

The control product (placebo–PBS) will consist of a 100 mM phosphate buffer + 150 mMNaCl + 0.01% Tween 80.

Placebo: Lot number: PPPXX01-130005-013

6.3.1 PREPARATION AND ADMINISTRATION

The control product or PBS (labeled placebo) will be contained in a 2 mL borosilicate vial (type 1) consisting of a volume of 0.75 mL of placebo per vial. For placebo administration, 0.75 mL vials will be used to withdraw 0.5 mL for injection in subjects. The remaining volume of placebo in each vial will be retained. Exact volume of placebo administered as well as remaining placebo volume in vial will be recorded by the clinical site on the Study Drug Administration Record form (refer to Study Procedures Manual), which will be separate from the study medication's record for drug preparation. Used vials will be destroyed according to applicable law.

NOTE: Although some differences can be noticed in the appearance of the different formulations to be injected, the randomization of each subject to treatment or placebo will be maintained blinded. Thus, the site staff involved in the preparation/injection of the vaccine will not be involved in any activity, such as safety evaluation, where a bias could occur. Moreover, as the Investigator will be aware of the aspect of the vaccine formulations, he will not be involved and will not witness vaccine injection; however, the Investigator will be available on study site.

6.3.2 PRECAUTIONS FOR USE

The study vaccine should be stored at 2ºC to 8ºC but should be at room temperature before administration (should not be administered cold).

6.4 Product Logistics

6.4.1 LABELING AND PACKAGING

It is the responsibility of the Sponsor to ensure that supplies provided for this study are manufactured under Good Manufacturing Practices (GMP) and are suitable for human use. It is also the responsibility of the Sponsor to ship a sufficient amount of dosage units to allow the clinical site to maintain an appropriate supply for the study.

The study vaccine and placebo will be packaged and labeled at Medicago in accordance with all applicable regulatory requirements. All products will be placed in secondary packaging and appropriately labeled.

6.4.2 STORAGE AND SHIPMENT CONDITIONS

The study vaccine and the placebo vials will be shipped to the clinical sites. This shipment will also contain: a shipment requisition, preparation and expedition form, as well as an electronic temperature monitor device (as example, TempTale®).

At arrival to the clinical site, vaccines and placebo vials will be immediately placed in a secured and monitored refrigerator that has been stabilized at a temperature of 2 to 8 ºC.

It is requested that the temperature monitor device be stopped upon receipt of the vaccine and the exact date and time is recorded on the device form. The device could be returned to Medicago or alternatively, World Courier that will be used as the transporter could take care of the temperature device, reading of temperature and the transfer of the data to Sponsor.

A written authorization per shipment will be required from the Sponsor contact (refer to Section 3 for details) prior to administering the study vaccine.

A manual log and calibrated thermometer or other temperature recording device will be used to document refrigerator temperature from the time of vaccine reception to the clinic up to the dosing period (up to the last subject dosed). This documentation will be maintained and made available to the Sponsor or designee for review. The site should ensure that appropriate back-up procedures are established for maintenance of the refrigerator temperatures, in the event of a power failure. Such procedures may include an alarm system, back-up electrical system/generator, or a documented contingency plan for maintaining proper refrigeration.

In the event the refrigerator deviates from the 2ºC to 8 ºC required temperature range, the clinical site staff will follow instruction provided in the Study Procedures Manual.

The site should maintain procedures relating to cold-chain maintenance. The Investigator has overall responsibility for ensuring that study vaccines are stored in a safe, locked with limited access location under the specified appropriate storage conditions. Responsibility may be delegated to a nominated member of the study staff, but this delegation must be documented.

6.4.3 PRODUCT ACCOUNTABILITY

The study vaccines and placebo must be used only as directed in the protocol and in Study Procedures Manual. On appropriate form, accountability will be performed for the vaccines and placebo used during the course of the study and also for the remaining study medication (unused). The Investigator will be provided with the appropriate forms to ensure accurate, written records are maintained for receipt and disposition of all vaccines received from the Sponsor.

6.4.4 REPLACEMENT DOSES

Study vaccine and placebo replacement doses will be shipped according to dose level and may be used in the event of breakage or accidental loss while being prepared. All instances must be documented on the appropriate form (refer to Study Procedure Manual).

6.4.5 RETURN OF UNUSED PRODUCTS

The Investigator (or designee) will sign all study vaccine/placebo accountability forms at the end of the vaccination period. Used and unused study vaccine/placebo vials must be retained until the unblinded study monitor (designated by Medicago) completes reconciliation of test article delivery records with disposition records and accountability of used and unused vials. All study vaccines/placebo vials must be accounted for and all discrepancies documented accurately. After drug accountability monitoring is completed, the Investigator (or designee) will return unused vials in accordance with instructions provided by the Sponsor and will destroy all used study vaccine/placebo vials according to the clinical site's procedures and to any applicable USA regulations.

Unused medication (including placebo vials) will be returned to the following address:

Medicago R&D Inc.
3220, rue Watt, suite 104Quebec, Quebec, Canada, G1X 4Z6
Tel.: 1.418.658.9393 ext. 321
E-mail: mercierd@medicago.com
Fax: 1.418.658.6699
Attn: M. Daniel Mercier (Logistic Coordinator)

6.5 Randomization/Allocation Procedures

The site will be provided a pre-determined sequence of randomization numbers according to the randomization code. Randomization will be performed using random block permutations obtained with the PLAN procedure of SAS®. A complete description of the randomization procedure will be described in a Randomization Plan.

Potential study subjects will be screened. Once all screening procedures including Day 0 pre-randomization procedures are completed and the study eligibility is confirmed, the randomization numbers of the pre-determined sequence will be allocated sequentially to subjects within the appropriate treatment group. Once a randomization number has been assigned, it will not be re-used in any event. No subjects will be entered into the study more than once. If a randomization number has been allocated incorrectly, no attempt will be made to remedy the error once study vaccine has been dispensed. The subject will continue with the randomization number and study

vaccine. The study staff will notify the Sponsor as soon as the error is discovered without disclosing the study vaccine administered. Admission of subsequent eligible subjects will continue using the next unallocated number in the sequence.

This randomization number and treatment will be recorded along with the four-digit screening number for each subject on the Investigational Product and Accountability Log (refer to Study Procedures Manual).

If a subject receives a randomization number but is not vaccinated, the following subjects in the assignment group will be randomized with the next randomization number that corresponds to the dismissed subject's treatment sequence. A complete description of the randomization procedure will be described in a Randomization Plan.

6.6 Blinding and Code Breaking Procedures

This is an observer-blind study. A sealed envelope containing the randomization scheme must be kept in a locked, secured location at the clinical site. The randomization code could not be broken except in emergency cases by the unblinded staff member at the clinical site (wherein the Investigator judges that the identification of the treatment is necessary for the management of the event, for appropriate treatment of the subject or if directed by the Agency). Individual envelopes containing the treatment received will also be provided. In case of emergency, only the subject's individual envelope will be opened which will preserve the blinding for other subjects. Any code break will be documented and reported to the Sponsor (refer to Section 3 for Sponsor Contact) in a timely manner. In the event of a non-emergency, the Investigator shall contact the Sponsor Contact (refer to Section 3) to discuss the rationale for unblinding and to determine if the blind should be broken for that subject or not.

This study is blinded through Day 201. Exceptionally, the randomization code will be broken after Day 21 of the study (last subject) for analyses purposes, only (with limited access). This will allow discussions about the clinical data and for public release of data, without having to wait after the 6-month follow-up period for study completion. It is anticipated approximately 5 people from Medicago staff members and few staff (clinic and CRO) will be allowed to see the randomization code. These staff members will not have any contact with the study subjects or to the subjects' data, and will not participate in the evaluation of any adverse reaction or reactogenicity evaluation of the subjects following immunization in order to avoid any bias. This unblinding procedure and targeted people will be documented. Also, the staff at Medicago responsible for doing the immunological analysis in the laboratory will remain blinded as well as the staff at the clinical site (except the staff involved in the preparation/administration of study vaccine and QA Auditor [Quality assurance] as well as QC reviewers [Quality control]).

6.7 Concomitant Therapy

Subjects should be instructed not to introduce any new medications without consulting or notifying the Investigator or designee. Any new or changed medications reported by the subject post-vaccination and through the Day 21 visit, will be recorded. Since AEs may be secondary to new medications, Investigator (or designee) will explore the reasons for the new medications intakes and document these AEs, if any. Concomitant medications will include prescription and over-the-counter (OTC) drugs, vitamins, herbal products, and nutritional supplements (any product viewed by the subject as distinct from food). Investigators and other study staff are

encouraged to capture the most detailed description of vitamins, herbals, and supplements possible; as otherwise, these products may be difficult to classify.

Concomitant medications must be reported in the CRF as indicated below:

- Within 30 days preceding vaccination or at any time from Day 0 through the final study contact (Day 201):
  - Any treatments and/or medications specifically contraindicated (e.g., any immunoglobulins or other blood products, or any immune modifying drugs).
- From Day 0 (day of any study vaccine dose) through Day 21 after vaccination:
  - All concomitant medications/vaccines.

    Note that any changes in concomitant medication intakes (as total daily dose, route of administration, or end date information) provided AFTER the "Day 21" visit will be collected in MA by the subjects. Clinical site staff will record any changes in source data. For the medically-attended event only, the subject's eCRF will be updated with new information, accordingly.
- From Day 0 through the final study contact (Day 201):
  - Any concomitant medication(s) administered to treat a medically-attended AE (defines as any event for which the subject received medical attention defined as hospitalization, an emergency room visit or a visit to or from medical specialist [such as (but not limited to) physician, dentist, physiotherapist, hospital personnel, pharmacist, etc.] regardless reason).
  - Any concomitant medication(s) administered for the treatment of an SAE.
  - Any vaccine not foreseen in the study protocol.
  - Any investigational medication or vaccine.

Given that an important goal of this study is to evaluate the tolerability of the study vaccine, the use of prophylactic medications to prevent or pre-empt symptoms due to vaccination is specifically prohibited over the course of this study. A prophylactic medication is a medication administered in the absence of ANY symptom and in anticipation of a reaction to the vaccination (e.g., an anti-pyretic is considered to be prophylactic when it is given in the absence of fever and any other symptom, to prevent fever from occurring, vitamins used to boost immune system, etc.).

7. SPECIMENS AND CLINICAL SUPPLIES

7.1 Management of Samples

Blood samples for biochemistry, haematology, and serology analyses will be performed at screening; urinalysis and serum pregnancy testing will also be performed at screening. Blood samples for biochemistry and haematology analyses and urinalysis will be repeated at Day 3 and at Day 201. Serology samples for immunogenicity analysis will also be taken at Day 0, 21 and 201. Refer to the Study Laboratory Manual for complete information on the handling and shipment of all laboratory samples.

7.1.1 SAMPLE COLLECTION

Blood samples will be collected at screening, Day 3, and Day 201 for:

Biochemistry analyses as follows: sodium, potassium, urea, creatinine, glucose, alkaline phosphatase (ALP), alaninetransferase (ALT), bilirubin (total), albumin, aspartatetransferase (AST), and gamma glutamyltransferase (GGT).

Haematology analyses as follows: haemoglobin, hematocrit, red blood cells (RBC), platelets, mean cell haemoglobin (MCH), mean cell concentration (MCHC), mean cell volume (MCV), white cell count (WBC total), neutrophils, lymphocytes, monocytes, eosinophils, and basophils.

Urine samples will be collected at screening, Day 3 and Day 201 for:

Urinalysis (using a dipstick) as follows: macroscopic examination (as color and aspect) and pH, specific gravity, glucose, protein, and blood.

Pregnancy testing will also be performed for all females. A serum pregnancy test will be performed during screening and urine pregnancy tests (dipstick) performed on Day 0 prior to randomization and on Day 21 (refer to the Study Laboratory Manual for details on pregnancy test procedures).

The following serology tests will also be conducted at screening: screen for HIV p24 antigen/antibodies, antibody/antigen profile for Hepatitis B and Hepatitis C testing.

In addition, 3 blood samples for serological analysis (HI and MN titer measurements) will be collected from each study subject (~10 mL each time) immediately prior to administration of the study vaccine (Day 0), at Day 21, and at Day 201. Should a subject be discontinued from the study, a sample will be drawn at the time of the final safety visit. Sera will be sent to Medicago for analysis (refer to Section 3 and to the Study Laboratory Manual for complete information about shipments).

Also, 3 blood samples for CMI assays will be collected (approximately 40 mL [4 tubes]) at each timepoint) on 15 subjects from each treatment group prior to the immunization at Day 0, Day 21, and Day 201. The blood samples will be used to prepare PBMCs at the MRA clinical site and will be cryopreserved as quickly as possible following collection (maximum 12 hours). PBMCs will be shipped under nitrogen vapor to an external laboratory (ImmuneCarta Services, refer to Section 3) and held there for later testing.

7.1.2 SAMPLE PREPARATION

Preparation of the laboratory samples for the collection and shipping of biochemistry, haematology and urinalysis will be described in the Study Laboratory Manual. Labeled samples will be ship to Central Clinical Laboratory (refer to Section 3) on the day of collection (or later, if this not alter specimen).

Blood samples for serology (HI or MN tests) will be collected in one (1) 10 mL red top (plain/no additives) tube. The samples will be kept at room temperature for a minimum of 30 minutes and then centrifuged (speed 1200 g) for 10 minutes at ambient temperature. Serum will be divided into 7 aliquots into 7 separate polypropylene tubes as soon as possible: four of at least 0.5 mL (when possible) and the other three containing equal

amounts of the remainder of the serum (when possible). In case, samples cannot be process shortly after the 30-minute period, the samples can be stored in the fridge until centrifugation (for a maximum of 24 hours).

Medicago will measure HI and MN titers in serum samples using validated methods. Serum samples from subjects who do not complete the study through Day 21, regardless of the reason, will be analyzed and reported, but the results will not be used for the study analysis.

Medicago will be responsible to send small quantities of serum to Laboratoires Bio-Medic (refer to Table 7) who will measure the anti-glycan antibodies, using the ImmunoCAP technology.

Blood samples for preparation of PBMC for cell-based assays will be transferred at room temperature from the MRA clinic to ImmuneCarta (refer to Section 3) for samples processing (there should be no more than 3 hours between samples collection and processing). There, peripheral blood mononuclear cells will be isolated according to ImmuneCarta procedures and cryopreserved into 2 to4 aliquots (depending upon cell counts) at ~5-10 x 10^6 cells/mL. These cells will be kept under liquid nitrogen at ImmuneCarta Services facility for further analyses.

NOTE: Some variations in sample preparation may be used upon agreement by Sponsor but in any case, all changes will be documented in appropriate form (refer to Study Procedures Manual). Also, further specific information relating to sample preparation, storage, and shipment will be presented in the Study Laboratory Manual.

7.1.3 SAMPLE STORAGE AND SHIPMENT

Serology samples:

Serology serum samples should be stored at approximately -80ºC (-20ºC is acceptable at the clinical sites prior to shipment) until analysis. Sera must be shipped frozen on dry ice. The Day 0, Day 21, and Day 201 sera samples (first set of aliquots) and back-up sera samples (second set of aliquots), will be sent to Medicago (refer to Section 3 for address and contact) in approximately 4 shipments, according to the following order:

1) Day 0 and Day 21 samples (depend on period between subjects immunization, this shipment could be divided in 2 batches);
2) All Day 0 and 21 back-up samples;
3) Day 201 samples;
4) Day 201 back-up samples.

Once Medicago confirms the reception of the first shipment (first set of aliquots), the back-up samples (second set of aliquots) will be sent. Samples could be sent earlier at Sponsor's request. No samples should be shipped on a Friday or the weekend. An alternate shipment schedule could be used, upon Medicago's approval. All shipments must be accompanied by the shipping manifest, an electronic temperature monitor device (such as TempTale®), and sufficient dry ice to maintain samples integrity (refer to the Study Laboratory Manual for details).

PBMC samples:

Prepared PBMC samples will be stored in liquid nitrogen at MRA until shipped under nitrogen vapor and then stored again in liquid nitrogen until use. PBMCs will be shipped under nitrogen vapor to ImmuneCarta Services (refer to Section 3 for address and contact) in approximately four (4) shipments:

1) Days 0 and 21 samples;
2) Days 0 and 21 back-up samples;
3) Day 201 samples;
4) Day 201 back-up samples.

Once ImmuneCarta Services confirms the receipt of the first shipment, the second set of aliquots of back-up samples will be sent. Samples could be sent earlier at Sponsor's request. No samples should be shipped on Thursday or Friday or the weekend. A different shipment schedule could be used, upon Medicago's approval.

All shipments must be accompanied by the shipping manifest, an electronic temperature monitor device (such as TempTale®), and sufficient dry ice to maintain samples integrity (refer to the Study Laboratory Manual for details).

7.2 Clinical Supplies

Clinical site will provide with serology sera supplies (sampling tubes, aliquot tubes, aliquot labels, aliquot storage, shipping boxes, and accompanying manifests). Disposable syringes, needles, and tubes will be supplied by the clinical site as well. Complete study sample management procedures are described in the Study Laboratory Manual.

8. SERIOUS ADVERSE EVENTS (SAEs) AND ADVERSE EVENTS (AEs)

8.1 Reporting of Serious Adverse Events (SAEs)

A SAE (experience) or reaction is any untoward medical occurrence (whether considered to be related to study vaccine or not) that at any dose:

- Results in Death;
- Is life-threatening (at the time of the event);
- NOTE: The term "life-threatening in the definition of "serious" refers to an event in which the subject was at risk of death at the time of the event; it does not refer to an event which hypothetically might have caused death if it were more severe.
- Requires inpatient hospitalization (≥24 hours) or prolongation of existing hospitalization (elective hospitalizations/procedures for pre-existing conditions that have not worsened are excluded);
- Results in persistent or significant disability/incapacity;
- Is a congenital abnormality/birth defect.

Medical and scientific judgment should be exercised in deciding whether expedited reporting is appropriate in other situations, such as important medical events that may not be immediately life-threatening or result in death or hospitalization but may jeopardize the subject or may require intervention to prevent one of the other outcomes listed in the definition above. These should be considered serious. Examples of such events are intensive treatment in an emergency room for allergic bronchospasm, blood dyscrasias or convulsions that do not result in hospitalization, or development of drug dependency or drug abuse.

See Section 8.1.3 for Initial SAE Reporting by the Investigator and Sponsor representatives.

8.1.1 Adverse Events

An AE or adverse experience is defined as any untoward medical occurrence in a subject or clinical investigation subject administered a pharmaceutical product and which does not necessarily have to have a causal relationship with the treatment. An AE can be any favorable and unintended sign (including an abnormal laboratory finding, for example), symptom, or disease temporally associated with the use of a medicinal product, whether or not considered to be a medicinal product.

The following information regarding each AE will be obtained: date and time of onset and resolution (duration), intensity (defined below), whether it was serious, any required treatment or action taken, outcome, relationship to the investigational vaccine, and whether the AE caused withdrawal from the study.

The intensity of all AEs (including lymphadenopathy [lymph node swelling of the neck and axilla]) will be graded as mild (1), moderate (2) severe (3), or potentially life-threatening (4), according to the FDA Guidance for industry[18]. Refer to Table 9 (Section 5.2.2.1) for grades definition. The Investigator (or delegated Sub-Investigator) will be instructed to closely monitor each subject who experiences an AE (whether ascribed to the investigational product or not) until the outcome of the AE has been determined.

Any AE/SAEs, any signs, symptoms or diseases experienced by the subject following the signature of the ICF but before administration of the vaccine will be recorded in source data as pre-dose events (as a part of subjects' medical histories).

Unsolicited AEs and/or solicited local and systemic reactions of greater than Grade 2 (moderate), they will have to report it promptly (as soon as possible) to the clinical site staff (refer to MA for emergency contact). The Investigator should be informed within 48 hours after the knowledge by the clinical site and may request the subject come to the clinic for evaluation. The Investigator will document review of these events on the source document.

Any clinical laboratory test result that meets the criteria for an AE in the absence of appropriate and/or adequate clinical diagnosis should be reported as an AE, unless the result is considered normal for the current trial subject population, as determined by the Investigator.

If any of the solicited local or systemic reactions persist beyond Day 7, these will also be recorded as an AE. In this case, the AE start will be set as 168 hours (7 days) post-

vaccination. The subject will be requested to note when the AEs resolve and report this information to the Investigator or clinic staff at the next visit at the clinical site or contact.

In the event of a clinically important AE, a suitable sample could be collected for drug assay or for relevant additional laboratory tests.

The clinical importance will be determined according to the Investigator's judgment. The Investigator must ensure that the sample is properly labeled and stored. The Investigator and others responsible for care of the subjects should institute any supplementary investigations of significant AEs based on the clinical judgment of the likely causative factor. This may include seeking a further opinion from a specialist in the field of the AE.

All AEs occurring within 21 days after vaccination must be recorded in the source document and reported in the "Adverse Event" screen in the subject's CRF, irrespective of intensity or whether or not they are considered vaccination-related. Thereafter, through completion of the Day 201 visit, new AEs will be recorded in the source document but will be reported in the CRF only if they meet the definition for an SAE or for a medically attended AE (refer to Section 6.8 for a definition). In any case, all AEs have to be reviewed by the Investigator for their relationship to the vaccine.

Medically relevant AEs will be followed up until complete resolution or based on Investigator's decision. Follow-up of unresolved AEs after Day 201 will continue under the discretion of the Investigator.

8.1.2 EXPECTEDNESS OF AN ADVERSE DRUG REACTION

An "unexpected" adverse reaction is one, in which the nature or severity of the event is not consistent with information in the current Investigator's Brochure. Expedited reporting to FDA is required if an AE is an SAE that is both unexpected and considered possibly related to the study drug.

8.1.3 INITIAL SAE REPORTING BY THE INVESTIGATOR

SAEs related to pre-treatment study procedures will be reported from the time the ICF is signed. All post-vaccination SAEs (treatment-emergent SAEs) will be reported from the time of receiving the study vaccine on Day 0 through to the Day 201 (Final Safety Visit). The Investigator must report via phone, all SAEs, whether considered related to the study vaccine or not, and whether considered unexpected or expected (as defined in Section 8.1.2), to the Sponsor, according to FDA's requirements (within 24 hours (one business day) of the Investigator's learning of the event). The Investigator must also complete, sign, and date the SAE report form, and send via email a copy to:

Dr. Brian J. Ward, M.D., Medical Officer
E-mail: brian.ward@mcgill.ca
Tel.: 1.514.934.1934 ext. 42810
After hours:
Mobile: 1.514.952.6583
Pager: 1.514.921.6953

A copy must also be emailed to:

Sonia Trepanier, Clinical Study Director
trepaniers@medicago.com
Tel.: 1.418.658.9393, ext. 137
Fax: 1.418.658.658.6699
Mobile: 1.418.655.7158

Sebastien Soucy, Clinical Study Manager
soucys@medicago.com
Tel.: 1.418.658.9393, ext. 122
Fax: 1.418.658.6699
Mobile: 1.418.446.203

Sponsor Contact (refer to Section 3) will be responsible to promptly (on the same day as received or at the latest the next business day) transfer the information to the Regulatory Affairs department at Medicago.

8.1.4 FOLLOW-UP REPORTING BY THE PI

All SAEs, regardless of causality, will be followed to resolution until the event returns to the baseline condition, the event stabilizes, or if ongoing at Day 21, the SAE will be followed until resolution, regardless of conclusion of the study. Where appropriate, documentation of any medical tests or examinations performed will be provided to document resolution of the event.

8.1.5 REPORTING OF SAEs OCCURRING AFTER TRIAL TERMINATION

All SAEs occurring during the study period will be followed until resolution or for a period of 30 days from the final study visit at Day 201, regardless of conclusion of the study.

A post-study AE/SAE is defined as any event that occurs outside of the AE/SAE detection period. Investigators are not obligated to actively seek AEs or SAEs in former study subjects. However, if the Investigator learns of any SAE, including a death, at any time after a subject has been discharged from the study, and he/she considers the event reasonably related to the investigational product, the Investigator will promptly notify the Study Contacts for Reporting SAEs (refer to Section 8.1.3).

8.1.6 CAUSAL RELATIONSHIP

The Investigator must make the determination of relationship to the study vaccine for each AE.

The Investigator should decide whether, in his/her medical judgement, there is a reasonable possibility that the event may have been caused by the investigational vaccine. If there is any valid reason, even if undetermined or untested, for suspecting a possible cause-and-effect relationship between the investigational vaccine and the occurrence of the AE, then the AE should be considered “definitely related”, “probably related”, or “possibly related”. Otherwise, if no valid reason exists for suggesting a possible

relationship, then the AE should be classified as "probably not related" or "definitely not related". The following guidance may be helpful:

Definitely Not Related: The AE is clearly not related to the administration of the study vaccine. Another cause of the event is most plausible, and/or a clinically plausible temporal sequence is inconsistent with the onset of the event and the study vaccine administration; and/or a causal relationship is considered biologically implausible.

Probably Not Related: There is no medical evidence to suggest that the AE is related to the study vaccine. The event can be readily explained by the subject's underlying medical condition or concomitant therapy or lacks a plausible temporal relationship to the study vaccine.

Possibly Related: A direct cause and effect relationship between the study vaccine and the AE has not been demonstrated but there is a reasonable possibility that the event was caused by the study vaccine.

Probably Related: There probably is a direct cause and effect relationship between the AE and the study vaccine. A plausible biologic mechanism and temporal relationship exist and there is no, more likely explanation.

Definitely Related: There is a direct cause and effect relationship between the AE and the study vaccine. Reactions at the injection site (redness, swelling, and pain) will automatically be entered as definitely related to the study vaccine.

The medical assessment of intensity will be determined by using the same definitions as for AEs (refer to Section 8.1.1).

The outcome may be classified as resolved, ongoing, resolved with sequelae, or death.

8.1.7 Reporting of SAEs to Health authorities and IRB

Medicago will be responsible for reporting SAEs that are deemed possibly related to study vaccine and unexpected (Unexpected refers to not appearing in the package labeling or in the study vaccine IB) to regulatory authorities in an expedited manner.

The Investigator will be responsible for reporting all SAEs directly to their relevant ethical review body (IRB) as soon as possible, and will also provide the ethical review body with any safety reports prepared by the Sponsor.

8.1.8 Seven (7)-Day Safety Monitoring

A review of 7-day safety data will be carried out to ensure ongoing safety of study subjects as well as maintaining study scientific integrity. This review will detect any early negative trends in the safety data. A phone call reminding study subjects to complete the Memory Aid (e.g. diary card) will be performed at Day 1 post-vaccination. Eight (8) days following the immunization, subjects will be contacted by clinic staff to review and record 7-day safety data. Early accumulated safety outcome data which will include all self-reported solicited local and systemic reactions, any AE/SAEs occurring following administration of the vaccine will be collected and tabulated.

The 7-day safety data of the first cohort will be reviewed by DSMB before allowing the vaccination of the cohort 2. A review of the 7-day safety data of the second cohort will

also be performed by the DSMB before allowing vaccination of cohort 3. During the slow enrolment, the DSMB safety review would be performed on all safety data available in order to confirm whether the clinic site can go ahead with the immunization of the next cohort or not.

Once Day 21 period of the study is completed, statistical analysis of safety data will be performed by comparing the placebo group to each dose level group and the dose levels will be compared to each other. However, due to the timeframe of the analysis involved, the 7- Day safety data may not be monitored for the purposes of the 7-day safety review, but these data will be monitored in advance of the clinical study report.

The safety experts will be provided the following blinded 7-day solicited local and systemic reactions:

- Occurrences of erythema, swelling, and pain at the injection site;
- Occurrences of fever, headache, muscle aches, joint aches, fatigue, chills, malaise, and swelling in the axilla or neck;
- Occurrence of any AE/SAEs;
- OT results from the first 7-day post-vaccination will also be provided;
- Concomitant medications, doctor or emergency room or hospital visits associated with an AE/SAEs.

The occurrence of one or more SAEs considered to be related (i.e., possible, probably or definitely related) will result in a halt to the study at any time during the duration of the study, for further review and assessment of the event(s). These include:

- Any death occurring during the study
- The study be halted for further review and assessment across all sites if 20% of the subjects (in total, regardless of treatment group) experience during the 7-day safety period after vaccination:
    - a severe (Grade 3 or higher) vaccine-related local reaction,
    - a severe (Grade 3 or higher) vaccine-related quantitative systemic
    - reaction,
    - a severe (Grade 3 or higher) vaccine-related subjective systemic reaction for which the severity (grade) is corroborated by study personnel.
- The study will be halted for further review and assessment across all sites if 20% of subjects (in total, regardless of treatment group) experience, during the 21-day evaluation, a severe (Grade 3 or higher) vaccine-related clinical laboratory AE.

All SAEs should be reported to FDA by phone or by facsimile transmission of any unexpected fatal or life-threatening experience associated with the use of the drug as soon as possible but in no event later than 7 calendar days after the Sponsor's initial receipt of the information; and should be followed to resolution, stabilization, or return to baseline regardless of conclusion of the study.

The previous stopping rules will also apply for the duration of the study. If a study subject has a SAE at any time during the study that the investigator determines may be related to the study vaccine (i.e., possibly, probably, or definitely related) then the study must stop

until a full investigation of the event is conducted. At any time during the study, a SAE that was thought to be unrelated to the study vaccine would not warrant stopping the trial.

9. DATA COLLECTION AND MANAGEMENT

9.1 Data Collection, CRF Completion

The CRO will perform data collection, transfer tasks, and monitoring (if applicable). A Clinical Monitor designated by the CRO (and approved by the Sponsor) or the Sponsor will be responsible (or partly responsible) for monitoring the clinical study, in accordance with current federal (US) regulations.

The Investigator will maintain the individual subjects' files. The files will include visit dates of the subject, records of vital signs, medical history or examinations, concomitant medications, any AE's encountered, and any other notes, as appropriate. These documents constitute source documents.

The study data will be entered into a 21 CFR Part 11 compliant CRF using electronic data capture (EDC). The data system will include password protection and internal quality checks such as automatic range checks to identify data that appears inconsistent, incomplete, or inaccurate. All entries on the CRF must be backed up by source data.

Routine laboratory analyses will be performed by a central lab (refer to Section 3). Laboratory data will be delivered to site personnel for entry into the CRF (by standard site procedures including a second person data check).

Queries will be generated for spurious data and clarification sought from the responsible Investigator or delegate at the clinical site. These data queries must be resolved in a timely manner by the clinical site personnel.

The study may be subject to an audit by an authorized representative(s) of Medicago and/or inspections by an authorized Regulatory Authority (e.g., FDA). Regulatory authorities may request access to all study documentation, including source documents for inspection and copying, in keeping with local regulations. The Sponsor will immediately notify the Investigator of an upcoming audit/inspection.

In the event of an audit, all pertinent study-related documentation must be made available to the auditor(s). If an audit or inspection occurs, the Investigator will permit the auditor/inspector direct access to all relevant documents and allocate their time as well as the time of relevant staff to discuss the findings and any relevant recommendations.

Immunogenicity data obtained at Medicago will be transferred to the CRO who will take care of data entry in the SAS® database for statistical analysis.

None of the data to be collected during this trial will be estimated, if missing. Once the final study subject has completed the study evaluations and the CRF have been entered and queries resolved, the database can be locked following appropriate QC/QA procedures.

The Investigator must submit a completed CRF for each subject who receives the study vaccine. CRFs should be signed and dated by the Investigator to document his review of the data and acknowledgement that the data are accurate.

The most recent version of the MedDRA dictionary will be used for coding AEs. The most recent version of the WHO-Drug dictionary will be used for coding concomitant medications.

CRF completion guidelines will be issued separately.

9.2 Data Management

Data management will be performed at INC Research early stage.

10. STATISTICAL METHODS AND DETERMINATION OF SAMPLE SIZE

10.1 General Considerations

Data analysis will be performed by INC Research. A complete description of the statistical analyses and methods will be reported in a Statistical Analysis Plan (SAP) to be finalized before database lock.

A clinical study report will be provided after Day 21. An addendum report containing all results analysis, including data from after Day 21 to Day 201, will be completed.

All descriptive and inferential statistical analyses will be performed using SAS® software (version 9.3 or higher). In general, continuous data will be summarized using descriptive statistics (i.e., mean or geometric mean, median, standard deviation, minimum, and maximum). Categorical data will be summarized using the number and percent of subjects in each category.

Descriptive and inferential statistics will be provided for the safety data. All inferential analyses of the immunogenicity endpoints will use a two-sided alpha level of 5%. No attempt will be made to control type I error rate inflation attributable to multiple comparisons unless otherwise stated.

Demographic and baseline characteristics will be summarized descriptively to assess the comparability between each vaccine group.

10.1.1 ANALYSIS OF PRIMARY SAFETY ENDPOINTS

All safety endpoints will be summarized by treatment group using descriptive statistics.

The following safety endpoints will be assessed:

- Percentage, intensity, and relationship of immediate complaints, 30 minutes post-vaccination;
- Percentage, intensity, and relationship to vaccination of solicited local and systemic signs and symptoms (for 7 days following study vaccine);
- Percentage, intensity, and relationship of unsolicited local and systemic signs and symptoms (for 21 days following study vaccine);
- Occurrences of all AEs/SAEs;
- Occurrences of new onset of a chronic disease (NOCD);
- The number and percentage of subjects with normal and abnormal urine, haematological, and biochemical values at Screening, Days 3 and 201.

The counts and proportions of subjects in each vaccine group with immediate complaints and solicited symptoms will be tabulated by severity and also by relationship; local and

systemic signs and symptoms will be tabulated separately and combined. The proportion of subjects with a non-zero grade, grade >2, grade >3 will be included. The number of subjects experiencing each solicited event and also experiencing immediate complaints will be compared between each vaccine dose level and also between vaccine dose levels and placebo group using Fisher's exact test.

The occurrence of unsolicited (coded) AEs will be tabulated and compared for the time period of 21 days after the vaccination. The occurrence of unsolicited (coded) AEs will also be tabulated by severity and relationship. Occurrence of unsolicited (coded) AEs will be compared (each vaccine dose level versus placebo) using Fisher's exact tests and also between vaccine dose groups.

AE incidence rates will be compared between pairs of vaccine groups using Fisher's exact tests; statistical comparisons will only be made for all vaccine-emergent AEs. Given the multiplicity of comparisons contemplated, the safety analyses for both solicited reactions and spontaneous AEs will feature markedly elevated type I error; however, no multiple comparisons corrections will be applied. Because of the increased probability of observing significant differences with multiple comparisons, patterns of AE rates and p-values will be examined rather than focusing on individual p-values. Additionally, since the study was not powered to detect differences in AE rates between treatment groups, it is understood that if significant differences are not apparent that this does not mean that they are non-existent.

Laboratory data and vital signs at each study timepoint will be summarized using descriptive statistics.

10.1.2 ANALYSIS OF PRIMARY IMMUNOGENICITY ENDPOINTS

The GMFR and GMT will be compared (each vaccine dose level compared together and also versus placebo at Day 21) using analysis of variance (ANOVA). Each dose group will be compared to each other and to placebo and multiple comparisons will be controlled using Dunnett's Test.

The primary analysis will be supplemented by pair-wise comparisons of each dose group using two-sided, 95% confidence limits on the treatment group difference in GMT ratios, based on the least-squares mean differences from the ANOVA model.

The difference in seroconversion and seroprotection rates between each vaccine dose level versus placebo and also between active dose groups will be assessed using Fisher's exact test. Descriptive statistics and 95% CIs will be calculated for the immunological parameters.

10.1.3 ANALYSIS OF SECONDARY IMMUNOGENICITY ENDPOINTS

Refer to Section 2.2.1 for a description of the secondary immunogenicity endpoints. GMT, GMFR and seroconversion rates will be compared between each vaccine dose level versus placebo and also between active dose groups at Day 21 using the same methods as those described for the primary immunogenicity endpoints.

Seroprotection rates (based on HI antibody response) will be compared (between each vaccine dose level versus placebo and also between active dose groups) using Fisher's

exact tests at Day 21. The difference and the 95 % CI on the difference between the proportions of seroconverted subjects in each dose group compared to placebo will be assessed using Fisher's exact method.

Number of subjects and proportion of subjects with a detectable HI antibody titer (≥LOQ) at Day 0, Day 21, and Day 201 will be tabulated. The number of subjects with a test result at each timepoint will be used as the denominator.

10.1.4 Exploratory Immunogenicity endpoints

Refer to section 2.3 for the description of the exploratory endpoints.

Data from exploratory endpoints will not be included in the clinical study report. If required, ANOVA or Fisher's exact tests will be performed on continuous or categorical endpoints respectively. Since the number of samples collected for exploratory endpoints may be small, nonparametric methods will be used in the event that the distribution of data does not meet the normality assumption.

10.2 Analysis Populations

10.2.1 Definition of Population

10.2.1.1 Safety Analysis Set

The Safety Analysis Set is defined as all subjects who received any study treatment. All safety analyses will be performed using the Safety Analysis set.

10.2.1.2 Full Analysis Set

The Full Analysis Set (FAS) will consist of the subset of the Safety Set with a Day 0 and any post-vaccine immunogenicity assessment. Subjects who received an incorrect treatment will be analyzed as randomized. All primary and secondary immunogenicity endpoints will be analyzed using the FAS.

10.2.1.3 Per-Protocol Immunogenicity Population

The Per Protocol Set (PP) will consist of the subset of the FAS who completed the study without a major protocol violation.

For the Day 21 analysis, this should include the subjects who received the vaccine dose and had Day 0 and Day 21 immunogenicity measurements. Subjects who had blood samples for immunogenicity taken outside of the time window are to be excluded from the PP set for this specific visit. Subjects who received the wrong vaccine, but for whom the treatment received can be unequivocally confirmed, will be analyzed in the PP set as treated, provided they have no other violations that compromise their data.

For the Day 201 analysis, this should include the subjects who received the vaccine dose and had Day 0 and Day 201 immunogenicity measurements. The Day 201 PP set is defined for data summaries of data collected at the Day 201 visit. Inclusion in this population has no impact on data summaries at other visit.

The immunogenicity endpoints of seroconversion factor, seroconversion rate and seropositive rate will be performed using the PP set as sensitivity analyses.

10.3 Determination of Sample Size

The sample size needed to meet CBER's criteria for seroprotection in adults: the lower bound of the 95% CI for the percentage of subjects achieving an HI reciprocal ≥40 should meet or exceed 70%.

Based on the HI titers results obtained for the quadrivalent vaccine tested in the previous clinical study in adults (CP-Q12VLP-004) and presented in Introduction, statistical analysis were performed to recommend a proper sample size. Statistical analysis was performed on the seroprotection criteria as this criterion is usually more difficult to achieve. Based on this analysis, in a treatment group of 75 subjects, seroprotection rates of at least 81% should have a lower bounded 95 % exact confidence limit that is above the desired percentage of 70% for the 4 vaccine strains. This study has not been powered to show differences between treatment groups, but it may be possible to show substantial differences between active treatments and placebo.

A study of this size is not powered to detect rare AEs; however, it will be possible to show substantial differences in rates of AEs. For AEs with an incidence of 4.5%, the probability of observing at least 1 event in a sample size of 75 is 97%.

10.4 Handling of Missing Data

Refer to the SAP for details regarding missing immunogenicity data.

11. ETHICAL AND LEGAL ISSUES AND PI/SPONSOR RESPONSIBILITIES

11.1 Ethical Conduct of the Trial / Good Clinical Practice (GCP)

The study will be conducted in accordance with the ethical principles that have their origins in the Declaration of Helsinki, International Conference on Harmonization (ICH) Guideline E6, GCP rules, and applicable USA regulatory requirements.

The Investigator is responsible for obtaining written approval from the IRB for the clinical study protocol (including all protocol amendments), the written ICF, informed consent updates, subject recruitment procedures (e.g., advertisements), and any other written information to be provided to the subject, which complies with local regulatory requirements.

The Investigator is responsible for maintaining a copy of the approval document in the study documentation files and for providing a copy to Medicago.

The Investigator should submit written reports of the clinical study status to the IRB annually or more frequently, if requested by the IRB. A final study notification should be forwarded to the IRB after the study has been completed or in the event of premature termination of the study. A copy of all clinical study status reports (including termination) should be provided to Medicago. The end of the trial is defined as the date of the last contact with any subject in the trial. The end of the trial must be notified to the IRB within 90 days or within 15 days if the trial is halted or terminated early.

Following the reporting of serious adverse reactions and events to Medicago (Section 8.1.1), the Investigator must also promptly inform the IRB of these; or other safety related information reported from Medicago.

11.2 Source Documents and Source Data

Source documents are original documents, data, and records of clinical findings, observations and other activities in a clinical study necessary for the reconstruction and evaluation of the study. Source documents will include: all logs and notes from the clinical site, laboratory records specific to this study, and relevant hospital records, clinical and office charts, laboratory notes, memoranda, evaluation checklists, pharmacy (or clinical site) dispensing records, recorded data from automated instruments, copies or transcriptions certified after verification as being accurate copies, microfiches, photographic negatives, microfilm or magnetic media, x-rays, subject files and records kept at the pharmacy (or clinical site), at the laboratories and at medical/technical departments involved in the clinical study.

11.3 Confidentiality of Data and Access to Subject Records

Data collected during this study will be used to support the development, registration, and future marketing of VLP seasonal influenza vaccine. All data collected during the study will be controlled by the Sponsor or designee and the Sponsor will abide by all relevant data protection laws. After subjects have consented to take part in the study, their medical records and data collected during the study will be reviewed by representatives of the Sponsor and/or the company organizing the research on the Sponsor's behalf to confirm that the data collected is accurate and for the purpose of analyzing the results. These records and data may additionally be reviewed by auditors, interested commercial parties or by regulatory authorities. Outside of the clinical site, subjects will be referred to by a unique subject number rather than by name. The results of this study may be used in submissions to other countries throughout the world, which have ensured an adequate level of protection for personal data.

To ensure compliance with relevant regulations, data generated by this study must be available for inspection upon request by the Sponsor or an authorized representative of Medicago, regulatory authorities and the IRB.

11.4 Monitoring, Auditing and Archiving

11.4.1 MONITORING

The study will be monitored by representatives of Medicago throughout its duration. Moreover, representatives of the Sponsor will participate to the Site Initiation and Close-out visits as well as to one dosing day, at the Investigator's facilities. Other visits by Sponsor to the clinical site(s) are not excluded. Visits by Sponsor staff should be scheduled at mutually agreeable times periodically throughout the study. These visits will be conducted to evaluate the progress of the study, verify the rights and well-being of the subjects are protected, verify the reported clinical study data are accurate, complete and verifiable from source documents, and the conduct of the study is in compliance with the approved protocol and amendments, GCP and applicable regulatory requirements. A monitoring visit should include a review of the essential clinical study documents (regulatory documents, CRFs, source documents, drug dispensing records, subject ICFs,

etc.) as well as discussion on the conduct of the study with the Investigator and staff. Blinded and unblinded monitors will be assigned depending on the record to be reviewed.

The Study Monitor should conduct these visits as frequently as appropriate for the clinical study. The Investigator and staff should be available during these visits to facilitate the review of the clinical study records and resolve/document any discrepancies found during the visit.

11.4.2 AUDITS AND INSPECTIONS

In accordance with the principles of GCP, the study may be inspected by regulatory authorities, the Sponsor or assigned designee. The Sponsor is entitled to access information about the status of the study and to review the original study documents or site documents supporting the study.

During the course of the study, Quality Assurance (QA) auditors may audit the investigational site. As Medicago representatives, they are entitled to access all subject and study information, excluding the randomized treatment assignments.

11.4.3 ARCHIVING

The Investigator will retain records, reports and source documentation including: the original ICFs, study documentation (e.g., IRB approval letters, etc.) and a copy of the completed CRFs for 2 years after licence approval or 2 years after the last shipment of the product for investigational use is discontinued and the FDA notified, as per US Food and Drug Regulations, 21 CFR 312.57(c). The Investigator must contact Medicago prior to the disposal of any study documents (Adopted from ICH guidelines, GCP, Section 4.9.5). If before the retention period is completed, the Investigator or institution can no longer maintain the study documentation, Medicago should be contacted to make other arrangements for storage.

11.5 Financial Contract and Insurance Coverage

Medicago has suitable insurance to cover clinical trials and product liability. The clinical sites must have suitable insurance to cover negligence and default liability.

11.6 Stipends for Participation

Subject stipends for clinical study participation must be clearly outlined in the ICF and approved by the IRB.

12. REFERENCES

[1] Osterholm MT, Kelley NS, Sommer A, Belongia EA. Efficacy and effectiveness of influenza vaccines: a systematic review and meta-analysis. Lancet Infect Dis 2012;12(1):36-44.

[2] Palache A. Seasonal influenza vaccine provision in 157 countries (2004-2009) and the potential influence of national public health policies. Vaccine 2011;29:9459-9466.

[3] Molinari NA, Ortega-Sanchez IR, Messonnier ML, Thompson WW, Wortley PM, Weintraub E, Bridges CB. The annual impact of seasonal influenza in the US: measuring disease burden and costs. Vaccine 2007;25:5086-5096.

[4] DiazGranados CA, Denis M, Plotkin S. Seasonal influenza vaccine efficacy and its determinants in children and non-elderly adults: a systematic review with meta-analyses of controlled trials. Vaccine 2012;31:49-57.

[5] Chiu C, Wrammert J, Li GM, McCausland M, Wilson PC, Ahmed R. Cross-reactive humoral responses to influenza and their implications for a universal vaccine. Ann N Y Acad Sci 2013;1283:13-21.

[6] Gatherer D. Passage in egg culture is a major cause of apparent positive selection in influenza B hemagglutinin. J Med Virol 2010;82:123-127.

[7] Kim TS, Sun J, Braciale TJ. T cell responses during influenza infection: getting and keeping control. Trends Immunol 2011;32:225-231.

[8] Kreijtz JH, Fouchier RA, Rimmelzwaan GF. Immune responses to influenza virus infection. Virus Res 2011;162:19-30.

[9] McElhaney JE. Influenza vaccine responses in older adults. Ageing Res Rev 2011;10:379-388.

[10] Galli G, Medini D, Borgogni E, Zedda L, Bardelli M, Malzone C, Nuti S, Tavarini S, Sammicheli C, Hilbert AK, Brauer V, Banzhoff A, Rappuoli R, Del Giudice G, Castellino F. Adjuvanted H5N1 vaccine induces early CD4+ T cell response that predicts long-term persistence of protective antibody levels. Proc Natl Acad Sci U S A 2009;106:3877-3882.

[11] Appay V, van Lier RA, Sallusto F, Roederer M. Phenotype and function of human T lymphocyte subsets: consensus and issues. Cytometry A 2008;73:975-983.

[12] Farber DL, Yudanin NA, Restifo NP. Human memory T cells: generation, compartmentalization and homeostasis. Nat Rev Immunol 2014;14:24-35.

[13] Seder RA, Darrah PA, Roederer M. T-cell quality in memory and protection: implications for vaccine design. Nat Rev Immunol 2008;8:247-258.

[14] FDA Guidance for industry, Clinical data needed to support the licensure of seasonal inactivated influenza vaccines, May 2007. http://www.fda.gov/biologicsbloodvaccines/guidancecomplianceregulatoryinformation/guidances/vaccines/ucm074794.htm

[15] Foetisch K, Westphal S, Lauer I, Retzek M, Altmann F, Kolarich D, Scheurer S, Vieths S. Biological activity of IgE specific for cross-reactive carbohydrate determinants. J Allergy Clin Immunol 2003;111:889-896.

[16] Fotisch K, Altmann F, Haustein D, Vieths S. Involvement of carbohydrate epitopes in the IgE response of celery-allergic patients. Int Arch Allergy Immunol 1999;120:30-42.

[17] Westphal S, Kolarich D, Foetisch K, Lauer I, Altmann F, Conti A, Crespo JF, Rodriguez J, Enrique E, Vieths S, Scheurer S. Molecular characterization and allergenic activity of Lyc e 2 (beta-fructofuranosidase), a glycosylated allergen of tomato. Eur J Biochem 2003;270:1327-1337.

[18] FDA Guidance for industry, Toxicity grading scale for healthy adult and adolescent volunteers enrolled in preventive vaccine clinical trials, September 2007. http://www.fda.gov/biologicsbloodvaccines/guidancecomplianceregulatoryinformation/guidances/vaccines/ucm074775.htm
