# Supplementary material for: Immunogenicity and safety of a quadrivalent plant-derived virus like particle influenza vaccine candidate—Two randomized Phase II clinical trials in 18 to 49 and ≥50 years old adults
Source: PLoS One. 2019 Jun 5;14(6):e0216533. doi: 10.1371/journal.pone.0216533 (PMC6550445; doi:10.1371/journal.pone.0216533)
Supplement: S2 File — Clinical study: Immunogenicity, safety, and tolerability of a Plant-derived seasonal VLP quadrivalent influenza vaccine in elderly. (PDF) [file pone.0216533.s008.pdf]

MEDICAGO

IMMUNOGENICITY, SAFETY, AND TOLERABILITY OF A PLANT-DERIVED SEASONAL VLP QUADRIVALENT INFLUENZA VACCINE IN ELDERLY

**PROTOCOL NUMBER:**	CP-Q13VLP-008
**INVESTIGATIONAL PRODUCT:**	Seasonal VLP Quadrivalent (H1N1, H3N2, B/Brisbane and B/Massachusetts) Influenza Vaccine
**DEVELOPMENT PHASE:**	Phase 2
**SPONSOR:**	Medicago R&D Inc.<br>1020, route de l'Eglise, bureau 600<br>Sainte-Foy, Quebec, Canada, G1V 3V9<br>Tel.: 1.418.658.9393
**PRINCIPAL INVESTIGATORS (PIs):**	Dr. Michael Libman, M.D.<br>MUHC-Vaccine Study Centre<br>14770 Boulevard Pierrefonds, suite 204<br>Pierrefonds, Quebec, Canada H9H 4Y6<br>Tel.: 1.514.624.7855<br><br>Dr. Guy Boivin, M.D.<br>Centre de recherche-CHU de Québec (CR-CHUQ)<br>2400 d'Estimauville,<br>Quebec, Quebec, Canada, G1E 7G9<br>Tel.: 1.418.525.4444<br><br>Dr. Richard Larouche, M.D.<br>inVentiv Health Clinique (IVH)<br>2500 Einstein<br>Quebec, Quebec, Canada, G1P 0A2<br>Tel.: 1.418.527.4000
**SPONSOR RESPONSIBLE MEDICAL OFFICER:**	Dr. Brian J. Ward, M.D.<br>Research Institute of the MUHC<br>Montreal General Hospital<br>1650 Cedar Avenue, Room L10-509<br>Montreal, Quebec, Canada, H3G 1A4<br>Pager: 1.514.921.6953
**CRO (Data Management, Biostatistics and Medical Writing):**	inVentiv Health Clinique<br>2500 Einstein<br>Quebec, Quebec, Canada, G1P 0A2<br>Tel.: 1.418.527.4000
**PROTOCOL DATE AND VERSION:**	June 23rd, 2014 – Final-Version 1.0

Information in this protocol is confidential and should not be disclosed, other than to those directly involved in the execution or the ethical review of the study, without written authorization from Medicago R&D Inc. (Herein after known as "Medicago"), and its affiliates. This study will be conducted in accordance with applicable Good Clinical Practice (GCP) guidelines, International Conference on Harmonization (ICH) guidelines, and other applicable Canadian regulatory requirements.

TABLE OF CONTENTS

TABLE OF CONTENTS 2
LIST OF TABLES 5
LIST OF FIGURES 5
PROTOCOL AGREEMENT 6
PROTOCOL SIGNATURE PAGE 7
SYNOPSIS 8
LIST OF ABBREVIATIONS 18
1. INTRODUCTION 20
1.1. BACKGROUND 20
1.2. BACKGROUND OF THE INVESTIGATIONAL PRODUCT 22
1.3. CLINICAL STUDIES - RESULTS FROM PHASE 1-2 TRIAL IN HEALTHY ADULTS 18-49 YEARS OF AGE 22
1.3.1 Safety *22*
1.3.2 Antibody Response *23*
1.3.3 Results for Plant Glycans *26*
1.3.4 Results for CMI *27*
1.4. CLINICAL STUDIES - SAFETY RESULTS FROM PHASE 2 TRIAL WITH ALUM-ADJUVANTED H5 VLP VACCINE IN HEALTHY ADULTS 18-64 YEARS OF AGE. 32
1.5. PRE-CLINICAL STUDIES 33
2. TRIAL OBJECTIVES AND ENDPOINTS 33
2.1 PRIMARY OBJECTIVES 33
2.1.1 Primary Endpoints *34*
2.2 SECONDARY OBJECTIVES 36
2.2.1 Secondary endpoints and measures *36*
2.3 EXPLORATORY OBJECTIVES 37
2.3.1 Exploratory Endpoints and measures *37*
3. INVESTIGATOR AND TRIAL CENTER 40
4. ETHICS APPROVAL 41
4.1 ETHICAL CONDUCT OF THE STUDY 41
5. INVESTIGATIONAL PLAN 41
5.1 DESCRIPTION OF THE OVERALL TRIAL DESIGN AND PLAN 41
5.1.1 Inclusion Criteria *45*
5.1.2 Exclusion Criteria *46*
5.2 STUDY VISIT PROCEDURES 48
5.2.1 Screening Procedures (up to 90 days prior to study vaccination) *48*
5.2.2 Dosing Visit (Day 0) *49*
5.2.3 Days 1 and 8 following vaccination phone Contact (acceptable interval for Day 1: +1 day; for day 8:-1 day/+2 days) *53*
5.2.4 Day 3 Blood collection (acceptable interval -1 day/+2 days) *55*
5.2.5 Day 21 Visit blood collection (acceptable interval: -2 days/+ 3 days) *55*

5.2.6 Day ≥28; phone Contact 7 days following vaccination with the licensed flu vaccine (acceptable interval ±4 days) ... 56
5.2.6 Day ≥42; Visit approximately 21 days following vaccination with the licensed vaccine (acceptable interval up to +56 days following vaccination; or Day ≥42 +35days window)57
5.2.7 Final Visit-Day 201 (acceptable interval is -14 days/+80 days) ... 57
5.3 SELECTION OF THE STUDY POPULATION ... 58
5.3.1 Recruitment Procedures ... 58
5.3.2 Subject Information and Consent ... 58
5.3.3 Temporary Contraindications ... 59
5.3.4 Removal of Subjects from Treatment or Assessment ... 60
5.4 MODIFICATION OF THE STUDY AND PROTOCOL ... 61
5.5 INTERRUPTION OF THE STUDY ... 61

6. TREATMENTS ... 61

6.1 VACCINES ADMINISTERED ... 61
6.2 IDENTITY OF INVESTIGATIONAL PRODUCT ... 62
6.2.1 Study vaccine composition ... 62
6.2.2 Preparation and Administration of study vaccine ... 62
6.2.3 Precautions for Use ... 64
6.2.4 Dose Selection and Timing ... 64
6.3 IDENTITY AND COMPOSITION OF THE CONTROL PRODUCT ... 65
6.3.1 Preparation and administration ... 65
6.3.2 Precautions for use ... 66
6.4 IDENTITY AND COMPOSITION OF OTHER PRODUCTS USED IN THIS STUDY ... 66
6.4.1 Preparation and Administration ... 66
6.4.2 Precautions for use ... 66
6.5 PRODUCT LOGISTICS ... 66
6.5.1 Labeling and Packaging ... 66
6.5.2 Storage and Shipment Conditions ... 67
6.5.3 Product Accountability ... 67
6.5.5 Return of Unused Products ... 68
6.6 RANDOMIZATION/ALLOCATION PROCEDURES ... 68
6.7 BLINDING AND CODE BREAKING PROCEDURES ... 69
6.8 CONCOMITANT THERAPY ... 69

7. SPECIMENS AND CLINICAL SUPPLIES ... 70

7.1 MANAGEMENT OF SAMPLES ... 70
7.1.1 Sample Collection ... 70
7.1.2 Sample Preparation ... 71
7.1.3 Sample Storage and Shipment ... 72
7.2 CLINICAL SUPPLIES ... 72

8. SERIOUS ADVERSE EVENTS (SAES) AND ADVERSE EVENTS (AES) ... 73

8.1 REPORTING OF SERIOUS ADVERSE EVENTS (SAES) ... 73
8.1.1 Adverse Events ... 73
8.1.2 Expectedness of an Adverse Drug Reaction ... 75
8.1.3 Initial SAE Reporting by the Investigator ... 75
8.1.4 Follow-up Reporting by the PI ... 75
8.1.5 Reporting of SAEs Occurring after Subject Trial Termination ... 76
8.1.6 Causal Relationship ... 76
8.1.7 Reporting of SAEs to Health authorities and IRB/REB ... 77

8.1.8 Seven (7)-Day Safety Monitoring ... 77

9. DATA COLLECTION AND MANAGEMENT ... 78

9.1 DATA COLLECTION, CRF COMPLETION ... 78
9.2 DATA MANAGEMENT ... 79

10. STATISTICAL METHODS AND DETERMINATION OF SAMPLE SIZE ... 79

10.1 GENERAL CONSIDERATIONS ... 79
10.1.1 Analysis of Primary Safety endpoints ... 80
10.1.2 Analysis of Primary immunogenicity Endpoints ... 81
10.1.3 Analysis of Secondary Immunogenicity Endpoints ... 81
10.1.4 Exploratory Immunogenicity endpoints ... 82
10.2 ANALYSIS POPULATIONS ... 82
10.2.1 Definition of Population ... 82
10.3 DETERMINATION OF SAMPLE SIZE ... 83
10.4 HANDLING OF MISSING DATA ... 83

11. ETHICAL AND LEGAL ISSUES AND PI/SPONSOR RESPONSIBILITIES ... 83

11.1 ETHICAL CONDUCT OF THE TRIAL / GOOD CLINICAL PRACTICE (GCP) ... 83
11.2 SOURCE DOCUMENTS AND SOURCE DATA ... 84
11.3 CONFIDENTIALITY OF DATA AND ACCESS TO SUBJECT RECORDS ... 84
11.4 MONITORING, AUDITING AND ARCHIVING ... 84
11.4.1 Monitoring ... 84
11.4.2 Audits and Inspections ... 85
11.4.3 Archiving ... 85
11.5 FINANCIAL CONTRACT AND INSURANCE COVERAGE ... 85
11.6 STIPENDS FOR PARTICIPATION ... 85

12. REFERENCES ... 85

13. APPENDICES ... 87

LIST OF TABLES

Table 1: Study visit procedures

Table 2: Incidence of systemic and local reactions within 7 days after the vaccination[1]

Table 3: Serum Hemagglutination-Inhibition Antibody Titers (Full set analysis)

Table 4: Serum Neutralizing Antibody Titers (non-audited data)

Table 5: IgE responses to plant glycans according to the bromelain assay

Table 6: Sub-populations of memory T-cell based on the expression of cell-surface markers

Table 7: Part A: Solicited Signs and Symptoms (Safety Set) for Phase II trial with H5 VLP Vaccine

Table 8: Sites and Responsible Key Personnel

Table 9: Dose levels to be used in this phase 2 trial (Immunization on Day 0)

Table 10: Severity grade to be used for Local and Systemic Symptoms and other AEs

Table 11: Vaccine formulations

LIST OF FIGURES

Figure 1: Relative distribution of the CD4 T cell functional response to *ex vivo* stimulation with each of the 4 VLPs included in the quadrivalent VLP vaccine.

Figure 2: Details of the functional response of the CD4 T-cell based on the expression of the 4 markers (CD107a, IFN-γ, IL-2 and TNF-α) after *ex vivo* stimulation with each of the 4 VLPs included in the quadrivalent VLP vaccine 21 days after immunization.

Figure 3: Details of the functional response of the TM CD4 T-cells based on the expression of the 4 markers (CD107a, IFN-γ, IL-2 and TNF-α) after *ex vivo* stimulation with each of the 4 VLPs included in the quadrivalent VLP vaccine 21 days after immunization.

Figure 4: Percentage of total cross-reactive CD4 (left) and CD8 (right) T-cell to B/Massachusetts HA protein peptide pool.

Figure 5: Details of the functional response of CD4 (A) and TM CD4 T-cells based on the expression of the 4 markers (CD107a, IFN-γ, IL-2 and TNF-α) after *ex vivo* stimulation with B/Massachusetts HA protein peptide pool 21 days after immunization.

Figure 6: Proposed study schedule with approximate enrolment dates.

PROTOCOL AGREEMENT

I have read this Medicago Protocol No. CP-Q13VLP-008

Title: Immunogenicity, Safety, and Tolerability of a Plant-Derived Seasonal VLP Quadrivalent Influenza Vaccine in Elderly

I have fully discussed the objectives of this study and the contents of this protocol with the Sponsor's representative.

I understand that the information in this protocol is confidential and should not be disclosed, other than to those directly involved in the execution or the ethical review of the study, without written authorization from Medicago R&D Inc. It is, however, permissible to provide information to a subject in order to obtain consent once Investigational Review Board (IRB)/Research Ethics Boards (REB) approval is obtained.

I agree to conduct this study according to this protocol and to comply with its requirements, subject to ethical and safety considerations and guidelines, and to conduct the study in accordance with International Conference on Harmonization (ICH) guidelines on Good Clinical Practice (GCP) and other applicable regulatory requirements.

I understand that the Sponsor may decide to suspend or prematurely terminate the study at any time for whatever reason and that such a decision will be communicated to me in writing. Conversely, should I decide to withdraw from execution of the study I will communicate my intention immediately in writing to the Sponsor.

Investigator Name and Address

Michael Libman, M.D.
MUHC-Vaccine Study Centre
14770 Boulevard Pierrefonds, suite 204, Pierrefonds, Quebec, Canada H9H 4Y6

Signature   Date

Guy Boivin, M.D.
Centre de recherche-CHU de Québec (CR-CHUQ)
2400 d'Estimauville, Quebec, Quebec, Canada G1E 7G9

Signature   Date

Richard Larouche, M.D.
inVentiv Health Clinique (IVH)
2500 Einstein, Quebec, Quebec, Canada G1P 0A2

Signature   Date

PROTOCOL SIGNATURE PAGE

PROTOCOL TITLE:
Immunogenicity, Safety, and Tolerability of a Plant-Derived Seasonal VLP Quadrivalent Influenza Vaccine in Elderly

PROTOCOL NUMBER: CP-Q13VLP-008
PROTOCOL DATE: June 23rd, 2014

SIGNATURES:
Protocol Author:

____________________	____________
Sonia Trépanier, Study Director, Clinical Studies	Date

Protocol Reviewed by:

____________________	____________
Michael Libman, MD, Principal Investigator	Date
____________________	____________
Guy Boivin, MD, Principal Investigator	Date
____________________	____________
Richard Larouche, MD, Principal Investigator	Date
____________________	____________
Eric Shink, Biostatistician	Date
____________________	____________
Brian J. Ward, MD, Sponsor Responsible Medical Officer	Date
____________________	____________
Isabelle-A. Vandandaigue, CRA, Study Monitor	Date

Protocol Authorized by:

____________________	____________
Nathalie Landry, Vice-president, Product Development	Date
____________________	____________
Irene Clement, Vice-president, Regulatory Affairs	Date

SYNOPSIS

**COMPANY:**	MEDICAGO R & D INC.
**INVESTIGATIONAL PRODUCT/ DRUG PRODUCT:**	PLANT-DERIVED SEASONAL VIRUS-LIKE PARTICLES (VLP) QUADRIVALENT INFLUENZA VACCINE (WILL BE REFERRED TO SEASONAL VLP INFLUENZA VACCINE)
**ACTIVE SUBSTANCE(S):**	MIX OF RECOMBINANT H1, H3, AND 2B PROTEINS [HEMAGGLUTININ (HA)] EXPRESSED AS VLPS FOR THE 4 INFLUENZA STRAINS RECOMMENDED BY WHO FOR VACCINATION IN THE NORTHERN HEMISPHERE IN 2013-2014 AS FOLLOWS:<br>• The H1 VLP Influenza vaccine contains an HA protein for which the sequence is derived from the A/California/7/2009 H1N1 virus.<br>• The H3 VLP Influenza vaccine contains an HA protein for which the sequence is derived from the wild-type A/Victoria/361/2011 H3N2 virus.<br>• The B VLP Influenza vaccine contains an HA protein for which the sequence is derived from the:<br>-B/Massachusetts/2/2012 virus (Yamagata lineage) and<br>-B/Brisbane/60/2008 virus (Victoria lineage).
**TITLE OF THE TRIAL:**	Immunogenicity, Safety, and Tolerability of a Plant-Derived Seasonal VLP Quadrivalent Influenza Vaccine (Seasonal VLP Influenza Vaccine) in Elderly
**BRIEF SUMMARY:**	This is a multiple sites (at least 3 clinical sites) phase 2, randomized, observer-blind, dose ranging, placebo-controlled clinical study to evaluate the immunogenicity, safety, and tolerability of a single intramuscular injection of plant-based Seasonal VLP Quadrivalent Influenza Vaccine administered in elderly subjects. A total of four hundred fifty (450) subjects will be randomized in six (6) groups of 75 subjects. This study will use cohort staggering (slow enrolment) for 3 non-adjuvanted dose levels (15 µg, 30 µg, and 60 µg VLP per strain), 2 adjuvanted dose levels (7.5 µg and 15 µg/strain) and a placebo-controlled group (ratio 1:1:1:1:1:1).<br>The 450 subjects (male and female subjects of 50 years of age or older) will be divided in 3 different cohorts (Refer to Sections 5.1 and 6.2.4 for details on composition of each cohort). The 7-day safety data after immunization will be collected by the clinical staff and will be reviewed by the Data and Safety Monitoring Board (DSMB). The DSMB members will determine if the clinical sites are allowed to continue immunization of the next cohort.<br>Subjects will also be divided into 2 age strata: subjects between 50 and 64 years old and subjects of 65 years or older.<br>Potential subjects may be screened:<br>• Up to 90 days prior to study immunization using an ICF under a general pre-study protocol which covers medical and medication history, vital signs, and safety laboratory tests. All other screening procedures will be performed at Day 0, once the Study ICF will be signed. Or<br>• Up to 30 days prior to immunization using the Study ICF and following all screening procedures as outlined in section 5.2.1.<br>Potential subjects will be interviewed over the phone and, if interested, invited to participate at a screening session at the clinical site (refer to Section 5.2.1 for complete details on procedures to be performed).<br>The subjects demonstrating a satisfactory baseline medical assessment as judged by the Investigator will be allowed to participate in the Day 0 procedures.

	In case the screening session is performed more than 30 days prior to subject immunization, a blood sample will be collected at Day 0, for safety analysis if deemed required by the Investigator. This extra sample will be stored. If changes in medical status, physical examination, or vital signs occurred between screening session and Day 0 visit and are judged significant by the Investigator, then, this extra sample will be tested for routine safety labs. In this case, the subject's enrolment will be postponed until laboratory results are reviewed, found acceptable by the Investigator and then the subject will be allowed to participate in Day 0 procedures. In addition, during the course of the study and based on Investigator's opinion, analysis of the safety laboratory samples collected at Day 0 may be performed to re-assess baseline. Refer to section 5.1 for more details on the screening and Day 0 procedures.<br><br>Subjects will be contacted by clinical staff on Day 1 (approximately 24 hours post-vaccination) and Day 8 to collect data on any solicited or unsolicited events.<br><br>They will also be required to return to the clinical site on Day 3 and on Day 21 for key safety (Day 3) and immunogenicity (Day 21) assessments. Blood samples for the assessment of the immune response (HI/MN) and for the isolation and analysis of PBMCs (only from 15 subjects per treatment group, at the MUHC site only) will also be collected at Day 21 visit.<br><br>Considering that the immunizations for this study will occur prior to and/or at the beginning of the seasonal influenza vaccination campaign, Medicago (via clinical sites) wants to ensure that the subjects are offered the opportunity to receive a licensed influenza (flu) vaccine. Many of the subjects that will be enrolled are among those who routinely access annual vaccination. Therefore, the sites plan to offer one of the licensed flu vaccine currently recommended for elderly, as part of this protocol. All subjects who received the licensed flu vaccine at the clinical site will be contacted approximately 7 days after this immunization for safety follow-up. They will also be asked to come to the clinical site approximately 21 days (or more) after having received this vaccine for safety and immunogenicity assessment.<br><br>Following the Day 21 visit until the time of the final visit at Day 201 (regardless if the subject received the licensed vaccine or not), monthly phone calls to each subject will be performed for safety assessment (refer to Section 5.2.5, last bullet point for details).<br><br>Subjects will be required to return to the clinical site for the final visit on Day 201 (6-months follow-up period that applies to the investigational vaccine), for safety and immunogenicity assessments.
**DEVELOPMENT PHASE:**	Phase 2
**INVESTIGATORS AND TRIAL CENTER:**	Dr. Michael Libman, M.D.<br>MUHC-Vaccine Study Centre<br>14770 Boulevard Pierrefonds, suite 204,<br>Pierrefonds, Quebec, Canada H9H 4Y6<br>Tel.: 1.514.624.7855<br><br>Dr. Guy Boivin, M.D.<br>Centre de recherche-CHU de Québec<br>2400 d'Estimauville,<br>Quebec, Quebec, Canada, G1E 7G9<br>Tel.: 1.418.525.4444<br><br>Dr. Richard Larouche, M.D.<br>inVentiv Health Clinique<br>2500 Einstein,<br>Quebec, Quebec, Canada, G1P 0A2<br>Tel.: 1.418.527.4000 ext. 7577

**EXPECTED START DATE:**	August 2014
**EXPECTED COMPLETION DATE:**	October 2014: for immunization (Day 0) and blood sampling at Day 21.<br>January 2015: report including results up to Day 21.<br>May 2015: final 6-months follow-up safety visit expected.
**PLANNED TRIAL PERIOD:**	Total planned trial period: Approximately 7-12months, from screening procedures up to Day 201 visit (depending when subjects undergo screening session and Day 201 visit).
**PRIMARY OBJECTIVE:**	The primary objective of this study is to assess the immunogenicity, safety, and tolerability of one dose of a plant-derived Seasonal VLP Quadrivalent Influenza Vaccine given non-adjuvanted at dose levels of 15 µg, 30 µg, and 60 µg/strain or adjuvanted (Alhydrogel) at dose levels of 7.5 and 15 µg/strain compared to a placebo.
**PRIMARY ENDPOINTS:**	<u>Immunogenicity</u><br>For the HI test against the homologous influenza vaccine strains:<br>Geometric mean titers (GMTs) of Hemagglutination Inhibition (HI) antibody for H1N1, H3N2, and B (B/Bris and B/Mass) vaccine strains on Days 0 and 21. Follow-up serology samples for GMTs will be collected at Day 201. GMTs will be analyzed as follows:<br>• Seroconversion factor or geometric mean fold rise (GMFR): is the geometric mean of the ratio of GMTs (Day 21/Day 0).<br>• Seroconversion rate: the proportion of subjects in a given treatment group with either a ≥4-fold increase in reciprocal HI titers between Day 0 and Day 21 or a rise of undetectable HI titer (i.e. <10) pre-vaccination (Day 0) to an HI titer of ≥40 at Day 21 post-vaccination.<br>• Seroprotection rate: the proportion of subjects in a given treatment group attaining a reciprocal HI titer of ≥40 at Day 21 post-vaccination (the percentage of vaccine recipients with a serum HI titer of at least 1:40 following vaccination).<br><u>Safety and Tolerability</u><br>• Percentage, intensity, and relationship to vaccination of immediate complaints (30 minutes post-vaccination).<br>• Percentage, intensity, and relationship to vaccination of solicited local and systemic signs and symptoms (for 7 days following study vaccine).<br>• Percentage, intensity, and relationship of unsolicited local and systemic signs and symptoms (for 21 days following study vaccine).<br>• Occurrences of all adverse events (AEs)/serious adverse events (SAEs).<br>• Occurrences of new onset of a chronic disease (NOCD).<br>• The number and percentage of subjects with normal and abnormal urine, haematological, and biochemical values at Screening, Days 3 and 201.
**STATISTICAL METHODS FOR PRIMARY ENDPOINTS:**	<u>Immunogenicity</u><br>Immunogenicity will be evaluated by the immune response induced in subjects as measured by the serum HI antibody titers at specified timepoints. Immunogenicity endpoints (including GMTs) will be compared between vaccine dose groups and also between each vaccine dose group to placebo.

**STATISTICAL METHODS FOR PRIMARY ENDPOINTS (CONT'D):**	Seroconversion and seroprotection rates will be compared using Fisher's exact tests at Day 21. The difference and the 95 % CI on the difference between the proportions of seroconverted or seroprotected subjects in each dose group compared to placebo will be calculated using Fisher's exact method. The lower bound of the 95% confidence limit for the percentage subjects achieving seroconversion or seroprotection rates within each treatment group of 75 subjects will be compared to FDA CBER's criteria for the population of ≥65 years of age. Refer to Section 2.1.1 for details on European CPMP and FDA's criteria and to section 10.3 for determination of sample size.<br><br>Results will also be presented by age strata (50-64 and 65 and older, in a ratio of 1:1 (37 and 38 subjects in each treatment group respectively). Considering the group size of each age strata per treatment group is too small to use the FDA criteria to analyze the immunogenicity endpoints, European CPMP criteria for respective age strata will be used to analyze the seroconversion factor (or GMFR), seroconversion rate and seroprotection rate obtained in each age strata, for each treatment group.<br><br><u>Safety and Tolerability</u><br><br>Safety and tolerability will be evaluated through reported AEs, history/symptom-directed physical examination findings, clinical laboratory results, and vital signs. Safety endpoints will be compared (each vaccine dose level versus placebo and also between vaccine dose groups) using Fisher's exact tests.<br>Results will also be presented by age strata (50-64 and 65 and older), in a ratio of 1:1 (37 and 38 subjects in each treatment group respectively).
**SECONDARY OBJECTIVES:**	The secondary objectives are as follow:<br>• To assess the reactivity of antibodies induced by one dose of seasonal VLP quadrivalent influenza vaccine at the three non-adjuvanted dose levels (15 µg, 30 µg, and 60 µg/strain) or at the two adjuvanted dose levels (7.5 µg and 15 µg/strain) compared to the placebo, as measured by HI antibody titers for the homologous influenza strains. This will be assessed using measures of percentage of subjects with a detectable HI antibody response (≥lower limit of quantification; ≥LOQ) at Days 0, 21, and 201.<br>• To assess the reactivity of antibodies induced by one dose of VLP quadrivalent influenza vaccine at the three non-adjuvanted dose levels (15 µg, 30 µg and 60 µg/strain) or at the two adjuvanted dose levels (7.5 µg and 15 µg/strain) compared to the placebo, as measured by microneutralisation (MN) antibody titers for the homologous influenza strains. This will be assessed using measures of GMFR, seroconversion rate, and of percentage of subjects with a detectable MN antibody response (≥LOQ) at Days 0, 21, and 201.<br>• To assess the cross-reactivity of antibodies induced by one dose of VLP quadrivalent influenza vaccine at the three non-adjuvanted dose levels (15 µg, 30 µg and 60 µg/strain) or at the two adjuvanted dose levels of (7.5 µg and 15 µg/strain) compared to the placebo, as measured by HI and MN antibody titers for heterologous influenza strains. This will be assessed using measures of GMFR, seroconversion rate, and seroprotection rate (for HI only), and of percentage of subjects with a detectable HI or MN antibody response (≥LOQ) at Days 0, 21, and 201.
**SECONDARY ENDPOINTS:**	<u>For the HI test (against the homologous influenza vaccine strains):</u><br>GMTs of HI antibody for H1N1, H3N2, and B (B/Bris and B/Mass) vaccine strains on Days 0 and 21. Follow-up serology samples for GMTs will be collected at Day 201. GMTs will be analyzed as follows:<br><br>• The percentage of subjects with a detectable HI antibody response (≥LOQ) at Days 0, 21, and 201 against the H1N1, H3N2, and B (B/Bris and B/Mass) vaccine strains.

**SECONDARY ENDPOINTS (CONT'D):**	For the HI test (against heterologous influenza strains)<br>GMTs of HI antibody for heterologous influenza strains on Days 0 and 21. Follow-up serology samples for GMTs will be collected at Day 201. GMTs will be analyzed as follows:<br>• Seroconversion factor or GMFR: is the geometric mean of the ratio of GMTs (Day 21/Day 0).<br>• Seroconversion rate: the proportion of subjects in a given treatment group with either a ≥4-fold increase in reciprocal HI titers between Day 0 and Day 21 or a rise of undetectable HI titer (i.e. <10) pre-vaccination (Day 0) to an HI titer of ≥40 at Day 21 post-vaccination.<br>• Seroprotection rate: the proportion of subjects in a given treatment group attaining a reciprocal HI titer of ≥40 at Day 21 post-vaccination (the percentage of vaccine recipients with a serum HI titer of at least 1:40 following vaccination).<br>The percentage of subjects with a detectable HI antibody response (≥LOQ) at Days 0, 21, and 201 against other influenza vaccine strains.For the MN test (against the homologous and heterologous influenza strains):<br>GMTs of MN antibody for homologous and heterologous H1N1, H3N2, and B (B/Bris and B/Mass) strains on Days 0 and 21. Follow-up serology samples for GMTs will be collected at Day 201. GMTs will be analyzed as follows:<br>• Seroconversion factor or GMFR: is the geometric mean of the ratio of GMTs (Day 21/Day 0).<br>• Seroconversion rate: the proportion of subjects in a given treatment group with either a ≥4-fold increase in reciprocal MN titers between Day 0 and Day 21 or a rise of undetectable MN titer (i.e. <10) pre-vaccination (Day 0) to an MN titer of ≥40 at Day 21 post-vaccination.<br>• The percentage of subjects with a detectable MN antibody response (≥LOQ) at Days 0, 21, and 201.
**STATISTICAL METHODS FOR SECONDARY ENDPOINTS:**	Seroconversion factor or GMFR will be compared between active dose groups and also between each active dose group to placebo using analysis of variance (ANOVA) methods. A multi-stage test (i.e., Dunnett's test and Tukey's correction) will be used to evaluate differences among the treatment groups.<br>Seroconversion rates and seroprotection rates (for HI only) will be compared (i.e., each vaccine dose level versus placebo and also between active dose groups) using Fisher's exact tests at Day 21. The difference and the 95 % CI on the difference between the proportions of seroconverted or seroprotected subjects in each dose group compared to placebo will be calculated using Fisher's exact method.<br>Results will also be presented by age strata (50-64 and 65 and older, in a ratio of 1:1 (37 and 38 subjects in each treatment group respectively).
**EXPLORATORY ENDPOINTS:**	Cell-mediated immune (CMI) response in PBMCs<br>• To characterize the T cell response in PBMC (in 15 subjects per treatment group, for a total of 90 subjects) induced by vaccination (Days 0, 21, and 201). Refer to section 2.3.1 for details on CMI analysis.<br>Humoral response<br>• To measure the increase in sera antibodies directed to plant-specific glycans (timeframe: Days 0, 21, and 201).<br>• Humoral response induced approximately 21 days after administration of the licensed flu vaccine (for those who receive it at the clinical site) will also be evaluated.

**STATISTICAL METHODS FOR EXPLORATORY ENDPOINTS**	When applicable for Exploratory endpoints and immunogenicity endpoints will be compared (each vaccine dose level versus placebo and between each vaccine group) using analysis of variance methods for continuous endpoints and Fishers' exact tests or chi-square tests for categorical endpoints. Non-parametric methods will be utilized if the continuous data does not meet normality assumptions.<br><br>Results will also be presented by age strata (50-64 and 65 and older, in a ratio of 1:1 (37 and 38 subjects in each treatment group respectively).
**MODE OF ADMINISTRATION AND DURATION OF TREATMENT:**	One intra-muscular (IM) injection on Day 0 of the adjuvanted or non adjuvanted Seasonal VLP Quadrivalent Vaccine or placebo into the deltoid muscle of the non-dominant arm (preferably). The volume of injection is 0.5 mL for all dose levels with the exception of the 60 µg per strain (240 µg total) for which the volume of injection will be 1.0 mL.
**INVESTIGATIONAL PRODUCT, DOSE, LOT NUMBERS:**	Seasonal VLP Quadrivalent Influenza vaccine, a plant-derived candidate Influenza Vaccine administered by IM injection of 15 µg, 30 µg, or 60 µg, of each strain without adjuvant or of 7.5 µg or 15 µg of each strain with Alhydrogel® as adjuvant.<br><br>The four influenza recombinant HA proteins of the VLP quadrivalent vaccine are based on the following human influenza strains:<br>• A/California/7/2009 (H1N1)<br>• A/Victoria/361/2011 (H3N2)<br>• B/Brisbane/60/2008 (B/Bris)<br>• B/Massachusetts/2/2012 (B/Mass)<br><br>The name in parenthesis will be used in the protocol to simplify the text.<br>The Seasonal VLP Influenza Vaccine lot numbers are defined below:<br>Vaccine at concentration of 30 µg/mL:<br>- Lot number: AAHAW01-140016-012<br>Vaccine at concentration of 60 µg/mL:<br>- Lot number: AAHAW01-140015-011
**OTHER PRODUCTS USED IN THIS STUDY (ADJUVANT):**	The Adjuvant (Alhydrogel®) lot number is defined below:<br>- Lot number: AAIXX01-130012-013
**CONTROL PRODUCT (PLACEBO):**	Phosphate buffered saline (PBS; Placebo):100 mM phosphate buffer + 150 mM sodium chloride + 0.01% Tween 80.<br>- Lot number: PPPXX01-130005-014
**NUMBER OF SUBJECTS AND TREATMENT GROUPS**	Total planned subjects: 450 subjects randomized into 6 groups in a 1:1:1:1:1:1 ratio:<br>Group 1: 75 subjects administered (IM) 15 µg per strain of the Seasonal VLP vaccine<br>Group 2: 75 subjects administered (IM) 30 µg per strain of the Seasonal VLP vaccine<br>Group 3: 75 subjects administered (IM) 60 µg per strain of the Seasonal VLP Vaccine<br>Group 4: 75 subjects administered (IM) 7.5 µg per strain of the Seasonal VLP vaccine mixed with Alhydrogel® (0.5 mg Al per dose)<br>Group 5: 75 subjects administered (IM) 15 µg per strain of the Seasonal VLP vaccine mixed with Alhydrogel® (0.5 mg Al per dose)<br>Group 6: 75 subjects administered (IM) placebo (PBS)

**SAFETY ASSESSMENTS**	Clinical safety assessments will include vital signs (including oral temperature), safety clinical laboratory testing (blood chemistry and haematology testing and urinalysis), and history/symptom-directed physical examinations.<br><br>In addition, a phone contact/memory aid (e.g. diary card) follow-up to collect solicited local and systemic symptoms and unsolicited AEs, SAEs, visits to healthcare providers, hospitalizations and any new medications taken or changes in concomitant medication will be performed at Days 1 and 8 following vaccination as well as ~7 days following administration of the offered licensed flu vaccine at Day ≥21 visit. Also, approximately 21 days after administration of the offered licensed flu vaccine, a safety assessment will be performed. Between Day 21 and Day 201 visits, any AEs/SAEs experienced during the study will be recorded on subjects' memory aid.
**INCLUSION CRITERIA:**	1) Male and female subjects, 50 years of age or older.<br>2) BMI of ≥18 and ≤32.<br>3) Give his/her consent to participate in this study (by signing the Study ICF). In the opinion of the Investigator, competence and willingness to provide written, informed consent for participation after reading the informed consent form. The subject must have adequate opportunity to discuss the study with an Investigator or qualified designee.<br>4) Healthy as judged by the Investigator or designee and determined by general physical examination, vital signs, clinical laboratory tests, and medical history conducted no more than 90 days prior to study vaccine administration. Subjects with a pre-existing chronic disease will be allowed to participate if the disease is stable and, according to the Investigator's judgment, the condition is unlikely confound the results of the study or pose additional risk to the subject by participating in the study. Stable disease is defined as no new onset of exacerbation of pre-existing chronic disease 6 months prior to immunization. Based on Investigator's judgment, a subject with more recent stabilisation of a disease may still be eligible.<br>5) Comprehension of the study requirements, expressed availability for the required study period, ability to attend scheduled visits, accessible by phone on a consistent basis.<br>6) If female, have a negative serum pregnancy test result at screening and negative urine pregnancy test on Day 0 prior to immunization.<br>7) Female of childbearing potential must use an effective method of contraception for 1 month prior to immunization and agrees to continue employing adequate birth control measures for at least 60 days post-immunization. Moreover, she must have no plan to become pregnant for at least 2 months post-immunization. Abstinent subjects should be asked what method(s) they would use, should their circumstances change, and subjects without a well-defined plan should be excluded.<br>The following relationship or methods of contraception are considered to be effectives:<br>• Hormonal contraceptives (e.g., injectable, topical [patch], estrogenic vaginal ring, etc.);<br>• Intra-uterine device (IUD) with or without hormonal release;<br>• Male partner using a condom plus spermicide or sterilized partner (at least 1 year prior to immunization);<br>• History of credible abstinence (self-reported);<br>• Heterosexual abstinence at least 60 days post-immunization;<br>• Female partner.

**INCLUSION CRITERIA (CONT'D):**	8) Non-childbearing females defines as:<br>• Surgically-sterile (defined as bilateral tubal ligation or hysterectomy performed more than 1 month prior to immunization);<br>• Post-menopausal (absence of menses for 24 consecutive months and age consistent with natural cessation of ovulation).
**EXCLUSION CRITERIA:**	1) According to Investigator's opinion, presence of significant acute or chronic, uncontrolled medical or neuropsychiatric illness. "Uncontrolled" is defined as:<br>a) Requiring a new medical or surgical treatment within one month prior to study vaccine administration which would interfere with vaccine evaluation or study completion (subject requiring medical or surgical treatment that would not interfere with evaluation [e.g., minor knee surgery, cataracts, etc.] would be eligible);<br>b) Requiring a change in medication dosage in one month prior to study vaccine administration due to uncontrolled symptoms or drug toxicity (elective dosage adjustments in stable subjects are acceptable).<br>2) Any medical or neuropsychiatric condition or any history of excessive alcohol use or drug abuse which, in the Investigator's opinion, would render the subject unable to provide informed consent or unable to provide valid safety observations and reporting.<br>3) Any autoimmune disease or any confirmed or suspected immunosuppressive condition or immunodeficiency including history of human immunodeficiency virus (HIV) infection, Hepatitis B or C, or the presence of lymphoproliferative disease.<br>4) Administration of any vaccine (including any other influenza vaccine) within 30 days prior to study enrolment or planned administration within the period from the vaccination up to blood sampling at Day 21 or within 30 days prior to blood sampling at Day 201. Immunization on an emergency basis will be evaluated in a case-by-case by the Investigator.<br>5) Administration of any adjuvanted or investigational influenza vaccine within 1 year prior to study enrolment or planned administration prior to the end of this trial (Day 201). Administration of any 'standard', not adjuvanted influenza vaccine (e.g., live attenuated TIV/QIV vaccine IN or split TIV/QIV vaccine by either intra-dermal or IM route) prior to the 30-day exclusion period mentioned above would be acceptable.<br>6) Use of any investigational or non-registered product within 30 days prior to study enrolment or planned use during the study period. Subjects may not participate in any other investigational or marketed drug study while participating in this study (approximately 7-12 months [depending when subjects undergo screening session and Day 201 visit]).<br>7) Treatment with systemic glucocorticoids at a dose exceeding 10 mg of prednisone per day, or equivalent for more than 7 consecutive days or for 10 or more days in total, within one month of study vaccine administration, any other cytotoxic or immunosuppressant drug, or any immunoglobulin preparation within 3 months of vaccination. Routine use of standard doses of nasal or inhaled glucocorticoids is allowed.<br>8) Any significant disorder of coagulation or treatment with warfarin derivatives or heparin. Persons receiving prophylactic anti-platelet medications, e.g., low-dose aspirin (no more than 325 mg/day), and without a clinically apparent bleeding tendency are eligible. Subjects treated with new generation drugs that will not increase risk of intramuscular bleeding (such as clopidogrel) are also eligible.

**EXCLUSION CRITERIA (CONT'D):**	9) History of allergy to any of the constituents of the VLP quadrivalent (including H1N1, H3N2, B/Bris and B/Mass) study vaccine, Alhydrogel® (aluminum hydroxide) or to the PBS (used as placebo), or tobacco allergy.<br>10) History of anaphylaxis reaction to any food, medication or bee sting.<br>11) Any history of severe asthma (e.g., status asthmaticus, hospitalization for asthma control) or recurrent asthma episodes requiring medical attention in the last 3 years (≥ 1 episode/year).<br>12) Continuous use of anti-histamines in the last 4 weeks prior to immunization or use of anti-histamines 48 hours prior to study immunization.<br>13) Have a rash, dermatological condition, tattoos, muscle mass, or any other abnormalities at injection site which may interfere with injection site reaction rating.<br>14) Have received a blood transfusion within 90 days prior to study vaccination.<br>15) If female, have a positive or doubtful pregnancy test prior to immunization or lactating females.<br>16) Vital sign abnormalities: systolic blood pressure ≥150 mmHg and/or diastolic blood pressure ≥90mmHg, heart rate ≤40 beats/min and ≥100 beats/min. Although a vital signs measurement is out of the acceptable ranges, a subject may be included in the study based on Investigator's judgment. Presence of any febrile illness (including oral temperature (OT) ≥38.0˚C within 24 hours prior to immunization). Such subjects may be re-evaluated for enrolment after resolution of illness.<br>17) Cancer or treatment for cancer within 3 years of study vaccine administration. Persons with a history of cancer who are disease-free without treatment for 3 years or more are eligible. Persons with treated and uncomplicated basal cell carcinoma of the skin are eligible. Person with non-treated, non-disseminated local prostate cancer are eligible.<br>18) Identified as an Investigator or employee of the Investigator or study center with direct involvement in the proposed study, or identified as an immediate family member (i.e., parent, spouse, natural or adopted child) of the Investigator or employee with direct involvement in the proposed study.

Table 1: Study visit procedures

Day of visit	Screen Visit	Day 0	Day 1	Day 3	Day 8	Day 21	Day ≥28[13] (~7d after licensed vaccine shot)	Day≥42[13] (≥21d after licensed vaccine shot)	Monthly calls[11]	Day 201[14]
**Visit type**	Enrol.	Vaccine/ Immuno	☎ Safety	Collect Safety	☎ Safety	Safety/ Immuno/Flu shot[14]	☎ Safety (Flu shot)	Safety/ Immuno/Flu shot	☎ Safety	Final Safety/ Immuno
**Visit allowed time window**	Day -90 to Day -0		+1 day	-1/+2 days	-1/+2 days	- 2/+3 days	±4 days	+35 days	±7 days	- 14/+80 days
**Informed consent (ICF)**	X	X[15]								
**Inclusion/Exclusion Criteria and Eligibility**	X	X								
**Eligibility assessment**		X		X		X				X
**Demographics**	X									
**Physical Examination History/symptom-directed Physical Examination**[1]	X	X		X		X		X		X
**Lymph node examination**[1]	X	X		X		X		X		X
**Medical & medication history (within 3 M prior to vaccination) & check for changes at each following visit or call**	X	X[9]	X	X	X	X	X	X	X	X
**Body measurements (Height, weight, BMI)**[2]	X	X								
**Vital Signs (BP, HR, RR, OT)**[3]	X	X				X		X		X
**Clinical Laboratory Samples (Chemistry, Haematology, and Urinalysis)**	X	X[12]		X						X
**Serology (HIV, Hepatitis B and C)**	X									
**Pregnancy test**[4]	X	X				X				
**Randomization**		X								
**Serology for HI, MN titers**[5]		X				X		X		X
**Cellular Immune Response (for PBMC preparation)**[6]		X				X				X
**Vaccine administration**		X								
**Safety Observation (for at least 30-minutes post-vaccination)**		X								
**Memory Aid (MA or diary card) Instructions**[7]		X				X				
**Collection of Solicited and systemic signs and symptoms**[8]		X	X		X					
**Occurrence of AEs/SAEs, NOCDs/Meds**		X[10]	X	X	X	X	X	X	X	X
**Study conclusion**										X

1 Physical examination (PE) to be done at Screening visit and history/symptom-directed PEs at Day 0, 3, 21, and 201 (PE will not be routinely performed, unless deemed to be necessary by the Investigator). If subject complains of arm and/or shoulder pain, a direct examination of the lymph node for swelling (neck and axilla) will be done.
2 During Day 0 visit, only weight will be measured while the height will be obtained from that measured at screening visit.
3 Blood pressure (BP), heart rate (HR), respiratory rate (RR; not done at screening), and oral temperature (OT) will be measured at screening, prior and after immunization on Day 0, and at Days 21, ≥43, and 201.
4 To be performed for all females; at screening it will be done in sera and at Day 0 (before immunization) and Day 21 in urine.
5 Blood sampling will be performed prior to immunization.
6 Blood sampling for CMI response analysis (PBMC) in 15 subjects per group, for a total of 90 subjects (applied to MUHC site, only).
7 This includes training on measurement device and use of the thermometer at Day 0, instructions for OT and solicited and systemic signs and symptoms collection from Day 0 (evening) up to Day 7 (evening). Emergency information and instructions will also be given. A MA will be given on Day 0 and Day 21 (to cover Day 0 to Day 21 and Day 21 to Day 201 periods, respectively). Depending on site's procedures, subjects might be asked to bring their MA with them to subsequent visits and/or for phone contacts.
8 During the phone contact, subjects will be reminded to record any information on their MA (including their next visit and/or phone contact).
9 Record any changes in medical history and medication intakes since screening and confirm subjects' eligibility.
10 Record the occurrence of any AEs/SAEs prior and post-immunization (during the 30-minute observation period).
11 Subjects should be reached once a month with no more than 45 days between phone contacts (use Day 21 date as starting reference).
12 Depending on screening strategy, blood sample for safety lab tests will be withdrawn and stored. Sample will be tested only if needed based on Investigator's opinion.
13 Licensed flu vaccine may be administered after Day 21. This vaccine would be available at the clinical sites until mid-December. Dates for the subsequent safety call and safety/immuno visit (respectively 7 days and~21 days (or more, up to 56 days)) after the licensed flu vaccine shot) will be adjusted accordingly.
14 Unscheduled visits could occur during the study (based on Investigator's request or for repeat measurements purpose).
15 Signature of the Study ICF may occur at Day 0 depending on the screening strategy (refer to section 5.2.1).

LIST OF ABBREVIATIONS

AE	Adverse Event
ALT	Alaninetransferase
ANOVA	Analysis of variance
AST/SGOT	Aspartatetransferas
BMI	Body Mass Index
BP	Blood Pressure
CBER	Center for Biologics Evaluation and Research
CCD	Cross-reactive carbohydrate determinant
CI	Confidence Interval
CMI	Cell-mediated immune response
CPMP	Committee for Proprietary Medicinal Products
CR-CHUQ	Centre de recherche - CHU de Québec
CRF	Case Report Form
CRO	Clinical Research Organization
DNA	Deoxyribonucleic acid
DSMB	Data Safety Monitoring Board
FAS	Full Analysis Set
FDA	Food and Drug Administration
GCP	Good Clinical Practice
GGT	Gamma glutamyltransferase
GLP	Good Laboratory Practice
GMFR	Geometric Mean Fold Rise
GMP	Good Manufacturing Practice
GMT	Geometric Mean Titre
H/HA	Hemagglutinin
HI	Hemagglutination Inhibition
HIV	Human immunodeficiency virus
HR	Heart Rate
IB	Investigational brochure
ICF	Informed Consent Form
ICH	International Conference on Harmonization
IgE	Immunoglobulin E
IND	Investigational New Drug
IM	Intramuscular
IRB	Investigational Review Board
IVH	inVentiv Health Clinique

LOQ	Limit of Quantification
MA	Memory Aid
MCH	Mean cell haemoglobin
MCHC	Mean cell haemoglobin concentration
MCV	Mean cell volume
MedDRA	Medical Dictionary for Drug Regulatory Affairs
MN	Microneutralization
MUHC	McGill University Health Center
N/NA	Neuraminidase
NOCD	New onset of a chronic disease
OT	Oral temperature
OTC	Over-the-Counter
PBMCs	Peripheral Blood Mononuclear Cells
PBS	Phosphate buffered saline
PI	Principal Investigator
PP	Per-Protocol
QC	Quality control
QA	Quality assurance
RNA	Ribonucleic acid
RR	Respiratory Rate
SAE	Serious Adverse Event
SAP	Statistical Analysis Plan
SAS®	Statistical Analysis System®
URTI	Upper Respiratory Tract Infection
VLP	Virus-like particle
WBC	White blood cells
WHO	World Health Organization

1. INTRODUCTION

1.1. Background

Seasonal influenza is a potentially serious infection associated with a wide range of clinical outcomes across the entire human lifespan. The influenza virus genome is an 8-segment single-stranded RNA with high potential for in situ recombination and mutations for host adaptation. Two segments code for the hemagglutinin (H) and neuraminidase (N) antigens that play an essential role for virus infectivity. At present, 17 H and 9 N subtypes are known in bird populations and some subtypes routinely circulate in humans such as the H1N1, H3N2 and B influenza viruses. When a new subtype acquires the capacity for human-to-human transmission, this can give rise to a major pandemic like the one caused by an H1N1 strain in 1918 referred to as the Spanish flu. Antibodies binding to the HA molecule can prevent or modify infection and are the basis on which influenza vaccines are formulated every year.

The main strategy for prevention and control of seasonal and pandemic influenza is still vaccination. Since 1960, annual influenza vaccination has been recommended in the USA for individuals with chronic disease, those aged 65 years or older and (more recently) pregnant women. In 2012, the Advisory Committee on Immunization Practices issued the first recommendation of national universal seasonal influenza vaccination[1].

Despite continuing advances in understanding of the immune response to natural infection and immunization, the disease remains a major cause of morbidity and mortality worldwide. In the USA alone, influenza is responsible for approximately 36,000 deaths per year and the World Health Organization estimates that influenza causes 3-5 million cases of severe illness and 250,000- 500,000 deaths per year[2]. Influenza is also responsible for a massive economic burden, with a total cost for each winter influenza season evaluated at over 87 billion dollars in the United States alone[3].

Licensure of influenza vaccines is based either on efficacy studies or on surrogate correlates of protection. The latter are primarily based on their ability to induce hemagglutination-inhibition (HI) titers. The current criteria used by the FDA for accelerated approval (using surrogate endpoints) of an influenza vaccine are the following:

For adults <65 years of age and for the pediatric population:

- The lower bound of the two-sided 95% CI for the percent of subjects achieving seroconversion for HI antibody should meet or exceed 40%.
- The lower bound of the two-sided 95% CI for the percent of subjects achieving an HI antibody titer > 1:40 should meet or exceed 70%.

For adults ≥65 years of age:

- The lower bound of the two-sided 95% CI for the percent of subjects achieving seroconversion for HI antibody should meet or exceed 30%.
- The lower bound of the two-sided 95% CI for the percent of subjects achieving an HI antibody titer >1:40 should meet or exceed 60%.

Despite the existence of these surrogate correlates of protection, recent reviews of vaccine efficacy studies suggest that the overall efficacy of licensed trivalent vaccines is highly variable and depends, among other factors, on the 'match' between circulating strains and the strains

used for vaccine production. One such analysis produced efficacy estimates from 43-89% in well-matched seasons and from 38-85% in poorly matched seasons (<25% of isolates matched the vaccine strains)[4], suggesting that there is still great room for improvement.

For the 2012-2013 season, vaccine efficacy numbers are particularly discouraging. When broken down by age group, the vaccine overall efficacy against influenza A and B viruses ranged from 27% in people 65 and older to 64% in children (aged 6 months to 17 years old). When specific strains and age groups were examined (e.g., H3N2 in the elderly), efficacy estimates could be as low as 9% (95% CI: -84% to 55%)[5].

Medicago's plant-derived VLP Quadrivalent Influenza vaccines may be able to address several of the limitations of currently licensed vaccines. First, the HA proteins in each monovalent VLP is based on the genetic sequence of circulating human influenza viruses selected by the WHO. In contrast, influenza strains grown in embryonic eggs or tissue culture tend to mutate for optimal growth in these substrates. Some of these mutations can occur in the critical HA protein and may even involve changes in cellular receptor tropism (e.g., adaptation to avian 2-3α sialic acid receptor rather than the human 2-6α sialic acid receptors). The extent of mutations in the HA protein is strain dependent and can vary widely from year to year. For example, the H1N1 virus strain currently used by egg-based vaccine manufacturers (reassortant X-179A H1N1 strain) contains 4 amino acid mutations in the globular domain of the HA protein. Similarly, when grown in eggs, most influenza B strains contain at least one amino acid mutation that affects HA glycosylation compared to human isolates[6]. In some cases, these mutations change the affinity of antibodies directed against human strains. Although such cross-species selection pressure can theoretically be avoided with the use of mammalian cell culture instead of embryonic eggs (e.g., Novartis' newly-licensed TIV - Flucelvax™), unpredictable mutations can arise with living virus in any artificial culture system. Furthermore, any living virus needs to be inactivated by chemicals and then split by a detergent before injection into humans, processes that can also influence antigenicity.

There are no mutations in the HA molecule produced with the plant-based manufacturing system since this platform does not rely on the growth of living viruses. Medicago's technology is based on the production of recombinant HA proteins that self-assemble into Virus-Like Particles (VLPs). These nanoparticules mimic the shape of the influenza virus and are efficiently recognized by the immune system that has evolved to recognize pathogens of such dimension.

Medicago has accumulated considerable pre-clinical and clinical data showing the extent of the immune response induced by such VLP vaccines. First, preclinical studies show that 1-2 doses of H5 or H7 VLP vaccine can protect animals from disease and death despite low to undetectable HI antibodies at some doses. Challenge studies in both ferret and mouse models further demonstrate that these VLP vaccines can even provide some degree of heterosubtypic protection. For example, mice immunized with a single dose of an H5 VLP vaccine are largely protected from lethal H2N2 challenge. These results strongly suggest that protection against influenza can be mediated by immune effectors other than HI antibodies and that plant-derived VLP vaccine have the potential to induce cross-reactive protection.

Medicago's clinical trial program has revealed that plant-derived H5 and H1 VLP vaccines also induce long-lasting and cross-reactive T cell responses which could be pivotal for protection against both matched and diverse influenza strains. After almost half a century of focusing on humoral antibody responses to influenza, there is increasing interest in revisiting the role of

T cell immunity after both influenza infection and vaccination[7,8]. In particular, T cell memory may be important for understanding natural infection and vaccine-induced immunity in the elderly who often derive significant benefit from vaccination despite little evidence of a humoral response[9]. Recent evidence suggests that cross-reactive T cells elicited by infection and vaccination contribute to cross-strain protection in both animal models and humans. These observations have led to the introduction of adjuvants that can boost the humoral and cell-mediated responses induced by traditional egg-derived split vaccines[10].

Our results to date suggest that plant-derived VLP vaccines induce strong T cell responses even in the absence of adjuvants. Although the primary end-points of the proposed phase 2 clinical trial of Medicago's seasonal VLP Quadrivalent vaccine will be safety and the induction of HI antibodies, secondary and exploratory end-points will include more detailed antibody responses (eg: microneutralization (MN) and antibodies against conserved epitopes of the stem region) as well as T cell responses. The latter will be measured using PBMCs collected from 15 subjects per group at Days 0, 21, and 201 (MUHC site only). T cell cross-reactivity will be evaluated by stimulating the PBMCs with various VLPs and peptide pools.

The data collected in these studies will help define the quality of the immune response induced by the plant-derived vaccine, establish potential competitive advantages and support the design of subsequent clinical trials.

1.2. Background of the Investigational Product

Please refer to Section 2.2 and 3 of the Investigator Brochure.

1.3. Clinical Studies - Results from Phase 1-2 trial in healthy adults 18-49 years of age

In 2013, Medicago completed a phase 1-2 study in the US (IND 15716) for its quadrivalent VLP influenza vaccine candidate. The current quadrivalent VLP vaccine is produced by a similar process, thus the results from the phase 1-2 study provide supportive safety and immunogenicity data for the quadrivalent formulation. In addition, potential reactions to plant glycans were investigated in this trial and the results are presented in 1.3.3.

1.3.1 SAFETY

Overall results from the phase 1-2 clinical study showed that the VLP Quadrivalent Influenza vaccine was well tolerated with an incidence of solicited signs and symptoms and an adverse event (AE) profile that did not represent a notably greater safety risk than that of the placebo (see Table 2). A few cases of fever over 38ºC have been reported and local and systemic reactions were mostly mild and of short duration. Neither SAEs nor allergic reactions were reported up to Day 21. No safety issues were noted with respect to physiological measurements. The 6 month safety follow up data will be available in July 2014.

Table 2: Incidence of systemic and local reactions within 7 days after the vaccination[1]

GROUP	PLACEBO	3 µg/strain VLP QUADRIVALENT VACCINE	9 µg/strain VLP QUADRIVALENT VACCINE	15µg/strain VLP QUADRIVALENT VACCINE
	N=30	N=30	N=30	N=30
SUBJECTS WITH AT LEAST ONE SOLICITED LOCAL OR SYSTEMIC REACTIONS				
	11(36.7)	14(46.7)	14(46.7)	18(60.0)
SYSTEMIC				
Fever	1(3.3)	0	2(6.7)	1(3.3)
Fatigue	5(16.7)	1(3.3)	4(13.3)	5(16.7)
Headaches	7(23.3)	4(13.3)	4(13.3)	7(23.3)
Muscle aches	3(10.0)	0	4(13.3)	5(16.7)
Feelings of general discomfort or uneasiness	4(13.3)	1(3.3)	3(10.0)	4(13.3)
Joint aches	2(6.7)	0	0	4(13.3)
Chills	1(3.3)	1(3.3)	2(6.7)	2(6.7)
Swelling in the Axilla	0	0	1(3.3)	0
Swelling in the Neck	1(3.3)	0	0	1(3.3)
Swelling in the Groin	0	0	0	0
LOCAL				
Redness	0	0	0	0
Swelling	2(6.7)	0	2(6.7)	2(6.7)
Pain	4(13.3)	11(36.7)	11(36.7)	15(50.0)

[1] Data are number of subjects reporting adverse reactions within 7 days of vaccination. Percentages are based on number of patients in the study population.

1.3.2 ANTIBODY RESPONSE

The antibody response against the four strains included in the vaccines is shown below. Table 3 shows the HI antibody results and Table 4 shows the serum neutralizing antibody results.

Briefly, 21 days after vaccination all tested dosages induced a detectable antibody response against the 4 strains that is statistically different from the placebo group. CHMP criteria were met at all tested dose for the A influenza strains and with dosages of 9 and 15 micrograms. There was no statistical difference in GMT, seroconversion and seroprotection rates between the 3, 9 or 15 microgram doses for the H1N1 and H3N2 influenza strains. However, for the B/Brisbane strain (Victoria lineage), the 15 microgram dose induced a significant higher antibody response compared to the 3 microgram dose (p=0.0188) and a higher seroconversion rate (p=0.0083). For the B\Wisconsin strain, the 15 microgram dose induced a higher antibody level and seroprotection rates than the 3 microgram dose (p values of 0.0394 and 0.0257). For the B\Wisconsin strain, the seroprotection rate of the 9 microgram dose was lower than the 15 microgram dose (p value of 0.0271). Based on these results, the next trial will evaluate dosages of 15, 30 and 60 micrograms of the VLP Quadrivalent vaccine. The vaccine will be evaluated in groups of 75 subjects per arm in order to evaluate immunogenicity according to CBER's criteria. At time of issue of this IB, cross-reactive HI antibody results were not available.

Table 3: Serum Hemagglutination-Inhibition Antibody Titers (Full set analysis)

Strain	A/California/07/2009, A/Victoria/361/2011, B/Wisconsin/1/2010 (Yamagata lineage), B/Brisbane/60/2008 (Victoria lineage)			
**Dose**	3µg/Strain (Total HA: 12µg)	9µg/Strain (Total HA: 36µg)	15µg/Strain (Total HA: 60µg)	Placebo
**N**	29	30	27	30
**Age (years)**	18-49 years	18-49 years	18-49 years	18-49 years
**Reference**	Results from Clinical Trial : CP-Q12VLP-004			
**H1N1**	**A/California/07/2009 WT**			
**GMT D21**	72.7 (44.4-119.0)	68.5 (40.7-115.0)	102.0 (57.7-180.4)	14.8 (9.5-23.0)
**Seroprotection D0**	34.5% (17.9-54.3)	30.0% (14.7-49.4)	48.1% (28.7-68.1)	26.7% (12.3-45.9)
**Seroprotection D21**	79.3% (60.3-92.0)	73.3% (54.1-87.7)	81.5% (61.9-93.7)	33.3% (17.3-52.8)
**Seroconversion D21**	41.4% (23.5-61.1)	50.0% (31.3-68.7)	40.7% (22.4-61.2)	0.0% (0.0-11.6)
**GMI (D21/D0)**	3.7 (2.6-5.2)	4.3 (3.1-6.0)	4.1 (2.9-5.8)	1.0 (0.7-1.4)
**H3N2**	**A/Texas/50/2012 (NIBSC #13/162)**			
**GMT D21**	91.8 (54.5-154.7)	129.9 (82.8-203.9)	128.7 (70.2-236.0)	14.5 (9.5-22.0)
**Seroprotection D0**	27.6% (12.7-47.2)	33.3% (17.3-52.8)	48.1% (28.7-68.1)	23.3% (9.9-42.3)
**Seroprotection D21**	79.3% (60.3-92.0)	90.0% (73.5-97.9)	81.5% (61.9-93.7)	23.3% (9.9-42.3)
**Seroconversion D21**	48.3% (29.4-67.5)	60.0% (40.6-77.3)	44.4% (25.5-64.7)	0.0% (0.0-11.6)
**GMI (D21/D0)**	4.8 (3.2-7.3)	7.4 (4.9-11.0)	5.8 (3.8-8.8)	0.9 (0.6-1.4)
**B (Victoria lineage)**	**B/Brisbane/60/2008 (NYMC BX-35) (NIBSC#10/106)**			
**GMT D21**	38.6 (25.4-58.6)	49.8 (32.6-76.1)	82.1 (50.4-133.6)	19.7 (12.1-32.1)
**Seroprotection D0**	34.5% (17.9-54.3)	16.7% (5.6-34.7)	40.7% (22.4-61.2)	30.0% (14.7-49.4)
**Seroprotection D21**	65.5% (45.7-82.1)	70.0% (50.6-85.3)	85.2% (66.3-95.8)	30.0% (14.7-49.4)
**Seroconversion D21**	13.8% (3.9-31.7)	43.3% (25.5-62.6)	48.1% (28.7-68.1)	0.0% (0.0-11.6)
**GMI (D21/D0)**	2.0 (1.5-2.7)	3.5 (2.6-4.7)	4.0 (3.0-5.4)	1.1 (0.8-1.5)
**B (Yamagata lineage)**	**B/Wisconsin/1/2010 (NIBSC #12/110)**			
**GMT D21**	64.5 (36.8-113.0)	74.5 (48.4-114.7)	136.3 (86.8-214.2)	22.4 (13.9-36.0)
**Seroprotection D0**	48.3% (29.5-67.5)	20.0% (7.7-38.6)	48.1% (28.7-68.1)	36.7% (19.9-56.1)
**Seroprotection D21**	72.4% (52.8-87.3)	73.3% (54.1-87.7)	96.3% (81.0-99.9)	43.3% (25.5-62.6)
**Seroconversion D21**	24.1% (10.3-43.5)	53.3% (34.3-71.7)	51.9% (31.9-71.3)	10.0% (2.1-26.5)
**GMI (D21/D0)**	2.6 (1.9-3.7)	5.2 (3.7-7.3)	5.5 (3.9-7.9)	1.2 (0.9-1.7)

The results shown in Table 4 confirm the trends seen with HI antibody results. In general seroconversion rates with the neutralizing antibody test are slightly higher for A influenza strains and comparable for B influenza strains. For this assay, one cross-reactivity result is available. Interestingly the VLP Quadrivalent vaccine induced 33.3% seroconversion rate against the newly selected B-Yamagata strain (B\Massachussets/2/2012) for the 2014-2015. These results suggest some level of cross-protection against heterosubtypic influenza strains.

Table 4: Serum Neutralizing Antibody Titers (non-audited data)

**Strain**	A/California/07/2009, A/Victoria/361/2011, B/Wisconsin/1/2010 (Yamagata lineage), B/Brisbane/60/2008 (Victoria lineage)			
**Dose**	3µg/Strain (Total HA: 12µg)	9µg/Strain (Total HA: 36µg)	15µg/Strain (Total HA: 60µg)	Placebo
**N**	29	30	27	30
**Age (years)**	18-49 years	18-49 years	18-49 years	18-49 years
**Reference**	Results from Clinical Trial : CP-Q12VLP-004			
**MN titers H1N1**	**A/California/07/2009**			
**GMT D0**	23.9 (13.2-43.2)	23.8 (14.4-39.2)	31.7 (15.9-63.1)	16.2 (9.9-26.7)
**GMT D21**	93.4 (53.7-162.5)	103.1 (56.4-188.6)	153.9 (86.4-274.1)	16.2 (9.9-26.5)
**Seroconversion D21**	44.8% (26.5-64.3)	46.7% (28.3-65.7)	55.6% (35.3-74.5)	0.0% (0.0-11.6)
**GMI (D21/D0)**	3.9 (2.3-6.6)	4.3 (2.7-6.9)	4.9 (2.8-8.3)	1.0 (0.9-1.1)
**MN titers H3N2**	**A/Texas/50/212**			
**GMT D0**	73.6 (42.4-127.7)	66.5 (39.6-111.6)	103.4 (59.1-181.0)	59.2 (36.4-96.5)
**GMT D21**	436.4 (286.3-665.7)	544.4 (370.8-799.4)	570.2 (395.9-821.1)	62.0 (37.9-101.5)
**Seroconversion D21**	48.3% (29.5-67.5)	70.0% (50.6-85.3)	63.0% (42.4-80.6)	0.0% (0.0-11.6)
**GMI (D21/D0)**	5.9 (3.4-10.3)	8.2 (5.1-13.1)	5.5 (3.5-8.8)	1.0 (1.0-1.2)
**MN titers B (Victoria lineage)**	**B/Brisbane/60/2008**			
**GMT D0**	12.5 (8.8-17.8)	10.8 (7.3-16.0)	15.3 (10.2-22.9)	13 (8.2-20.7)
**GMT D21**	24.5 (16.1-37.2)	31.0 (20.4-47.0)	57.3 (35.2-93.2)	13.0 (8.1-20.9)
**Seroconversion D21**	20.7% (8.0-39.7)	26.7% (12.3-45.9)	40.7% (22.4-61.2)	0.0% (0.0-11.6)
**GMI (D21/D0)**	2.0 (1.4-2.7)	2.9 (2.1-3.9)	3.8 (2.3-6.0)	1.0 (0.9-1.1)
**MN titers B (Yamagata lineage)**	**B/Wisconsin/1/2010**			
**GMT D0**	27.3 (17.0-43.9)	17.6 (10.9-28.4)	29.4 (18.6-46.4)	28.3 (19.7-40.7)
**GMT D21**	69.3 (40.3-119.2)	71.3 (49.6-102.3)	107.5 (65.5-176.4)	23.0 (14.9-35.4)
**Seroconversion D21**	34.5% (17.9-54.3)	53.3% (34.3-71.7)	44.4% (25.5-64.7)	0.0% (0.0-11.6)
**GMI (D21/D0)**	2.5 (1.8-3.6)	4.0 (2.9-5.7)	3.7 (2.3-5.9)	0.8 (0.6-1.0)

**Strain**	A/California/07/2009, A/Victoria/361/2011, B/Wisconsin/1/2010 (Yamagata lineage), B/Brisbane/60/2008 (Victoria lineage)			
**Dose**	3µg/Strain (Total HA: 12µg)	9µg/Strain (Total HA: 36µg)	15µg/Strain (Total HA: 60µg)	Placebo
**MN titers B (newly recommended Yamagata lineage for 2014-2015 season)**	**B/Massachusetts/2/2012**			
**GMT D0**	14.6 (9.8-21.8)	9.8 (6.3-15.1)	18.5 (11.2-30.7)	13.2 (8.9-19.4)
**GMT D21**	27.9 (16.6-47.0)	30.6 (19.7-47.4)	62.6 (38.7-101.5)	12.7 (8.7-18.6)
**Seroconversion D21**	13.8% (3.9-31.7)	26.7% (12.3-45.9)	33.3% (16.5-54.0)	0.0% (0.0-11.6)
**GMI (D21/D0)**	1.9 (1.4-2.5)	3.1 (2.3-4.3)	3.4 (2.1-5.4)	1.0 (0.9-1.0)

1.3.3 RESULTS FOR PLANT GLYCANS

The serum at Day 0, 21 were analyzed for the presence of IgE to plant-specific β(1-2) xylose and α(1-3)fucose carbohydrate residues according to the bromelain test (ImmunoCap, Thermo Scientific). This test is used to identify subjects with potentially clinically-relevant allergies to plant glycans. The level of IgEs to bromelain (glycoprotein extracted from pineapple) are classified into five grades (0 to 4) corresponding to symptom levels. As shown in Table 5, vaccination with the quadrivalent VLP vaccine did not increase the IgE level of any subject 21 days post-dose, confirming the general safety of plant glycans found in the plant-derived vaccine. By comparison, one subject in the placebo group showed an increase in anti-bromelain glycan. This is most probably due to natural exposure to allergens.

Table 5: IgE responses to plant glycans according to the bromelain assay

CLINICAL TRIAL	GROUP	NUMBER OF SUBJECTS WITH IGES ≥GRADE 1 TO BROMELAIN AT SCREENING	NUMBER OF SUBJECTS THAT SHOWED AN IGE INCREASE 1 MONTH AFTER VACCINATION
PHASE 1-2 WITH VLP QUADRIVALENT VACCINE (ONE DOSE)	3 µg/strain	0/29	0/29
	9 µg/strain	0/30	0/30
	15 µg/strain	1/27	0/27
	Placebo	0/30	1/30

Medicago is not expecting that the dosages proposed in the phase 2 study will have any impact regarding development of allergy symptoms and IgEs levels but this will of course, be part of the investigation for the VLP quadrivalent product.

1.3.4 RESULTS FOR CMI

In order to better characterize the CMI generated by the plant-derived quadrivalent VLP vaccine, the peripheral blood mononuclear cells (PBMC) were collected from 10 subjects randomly chosen in the different treatment groups. Unfortunately, some samples were not properly processed and cannot be used in the analysis. The technical problem was fixed but due to these issues we had to restrict the analysis to 6 Placebo subjects and 9 subjects who received 15 µg/strain of the quadrivalent VLP.

Homotypic immunity

A first analysis revealed qualitative changes in the response between patients who received 15 µg/strain quadrivalent VLP vaccine and Placebo (Figure 1). $CD4^+$ cells from vaccinated patients stimulated *ex vivo* with each of the 4 VLP included in the quadrivalent VLP vaccine exhibited a greater diversity in the polyfunctional response with a higher proportion of $CD4^+$ positive for at least 2 out of the 4 functional markers, namely IFN-γ, IL-2, TNF-α and CD107a as compared to Placebo, 21 days after immunization (Figure 1).

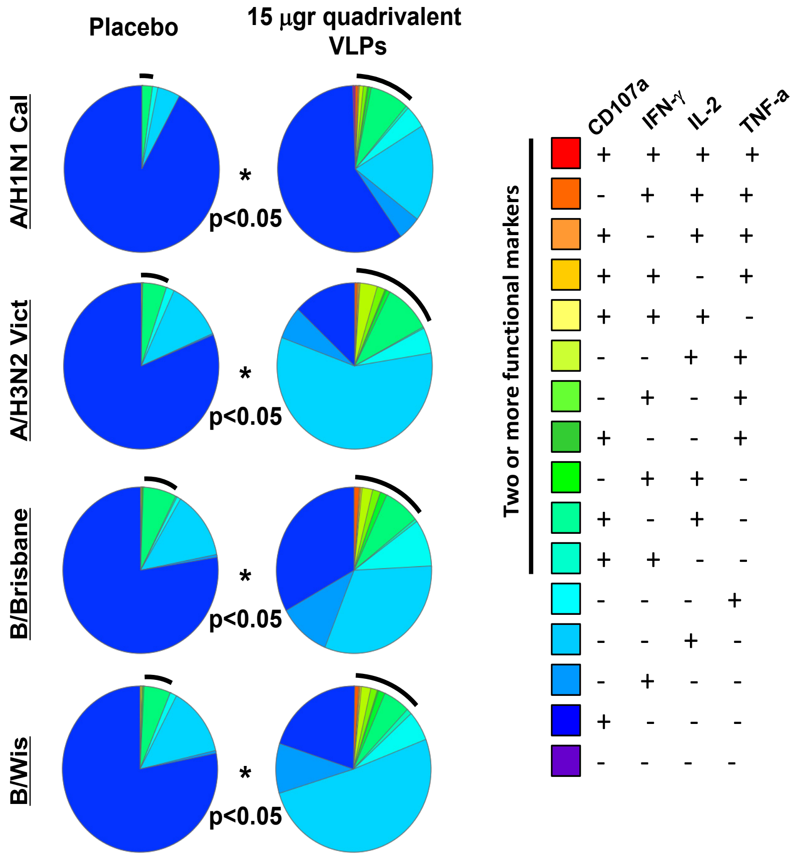

Figure 1: Relative distribution of the CD4 T cell functional response to *ex vivo* stimulation with each of the 4 VLPs included in the quadrivalent VLP vaccine. All the functional signature based on the expression of the 4 functional markers (CD107a, IFN-γ, IL-2 and TNF-α) were color-ranked from the more poly-functional (quadruple positive = red) to the quadruple negative (purple). The proportion of CD4 T cell expressing two or more functional markers are highlighted in black.

* p<0.05 indicates significant differences between the distribution of the response in Placebo and quadrivalent VLP vaccine groups (permutation analysis, SPICE v5.3 http://exon.niaid.nih.gov/spice/)

The presence of polyfunctional T cells has been associated with protection and better clinical outcome in several infections[11-13].

We also observed a recurrent increase of particular functional signatures in PBMCs from patients who received the quadrivalent VLP vaccine after *ex vivo* stimulation with each of the 4 VLPs included in the vaccine, 21 days after the immunization. All these functional signatures involved IFN-γ, alone or with other functional maker(s) (Figure 2).

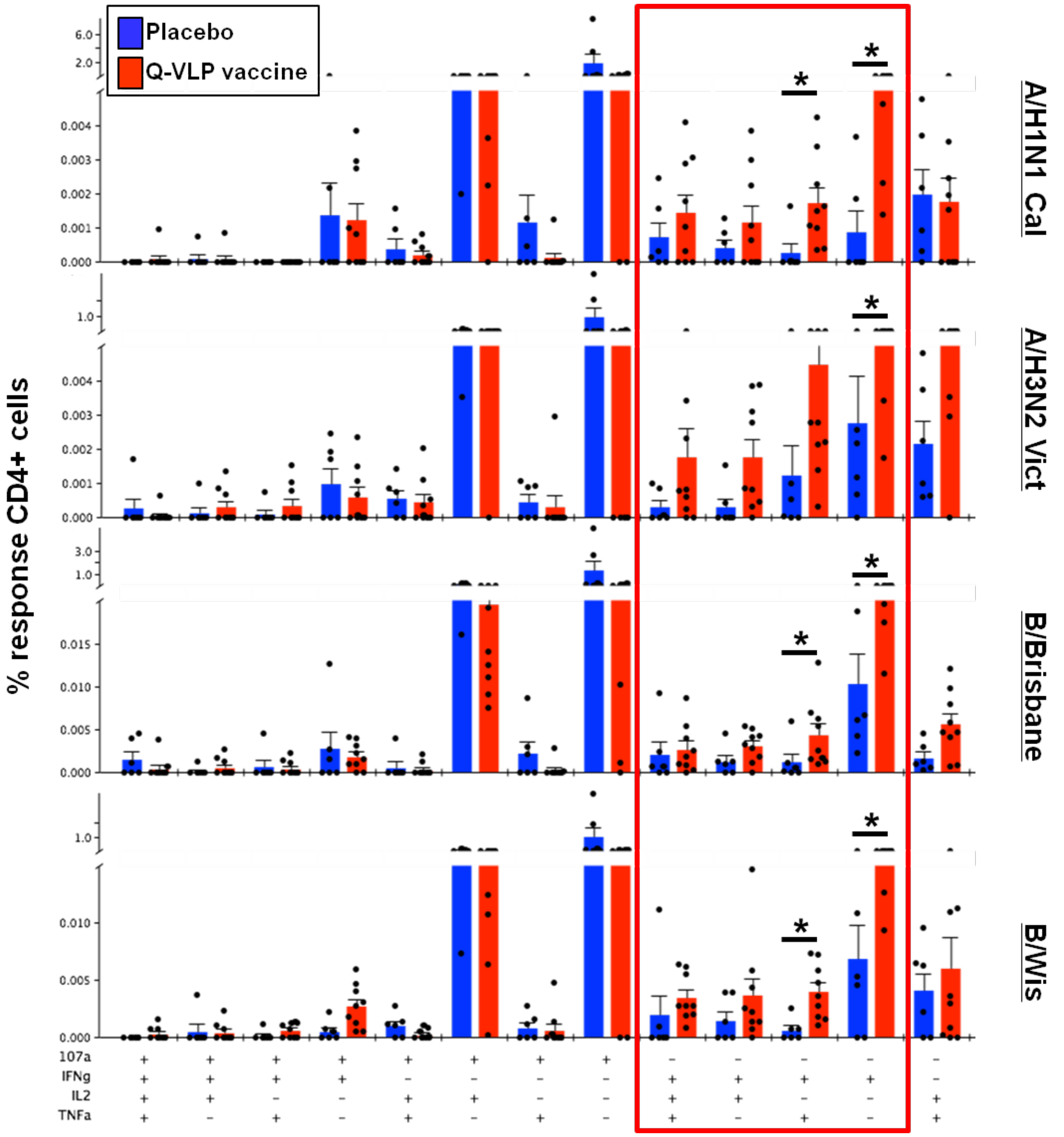

Figure 2: Details of the functional response of the CD4 T-cell based on the expression of the 4 markers (CD107a, IFN-γ, IL-2 and TNF-α) after *ex vivo* stimulation with each of the 4 VLPs included in the quadrivalent VLP vaccine 21 days after immunization. Histograms represent the percentage (mean ± SEM) of positive cells for each functional signature detailed on the bottom of the bar graphs.

* Indicate significant ($p<0.05$) differences between Placebo and quadrivalent VLP vaccine (Wilcoxon signed rank test, SPICE v5.3 http://exon.niaid.nih.gov/spice/).

The presence of IFN-γ indicates an effector phenotype. Effector cells have been associated with the clearance of virus and the effector memory cells are seen as one of the most desirable population to be elicited by vaccination[11,12].

The heterogenicity of the T-cell memory response has been extensively investigated over the last 15 years however remaining largely unexplained. Basically, memory T-cell can be defined as Central Memory (CM), Transitional memory (TM) and Effector Memory (EM) based on the expression of CD45RA, CCR7 and CD27 at the surface of the T-cell (Table 6)[12].

Table 6: Sub-populations of memory T-cell based on the expression of cell-surface markers

	CD45RA	CCR7	CD27
CENTRAL MEMORY	-	+	+
TRANSITIONAL MEMORY	-	-	+
EFFECTOR MEMORY	-	-	-

We observed an increase of the same functional signatures in patients who received the quadrivalent VLP vaccine when analysis was restricted to the CD4+ TM cells ($CD45RA^-$/$CCR7^-$/$CD27^+$) and a significant difference was observed in CD4+ TM IFN-γ monovalent cells for each of the 4 VLPs included in the quadrivalent VLP vaccine after *ex vivo* stimulation of the PBMCs 21 days after immunization (Figure 3). The absence of statistically significant difference in some of the 3 other functional signatures involving IFN-γ highlighted in the Figure 3 is likely due to low statistical power resulting from the reduced number of samples.

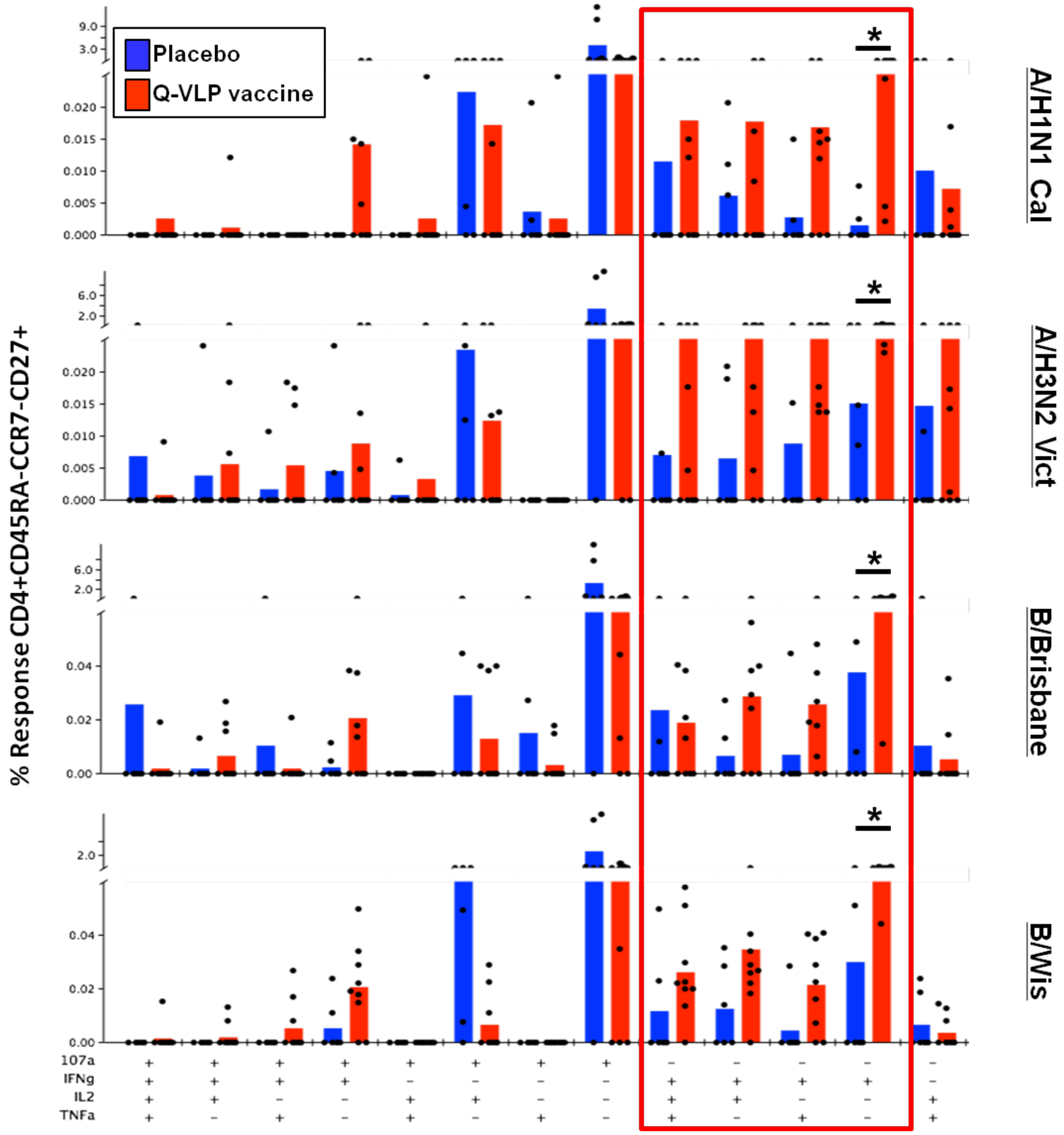

Figure 3: Details of the functional response of the TM CD4 T-cells based on the expression of the 4 markers (CD107a, IFN-γ, IL-2 and TNF-α) after *ex vivo* stimulation with each of the 4 VLPs included in the quadrivalent VLP vaccine 21 days after immunization.

Histograms represent the percentage (mean) of positive cells for each functional signature detailed below the bar graphs.

* Indicate significant ($p<0.05$) difference between Placebo and quadrivalent VLP vaccine (Wilcoxon signed rank test, SPICE v5.3 http://exon.niaid.nih.gov/spice/).

Heterotypic immunity

The PBMCs were also stimulated *ex vivo* with a peptide pool consisting in 15-mer peptides overlapping by 11 amino acids and covering the entire sequence of B/Massachusetts HA protein to test for potential heterotypic CIM induced by the quadrivalent VLP vaccine.

Twenty-one days after immunization, patients who received the quadrivalent VLP vaccine had significantly more cross-reactive $CD4^+$ T-cells to B/Massachusetts HA peptide pool than Placebo (Figure 4). A similar increase, although not statistically significant, was observed in $CD8^+$ T-cells.

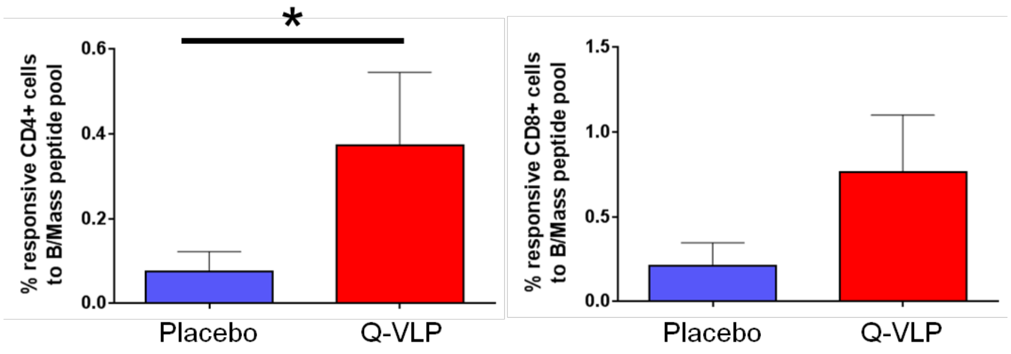

Figure 4: Percentage of total cross-reactive CD4 (left) and CD8 (right) T-cell to B/Massachusetts HA protein peptide pool.
Histograms represent the percentage (mean ± SEM) of responsive $CD4^+$ or $CD8^+$ T-cells defined as a T-cell expressing at least one of the four functional markers after *ex vivo* stimulation with the peptide pool.
* Indicate significant ($p<0.05$) difference between Placebo and quadrivalent VLP vaccine (Mann Whitney test, GraphPad Prism v6.03).

The detailed analysis of the response revealed that vaccination with the quadrivalent VLP vaccine elicited a significant increase of the highly poly-functional $CD107a^-$/IFN-γ^+/IL-2^+/TNF-α^+ CD4 T-cells (Figure 5A). We also observed an increase, although non statistically significant (p=0.06) of the proportion of the quadruple positive $CD107a^+$/IFN-γ^+/IL-2^+/TNF-α^+ CD4 T-cells in patients who received the quadrivalent VLP as compared to the Placebo, 21 days after immunization.

A similar pattern was observed in TM CD4 T-cells which reflect the establishment of a population of cross-reactive transitional memory T-cells in patients vaccinated with the plant-derived quadrivalent VLP (Figure 5B).

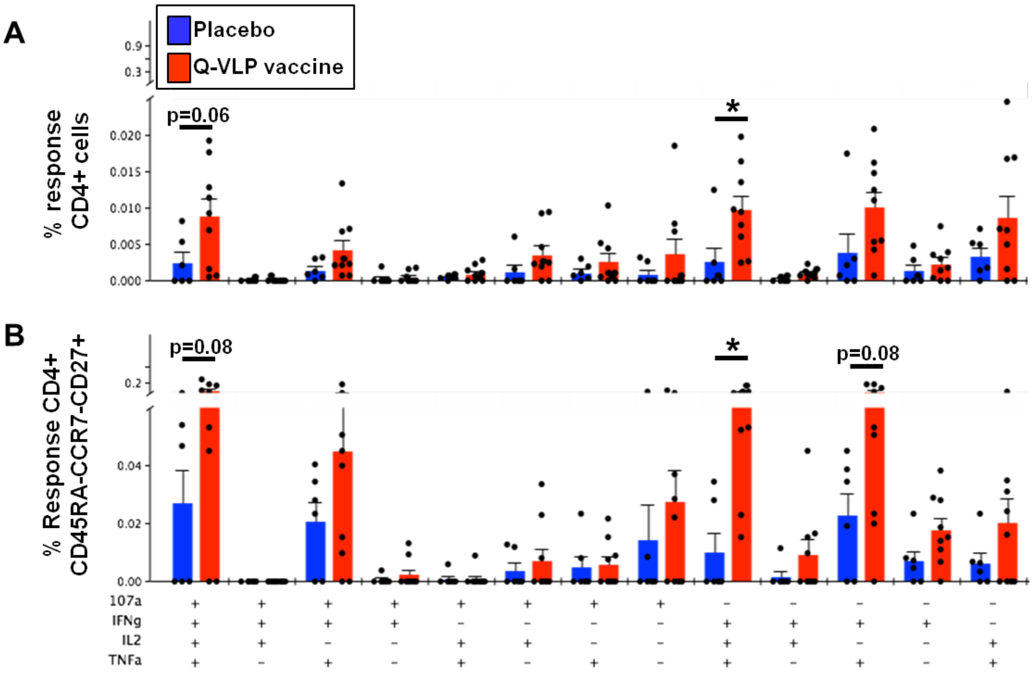

Figure 5: Details of the functional response of CD4 (A) and TM CD4 T-cells based on the expression of the 4 markers (CD107a, IFN-γ, IL-2 and TNF-α) after *ex vivo* stimulation with B/Massachusetts HA protein peptide pool 21 days after immunization.

Histograms represent the percentage (mean ± SEM) of positive cells for each functional signature detailed below the bar graphs.

* Indicate significant (p<0.05) differences between Placebo and quadrivalent VLP vaccine (Wilcoxon signed rank test, SPICE v5.3 http://exon.niaid.nih.gov/spice/).

As stated above, the presence of poly-functional cells, and especially poly-functional effector memory cells, has been associated with better protection and clinical outcome in several infections.

1.4. Clinical Studies - Safety results from Phase 2 trial with alum-adjuvanted H5 VLP Vaccine in healthy adults 18-64 years of age.

In this trial, dosages of 20, 30 or 45 µg of the monovalent H5 VLP vaccine were mixed with Alhydrogel®. By comparison, the proposed Phase 2 trial plans for administration of a maximum of 60 µg total HA mixed with the adjuvant, thus Medicago considers the safety data shown in Table 7 are supportive of the proposed clinical trial. The use of Alhydrogel® improved the antibody and T cell response compared to the non-adjuvanted formulation (data not shown).

Table 7: Part A: Solicited Signs and Symptoms (Safety Set) for Phase II trial with H5 VLP Vaccine

	FIRST DOSE, N (%)[1]						SECOND DOSE, N (%)[1]					
	H5 VLP WITH ADJUVANT			45 µg H5 VLP without adjuvant			H5 VLP WITH ADJUVANT			45 µg H5 VLP without adjuvant		
	20 µg (N=30)	30 µg (N=30)	45 µg (N=30)	(N=30)	Placebo (N=15)	P-value[2]	20 µg (N=30)	30 µg (N=30)	45 µg (N=30)	(N=30)	Placebo (N=15)	P-value[2]
ANY SYMPTOM, N (%)	29 (96.7)	22 (73.3)	20 (66.7)	18 (60.0)	4 (26.7)	<0.001	20 (66.7)	16 (55.2)	18 (60.0)	10 (34.5)	3 (21.4)	0.016
LOCAL SYMPTOM	25 (83.3)	22 (73.3)	19 (63.3)	15 (50.0)	4 (26.7)	0.002	18 (60.0)	16 (55.2)	17 (56.7)	8 (27.6)	3 (21.4)	0.017
Redness	2 (6.7)	0	1 (3.3)	4 (13.3)	3 (20.0)	-	2 (6.7)	2 (6.9)	0	2 (6.9)	2 (14.3)	-
Injection Site Swelling	3 (10.0)	3 (10.0)	3 (10.0)	3 (10.0)	2 (13.3)	-	0	2 (6.9)	0	2 (6.9)	1 (7.1)	-
Pain	25 (83.3)	22 (73.3)	19 (63.3)	12 (40.0)	1 (6.7)	-	17 (56.7)	15 (51.7)	17 (56.7)	8 (27.6)	2 (14.3)	-
GENERAL SYMPTOM	10 (33.3)	4 (13.3)	10 (33.3)	9 (30.0)	3 (20.0)	0.312	7 (23.3)	6 (20.7)	9 (30.0)	4 (13.8)	1 (7.1)	0.426
Fever[3]	1 (3.3)	0	0	0	0	-	0	0	0	0	0	-
Headache	5 (16.7)	2 (6.7)	2 (6.7)	3 (10.0)	1 (6.7)	-	0	1 (3.4)	2 (6.7)	2 (6.9)	1 (7.1)	-
Muscle Ache	5 (16.7)	2 (6.7)	6 (20.0)	6 (20.0)	2 (13.3)	-	7 (23.3)	4 (13.8)	7 (23.3)	4 (13.8)	1 (7.1)	-
Swelling[4]	0	0	0	1 (3.3)	1 (6.7)	-	0	1 (3.4)	1 (3.3)	0	1 (7.1)	-
Malaise	1 (3.3)	1 (3.3)	2 (6.7)	1 (3.3)	2 (13.3)	-	0	3 (10.3)	1 (3.3)	1 (3.4)	1 (7.1)	-
Chills	1 (3.3)	0	0	2 (6.7)	1 (6.7)	-	0	0	0	1 (3.4)	0	-
Joint Aches	0	1 (3.3)	1 (3.3)	1 (3.3)	1 (6.7)	-	2 (6.7)	1 (3.4)	0	0	1 (7.1)	-
Fatigue	5 (16.7)	0	4 (13.3)	2 (6.7)	2 (13.3)	-	0	1 (3.4)	2 (6.7)	2 (6.9)	1 (7.1)	-

1 Percentage is based on the number of subjects who received the dose.
2 P-value compared to placebo based on Fisher's exact test.
3 Fever is defined as oral temperature >38°C.
4 Includes swelling of axilla, neck, joints, or chest.

Also, during the phase 2 trial with alum-adjuvanted H5 VLP vaccine there was no increase in IgE to plant glycans as measured with the bromelain assay (data not shown).

1.5. Pre-Clinical Studies

Please refer to Section 4.0 of the Investigator Brochure.

2. TRIAL OBJECTIVES AND ENDPOINTS

2.1 Primary Objectives

The primary objective of this study is to assess the immunogenicity, safety, and tolerability of one dose of a plant-derived Seasonal VLP Quadrivalent Influenza Vaccine given non-adjuvanted at dose levels of 15 µg, 30 µg, and 60 µg per strain or adjuvanted (Alhydrogel®) at a dose levels of 7.5 and 15 µg per strain compared to a placebo (saline).

One primary objective of this study is to measure the capacity of the VLP quadrivalent vaccine to induce specific antibodies against the H1N1, H3N2, and the B/Brisbane and B/Massachusetts virus strains contained in the vaccine. The European CPMP has established criteria on which seasonal influenza vaccines obtain licensure in a number of countries. These criteria are based on

the level of antibodies induced and the percentage of subjects achieving a target antibody titer measured by the HI test. The HI test will measure the level of antibodies for the A/California/7/2009 H1N1, A/Victoria/361/2011 H3N2, B/Massachusetts/2/2012 (Yamagata lineage) and B/Brisbane/60/2008 (Victoria lineage) vaccine strains. As outlined in the next section, the Center for Biologics Evaluation and Research (CBER) also has criteria very similar to the CPMP, for evaluation of antibody levels in adults and elderly that are required for licensure in the US and these are outlined in the next section.

2.1.1 PRIMARY ENDPOINTS

As primary endpoint, immunogenicity will be evaluated primarily by measuring the serum HI response induced in subjects. Serum HI responses will be described in terms of Geometric mean titers (GMTs) of hemagglutination inhibition (HI) antibody on Day 0 and Day 21. Follow-up serology samples for GMTs will be collected at Day 201 (6-months follow-up period that applies to the investigational vaccine).

In accordance with the CPMP guidance (Committee for Proprietary Medicinal Products [CPMP]. Note for guidance on harmonization of requirements for influenza vaccines. CPMP/BWP/214/96. The European Agency for the Evaluation of Medicinal Products (EMEA), March 1997) most recent serological criteria for influenza vaccine immunogenicity in adults (<65 years of age and ≥65 years of age) in FDA Guidelines[14] **(see in bold for CBER criteria)**, the three following criteria (seroconversion rate, seroprotection rate and seroconversion factor or GMFR (Geometric Mean Fold Rise)) are to be considered in the immunogenicity assessment of the seasonal influenza vaccine:

For adults <65 years of age:

- Seroconversion rate: the proportion of subjects in a given treatment group with either a ≥4-fold increase in reciprocal HI titers between Day 0 and Day 21; or a rise of undetectable HI titer (i.e. <10) pre-vaccination, (Day 0) to an HI titer of ≥40 at Day 21 post-vaccination. The percent of subjects achieving seroconversion for HI antibody should exceed 40%. **The lower bound of the two-sided 95% confidence interval (CI) for the percent of subjects achieving seroconversion for HI antibody should meet or exceed 40%.**
- Seroprotection rate: the proportion of subjects in a given treatment group attaining a reciprocal HI titer of ≥40 at 21 days post-vaccination (the percentage of vaccine recipients with a serum HI titer of at least 1:40 following vaccination). The percent of subjects achieving an HI antibody titer >1:40 should exceed 70%. **The lower bound of the two-sided 95% CI for the percent of subjects achieving an HI antibody titer >1:40 should meet or exceed 70%.**
- Seroconversion factor or GMFR (Geometric Mean Fold Rise) is the geometric mean of the ratio of GMTs (Day 21/Day 0). Mean geometric increase >2.5.

For adults ≥65 years of age:

- Seroconversion rate: the proportion of subjects in a given treatment group with either a ≥4-fold increase in reciprocal HI titers between Day 0 and Day 21; or a rise of undetectable HI titer (i.e. <10) pre-vaccination, (Day 0) to an HI titer of ≥40 at Day 21 post-vaccination. The percent of subjects achieving seroconversion for HI

antibody should exceed 30%. **The lower bound of the two-sided 95% CI for the percent of subjects achieving seroconversion for HI antibody should meet or exceed 30%.**

- Seroprotection rate: the proportion of subjects in a given treatment group attaining a reciprocal HI titer of ≥40 at 21 days post-vaccination (the percentage of vaccine recipients with a serum HI titer of at least 1:40 following vaccination). The percent of subjects achieving an HI antibody titer >1:40 should exceed 60%. **The lower bound of the two-sided 95% CI for the percent of subjects achieving an HI antibody titer >1:40 should meet or exceed 60%.**
- Seroconversion factor or GMFR (Geometric Mean Fold Rise) is the geometric mean of the ratio of GMTs (Day 21/Day 0). Mean geometric increase >2.0.

The FDA's criteria for the population of ≥65 years of age, as described above, will be used to analyze the primary immunogenicity endpoints achieved within each treatment groups of 75 subjects. Refer to Section 10.3 for determination of sample size.

Results will also be presented by age strata (50-64 and 65 and older, in a ratio of 1:1 [37 and 38 subjects in each treatment group respectively]). Considering the group size of each age strata per treatment group is too small to use the FDA criteria to analyze the immunogenicity endpoints, European CPMP criteria for respective age strata will be used to analyze the seroconversion factor (or GMFR), seroconversion rate and seroprotection rate obtained in each age strata, for each treatment group, as described above:

As another primary endpoint, safety and tolerability will also be assessed, including solicited local events as follows: erythema (redness), swelling, and pain at the injection site. The following systemic symptoms will also be solicited: headache, fever, muscle aches, joint aches, fatigue, chills, malaise, swelling in the axilla and neck. The causal relationship of the solicited local and systemic reactions will be graded as: mild (1), moderate (2), severe (3) or potentially life threatening (4). The occurrence of any AEs/SAEs, history/symptom-directed physical examination findings, clinical laboratory results, and vital signs (including oral temperature [OT]) will be assessed. The signs and symptoms will be measured and reported by each study subject from the time of study vaccine administration up to seven days post-vaccination (from Day 0–Day 1 and from Day 2–Day 7).

- Percentage, intensity, and relationship of immediate complaints (30 minutes post-vaccination);
- Percentage, intensity, and relationship to vaccination of solicited local (erythema, swelling, and pain at the injection site) and systemic signs and symptoms (headache, muscle aches, joint aches, fatigue, chills, malaise, and swelling in the axilla or neck) for 7 days following the study vaccine;
- Percentage, intensity, and relationship of unsolicited local and systemic signs and symptoms for 21 days following study vaccine;
- Occurrences of all AEs/SAEs;
- Occurrences of new onset of a chronic disease (NOCD);
- The number and percentage of subjects with normal and abnormal urine, haematological, and biochemical values at Screening, Days 3 and 201.

2.2 Secondary Objectives

The secondary objectives of this study include:

- The assessment of the reactivity of antibodies induced by one dose of seasonal VLP quadrivalent influenza vaccine at the three non-adjuvanted dose levels (15 µg, 30 µg, and 60 µg/strain) or at the two adjuvanted dose levels (7.5 µg and 15 µg/strain) compared to the placebo, as measured by HI antibody titers for the homologous influenza strains, described in terms of Geometric mean titers (GMTs) of hemagglutination inhibition (HI) antibody on Day 0 and Day 21. Follow-up serology samples for GMTs will be taken at Day 201. This will be assessed using measures of GMFR, seroconversion rate, and of percentage of subjects with a detectable HI antibody response (≥lower limit of quantification; ≥LOQ) at Days 0, 21, and 201.
- The assessment of the reactivity of antibodies induced by one dose of VLP quadrivalent influenza vaccine at the three non-adjuvanted dose levels (15 µg, 30 µg and 60 µg/strain) or at the two adjuvanted dose levels (7.5 µg and 15 µg/strain) compared to the placebo, as measured by microneutralisation (MN) antibody titers for the homologous influenza strains. This will be assessed using measures of GMFR, seroconversion rate, and of percentage of subjects with a detectable MN antibody response (≥LOQ) at Days 0, 21, and 201, against the homologous strains.
- The assessment of the cross-reactivity of antibodies induced by one dose of VLP quadrivalent influenza vaccine at the three non-adjuvanted dose levels (15 µg, 30 µg and 60 µg/strain) or at the two adjuvanted dose levels of (7.5 µg and 15 µg/strain) compared to the placebo, as measured by HI and MN antibody titers for heterologous influenza strains. This will be assessed using measures of GMFR, seroconversion rate, seroprotection rate (for HI only), and of percentage of subjects with a detectable HI or MN antibody response (≥LOQ) at Days 0, 21, and 201.

2.2.1 SECONDARY ENDPOINTS AND MEASURES

For the HI test against the homologous influenza vaccine strains:

As mentioned in primary objectives, immunogenicity will be evaluated by measuring the serum antibody response induced in subjects, using the HI test against the vaccine strains. A secondary endpoint from this analysis will be the measurement of the percentage of subjects with a detectable HI antibody response (≥LOQ) at Days 0, 21, and 201, against the homologous strains.

For the HI test against the heterologous influenza strains:

GMTs of HI antibody for heterologous influenza strains on Day 0 and Day 21. Follow-up serology samples for GMTs will be collected at Day 201.

GMTs endpoints will be analyzed as follows:

- Seroconversion factor or GMFR: is the geometric mean of the ratio of GMTs (Day 21/Day 0).
- Seroconversion rate: the proportion of subjects in a given treatment group with either a ≥4-fold increase in reciprocal HI titers between Day 0 and Day 21; or a rise of undetectable HI titer (i.e. <10) pre-vaccination, (Day 0) to an HI titer of ≥40 at Day 21 post-vaccination.

- Seroprotection rate: the proportion of subjects in a given treatment group attaining a reciprocal HI titer of ≥40 at 21 days post-vaccination (the percentage of vaccine recipients with a serum HI titer of at least 1:40 following vaccination).
- The percentage of subjects with a detectable HI antibody response (≥LOQ) at Days 0, 21, and 201.

For the MN test against homologous and heterologous influenza strains:

GMTs of MN antibody for homologous and heterologous H1N1, H3N2, and B (B/Bris and B/Mass) strains on Days 0 and 21. Follow-up serology samples for GMTs will be collected at Day 201. GMTs will be analyzed as follows:

- Seroconversion factor or GMFR: is the geometric mean of the ratio of GMTs (Day 21/Day 0).
- Seroconversion rate: the proportion of subjects in a given treatment group with either a ≥4-fold increase in reciprocal MN titers between Day 0 and Day 21; or a rise of undetectable MN titer (i.e. <10) pre-vaccination, (Day 0) to an MN titer of ≥40 at Day 21 post-vaccination.
- The percentage of subjects with a detectable MN antibody response (≥LOQ) at Days 0, 21 and 201.

2.3 Exploratory Objectives

The exploratory objectives of this study are as follows:

- To evaluate cellular responses induced by one dose of a Seasonal VLP Quadrivalent Influenza Vaccine given non-adjuvanted at dose levels of 15 µg, 30 µg, and 60 µg per strain or adjuvanted (Alhydrogel®) at a dose levels of 7.5 and 15 µg per strain compared to a placebo. T cell responses will be assessed at Days 0, 21, and 201 in a subset of subjects.
- Since the HA protein of the plant-derived VLP quadrivalent vaccinecontains plant-specific glycans, there is at least a theoretically concern that these glycans might trigger an allergic response. In clinical studies with Medicago's H5 VLP, H1 VLP and VLP quadrivalent vaccines to date, no allergic reactions have occurred and there has been no long-term induction of IgE to plant-specific sugars. An exploratory objective of this VLP quadrivalent vaccine study will be to monitor for IgE antibodies directed against plant glycans induced by vaccination (timeframe: Days 0, 21, and 201).
- Humoral response induced approximately 21 days after administration of the licensed flu vaccine (for those who will receive it at the clinical site) will also be evaluated.

2.3.1 EXPLORATORY ENDPOINTS AND MEASURES

Cell-mediated immune response (CMI):

PBMCs will be isolated to assess the CMI induced by the quadrivalent vaccine. The role of cellular immunity in influenza is currently the subject of considerable research. In particular, some cellular responses appear to be good correlates of protection in the elderly as well as conferring cross-reactivity against other viruses. A vaccine that can induce protection against

drifted strains would clearly be a significant advantage for seasonal use when vaccines are typically produced months in advance of their use.

Activation of CD4+ and CD8+ T cells of the adaptive immunity is also important in protection against viral infection and is not assessed by the measurement of specific antibodies. Cellular immune response analysis with PBMC collected in our previous clinical study (NCT01244867) revealed that the H5 VLP vaccine induced long lasting (6 months) polyfunctional CD4+ cells. Polyfunctional memory T cells are widely considered to have better effector function than monofunctional. [12] Additional analyses revealed that these CD4+ T cells are also cross-reactive towards other influenza strains. During the phase 1 trial conducted with the non-adjuvanted H1 VLP made for the A/California/7/09 H1N1 strain (NCT01302990), it was demonstrated that the plant-derived H1 VLP induced a higher percentage of polyfunctional CD4+ T cells than the comparator vaccine (Fluzone® trivalent vaccine form Sanofi USA) and that only the plant-derived vaccine induced T cells cross-reactive for other influenza strains. PBMC were also collected from a subset of subjects during the phase 1-2 trial with our seasonal VLP quadrivalent vaccine (NCT01991587). These analyses are still ongoing but it can be said that subjects tested so far showed good CD4+ and CD8+ T cell responses against the four strains included in the vaccine. These features are important differentiation factors for the plant-derived vaccines that are of high importance for seasonal vaccine and also in the context of a pandemic. The quality of the T cell response induced by the plant-derived vaccine is thought to play an important role in the protection against heterologous influenza strains when antibody levels are low. Medicago plans to continue investigating the nature of both humoral and cellular immune responses induced by the plant-derived VLP quadrivalent in this study.

Blood samples for PBMCs isolation will be taken from 15 subjects enrolled per treatment group on Days 0, 21, and 201, at the MUHC site only.

CMI assay could include: Flow cytometry panel to characterize subset cell populations, including CD3, CD4, CD8, CD45RA, CD27 and CD16/CD56 and viability markers (Aqua) as well as functional markers including IL-2, TNFα and IFNγ and cytokines in combination with the cell surface expression of CD107a (lysosomal-associated membrane protein-1, or LAMP-1), granzyme B and perforin (clone B-D48) as markers of T cell degranulation and cytolytic activity.

Antibody to plant-specific glycans:

The possible induction of antibodies to plant-specific sugars found on the VLP vaccine will be assessed using the ImmunoCAP technology that measures IgE specific to the cross-reactive carbohydrate determinant (CCD) MUXF3 present on bromelain (ImmunoCAP: Pharmacia&Upjohn, Uppsala, Sweden). Bromelain contains only the MUXF3 carbohydrate epitope. This CCD is found on many plant allergens and can trigger histamine release by mast cells and basophils *in vitro*[15-17]. Results of this assay are reported in kUA/l with an interpretation of potential clinical impact. Grade 0 is considered to be 'negative', Grades 1 and 2 are 'low-' and 'moderate positive' respectively while Grades 3-6 define varying degrees of 'high' and 'very high' positive. Values higher than 2 are thought to be predictive of clinically-relevant allergy. Data will be analyzed at Days 0, 21, and 201.

Humoral immune response ≥Day 42, after licensed flu vaccine administration

The Humoral response induced~21 days after administration of the licensed flu vaccine (Day ≥42) (for those who will receive it at the clinical site) will also be evaluated as measured by GMTs of HI antibody for influenza strains on Day 21 (baseline) and Day ≥42.

GMTs endpoints will be analyzed as follows:

- Seroconversion factor or GMFR: is the geometric mean of the ratio of GMTs (Day ≥42/Day 21).
- Seroconversion rate: the proportion of subjects in a given treatment group with either a ≥4-fold increase in reciprocal HI titers between Day 21 and Day ≥42; or a rise of undetectable HI titer (i.e. <10) pre-vaccination at Day 21 to an HI titer of ≥40 at Day ≥42 post-vaccination.
- Seroprotection rate: the proportion of subjects in a given treatment group attaining a reciprocal HI titer of ≥40 at ≥42 days post-vaccination (the percentage of vaccine recipients with a serum HI titer of at least 1:40 following vaccination).
- The percentage of subjects with a detectable HI antibody response (≥LOQ) at ≥42.

3. INVESTIGATOR AND TRIAL CENTER

Table 8: Sites and Responsible Key Personnel

Sponsor	Clinical Sites	DSMB	Other key roles	
**Medicago R & D Inc.**<br>1020, route de l'Eglise,<br>bureau 600<br>Sainte-Foy, Quebec,<br>Canada, G1V 3V9<br>Office: 1.418.658.9393<br>Fax: 1.418.658.6699<br>**Clinical Study Director**<br>Sonia Trepanier<br>Tel.: 1.418.658.9393 ext. 137<br>Mobile: 1.418.655.7158<br>trepaniers@medicago.com<br>**Sponsor Contact, Manager Clinical studies**<br>Sebastien Soucy<br>Tel.: 1.418.658.9393 ext. 122<br>Mobile: 1.418.446.2032<br>soucys@medicago.com<br>**Sponsor Medical Officer**<br>Dr. Brian J. Ward, M.D.<br>Research Institute of the MUHC<br>Montreal General Hospital<br>1650 Cedar Avenue,<br>Room L10-509<br>Montreal, Quebec,<br>Canada, H3G 1A4<br>Tel.: 1.514.934.1934 ext. 42810<br>After hours:<br>Mobile: 1.514.952.6583<br>Pager: 1.514.921.6953<br>brian.ward@mcgill.ca	**Sites and PIs**<br>MUHC-Vaccine Study Centre<br>14770 Boul.Pierrefonds, suite 204<br>Pierrefonds, Quebec,<br>Canada, H9H 4Y6<br>PI: Michael Libman, M.D.<br>Tel.: 514.624.7855<br>michael.libman@mcgill.ca<br>Centre de recherche CHU de Québec<br>2400 d'Estimauville,<br>Québec, Quebec,<br>Canada, G1E 7G9<br>PI: Guy Boivin, M.D.<br>Tel.: 418.525.4444 ext. 47762<br>guy.boivin@crchudequebec.ulaval.ca<br>inVentiv Health Clinique<br>2500 Einstein<br>Québec, Quebec,<br>Canada, G1P 0A2<br>PI: Richard Larouche, M.D.<br>Tel.: 418.527.4000 ext 7775<br>richard.larouche@inventivhealth.com	**Members of DSMB**<br>Dr. Brian J. Ward, M.D.<br>Research Institute of the MUHC<br>brian.ward@mcgill.ca<br>Dr. Michael Libman, M.D.<br>Research Institute of the MUHC<br>michael.libman@mcgill.ca<br>Dr. Guy Boivin, M.D.<br>Centre de recherche CHU de Québec<br>guy.boivin@crchudequebec.ulaval.ca<br>Dr. Richard Larouche, M.D<br>inVentiv Health Clinique<br>richard.larouche@inventivhealth.com<br>**3 External expert (voting members):**<br>Dr. Scott A. Halperin, M.D.<br>IWK Health Centre,<br>Dalhousie University,<br>5850/5980 University Avenue,<br>Halifax, Nova Scotia, Canada, B3K 6R8<br>Tel: 1.902.470.8141, Fax: 1.902.470.7232<br>scott.halperin@dal.ca<br>Dr. Joanne Langley, M.D.<br>IWK Health Centre,<br>Dalhousie University,<br>5850 University Avenue,<br>Halifax, Nova Scotia, Canada, B3K 6R8<br>Tel: 1.902.470.8141, Fax: 1.902.470.7232<br>joanne.langley@dal.ca<br>Dr. Allison McGeer, M.D.<br>Mount Sinai Hospital,<br>600 University Avenue<br>Toronto, Ontario, Canada, M5G 1X5<br>Tel: 1.416.586.3118, Fax: 1.416.586.3140<br>amcgeer@mtsinai.on.ca	**Safety laboratory**<br>**IVH**<br>inVentiv Health Clinique<br>2500 Einstein<br>Québec, Quebec,<br>Canada, G1P 0A2<br>Tel.: 418.527.4000<br>**MUHC, CR-CHUQ**<br>Les Laboratoires BNK Canada Inc.<br>500 Boul. Cartier Ouest, suite 205<br>Laval, QC, H7V 5B7<br>Tél: 450-688-4432<br>Fax: 450-688-7566<br>**Serological Laboratory (HI titer analysis)**<br>Medicago R&D Inc.<br>1020, route de l'Eglise, #600<br>Sainte-Foy, Quebec,<br>Canada, G1V 3V9<br>Michele Dargis, Director, Analytical Development<br>Tel.: 1.418.658.9393 ext. 150<br>Fax: 1.418.658.6699<br>dargism@medicago.com	**Data Management, Statistical Analysis, and Medical Report writing**<br>inVentiv Health Clinique<br>2500 Einstein<br>Québec, Quebec,<br>Canada, G1P 0A2<br>Fadi Achkar, B.Sc.<br>Project Data Manager<br>Tel.: 418.527.4000 ext.: 5227<br>fadi.achkar@inventivhealth.com<br>inVentiv Health Clinique<br>Eric Shink, Ph.D.<br>Senior Biostatistician<br>Tel.: 1 418 527 3067 x4391<br>Fax: 1 418 527 3456<br>eric.shink@inventivhealth.com<br>**Analytical Laboratory (PBMC analysis)**<br>ImmuneCarta Services<br>201 Avenue President-Kennedy,<br>Suite PK-3850<br>Montreal, Quebec,<br>Canada, H2X 3Y7<br>Jean-François Poulin, Ph.D.,<br>Principal Scientist<br>Tel.: 1.514.335.8350 ext. 54945<br>Fax: 1.514.333.0542<br>jpoulin@immunecarta.com

4. ETHICS APPROVAL

4.1 Ethical Conduct of the Study

The Ethical Review Boards for this study will be as follows:

For MUHC site:	BMD REB of the McGill University Health Center, Montreal, Quebec, Canada;
For CR-CHUQ site:	Comité d'éthique de la recherche du CHU de Quebec, Quebec, Canada
For inVentiv site:	IRB Services, Aurora, Ontario, Canada

The study will be conducted in accordance with the ethical principles that have their origins in the most recent update of the Declaration of Helsinki, in the International Conference on Harmonization (ICH) Guideline E6, in the GCP rules, and other applicable regulatory requirements.

5. INVESTIGATIONAL PLAN

5.1 Description of the Overall Trial Design and Plan

This will be a multiple centres (at least 3 clinical sites) phase 2, randomized, observer-blind, dose ranging, placebo-controlled clinical study in elderly. A total of four hundred fifty (450) subjects will be randomized in six (6) groups of 75 subjects. This study will be a cohort staggering (slow enrolment) for 3 non-adjuvanted dose levels (15 µg, 30 µg, and 60 µg VLP per strain), 2 adjuvanted dose levels (7.5 µg and 15 µg/strain) and a placebo-controlled group (ratio 1:1:1:1:1:1).

The 450 subjects (male and female subjects of 50 years of age or older) will be divided in 3 different cohorts. A 7-day safety data after the immunization will be collected by the clinical staff and will be reviewed by the Data and Safety Monitoring Board (DSMB). The DSMB members will determine if the clinical sites are allowed to continue immunization of the next cohort.

The proposed study schedule is presented in the Figure 6.

Figure 6: Proposed study schedule with approximate enrolment dates.

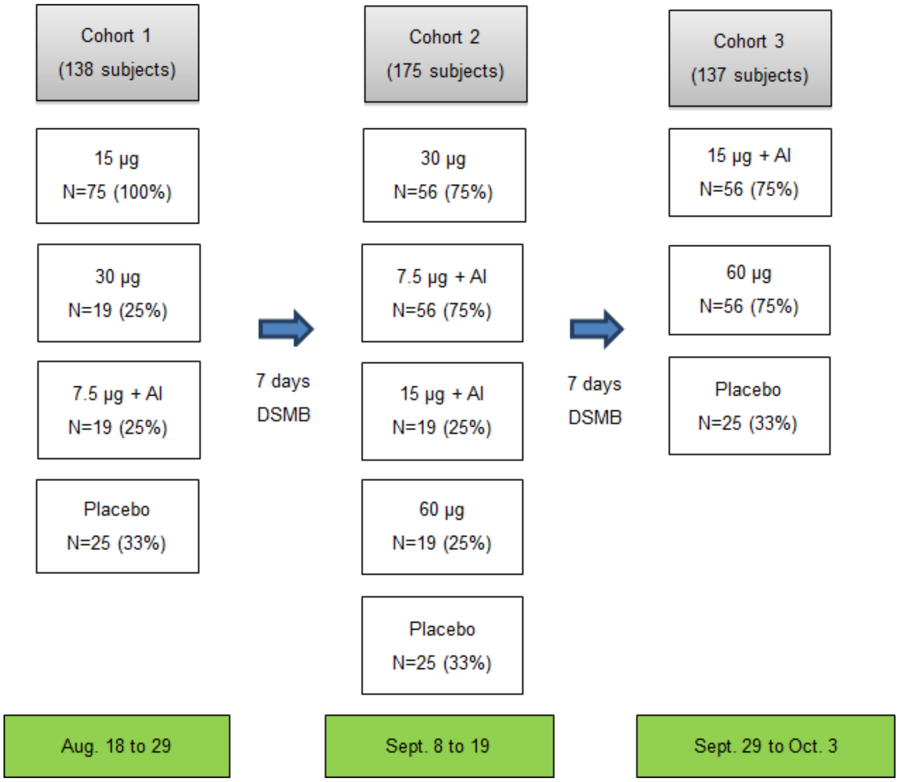

Al: Alhydrogel® 0.4% (0.5 mg per dose injected)

Subjects will also be randomized within 2 different age strata: subjects between 50 and 64 years old and subjects of 65 years or older, in a ratio of 1:1 (37 and 38 subjects in each treatment group, respectively). Every effort will be made to enrol an equal number of subjects in each age strata (a difference of around 10% between groups will be judged acceptable).

Potential subjects may be screened:

- Up to 90 days prior to study immunization using an ICF under a general pre-study protocol which covers medical and medication history, vital signs, and safety laboratory tests. All other screening procedures will be performed at Day 0, once the Study ICF will be signed. Or;
- Up to 30 days prior to immunization using the Study ICF and following all screening procedures as outlined in section 5.2.1.

Potential subjects will be interviewed over the phone and, if interested, invited to participate at a screening session at the clinical site (refer to Section 5.2.1 for complete details on procedures to be performed).

The subjects demonstrating a satisfactory baseline medical assessment (as stable health status with no exclusionary medical or psychiatric conditions) as judged by the Investigator will be allowed to participate in the Day 0 procedures.

In case the screening session is performed more than 30 days prior to subject immunization, a blood sample will be collected at Day 0, for safety analysis if deemed required by the Investigator. This extra sample will be stored. If changes in medical status, physical examination,

or vital signs occurred between screening session and Day 0 visit and are judged significant by the Investigator; then, this extra sample will be tested for routine safety labs. In this case, the subject's enrolment will be postponed until laboratory results are reviewed, found acceptable by the Investigator and then the subject will be allowed to participate in Day 0 procedures. In addition, during the course of the study and based on Investigator's opinion, analysis of the safety laboratory samples collected at Day 0 may be performed to re-assess baseline.

Screening session may be performed in more than one visit; any extra visit will be considered as unscheduled visit and subjects will not be immunized until the eligibility is confirmed by the Investigator.

On Day 0, prior to immunization procedures, a urine pregnancy test (on all females) and vital signs measurements will be performed (refer to Section 5.2.2 for complete details on procedures to be performed). After confirmation of the subjects' continued eligibility by Investigator, the subjects will be randomized. Blood samples for the assessment of the immune response (HI/MN) and for the isolation and analysis of PBMCs (only from 15 subjects per treatment group; applied to MUHC site only) will be collected and then the subjects will be immunized with the study vaccine.

The subjects will be administered either one of the 3 non-adjuvanted dose levels (15 µg, 30 µg, or 60 µg per strain) or one of the 2 adjuvanted dose levels (7.5 µg or 15 µg per strain) or the placebo. All subjects (regardless of treatment) will receive the test article intramuscularly. Dose levels to be used in this study are presented below in Table 9.

Table 9: Dose levels to be used in this phase 2 trial (Immunization on Day 0)

Test Material	***Treatment Group (Grp)***	***Dose Level***	***No. of Subjects to be dosed***
VLP Quadrivalent Influenza vaccine	Grp1	15 µg per strain	75
VLP Quadrivalent Influenza vaccine	Grp 2	30 µg per strain	75
VLP Quadrivalent Influenza vaccine	Grp3	60 µg per strain	75
VLP Quadrivalent Influenza vaccine	Grp4	7.5 µg per strain + Alhydrogel® 0.4%	75
VLP Quadrivalent Influenza vaccine	Grp 5	15 µg per strain + Alhydrogel® 0.4%	75
Placebo	Grp 6	Not applicable; 100mM phosphate buffer + 150 mM NaCl + 0.01% Tween 80	75

Thereafter, subjects will be observed for at least 30 minutes after immunization for any signs and symptoms of local and systemic intolerance to the study vaccine. Any unusual signs or symptoms reported during the initial 30-minute of observation period will be promptly monitored closely.

Vital signs will be measured within 10 minutes after the 30-minutes observation period and hourly thereafter, if deemed necessary by the Investigator. During the 30-minute observation period, subjects will be given a Memory Aid (MA; or diary card), a measuring device, and a digital thermometer and will be instructed on their use. Solicited local and systemic symptoms, as well as any unsolicited AEs and any concomitant medication intakes will be recorded by subjects in their MA for the 21 days following the administration of the study vaccine (i.e. from Day 0 post-vaccination through Day 21 visit). Depending on site's procedures, subjects might be asked to bring their MA with them to subsequent visits and/or for phone contacts.

Subjects will be contacted by clinical staff on Day 1 (approximately 24 hours post-vaccination) and Day 8 to collect data on any solicited or unsolicited events and to remind them to continue completing the MA up to the Day 21 visit.

Subjects will be required to return to the clinical site on Day 3 and on Day 21. Subjects will be asked about the occurrence of any new AEs and any changes in medication intakes; all such safety data will be recorded in the source data. Blood chemistry and haematology testing, a urinalysis, and a urine pregnancy test (on all females) will be performed at Day 3 visit. Blood samples for the assessment of the immune response (HI/MN) and for the isolation and analysis of PBMCs (only from 15 subjects per treatment group; at the MUHC site only) will also be collected at Day 21 visit. During the Day 21 visit, subjects will be provided a second MA and will be instructed to record any AEs and any concomitant medication intakes occurring from Day 21 to Day 201.

Considering that the immunizations for this study will occur prior to and/or at the beginning of the seasonal influenza vaccination campaign, Medicago (via clinical sites) wants to ensure that the subjects are offered the opportunity to receive a licensed influenza (flu) vaccine. Many of the subjects that will be enrolled are among those who routinely access annual vaccination. Therefore, the sites plan to offer one of the licensed flu vaccine currently recommended for elderly, as part of this protocol. All subjects who received the licensed flu vaccine at the clinical site will be contacted approximately 7 days after this immunization for safety follow-up. They will also be asked to come to the clinical site approximately 21 days after having received this vaccine for safety and immunogenicity assessment.

Following the Day 21 visit until the time of the final visit at Day 201 (regardless if the subject received the licensed vaccine or not), monthly phone calls to each subject will be performed in order to collect safety data such as new AEs/SAEs, NOCDs, new medications for AE/SAE and/or NOCD, subjects' hospitalizations, or unscheduled visit to the Investigator's sites (refer to Section 5.2.5, last bullet point for details).

Subjects will be required to return to the clinical site for the final visit on Day 201 (6-months follow-up period that applies to the investigational vaccine). Subjects will be asked about the occurrence of any AEs and any medication intakes; all such safety data will be recorded in the source data. Blood chemistry and haematology testing, a urinalysis, and blood samples for the assessment of the immune response (HI/MN) and for the isolation and analysis of PBMCs (only from 15 subjects per treatment group, at the MUHC site only) will also be collected.

Depending on site's procedures, subjects might be asked to bring their MA with them at the final visit (Day 201). At the time of this visit, all safety data will be recorded in source data.

The duration of the study is approximately 7-12 months, from screening procedures up to Day 201 visit (depending when subjects undergo screening session and Day 201 visit).

5.1.1 INCLUSION CRITERIA

The subjects enrolled in this study must meet all of the following inclusion criteria:

1) Male and female subjects, 50 years of age or older.
2) BMI of ≥18 and ≤32.
3) Give his/her consent to participate in this study (by signing the Study ICF). In the opinion of the Investigator, competence and willingness to provide written, informed consent for participation after reading the informed consent form. The subject must have adequate opportunity to discuss the study with an Investigator or qualified designee.
4) Healthy as judged by the Investigator or designee and determined by general physical examination, vital signs, clinical laboratory tests, and medical history conducted no more than 90 days prior to study vaccine administration. Subjects with a pre-existing chronic disease will be allowed to participate if the disease is stable and, according to the Investigator's judgment, the condition is unlikely confound the results of the study or pose additional risk to the subject by participating in the study. Stable disease is defined as no new onset of exacerbation of pre-existing chronic disease 6 months prior to immunization. Based on Investigator's judgment, a subject with more recent stabilisation of a disease may still be eligible.
5) Comprehension of the study requirements, expressed availability for the required study period, ability to attend scheduled visits, accessible by phone on a consistent basis.
6) If female, have a negative serum pregnancy test result at screening and negative urine pregnancy test on Day 0 prior to immunization.
7) Female of childbearing potential must use an effective method of contraception for 1 month prior to immunization and agrees to continue employing adequate birth control measures for at least 60 days post-immunization. Moreover, she must have no plan to become pregnant for at least 2 months post-immunization. Abstinent subjects should be asked what method(s) they would use, should their circumstances change, and subjects without a well-defined plan should be excluded.

   The following relationship or methods of contraception are considered to be effectives:

   - Hormonal contraceptives (e.g., injectable, topical [patch], estrogenic vaginal ring, etc.);
   - Intra-uterine device (IUD) with or without hormonal release;
   - Male partner using a condom plus spermicide or sterilized partner (at least 1 year prior to immunization);
   - History of credible abstinence (self-reported);
   - Heterosexual abstinence at least 60 days post-immunization;
   - Female partner.

8) Non-childbearing females defines as:
    - Surgically-sterile (defined as bilateral tubal ligation or hysterectomy performed more than 1 month prior to immunization);
    - Post-menopausal (absence of menses for 24 consecutive months and age consistent with natural cessation of ovulation).

5.1.2 EXCLUSION CRITERIA

The subjects enrolled in this study must not meet any one of the following exclusion criterion:

1) According to Investigator's opinion, presence of significant acute or chronic, uncontrolled medical or neuropsychiatric illness. "Uncontrolled" is defined as:
    a) Requiring a new medical or surgical treatment within one month prior to study vaccine administration which would interfere with vaccine evaluation or study completion (subject requiring medical or surgical treatment that would not interfere with evaluation [e.g., minor knee surgery, cataracts, etc.] would be eligible);
    b) Requiring a change in medication dosage in one month prior to study vaccine administration due to uncontrolled symptoms or drug toxicity (elective dosage adjustments in stable subjects are acceptable).
2) Any medical or neuropsychiatric condition or any history of excessive alcohol use or drug abuse which, in the Investigator's opinion, would render the subject unable to provide informed consent or unable to provide valid safety observations and reporting.
3) Any autoimmune disease or any confirmed or suspected immunosuppressive condition or immunodeficiency including history of human immunodeficiency virus (HIV) infection, Hepatitis B or C, or the presence of lymphoproliferative disease.
4) Administration of any vaccine (including any other influenza vaccine) within 30 days prior to study enrolment or planned administration within the period from the vaccination up to blood sampling at Day 21 or within 30 days prior to blood sampling at Day 201. Immunization on an emergency basis will be evaluated in a case-by-case by the Investigator.
5) Administration of any adjuvanted or investigational influenza vaccine within 1 year prior to study enrolment or planned administration prior to the end of this trial (Day 201). Administration of any 'standard', not adjuvanted influenza vaccine (e.g., live attenuated TIV/QIV vaccine IN or split TIV/QIV vaccine by either intra-dermal or IM route) prior to the 30-day exclusion period mentioned above would be acceptable.
6) Use of any investigational or non-registered product within 30 days prior to study enrolment or planned use during the study period. Subjects may not participate in any other investigational or marketed drug study while participating in this study (approximately 7-12 months [depending when subjects undergo screening session and Day 201 visit]).

7) Treatment with systemic glucocorticoids at a dose exceeding 10 mg of prednisone per day, or equivalent for more than 7 consecutive days or for 10 or more days in total, within one month of study vaccine administration, any other cytotoxic or immunosuppressant drug, or any immunoglobulin preparation within 3 months of vaccination. Routine use of standard doses of nasal or inhaled glucocorticoids is allowed.
8) Any significant disorder of coagulation or treatment with warfarin derivatives or heparin. Persons receiving prophylactic anti-platelet medications, e.g., low-dose aspirin (no more than 325 mg/day), and without a clinically apparent bleeding tendency are eligible. Subjects treated with new generation drugs that will not increase risk of intramuscular bleeding (such as clopidogrel) are also eligible.
9) History of allergy to any of the constituents of the VLP quadrivalent (including H1N1, H3N2, B/Bris and B/Mass) study vaccine, Alhydrogel® (aluminum hydroxide) or to the PBS (used as placebo), or tobacco allergy.
10) History of anaphylaxis reaction to any food, medication or bee sting.
11) Any history of severe asthma (e.g., status asthmaticus, hospitalization for asthma control) or recurrent asthma episodes requiring medical attention in the last 3 years (≥ 1 episode/year).
12) Continuous use of anti-histamines in the last 4 weeks prior to immunization or use of anti-histamines 48 hours prior to study immunization.
13) Have a rash, dermatological condition, tattoos, muscle mass, or any other abnormalities at injection site which may interfere with injection site reaction rating.
14) Have received a blood transfusion within 90 days prior to study vaccination.
15) If female, have a positive or doubtful pregnancy test prior to immunization or lactating females.
16) Vital sign abnormalities: systolic blood pressure ≥150 mmHg and/or diastolic blood pressure ≥90mmHg, heart rate ≤40 beats/min and ≥100 beats/min. Although a vital signs measurement is out of the acceptable ranges, a subject may be included in the study based on Investigator's judgment. Presence of any febrile illness (including oral temperature (OT) ≥38.0˚C within 24 hours prior to immunization). Such subjects may be re-evaluated for enrolment after resolution of illness.
17) Cancer or treatment for cancer within 3 years of study vaccine administration. Persons with a history of cancer who are disease-free without treatment for 3 years or more are eligible. Persons with treated and uncomplicated basal cell carcinoma of the skin are eligible. Person with non-treated, non-disseminated local prostate cancer are eligible.
18) Identified as an Investigator or employee of the Investigator or study center with direct involvement in the proposed study, or identified as an immediate family member (i.e., parent, spouse, natural or adopted child) of the Investigator or employee with direct involvement in the proposed study.

5.2 Study Visit Procedures

5.2.1 SCREENING PROCEDURES (UP TO 90 DAYS PRIOR TO STUDY VACCINATION)

The following procedures will be included during screening session (could be performed in more than one visit meaning that any extra visit will be considered as unscheduled visit). Depending on the method of screening, some of these items may be performed at Day 0:

- Interview subjects (over the phone) in regard to a potential interest to participate in a study on Influenza vaccine. Invite interested subjects to come to the clinical site to participate in a screening session. Inform the subject on the nature and scope of the screening visit, potential risks and benefits of participation (to be done by the Investigator or his/her designee). In addition, inform the subjects on specific study procedures and answer all questions prior to requesting the subject's signature on the ICF. Subject's consent must be obtained prior to performing any procedures. Source documentation of the ICF being signed prior to any screening-related procedures being performed is necessary and a copy of the ICF must be provided to the subject.
- In case the screening session is performed more than 30 days prior to subject immunization and/or if REB approval is not received for the study at time of screening session, subjects will be requested to sign an ICF under a general pre-study protocol in addition to the Study ICF (at Day 0); otherwise, the current protocol will be used and subjects will be requested to sign only the Study ICF.
- Collect and review demographics (gender, date of birth, age, race, and ethnicity) and body measurements (BMI, weight, and height) data.

  Note: BMI is to be calculated as follows:

  Metric: weight (kg)/height $(m)^2$
  Non-metric: weight (lb)/height $(in)^2$ X 703.

  BMI will be rounded to nearest whole number using the standard convention:

  0.1-0.4 round down and 0.5-0.9 round up.
- Collect and review medical history. The medical history should record significant problems active at the time of screening and within the prior year. Problems that have been clinically inactive within the prior year, but which might alter the subject's current or future medical management, should also be noted (e.g., known mitral valve prolapse or history of seizure disorder).
- Review and record current and previous (up to 30 days prior immunization day) medication use.
- Record influenza immunizations received within 24 months prior to the administration of study vaccine and record any adverse reactions following these immunizations.
- Perform vital signs measurements including resting blood pressure (BP), heart rate (HR), and oral temperature (OT should be collected in ºC format; the measurement should be collect not following a recent hot or cold beverage or following smoking). BP will be taken after the subject has been in a seated position for at least 3 minutes. BP may be repeated one time following a 5-minute resting

period in a seated position, if judged necessary. All values are to be recorded in the source data including the reason for repeating. Inclusion of subjects in regards to out-of-range BP and HR measurement will be based on Investigator's judgment.

- Perform a physical examination of all relevant body systems, including general appearance, head and neck, lungs, heart and abdomen, extremities, skin and nervous system as well as a directed examination of the neck and axilla lymph nodes. These examinations should be performed by the Investigator (or a Sub-Investigator designated by the Investigator). If this physical examination cannot be performed during screening session, perform this physical examination prior to immunization at Day 0 instead of a history/symptom-directed physical examination.
- Collect blood samples (approximately 20 mL) for biochemistry, haematology, and serology (screen for HIV and Hepatitis B and Hepatitis C testing) analysis.
- Collect a urine sample for urinalysis.
- Perform serum pregnancy testing for all females.
- Review of inclusion and exclusion criteria and determine subject's eligibility to participate in this study. If the screening session is performed more than 30 days prior to subjects' immunization, this can be assessed during a phone contact or at Day 0 prior to immunization and then the next visit for the subjects eligible will be scheduled (Day 0).
- Subjects will be encouraged not to take any over-the-counter (OTC) antipyretics (such as acetaminophen, aspirin, paracetamol, naproxen, or ibuprofen) from 24 hours prior to immunization visit (Day 0).

5.2.2 DOSING VISIT (DAY 0)

Emergency equipment must be available on site and appropriate treatment must be instituted as soon as possible in the event of anaphylaxis or any other immediate hypersensitivity reaction. The Investigator (or a Sub-Investigator designated by the Investigator) will be on site for vaccine administration and remain for at least 30 minutes (and more if deemed necessary) after administration of the vaccine to the last subject. The Investigator (or a Sub-Investigator designated by the Investigator) will be on call for the remainder of the study. If necessary, a physician should be immediately available at the clinical site to administer treatment or to apply procedures for any immediate AEs/SAEs.

In case the screening session is performed using a separate screening ICF under a general pre-study protocol, the following procedures will be performed at the dosing visit prior to immunization (Day 0; immunization day):

- Review and Sign the Study Informed Consent Form (ICF). The Investigator or his/her designee, will fully inform the subject of the nature and scope of the study, potential risks and benefits of participation, the study procedures involved and will answer all questions prior to requesting the subject's signature on the ICF. Subject's consent must be obtained prior to performing any study-related procedures. Source documentation of the ICF being signed prior to any study-related procedures being performed is necessary and a copy of the ICF must be provided to the subject.
- If the screening session is performed more than 30 days prior to subject immunization, a blood sample will be collected for safety analysis. This extra

sample will be stored. If changes in medical status, physical examination, or vital signs occurred between screening session and Day 0 visit and are judged significant by the Investigator; then, this extra sample will be tested for routine safety labs. In this case, the subject's enrolment will be postponed until laboratory results are reviewed, found acceptable by the Investigator and the subject will be allowed to participate in Day 0 procedures. In addition, during the course of the study and based on Investigator's opinion, analysis of the safety laboratory samples collected at Day 0 may be performed to re-assess baseline.

The following procedures will be performed at the dosing visit prior to immunization (Day 0; immunization day):

- Record updates in medical history and medications and confirm that the subjects meet all inclusion criteria and none of the exclusion criterion since screening.
- Perform a lymphadenopathy examination only if subject complains of arm and/or shoulder pain (a directed exam for lymph node swelling of the neck and axilla), to obtain a baseline.
- A history/symptom-directed physical examination of all relevant body systems could also be performed based on Investigator's opinion. In case the physical examination of all relevant body systems cannot be performed during screening session, perform a physical examination of all relevant body systems, including lymphadenopathy instead of a history/symptom-directed physical examination during this visit.

  These examinations should be performed by the Investigator (or a Sub-Investigator designated by the Investigator).
- Measurement of vital signs (including BP, HR, OT, and respiratory rate [RR]). BP will be taken after the subject has been in a seated position for at least 3 minutes. OT will be collected using a digital thermometer (in ºC). Inclusion of subjects in regards of BP and HR or RR results will be based on Investigator's judgment.
- Review BMI. For this visit, only the weight will be measured (height will be obtained from result in the screening visit). Refer to Section 5.2.1, bullet point 2, for formula to calculate BMI).
- Collect a urine sample for all female subjects and perform urine pregnancy testing. No study vaccine must be administered until a negative result is obtained and documented.
- Once eligibility is confirmed, the subject will be randomized to a treatment group or placebo (refer to Section 6.6). The assigned randomization number will be documented on source data.
- Collect blood samples (serology, approximate 10 mL) for HI/MN titer assessment.
- Collect blood samples (approximately 40 mL) on 15 subjects per treatment group for cellular immune assays (PBMC) applied to MUHC site, only.

Once all pre-vaccination procedures are completed, the study vaccine will be administered intramuscularly (IM) into the deltoid muscle of the non-dominant arm (if possible) using a 25 gauge needle of sufficient length to reach the substance of the muscle based on the subject's weight/BMI (at least 1 inch or 2.5 cm or longer; for subjects with BMI over than 30, the use of a 1.5 inch or 3.8 cm is recommended). In

order to prevent confounding reaction around immunization site, whenever possible blood samples will not be collected from the same arm as the one used for immunization. The arms for blood collection and immunization will be documented.

5.2.2.1 DAY 0, MINIMUM OF 30 MINUTES POST-DOSE OBSERVATION

The following procedures will be performed at the dosing visit after immunization (Day 0; immunization day):

- Subjects will remain in the clinic for at least 30 minutes post-vaccination. The observation period will include an assessment of solicited local and systemic reactions. Any unusual signs or symptoms reported during the initial 30 minutes of observation will prompt continued close monitoring. Based on their condition, subjects may be asked to remain in the clinic for more than 30 minutes after the immunization (reason will be recorded in source data). All data (including assessment of solicited local signs and systemic symptoms) will be recorded in the source data during and after the post-observation period, when applicable. Refer to Section 8.1.1 for Assessment of AE and/or local and systemic reactions.
- During the observation period, subjects will be provided a Memory Aid (MA, or diary card), a measurement device template (in mm) for measuring solicited local reactions, and an oral digital thermometer for recording daily temperature (in ºC). Clinical sites will be responsible to purchase thermometers (in ºC) and measurement devices (in mm) for subjects. The clinic staff will also provide to subjects training on how to use adequately these tools. The subjects will be:
    - Instructed on how to collect their OT with the digital thermometer (in ºC) supplied to them for purposes of the study. From the evening of Day 0 to Day 7 following vaccination, the subject's OT will be taken at approximately the same time each evening and the findings will be recorded in the MA. Subjects will be asked to take their temperature at any other time if they feel feverish and to record the highest temperature of the day in the MA. In which case, they are allowed take OTC antipyretics (e.g., acetaminophen, aspirin, paracetamol, naproxen, or ibuprofen) and will be advised to increase the frequency of their temperature measurements to approximately every four (4) hours, until no longer febrile (defined as a temperature of ≥38.0˚C). Medication intake will be documented in MA and transcribed in source data.
    - Instructed on how to measure any local symptoms including erythema (redness) and swelling diameter at the injection site using the measurement template supplied to them for purposes of the study. Subjects will be also requested to evaluate pain at the injection site. Local symptoms will be assessed every day starting the evening of Day 0 up to the evening of Day 7 (post-immunization) and recorded on the MA.
    - Instructed to grade, on a daily basis from the evening of Day 0 through the evening of Day 7 following vaccination, each of the solicited systemic symptoms.

The severity of local and systemic symptoms or any AEs will be graded according to the FDA Guidance for industry[18].

Table 10 below presents different severity grades to be used in this study.

Table 10: Severity grade to be used for Local and Systemic Symptoms and other AEs

Symptoms	Severity				
	None	Grade 1 (Mild)	Grade 2 (Moderate)	Grade 3 (Severe)	Grade 4 (Potential life-threatening)
**Injection site reactions (local reactions)**					
**Erythema (redness)**	Less than 25 mm	Between 25mm and 50 mm	Between 51 and 100 mm	More than 100 mm	Necrosis or exfoliative dermatitis
**Swelling**	Less than 25 mm	Between 25mm and 50 mm and does not interfere with activity	Between 51 and 100 mm or interfere with activity	More than 100 mm or prevents daily activity	Necrosis
**Pain**	None	Does not interfere with activity	Repeated use of non-narcotic pain reliever >24 hours or interferes with activity	Any use of narcotic pain reliever or prevents daily activity	Results in a visit to emergency room (ER) or hospitalization
**Solicited systemic reaction of other symptoms**					
**Fever (ºC)**	<38.0ºC	≥38.0 and ≤38.4ºC	≥38.5 and ≤38.9ºC	≥ 39.0 and ≤40.0 ºC	>40.0ºC
**Headache**	None	No interference with activity	Repeated use of non-narcotic pain reliever >24 hours or some interferes with activity	Significant; any use of narcotic pain reliever or prevents daily activity	Results in a visit to emergency room (ER) or hospitalization
**Fatigue or Myalgia**	None	No interference with activity	Some interference with activity	Significant; prevents daily activity	Results in a visit to emergency room (ER) or hospitalization
**Nausea/ Vomiting**	None	No interference with activity or 1-2 episodes/24 hrs	Some interference with activity or >2 episodes/24 hrs	Prevents daily activity, requires outpatient IV hydration	Results in a visit to emergency room (ER) or hospitalization for hypotensive shock
**Diarrhea**	None	2-3 loose stools/ <400gms/24 hrs	4-5 stools or 400-800gms/24 hrs	6 or more watery stools or >800gms /24 hrs or requires outpatient IV hydration	Results in a visit to emergency room (ER) or hospitalization
**Other illness (than listed above)**	None	No interference with activity	Some interference with activity not requiring medical intervention	Prevents daily activity and requires medical intervention	Results in a visit to emergency room (ER) or hospitalization

- Instructed to examine swelling in the neck and axilla and record any feeling and/or swelling in their MA.
- Requested to record their individual data in their MA, as described above.
- Requested to use their MA to support their recall in providing information during the scheduled phone interview conducted on Days 1 and 8 following vaccination.
- Advised that they will be asked about the occurrence of any symptoms or events and the use of concomitant medication during the 21-day post-vaccination period as well as throughout the 6-month follow-up period.
- Depending on site's procedures, subjects will be informed that they might be asked to bring their MA to each clinic visit. The staff would review the MA entries and grade entries with the subject.
- Advised that if they experience unsolicited AEs and/or solicited local and systemic severe reactions (grade 3), they will have to report it promptly (as soon as possible) to the clinical site staff (refer to MA for emergency contact). The Investigator should be informed within 48 hours after the knowledge by the clinical site and may request the subject come to the clinic for evaluation.
- Advised on emergency contact information and instructions for contacting study personnel. Subjects will be advised to immediately contact the Investigator (or his designee), in the event of a SAE or medical emergency. Subjects will be provided with a phone contact number and be instructed to call if any reaction to vaccination is significant or of concern.
- Advised to notify their health care professional(s) (e.g., primary care physician) that they are participating in a clinical research study of an influenza vaccine.

- Measurement of vital signs (including BP, HR, RR, and OT). BP will be taken after the 30-minute observation period with the subject in a seated position for at least 3 minutes. Any out-of-range measurements will be assessed by the Investigator (or his designee) and any further action will be determined upon his medical decision.
- Provide appointments (date and time) for the next planned visits to the clinical site (Days 3 and 21) and also for the Day 8 phone contact.
- Subjects will be released from the clinical site once all post-vaccination procedures are completed and the subjects have a stable condition.

5.2.3 DAYS 1 AND 8 FOLLOWING VACCINATION PHONE CONTACT (ACCEPTABLE INTERVAL FOR DAY 1: +1 DAY; FOR DAY 8:-1 DAY/+2 DAYS)

The following procedures will be performed during the phone contacts:

- Subjects will be asked to use their MA to support their recall in providing information during the scheduled phone interview conducted on Days 1 and 8 following vaccination.

Unsolicited AEs and/or solicited local and systemic reactions of greater than Grade 2 (moderate), they will have to report it promptly (as soon as possible) to the clinical site staff (refer to MA for emergency contact). The Investigator should be informed within 48 hours after the knowledge by the clinical site and may request the subject come to the clinic for evaluation. The Investigator will document review of these events on the source data.

If any of the solicited local or systemic symptoms persist beyond Day 7, these will also be recorded as an AE. In this case, the AE start will be set as 168 hours (7 days) post-vaccination. The subject will be requested to note when the AEs resolved and report this information to the Investigator or clinic staff at the next visit to the clinical site or contact.

- Subjects will also be asked about any difficulties in completing the MA, any change in health, any visits to health care facilities, and/or medical practitioners and use of any concomitant medications.
- Subjects will be reminded of their next appointment (date and time) for the clinical visit and/or the next phone contact.
- Especially at the Day 1 phone contact, subjects will again be instructed to take and record their OT on a daily basis (approximately the same time each evening) during the 7-day safety period and the findings will be recorded in the MA. Subjects will be asked to take their temperature at any other time if they feel feverish (defined as a temperature of ≥38.0ºC) and to record the highest temperature of the day in the MA. In which case, they are allowed take OTC antipyretics (e.g., acetaminophen, aspirin, paracetamol, naproxen, or ibuprofen) and will be advised to increase the frequency of their temperature measurements to approximately every four (4) hours, until no longer febrile.

NOTE: As previously mentioned in this protocol, a maximum of -1 day to +2 days from scheduled visit to the clinic is allowed for the phone contact on Day 8. The subjects will be instructed to perform procedures (refer to Section 5.2.2.1) approximately at the same time each evening from the evening of Day 0 up to the evening of Day 7 (post-immunization). However, the subjects could be reached prior the Day 7 evening, due to the -1 day window. If a subject is reached on Day 7, the phone contact should be performed at least 168 hours after the subject's immunization. Moreover, any solicited or unsolicited signs and symptoms happening on Day 7 after the safety phone call should be recorded on the MA for that day. During the subsequent visit to the clinical site, the subject will be questioned in regard to such events which could have occurred since last contact.

In case the subject cannot be reached via phone, he could be contacted by text message or via email (if these contacts are available). However, the phone should be the initial and preferred way of communication.

5.2.4 DAY 3 BLOOD COLLECTION (ACCEPTABLE INTERVAL -1 DAY/+2 DAYS)

The following procedures will be performed during the safety visit on Day 3:

- Record updates on any change in subjects' health or complaints of lymphadenopathy or any significant visits to health care facilities and/or medical practitioners and use of any concomitant medications and confirm that the subject continued eligibility since last visit. As per site's procedures, subjects might be asked to bring their MA for that visit.
- Performed, based on Investigator's opinion, a lymphadenopathy examination only if subject complains of arm and/or shoulder pain (a directed exam for lymph node swelling of the neck and axilla). In addition, a history/symptom-directed physical examination of all relevant body systems could also be performed based on Investigator's opinion. This examination should be performed by the Investigator (or a Sub-Investigator designated by the Investigator).
- Perform urinalysis on all subjects.
- Collect blood samples (approximately 15 mL) for biochemistry and haematology.

  Any out of acceptable range clinical laboratory values considered to be a clinically significant change from baseline by the Investigator, will be repeated upon Investigator's request.
- Remind to subjects date and time of their Day 8 phone call and the Day 21 visit.

5.2.5 DAY 21 VISIT BLOOD COLLECTION (ACCEPTABLE INTERVAL: -2 DAYS/+ 3 DAYS)

The following procedures will be performed during the Day 21 visit:

- Record updates on any change in subjects' health or complaints of lymphadenopathy or any significant visits to health care facilities and/or medical practitioners and use of any concomitant medications and confirm that the subject continues to meet all inclusion criteria and none of the exclusion criterion since last visit.
- Perform a lymphadenopathy examination only if subject complains of arm and/or shoulder pain (a directed exam for lymph node swelling of the neck and axilla). In addition, a history/symptom-directed physical examination of all relevant body systems could also be performed based on Investigator's opinion. These examinations should be performed by the Investigator (or a Sub-Investigator designated by the Investigator).
- Measure vital signs (including BP, HR, OT, and RR). BP will be taken after the subject has been in a seated position for 3 minutes. OT will be collected using a digital thermometer (in ºC format).
- Perform urine pregnancy testing on all females.
- Collect blood samples (serology, approximate 10 mL) for HI/MN titer assessment.
- Collect blood samples (approximately 40 mL) on 15 subjects per treatment group for cellular immune assays (PBMC) applied to MUHC site, only.
- Administer licensed Influenza vaccine (according to the manufacturer) to subjects who chose to receive it. Depending on availability of the licensed vaccine, the

administration may be performed later. Licensed flu vaccine would be available at the clinical sites until mid-December.

All subjects who chose to receive the licensed flu vaccine at the clinical site will be contacted approximately 8 days after this immunization for safety follow-up. They will also be asked to come at the clinical site approximately 21 days after having received this vaccine for safety and immunogenicity assessment.

- Provide a MA to subjects, for collection of safety data from Day 21 to Day 201. Subjects will also be provided with a date and time to return to the clinic for the Day 201 follow-up visit.
- Perform monthly phone calls to collect AEs/SAEs and NOCDs following the Day 21 visit until the final visit at Day 201. Subjects should be reached once a month with no more than 45 days between phone contacts (use Day 21 date as starting reference). Subjects will be asked about any change in their health, any visits to health care facilities including hospitalizations or emergency room visits and/or medical practitioners, and use of any concomitant medications. The 6-month (180 days) follow up period will start after the Day 21 visit and will, therefore, be up to Day 201. In case the subject cannot be reached via phone, he could be contacted by text message or via email (if these contacts are available). However, the phone should be the initial and preferred way of communication.

NOTE: As previously mentioned in this protocol, a maximum of -2 days to +3 days from scheduled visit to the clinic is allowed for the Day 21 visit and -14 days to +80 days are allowed for the Day 201 visit. All effort will be made to have the subjects returned on the planned date for Day 21 activities. If for any reason the visit at Day 21 is done before or after this exact planned date, subsequent visit (Day 201) will be adjusted accordingly.

5.2.6 DAY ≥28; PHONE CONTACT 7 DAYS FOLLOWING VACCINATION WITH THE LICENSED FLU VACCINE (ACCEPTABLE INTERVAL ±4 DAYS)

The following procedures will be performed for all subjects who chose to receive the licensed flu vaccine at the clinical site:

- Subjects will be asked to use their MA to support their recall in providing information during the scheduled phone interview conducted on Days 28 following vaccination (7 days following the licensed flu vaccine administration).
- Record updates on any change in subjects' health (including any new AEs) or complaints of lymphadenopathy or any significant visits to health care facilities and/or medical practitioners and use of any concomitant medication since last visit.
- Subjects will be reminded of their next appointment (date and time) for the clinical visit and/or the next phone contact.
- Depending on site's procedures, subjects might be asked to bring their MA to support their recall in providing information during the next visit, as per internal site's procedures.

NOTE: This call is performed as a safety contact and will not replace the first month contact.

5.2.6 DAY ≥42; VISIT APPROXIMATELY 21 DAYS FOLLOWING VACCINATION WITH THE LICENSED VACCINE (ACCEPTABLE INTERVAL UP TO +56 DAYS FOLLOWING VACCINATION; OR DAY ≥42 +35DAYS WINDOW)

The following procedures will be performed for all subjects who chose to receive the licensed flu vaccine at the clinical site:

- Subjects could be asked to use their MA to support their recall in providing information during the visit, as per internal site's procedures.
- Record updates on any change in subjects' health or complaints of lymphadenopathy or any significant visits to health care facilities and/or medical practitioners and use of any concomitant medications and confirm that the subject continues to meet all inclusion criteria and none of the exclusion criterion since last visit.
- Perform a lymphadenopathy examination only if subject complains of arm and/or shoulder pain (a directed exam for lymph node swelling of the neck and axilla). In addition, a history/symptom-directed physical examination of all relevant body systems could also be performed based on Investigator's opinion. These examinations should be performed by the Investigator (or a Sub-Investigator designated by the Investigator).
- Measure vital signs (including BP, HR, OT, and RR). BP will be taken after the subject has been in a seated position for 3 minutes. OT will be collected using a digital thermometer (in ºC format).
- Collect blood samples (serology, approximate 10 mL) for HI/MN titer assessment.
- Subjects will be reminded of their next appointment (date and time) for the clinical visit and/or the next phone contact.
- Depending on site's procedures, subjects might be asked to bring their MA to support their recall in providing information during the next visit.

5.2.7 FINAL VISIT-DAY 201 (ACCEPTABLE INTERVAL IS -14 DAYS/+80 DAYS)

The following procedures will be performed during the final visit on Day 201 (6-months follow-up period that applies to the investigational vaccine):

- Record updates on any change in subjects' health, complaints of lymphadenopathy, or any significant visits to health care facilities and/or medical practitioners and use of any concomitant medication and confirm that the subject continues to meet all inclusion criteria and none of the exclusion criterion since last visit. Depending on site's procedures, subjects will be asked to bring their MA for that visit.
- Perform a lymphadenopathy examination only if subject complains of arm and/or shoulder pain (a directed exam for lymph node swelling of the neck and axilla). In addition, a history/symptom-directed physical examination of all relevant body systems could also be performed based on Investigator's opinion. These examinations should be performed by the Investigator (or a Sub-Investigator designated by the Investigator).
- Measure vital signs (including BP, HR, OT, and RR). BP will be taken after the subject has been in a seated position for 3 minutes. OT will be collected using a digital thermometer (in ºC format).

- Collect blood samples (approximately 15 mL) for biochemistry and haematology.

  Any out of acceptable range clinical laboratory values considered to be a clinically significant change from baseline by the Investigator, will be repeated upon Investigator's request.
- Perform urinalysis on all subjects.
- Collect blood samples (serology, approximate 10 mL) for HI/MN titer assessment to evaluate antibody persistence for approximately 6 months following vaccination.
- Collect blood samples (approximately 40 mL) on 15 subjects per treatment group for cellular immune assays (PBMC) applied to MUHC site, only.

5.3 Selection of the Study Population

5.3.1 RECRUITMENT PROCEDURES

Subjects enrolled in this study will be healthy subjects, 50 years of age or older and will be volunteer members of the community at large. Following IRB/REB approval potential subjects could be contacted and invited to participate in this study, if they meet all inclusion criteria and none of the exclusion criterion.

5.3.2 SUBJECT INFORMATION AND CONSENT

Voluntary written informed consent must be obtained from each subject prior to performing any study-related procedures (including screening procedures). Each subject should be given both verbal and written information describing the nature and duration of the clinical study. The informed consent process should take place under conditions where the subject has adequate time to consider the risks and benefits associated with his/her participation in the study. Subjects should not be screened until they have signed an approved ICF written in a language that is understandable to them.

The IRB/REB-approved ICF should be signed and dated by the subject and the Investigator (or their nominated designee) who conducted the informed consent discussion. The Investigator is responsible for assuring the appropriate content of the ICF and that an informed consent is obtained from each subject in accordance to all applicable regulations and guidelines. The original signed ICF should be retained in the Investigator's files. Each subject should receive a copy of the signed and dated written ICF along with any other written information provided to the subject.

The Investigator should maintain a log of all subjects who sign the ICF and indicate if the subject was randomized and received study vaccine or, if not, the reason why. The subject's medical records (source data) should also document that the ICF was signed and dated prior to any study-related screening procedures being performed.

5.3.3 TEMPORARY CONTRAINDICATIONS

The following conditions constitute temporary contraindications to administration of study vaccine:

- Temperature ≥38.0ºC within 24 hours prior to immunization.
- Acute cold symptoms such as minor upper respiratory tract infection (URTI) symptoms that typically resolve in 48-72 hours prior to immunization.

Such subjects may be re-evaluated for enrolment after resolution of illness (or the results deemed acceptable).

If any of the following AEs occur during the study, the subject may continue study procedures at the discretion of the Investigator, with the approbation of the Sponsor.

- Any confirmed or suspected immunosuppressive or immunodeficient condition, including HIV infection.
- Discovery of any health condition or a change in the subject's status which renders him/her unable to comply with protocol-mandated safety follow-up, based on the Investigator's opinion.
- Pregnancy: Subjects who become pregnant during the study will be followed for safety. The Investigator (or designee), will collect pregnancy information on any subject who becomes pregnant while participating in this study. The Investigator (or designee) will record pregnancy information on the Pregnancy Report Form (refer to Study Procedures Manual) and submit it to the Safety Contact (refer to Section 8.1.3) within 24 hours of learning of a subject's pregnancy. The subject will be followed to determine the outcome of the pregnancy. At the end of the pregnancy, whether that will be full-term or prematurely, information on the status of the mother and child will be forwarded to the Safety Contact. Generally, follow-up will be no longer than 6 to 8 weeks following the estimated delivery date. While pregnancy itself is not considered an AE/SAE, any pregnancy complication or elective termination of a pregnancy for medical reasons will be recorded as an AE or a SAE, and will be followed. A spontaneous abortion is always considered to be a SAE and will be reported. Furthermore, any SAE occurring as a result of a post-study pregnancy and considered reasonably related in time to receipt of the investigational product by the investigator, will be reported to the protocol safety contact. While the Investigator is not obligated to actively seek this information from former study subjects, he/she may learn of a pregnancy through spontaneous reporting. Information on pregnancies identified during the screening phase/prior to vaccine administration does not need to be collected; this information need not be communicated to the Sponsor.

The subject will be followed until resolution or stabilization of the event or if the results return to a normal baseline, based on Investigator's judgment.

5.3.4 REMOVAL OF SUBJECTS FROM TREATMENT OR ASSESSMENT

Subjects will be advised that they are free to withdraw from the study at any time without prejudice to their future medical care by the physician or the institution. Subjects who withdraw or are withdrawn from the study after immunization will not be replaced. The primary reason for withdrawal must be recorded in source data and captured in the CRF.

5.3.4.1 CONDITIONS FOR WITHDRAWAL

Every reasonable effort should be made to ensure that each subject complies with the protocol and completes all study visits. However, a subject may withdraw or be withdrawn from participation if:

- The subject withdraws consent,
- The subject displays non-compliance to the terms of their participation in the clinical study (based on Investigator's opinion);
- Safety reasons as judged by Data Safety Monitoring Board (DSMB), the Investigator and/or Medicago;
- Medicago or Health Canada terminates the clinical study.

In case of withdrawal due to a SAE, a subject will not be replaced. Drop-outs will be subjects who leave the study earlier than planned for whatever reason; drop-outs will not be replaced. Drop-outs who received the study medication will be asked to return to the clinical site for a final assessment. The procedures performed for the final assessment will comprise those of safety assessment done for Day 201. All drop-outs must be reported to Medicago. The reason for drop-out should be documented in the subjects' records and in the appropriate section of the CRF. The CRF must be completed up to and including the time of drop-out/final assessment.

5.3.4.2 LOST TO FOLLOW-UP PROCEDURES

Every attempt will be made to contact study subjects who are lost to follow-up. At least 3 contacts will be attempted and recorded in the source data. As a last resort, one registered letter requesting contact with the site will be sent to any subject with whom the clinic staff no longer has contact. Such contact will be documented in the subject's source data.

5.3.4.3 SCREEN FAILURES

Screen failures are subjects that have signed the study-specific ICF and are not eligible for enrolment (subjects who were not randomized), due to failure on one or more inclusion or exclusion criteria or because the subject withdrew consent prior to randomization.

Eligible/not treated subjects are subjects that have signed the ICF and are eligible for enrolment, but were either not randomized or randomized and not treated.

Tracking of both classes of screen failures will be carried out within the study records maintained within the participating clinical sites. Neither class of subjects will receive a safety follow-up.

5.3.4.4 FOLLOW-UP OF DISCONTINUATIONS

Subjects who receive a study vaccine will be followed for safety until the end of study (Day 201).

5.3.4.5 MEDICAL HISTORY

The medical history should record significant problems active at the time of screening and within the prior year. Problems that have been clinically inactive within the prior year, but which might alter the subject's current or future medical management, should also be noted (e.g., known mitral valve prolapsed or history of seizure disorder).

5.4 Modification of the Study and Protocol

Modifications to the protocol should be made as an amendment and should be approved both by Medicago and the IRB/REBs prior to being implemented unless the amendment is made to eliminate an immediate hazard to the clinical study subjects. Medicago shall be responsible for notifying the regulatory authority of any amendments to the protocol.

The Investigator is responsible for notifying the IRB/REBs of all protocol amendments and ensuring that the IRB/REBs have approved any amendment.

Note that administrative changes (e.g., change in monitor phone number) may be implemented without prior IRB/REBs approval; however, the Investigator must notify the IRB/REBs of any administrative change and ensure that IRB/REBs acknowledges receipt of the administrative changes.

5.5 Interruption of the Study

The Investigator (in consultation with the Sponsor), the Sponsor, or Regulatory Authorities may interrupt or terminate this study, for any reason judged valid. The Investigator will immediately, on discontinuance of the clinical study at the clinical site, inform both the clinical study subjects and the IRB/REBs responsible for the study of the discontinuance, provide them with reasons for the discontinuance and advise them in writing of any potential risks to the health of the clinical study subjects or other persons. It is the Sponsor's responsibility to report discontinuance of the study to the regulatory agencies, within the appropriate timeframe and providing them with the reasons for the discontinuance, and advise them in writing of any potential risks to the health of clinical study subjects or other persons. The Sponsor must then inform the Investigator that the appropriate notifications were made.

6. TREATMENTS

6.1 Vaccines Administered

The subjects will receive either one of the 3 non-adjuvanted doses of the study vaccine (i.e. 15 µg, 30 µg, or 60 µg per strain) or one of the 2 adjuvanted doses of the study vaccine (i.e. 7.5 µg or 15 µg per strain mixed with Alhydrogel® adjuvant) or a placebo as an intramuscular injection.

6.2 Identity of Investigational Product

The study vaccine is a VLP quadrivalent seasonal influenza vaccine composed of recombinant H1, H3, and 2 B proteins [hemagglutinins (HA)] from the 4 influenza strains recommended by WHO for vaccination in the Northern hemisphere in 2013-2014, assembled into VLPs and produced in a plant-based (*Nicotiana benthamiana*) transient expression system. The transfer vector used to insert the plasmids (containing the constructs for proteins of interest) into the plant for expression is a bacterium, *Agrobacterium tumefaciens*. The HA protein sequences utilized in the expression constructs were all obtained from viruses isolated from humans, not from egg or cell grown viruses.

Transient expression is a protein expression technology which does not require or use the stable integration process of a foreign gene into the host genome. Transient expression can be achieved by various means, but generally implies the use of a "transfer vector," bacterial, viral, or purely mechanical, which mediates the passage of genetic information in the form of a polynucleotide (in this example, a strand of DNA) into a host cell. Once in the cell, the genetic information is generally used directly as a template for transcription into mRNA molecules that are subsequently translated into a protein by the host cellular machinery. For further information refer to the Investigator Brochure (IB).

6.2.1 STUDY VACCINE COMPOSITION

The seasonal VLP Influenza vaccine is a clear liquid suspension consisting of a mix of 4 VLPs each bearing the HA of an approximate molecular weight of 72 kDa of one of the following virus strains A/California/7/2009 [H1N1], wild-type A/Victoria/361/2011 [H3N2], B/Brisbane/60/2008 (Victoria lineage) [B/Bris], or B/Massachusetts/2/2012 (Yamagata lineage) [B/Mass]), in a PBS (100 mM PO4, 150 mM NaCl, 0.01%Tween 80; pH 7-7.6).

The seasonal VLP Influenza Vaccine will be contained in a 2 mL borosilicate vial (Type 1+). The Hemagglutinin protein concentration in the vaccine vials provided will be 30 µg/mL per strain, in a volume of 0.7 mL per vaccine vial or 60 µg/mL per strain, in a volume of 1.2 mL per vaccine vial, which will be mixed with appropriate quantity of adjuvant (Alhydrogel® 0.4%), when applicable, for final dosages of 15 µg, 30 µg or 60 µg per strain without adjuvant or of 7.5µg or 15µg per strain + Alhydrogel® (0.5 mg Al per dose). Doses of 7.5 µg 15 µg, 30 µg and 60 µg per vaccine strain correspond to 30 µg, 60 µg, 120 µg, or 240 µg in total HA of seasonal VLP vaccine, respectively.

6.2.2 PREPARATION AND ADMINISTRATION OF STUDY VACCINE

Study vaccine or placebo will be preferably prepared by 2 staff member at the clinical site as described in the "Preparation of Investigational Study Vaccine/placebo" section of the Study Procedures Manual. Prepared study vaccine (or placebo) will subsequently be administered to subjects by an unblinded staff member.

> NOTE: The personnel responsible for drug preparation can also perform the vaccination (if certified and qualified to do it) as this person would already be unblinded. The unblinded staff members must not be involved in the evaluation of any AEs or reactogenicity evaluation of the subjects following immunization.

Vials with a vaccine concentration of 30 µg/mL per strain (volume of 0.7 mL per vial) or at a concentration of 60 µg/mL per strain (volume of 1.2 mL per vial) which will be mixed with appropriate quantity of adjuvant (when applicable) in a clean environment in an aseptic manner for preparation for injection as follows:

Non-adjuvanted doses:

- For the administration of the 15 µg vaccine dose without adjuvant, the vaccine vials that contain a volume of 0.7 mL of vaccine at concentrations of 30 µg/mL per strain, will be used to withdraw 0.5 mL (15 µg per strain of vaccine) to be administered to subjects.
- For the administration of the 30 µg vaccine dose without adjuvant, the vaccine vials that contain a volume of 1.2 mL of vaccine at concentrations of 60 µg/mL per strain, will be used to withdraw 0.5 mL (30 µg per strain of vaccine) to be administered to subjects.
- For the administration of the 60 µg vaccine dose without adjuvant, the vaccine vials that contain a volume of 1.2 mL of vaccine at concentrations of 60 µg/mL per strain, will be used to withdraw 1.0 mL (60 µg per strain of vaccine) to be administered to subjects.

Adjuvanted doses:

- For the administration of the 7.5 µg vaccine dose with adjuvant, a volume of 0.7 mL of Alhydrogel® 0.4% (from 1 Alhydrogel® vial containing 1.0 mL) will be added to the vaccine vials that contain a volume of 0.7 mL of vaccine at concentrations of 30 µg/mL per strain for a total volume of 1.4 mL, from which will be withdrawn 0.5 mL (7.5 µg per strain of vaccine) to be administered to subjects.
- For the administration of the 15 µg vaccine dose with adjuvant, a volume of 1.2 mL Alhydrogel® 0.4% (from 2 Alhydrogel® vials containing 1.0 mL) will be added to the vaccine vials that contain a volume of 1.2 mL at concentrations of 60 µg/mL per strain for a total volume of 2.4 mL, from which will be withdrawn 0.5 mL (15 µg per strain of vaccine) to be administered to subjects.

Refer to Table 11 for an overview of vaccine formulations.

Table 11: Vaccine formulations

Dose to be administered	Vaccine Formulation (Vials)				Volume withdrawn to be administered
	Starting concentration	Starting volume	Alhydrogel® volume to be added in the vaccine vials	Total volume	
**15 µg/strain**	30µg/mL	0.7 mL	Nothing	0.7 mL	0.5 mL
**30 µg/strain**	60µg/mL	1.2 mL	Nothing	1.2 mL	0.5 mL
**60 µg/strain**	60µg/mL	1.2 mL	Nothing	1.2 mL	1.0 mL
**7.5 µg/strain With adjuvant**	30µg/mL	0.7 mL	0.7 mL (from 1 vial of 1.0 mL)	1.4 mL	0.5 mL
**15 µg/strain With adjuvant**	60µg/mL	1.2 mL	1.2 mL (from 2 vials of 1.0 mL)	2.4 mL	0.5 mL

The needle used for the injection should be of sufficient length to reach the substance of the muscle. According to randomization scheme, subjects will receive 0.5 mL or 1.0 mL (depending on which vaccine strength to be administered) of the seasonal VLP influenza vaccine formulated with Alhydrogel when indicated, intramuscularly in the deltoid muscle, preferably in the non-dominant arm. Whenever possible, the injection will be given in the opposite arm from which blood samples are drawn.

Elastomer stoppers should never be removed from any vial. After withdrawal of the sufficient volume of formulated vaccine to deliver 0.5 mL or 1.0 mL, the remaining volume of formulated vaccine in each vial will be retained. The 0.5 mL or 1.0 mL vaccine dose administered will be recorded in the Study Drug Administration Record form (refer to Study Procedures Manual) by the clinical site, which will be separate from the study medication record for drug preparation. After drug accountability monitoring is completed, the Investigator (or designee) will return all study vaccines (including adjuvant vials)/placebo vials (used and unused vials) in accordance with instructions provided by the Sponsor (refer to Section 6.5.5 for details).

Some variations in the method for vaccine preparation may be used upon agreement by Sponsor but in any case, all vaccine preparation will be documented in appropriate form (refer to Study Procedures Manual). Also, further specific information relating to vaccine or placebo preparation, storage, and shipment will be presented in the Study Procedures Manual.

6.2.3 PRECAUTIONS FOR USE

The study vaccine should be stored at 2ºC to 8ºC but should be at room temperature before administration (should not be administered cold).

Do not freeze vaccine as this destroys activity. Do not use vaccine that has been frozen.

DO NOT SHAKE the vaccine preparations. Do not vortex vaccine.

Study vaccine must not be administered intravenously.

6.2.4 DOSE SELECTION AND TIMING

This study will be a dose escalation, cohort staggering (slow enrolment) for 3 non-adjuvanted dose levels (15 µg, 30 µg, and 60 µg/strain) and for the 2 adjuvanted dose levels (7.5 µg and 15 µg/strain) with a placebo-controlled group divided in 3 cohorts.

Cohort 1: Approximately 138 subjects will be randomized and dosed as follows:

- 75 subjects with the 15 µg/strain non-adjuvanted dose (100% of this treatment group),
- 19 subjects with 30 µg/strain non-adjuvanted dose (25% of this treatment group),
- 19 subjects with 7.5 µg/strain adjuvanted dose (25% of this treatment group),
- 25 subjects with a placebo (33% of placebo group).

The 7-day safety data after the immunization will be collected by the clinical staff and will be reviewed by the Data and Safety Monitoring Board (DSMB). The DSMB consisting of the PIs, the Sponsor's Medical Officer and 3 external medical advisors (voting members) (refer to Section 3); the members will determine if the clinical sites are allowed to continue with the immunization of the second cohort.

Cohort 2: Approximately 175 subjects will be randomized and dosed as follows:

- 56 subjects with the 30 µg/strain non-adjuvanted dose (75% of this treatment group),
- 56 subjects with the 7.5 µg/strain adjuvanted dose (75% of this treatment group),
- 19 subjects with the 15 µg/strain adjuvanted dose (25% of this treatment group),
- 19 subjects with the 60 µg/strain non-adjuvanted dose (25% of this treatment group),
- 25 subjects with a placebo (33% of placebo group).

The 7-day safety data after the immunization will be collected by the clinical staff and reviewed by the DSMB prior to allowing immunization of the third cohort.

Cohort 3: Approximately 137 subjects will be randomized and dosed as follows:

- 56 subjects with the 15 µg/strain adjuvanted dose (75% of this treatment group),
- 56 subjects with the 60 µg/strain non-adjuvanted dose (75% of this treatment group),
- 25 subjects with a placebo (33% of placebo group).

The Seasonal VLP Influenza Vaccine lot numbers are defined below:

Vaccine concentration of 30µg/mL: Lot number: AAHAW01-140016-012

Vaccine concentration of 60µg/mL: Lot number: AAHAW01-140015-011

6.3 Identity and Composition of the Control Product

The control product (placebo–PBS) will consist of a 100 mM phosphate buffer + 150 mMNaCl + 0.01% Tween 80.

Placebo: Lot number: PPPXX01-130005-014

6.3.1 PREPARATION AND ADMINISTRATION

The control product or PBS (labeled placebo) will be contained in a 2 mL borosilicate vial (Type 1) consisting of a volume of 0.75 mL of placebo per vial. For placebo administration, 0.75 mL vials will be used to withdraw 0.5 mL for injection in subjects. Exact volume of placebo administered will be recorded by the clinical site on the Study Drug Administration Record form (refer to Study Procedures Manual), which will be separate from the study medication's record for drug preparation. After drug accountability monitoring is completed, the Investigator (or designee) will return all study vaccines (including adjuvant vials)/placebo vials (used and unused vials) in accordance with instructions provided by the Sponsor (refer to Section 6.5.5 for details).

NOTE: Although some differences can be noticed in the appearance of the different formulations to be injected, the randomization of each subject to treatment or placebo will be maintained blinded. Thus, the site staff involved in the preparation/injection of the

vaccine will not be involved in any activity, such as safety evaluation, where a bias could occur. Moreover, as the Investigator will be aware of the aspect of the vaccine formulations, he will not be involved and will not witness vaccine injection; however, the Investigator will be available on study site. Refer to the Study Procedure Manual.

6.3.2 PRECAUTIONS FOR USE

The study vaccine should be stored at 2ºC to 8ºC but should be at room temperature before administration (should not be administered cold).

6.4 Identity and Composition of Other Products used in this Study

Adjuvant: The adjuvant to be used for mixing with vaccine (when required) in this study is Alhydrogel® 0.4%

Adjuvant: Alhydrogel®: Lot number:AAIXX01-130012-013

6.4.1 PREPARATION AND ADMINISTRATION

Adjuvant vials consist in a 1 mL vial. For formulation of the adjuvanted vaccine (7.5 µg and 15 µg/strain doses), 0.7 mL or 1.2 mL of adjuvant will be withdrawn and added in the appropriate vaccine vials, to formulate for administration of 7.5 µg and the 15 µg/strain VLP vaccine, respectively. Exact volume of adjuvant used will be recorded by the clinical site on the Study Drug Administration Record form (refer to Study Procedures Manual), which will be separate from the study medication's record for drug preparation. After drug accountability monitoring is completed, the Investigator (or designee) will return all study vaccines (including adjuvant) vials)/placebo vials (used and unused vials) in accordance with instructions provided by the Sponsor (refer to Section 6.5.5 for details).

The needle on the syringe containing the Q13 VLP influenza vaccine combined with Alhydrogel® 0.4% should be changed prior to administration to the subject. A separate sterile syringe and needle should be used for each injection to prevent transmission of any infectious agents.

6.4.2 PRECAUTIONS FOR USE

The adjuvant should be stored at room temperature (between 20 to 25ºC) before usage for formulation of the vaccine (formulated vaccine should not be administered cold).

6.5 Product Logistics

6.5.1 LABELING AND PACKAGING

It is the responsibility of the Sponsor to ensure that supplies provided for this study are manufactured under Good Manufacturing Practices (GMP) and are suitable for human use. It is also the responsibility of the Sponsor to ship a sufficient amount of dosage units to allow the clinical site to maintain an appropriate supply for the study.

The study vaccine, placebo, and adjuvant will be packaged and labeled at Medicago in accordance with all applicable regulatory requirements. All products will be placed in secondary packaging and appropriately labeled.

6.5.2 STORAGE AND SHIPMENT CONDITIONS

The study vaccine, the placebo and adjuvant vials will be shipped to the clinical sites. This shipment will also contain: a shipment requisition, preparation and expedition form, as well as an electronic temperature monitor device (as example, TempTale®).

At arrival to the clinical site, vaccines and placebo vials will be immediately placed in a secured and monitored refrigerator which has been stabilized at a temperature of 2 to 8 ºC. Adjuvant will be placed in a secured and monitored room at 20-25ºC.

It is requested that the temperature monitor device be stopped upon receipt of the vaccine and the exact date and time is recorded on the device form. The device could be returned to Medicago or alternatively, World Courier that will be used as the transporter could take care of the temperature device, reading of temperature and the transfer of the data to Sponsor.

A written authorization per shipment will be required from the Sponsor contact (refer to Section 3 for details) prior to administering the study vaccine.

A manual log and calibrated thermometer or other temperature recording device will be used to document refrigerator temperature from the time of vaccine reception to the clinic up to the dosing period (up to the last subject dosed). This documentation will be maintained and made available to the Sponsor or designee for review. The site should ensure that appropriate back-up procedures are established for maintenance of the refrigerator temperatures, in the event of a power failure. Such procedures may include an alarm system, back-up electrical system/generator, or a documented contingency plan for maintaining proper refrigeration.

In the event the refrigerator deviates from the 2ºC to 8 ºC required temperature range, the clinical site staff will follow instruction provided in the Study Procedures Manual.

The site should maintain procedures relating to cold-chain maintenance. The Investigator has overall responsibility for ensuring that study vaccines are stored in a safe, locked with limited access location under the specified appropriate storage conditions. Responsibility may be delegated to a nominated member of the study staff, but this delegation must be documented.

6.5.3 PRODUCT ACCOUNTABILITY

The study vaccines (including adjuvant vials) and placebo must be used only as directed in the protocol and in Study Procedures Manual. On appropriate form, accountability will be performed for the vaccines (including adjuvant vials) and placebo used during the course of the study and also for the remaining study medication (unused). Written records are maintained for receipt and disposition of all vaccines received from the Sponsor.

6.5.4 Replacement Doses

Study vaccine (including the adjuvant vials) and placebo replacement doses will be shipped according to dose level and may be used in the event of breakage or accidental loss while being prepared. All instances must be documented on the appropriate form (refer to Study Procedure Manual).

6.5.5 RETURN OF UNUSED PRODUCTS

The Investigator (or designee) will sign all study vaccine/placebo accountability forms at the end of the vaccination period. Used and unused study vaccine (including adjuvant vials)/placebo vials must be retained until the unblinded study monitor (designated by Medicago) completes reconciliation of test article delivery records with disposition records and accountability of used and unused vials. All study vaccines (including adjuvant) vials)/placebo vials must be accounted for and all discrepancies documented accurately. After drug accountability monitoring is completed, the Investigator (or designee) will return all study vaccines (including adjuvant vials)/placebo vials (used and unused vials) to the address below-mentioned in accordance with instructions provided by the Sponsor:

Medicago R&D Inc.
3220, rue Watt, suite 104
Quebec, Quebec, Canada, G1X 4Z6
Tel.: 1.418.658.9393 ext. 321
E-mail: mercierd@medicago.com
Fax: 1.418.658.6699
Attn: M. Daniel Mercier (Logistic Coordinator)

6.6 Randomization/Allocation Procedures

The sites will be provided a pre-determined sequence of randomization numbers according to the randomization code. Randomization will be performed using random block permutations obtained with the PLAN procedure of SAS®. A complete description of the randomization procedure will be described in a Randomization Plan.

Potential study subjects will be screened. Once all screening procedures including Day 0 pre-randomization procedures are completed and the study eligibility is confirmed, the randomization numbers of the pre-determined sequence will be allocated sequentially to subjects within the appropriate treatment group. Once a randomization number has been assigned, it will not be re-used in any event. No subjects will be entered into the study more than once. If a randomization number has been allocated incorrectly, no attempt will be made to remedy the error once study vaccine has been dispensed. The subject will continue with the randomization number and study vaccine. The study staff will notify the Sponsor as soon as the error is discovered without disclosing the study vaccine administered. Admission of subsequent eligible subjects will continue using the next unallocated number in the sequence.

This randomization number and treatment will be recorded along with the screening number for each subject on the Investigational Product and Accountability Log (refer to Study Procedures Manual).

If a subject receives a randomization number but is not vaccinated, the following subjects in the assignment group will be randomized with the next randomization number that corresponds to the dismissed subject's treatment sequence. A complete description of the randomization procedure will be described in a Randomization Plan.

6.7 Blinding and Code Breaking Procedures

This is an observer-blind study. A sealed envelope containing the randomization scheme must be kept in a locked, secured location at the clinical site. The randomization code could not be broken except in emergency cases by the unblinded staff member at the clinical site (wherein the Investigator judges that the identification of the treatment is necessary for the management of the event, for appropriate treatment of the subject or if directed by the Agency). Individual envelopes containing the treatment received will also be provided. In case of emergency, only the subject's individual envelope will be opened which will preserve the blinding for other subjects. Any code break will be documented and reported to the Sponsor (refer to Section 3 for Sponsor Contact) in a timely manner. In the event of a non-emergency, the Investigator shall contact the Sponsor Contact (refer to Section 3) to discuss the rationale for unblinding and to determine if the blind should be broken for that subject or not.

This study is blinded through Day 201. Exceptionally, the randomization code will be broken after Day 21 of the study (last subject) for analyses purposes, only (with limited access). This will allow discussions about the clinical data and for public release of data, without having to wait after the 6-month follow-up period for study completion. It is anticipated approximately 5 people from Medicago staff members and few staff (clinic and CRO) will be allowed to see the randomization code. These staff members will not have any contact with the study subjects or to the subjects' data, and will not participate in the evaluation of any adverse reaction or reactogenicity evaluation of the subjects following immunization in order to avoid any bias. This unblinding procedure and targeted people will be documented. Also, the staff at Medicago responsible for doing the immunological analysis in the laboratory will remain blinded as well as the staff at the clinical site (except the staff involved in the preparation/administration of study vaccine and QA Auditor [Quality assurance] as well as QC reviewers [Quality control]).

6.8 Concomitant Therapy

Subjects should be instructed to notify the Investigator or designee about any new medications intakes or changes. Any new or changed medications reported by the subject post-vaccination and through the Day 21 visit, will be recorded. Since AEs may be secondary to new medications, Investigator or designee will explore the reasons for the new medications intakes and document these AEs, if any. Concomitant medications will include prescription and over-the-counter (OTC) drugs, vitamins, herbal products, and nutritional supplements (any product viewed by the subject as distinct from food). Investigators and other study staff are encouraged to capture the most detailed description of vitamins, herbals, and supplements possible; as otherwise, these products may be difficult to classify.

Concomitant medications must be reported in the CRF as indicated below:

- Within 30 days preceding vaccination or at any time from Day 0 through the final study contact (Day 201):
    - Any treatments and/or medications specifically contraindicated (e.g., any immunoglobulins or other blood products, or any immune modifying drugs).

- From Day 0 (day of any study vaccine dose) through Day 21 after vaccination:
  - All concomitant medications/vaccines.

    NOTE: Any changes in concomitant medication intakes (as total daily dose, route of administration, or end date information) provided after Day 21 will also be collected in MA by the subjects. Clinical site staff will record any changes in source data. For the medically-attended event only, the subject's eCRF will be updated with new information, accordingly.
- From Day 0 through the final study contact (Day 201):
  - Any concomitant medication(s) administered to treat a medically-attended AE (refer to Section 8.1.1 for definition).
  - Any concomitant medication(s) administered for the treatment of an SAE.
  - Any vaccine not foreseen in the study protocol.
  - Any investigational medication or vaccine.

Given that an important goal of this study is to evaluate the tolerability of the study vaccine, the use of prophylactic medications to prevent or pre-empt symptoms due to vaccination is specifically prohibited over the course of this study. A prophylactic medication is a medication administered in the absence of ANY symptom and in anticipation of a reaction to the vaccination (e.g., an anti-pyretic is considered to be prophylactic when it is given in the absence of fever and any other symptom, to prevent fever from occurring, vitamins used to boost immune system, etc.).

7. SPECIMENS AND CLINICAL SUPPLIES

7.1 Management of Samples

Blood samples for biochemistry, haematology, and serology analyses will be performed at screening; urinalysis and serum pregnancy testing will also be performed at screening. Blood samples for biochemistry and haematology analyses and urinalysis will be repeated at Day 3 and at Day 201. In case the screening procedures is performed more than 30 days prior to subject immunization, an extra blood sample will also be collected at Day 0 and will be analyzed only if deemed required according to Investigator's opinion. Serology samples for immunogenicity analysis will also be taken at Days 0, 21, ≥42 (when applicable), and 201. Refer to the Study Laboratory Manual for complete information on the handling and shipment of all laboratory samples.

7.1.1 SAMPLE COLLECTION

Blood samples will be collected at screening, Day 0 (when applicable), Day 3, and Day 201 for:

Biochemistry analyses as follows: haemoglucose (HbA1c), sodium, potassium, urea, creatinine, alkaline phosphatase, alaninetransferase (ALT), bilirubin, albumin, aspartatetransferase (AST), and gamma glutamyltransferase (GGT).

Haematology analyses as follows: haemoglobin, hematocrit, red blood cells (RBC), platelets, mean cell haemoglobin (MCH), mean cell concentration (MCHC), mean cell volume (MCV), white cell count (WBC total), neutrophils, lymphocytes, monocytes, eosinophils, and basophils.

Urine samples will be collected at screening, Day 3, and Day 201 for:

Urinalysis as follows: macroscopic examination (as color and aspect) and pH, specific gravity, glucose, protein, and blood.

Pregnancy testing will also be performed for all females. A serum pregnancy test will be performed during screening and a urine pregnancy test (dipstick) performed on Day 0 prior to randomization and Day 21 (refer to the Study Laboratory Manual for details on pregnancy test procedures).

The following serology tests will also be conducted at screening: screen for HIV p24 antigen/antibodies, antibody/antigen profile for Hepatitis B and Hepatitis C testing.

In addition, 4 blood samples (or 5 for subjects who will have received the licensed vaccine at the clinical site) for serological analysis (HI and MN titer measurements) will be collected from each study subject (approximately 10 mL each time) prior to administration of the study vaccine (Day 0), at Day 21, Day 42 (when applicable) and at Day 201. Should a subject be discontinued from the study, a sample will be drawn at the time of the final safety visit. Sera will be sent to Medicago for analysis (refer to Section 3 and to the Study Laboratory Manual for complete information about shipments).

Also, 3 blood samples for CMI assays will be collected (approximately 40 mL [4 tubes]) at each timepoint) on 15 subjects from each treatment group prior to the immunization (at Day 0), Day 21, and Day 201. The blood samples (collected only at the MUHC site), will be shipped and used to prepare PBMCs at ImmuneCarta (refer to Section 3) and will be cryopreserved as quickly as possible following collection (maximum 12 hours).

7.1.2 SAMPLE PREPARATION

Preparation of the laboratory samples for the collection and shipping of biochemistry, haematology and urinalysis will be described in the Study Laboratory Manual. Labeled samples will be ship to Safety Laboratory (refer to Section 3) on the day of collection (or later, if this not alter specimen).

Blood samples for serology (HI or MN tests) will be collected in one (1) 10 mL red top (plain/no additives) tube. The samples will be kept at room temperature for a minimum of 30 minutes and then centrifuged (speed 1200 g) for 10 minutes at ambient temperature. Serum will be divided into 7 aliquots into 7 separate polypropylene tubes as soon as possible: four of at least 0.5 mL (when possible) and the other three containing equal amounts of the remainder of the serum (when possible). In case the samples cannot be process shortly after the 30-minute period, the samples will be kept at room temperature for 30 minutes and then stored in the fridge until centrifugation (for a maximum of 24 hours).

Medicago will measure HI and MN titers in serum samples using validated methods. Serum samples from subjects who do not complete the study through Day 21, regardless of the reason, will be analyzed and reported, but the results will not be used for the study analysis.

Medicago will be responsible to send small quantities of serum to Laboratoires Bio-Medic (Refer to Section 3) who will measure the anti-glycan antibodies, using the ImmunoCAP technology.

Blood samples for preparation of PBMC for cell-based assays will be transferred at room temperature from the MUHC clinic to ImmuneCarta (refer to Section 3) for samples processing (there should be no more than 3 hours between samples collection and processing). There, peripheral blood mononuclear cells will be isolated according to ImmuneCarta procedures and cryopreserved into 2 to 4 aliquots (depending upon cell counts) at approximately 5-10 x 10^6 cells/mL. These cells will be kept under liquid nitrogen at ImmuneCarta Services facility for further analyses.

NOTE: Some variations in sample preparation may be used upon agreement by Sponsor but in any case, all changes will be documented in appropriate form (refer to Study Procedures Manual). Also, further specific information relating to sample preparation, storage, and shipment will be presented in the Study Laboratory Manual.

7.1.3 SAMPLE STORAGE AND SHIPMENT

Serology samples:

Serology serum samples should be stored at approximately -80ºC (-20ºC is acceptable at the clinical sites prior to shipment) until analysis. Sera must be shipped frozen on dry ice. The Day 0, Day 21, (Day 42, approximate) and Day 201 sera samples (first set of aliquots) and back-up sera samples (second set of aliquots), will be sent to Medicago (refer to Section 3 for address and contact) in at least 5 shipments, according to the following order:

1) Day 0 and Day 21 samples. This shipment could be divided in 2 batches;
2) Day 42 samples (from subjects who chose to receive the licensed vaccine). This shipment could be divided in 2 batches;
3) All Day 0, 21, and 42 (see above) back-up samples;
4) Day 201 samples;
5) Day 201 back-up samples.

Once Medicago confirms the reception of the first shipment (first set of aliquots), the back-up samples (second set of aliquots) will be sent. Samples could be sent earlier at Sponsor's request. No samples should be shipped on a Friday or the weekend. An alternate shipment schedule could be used, upon Medicago's approval. All shipments must be accompanied by the shipping manifest, an electronic temperature monitor device (such as TempTale®), and sufficient dry ice to maintain samples integrity (refer to the Study Laboratory Manual for details).

PBMC samples:

Samples prepared at ImmuneCarta will be stored in liquid nitrogen until use for analysis at ImmuneCarta.

7.2 Clinical Supplies

Clinical site will provide serology sera supplies (sampling tubes, aliquot tubes, aliquot labels, aliquot storage, shipping boxes, and accompanying manifests). Disposable syringes, needles, and tubes will be supplied by the clinical site as well. Complete study sample management procedures are described in the Study Laboratory Manual.

8. SERIOUS ADVERSE EVENTS (SAES) AND ADVERSE EVENTS (AES)

8.1 Reporting of Serious Adverse Events (SAEs)

A SAE (experience) or reaction is any untoward medical occurrence (whether considered to be related to study vaccine or not) that at any dose:

- Results in Death;
- Is life-threatening (at the time of the event);
- NOTE: The term "life-threatening in the definition of "serious" refers to an event in which the subject was at risk of death at the time of the event; it does not refer to an event which hypothetically might have caused death if it were more severe.
- Requires inpatient hospitalization (≥24 hours) or prolongation of existing hospitalization (elective hospitalizations/procedures for pre-existing conditions that have not worsened are excluded);
- Results in persistent or significant disability/incapacity;
- Is a congenital abnormality/birth defect.

Medical and scientific judgment should be exercised in deciding whether expedited reporting is appropriate in other situations, such as important medical events that may not be immediately life-threatening or result in death or hospitalization but may jeopardize the subject or may require intervention to prevent one of the other outcomes listed in the definition above. These should be considered serious. Examples of such events are intensive treatment in an emergency room for allergic bronchospasm, blood dyscrasias or convulsions that do not result in hospitalization, or development of drug dependency or drug abuse.

See Section 8.1.3 for Initial SAE Reporting by the Investigator and Sponsor representatives.

8.1.1 ADVERSE EVENTS

An AE or adverse experience is defined as any untoward medical occurrence in a subject or clinical investigation subject administered a pharmaceutical product and which does not necessarily have to have a causal relationship with the treatment. An AE can be any favorable and unintended sign (including an abnormal laboratory finding, for example), symptom, or disease temporally associated with the use of a medicinal product, whether or not considered to be a medicinal product.

The following information regarding each AE will be obtained: date and time of onset and resolution (duration), intensity (defined below), whether it was serious, any required treatment or action taken, outcome, relationship to the investigational vaccine, and whether the AE caused withdrawal from the study.

The intensity of all AEs (including lymphadenopathy [lymph node swelling of the neck and axilla]) will be graded as mild (1), moderate (2) severe (3), or potentially life-threatening (4), according to the FDA Guidance for industry[18]. Refer to Table 10 (Section 5.2.2.1) for grades definition. The Investigator (or delegated Sub-Investigator) will be instructed to closely monitor each subject who experiences an AE (whether

ascribed to the investigational product or not) until the outcome of the AE has been determined.

Any AE/SAEs, any signs, symptoms or diseases experienced by the subject following the signature of the ICF but before administration of the vaccine will be recorded in source data as pre-dose events (as a part of subjects' medical histories).

Unsolicited AEs and/or solicited local and systemic reactions of greater than Grade 2 (moderate), they will have to report it promptly (as soon as possible) to the clinical site staff (refer to MA for emergency contact). The Investigator should be informed within 48 hours after the knowledge by the clinical site and may request the subject come to the clinic for evaluation. The Investigator will document review of these events on the source data.

Any clinical laboratory test result that meets the criteria for an AE in the absence of appropriate and/or adequate clinical diagnosis should be reported as an AE, unless the result is considered normal for the current trial subject population, as determined by the Investigator.

If any of the solicited local or systemic reactions persist beyond Day 7, these will also be recorded as an AE. In this case, the AE start will be set as 168 hours (7 days) post-vaccination. The subject will be requested to note when the AEs resolve and report this information to the Investigator or clinic staff at the next visit at the clinical site or contact.

In the event of a clinically important AE, a suitable sample could be collected for drug assay or for relevant additional laboratory tests.

The clinical importance will be determined according to the Investigator's judgment. The Investigator must ensure that the sample is properly labeled and stored. The Investigator and others responsible for care of the subjects should institute any supplementary investigations of significant AEs based on the clinical judgment of the likely causative factor. This may include seeking a further opinion from a specialist in the field of the AE.

All AEs occurring within 21 days after vaccination must be recorded in the source data and reported in the "Adverse Event" screen in the subject's CRF, irrespective of intensity or whether or not they are considered vaccination-related. Thereafter, through completion of the Day 201 visit, new AEs will be recorded in the source data but will be reported in the CRF only if they meet the definition for an SAE or for a medically attended AE (defined as any event for which the subject received medical attention such as hospitalization, an emergency room visit or a visit to or from medical specialist [i.e., (but not limited to) physician, dentist, physiotherapist, hospital personnel, pharmacist, etc.] regardless reason). In any case, all AEs have to be reviewed by the Investigator for their relationship to the vaccine.

Medically relevant AEs will be followed up until complete resolution or based on Investigator's decision. Follow-up of unresolved AEs after Day 201 will continue under the discretion of the Investigator.

8.1.2 EXPECTEDNESS OF AN ADVERSE DRUG REACTION

An "unexpected" adverse reaction is one, in which the nature or severity of the event is not consistent with information in the current Investigator's Brochure. Expedited reporting to regulatory authority is required if an AE is an SAE that is both unexpected and considered possibly related to the study drug.

8.1.3 INITIAL SAE REPORTING BY THE INVESTIGATOR

SAEs related to pre-treatment study procedures will be reported from the time the ICF is signed. All post-vaccination SAEs (treatment-emergent SAEs) will be reported from the time of receiving the study vaccine on Day 0 through to the Day 201 (Final Safety Visit). The Investigator must report via phone, all SAEs, whether considered related to the study vaccine or not, and whether considered unexpected or expected (as defined in Section 8.1.2), to the Sponsor, according to appropriate authority requirements (within 24 hours [one business day] of the Investigator's learning of the event). The Investigator must also complete, sign, and date the SAE report form, and send via email a copy to the Safety Contact (below):

Dr. Brian J. Ward, M.D., Medical Officer
brian.ward@mcgill.ca
Tel.: 1.514.934.1934 ext. 42810
After hours:
Mobile: 1.514.952.6583
Pager: 1.514.921.6953

A copy must also be emailed to:

Sonia Trepanier, Clinical Study Director
trepaniers@medicago.com
Tel.: 1.418.658.9393, ext. 137
Fax: 1.418.658.658.6699
Mobile: 1.418.655.7158

Sebastien Soucy, Clinical Study Manager
soucys@medicago.com
Tel.: 1.418.658.9393, ext. 122
Fax: 1.418.658.6699
Mobile: 1.418.446.2032

Sponsor Contact (refer to Section 3) will be responsible to promptly (on the same day as received or at the latest the next business day) transfer the information to the Regulatory Affairs department at Medicago.

8.1.4 FOLLOW-UP REPORTING BY THE PI

All SAEs, regardless of causality, will be followed to resolution until the event returns to the baseline condition, the event stabilizes, or if ongoing at Day 21, the SAE will be followed until resolution, regardless of conclusion of the study. Where appropriate, documentation of any medical tests or examinations performed will be provided to document resolution of the event.

8.1.5 REPORTING OF SAEs OCCURRING AFTER SUBJECT TRIAL TERMINATION

All SAEs occurring during the study period will be followed until resolution or for a period of 30 days from the final study visit at Day 201, regardless of conclusion of the study.

A post-study AE/SAE is defined as any event that occurs outside of the AE/SAE detection period. Investigators are not obligated to actively seek AEs or SAEs in former study subjects. However, if the Investigator learns of any SAE, including a death, at any time after a subject has been discharged from the study, and he/she considers the event reasonably related to the investigational product, the Investigator will promptly notify the Study Contact for Reporting SAEs (refer to Section 8.1.3).

8.1.6 CAUSAL RELATIONSHIP

The Investigator must make the determination of relationship to the study vaccine for each AE.

The Investigator should decide whether, in his/her medical judgement, there is a reasonable possibility that the event may have been caused by the investigational vaccine. If there is any valid reason, even if undetermined or untested, for suspecting a possible cause-and-effect relationship between the investigational vaccine and the occurrence of the AE, then the AE should be considered "definitely related", "probably related", or "possibly related". Otherwise, if no valid reason exists for suggesting a possible relationship, then the AE should be classified as "probably not related" or "definitely not related". The following guidance may be helpful:

Definitely Not Related: The AE is clearly not related to the administration of the study vaccine. Another cause of the event is most plausible, and/or a clinically plausible temporal sequence is inconsistent with the onset of the event and the study vaccine administration; and/or a causal relationship is considered biologically implausible.

Probably Not Related: There is no medical evidence to suggest that the AE is related to the study vaccine. The event can be readily explained by the subject's underlying medical condition or concomitant therapy or lacks a plausible temporal relationship to the study vaccine.

Possibly Related: A direct cause and effect relationship between the study vaccine and the AE has not been demonstrated but there is a reasonable possibility that the event was caused by the study vaccine.

Probably Related: There probably is a direct cause and effect relationship between the AE and the study vaccine. A plausible biologic mechanism and temporal relationship exist and there is no, more likely explanation.

Definitely Related: There is a direct cause and effect relationship between the AE and the study vaccine. Reactions at the injection site (redness, swelling, and pain) will automatically be entered as definitely related to the study vaccine.

The medical assessment of intensity will be determined by using the same definitions as for AEs (refer to Section 8.1.1).

The outcome may be classified as resolved, ongoing, resolved with sequelae, or death.

8.1.7 REPORTING OF SAEs TO HEALTH AUTHORITIES AND IRB/REB

Medicago will be responsible for reporting SAEs that are deemed possibly related to study vaccine and unexpected (Unexpected refers to not appearing in the package labeling or in the study vaccine IB) to regulatory authorities in an expedited manner.

The Investigator will be responsible for reporting all SAEs directly to their relevant ethical review body (IRB or REB) as soon as possible, and will also provide the ethical review body with any safety reports prepared by the Sponsor.

8.1.8 SEVEN (7)-DAY SAFETY MONITORING

A review of 7-day safety data will be carried out to ensure ongoing safety of study subjects as well as maintaining study scientific integrity. This review will detect any early negative trends in the safety data and may necessitate a decision to not administer a second immunization to study subjects (who choose to receive the licensed vaccine at the clinical site). A phone call reminding study subjects to complete the Memory Aid (e.g. diary card) will be performed at Day 1 post-vaccination. Eight (8) days following the immunization, subjects will be contacted by clinic staff to review and record 7-day safety data. Early accumulated safety outcome data which will include all self-reported solicited local and systemic reactions, any AE/SAEs occurring following administration of each injection will be collected and tabulated. For subjects immunized with the licensed vaccine at the clinical site, a review of safety data will also be performed (via phone contact) 7 days after the second immunization.

The 7-day safety data of the first cohort will be reviewed by DSMB before allowing the vaccination of the cohort 2. A review of the 7-day safety data of the second cohort will also be performed by the DSMB before allowing vaccination of cohort 3. During the slow enrolment, the DSMB safety review would be performed on all safety data available in order to confirm whether the clinic site can go ahead with the immunization of the next cohort or not.

Once Day 21 period of the study is completed, statistical analysis of safety data will be performed by comparing the placebo group to each dose level group and the dose levels will be compared to each other. However, due to the timeframe of the analysis involved, the 7- Day safety data may not be monitored for the purposes of the 7-day safety review, but these data will be monitored in advance of the clinical study report.

The safety experts will be provided the following blinded 7-day solicited local and systemic reactions:

- Occurrences of erythema, swelling, and pain at the injection site;
- Occurrences of fever, headache, muscle aches, joint aches, fatigue, chills, malaise, and swelling in the axilla or neck;
- Occurrence of any AE/SAEs;
- OT results from the first 7-day post-vaccination will also be provided;
- Concomitant medications, doctor or emergency room or hospital visits associated with an AE/SAEs.

The occurrence of one or more SAEs considered to be related (i.e., possible, probably or definitely related) will result in a halt to the study at any time during the duration of the study, for further review and assessment of the event(s). The halting rules for this study also include:

- Any death occurring during the study
- The study be halted for further review and assessment across all sites if 20% of the subjects (in total, regardless of treatment group) experience during the 7-day safety period after vaccination:
  - a severe (Grade 3 or higher) vaccine-related local reaction,
  - a severe (Grade 3 or higher) vaccine-related quantitative systemic
  - reaction,
  - a severe (Grade 3 or higher) vaccine-related subjective systemic reaction of that the severity (grade) is corroborated by study personnel.
- The study will be halted for further review and assessment across all sites if 20% of subjects (in total, regardless of treatment group) experience, during the 21-day evaluation, a severe (Grade 3 or higher) vaccine-related clinical laboratory AE.

All SAEs should be reported to Health Canada by phone or by facsimile transmission of any unexpected fatal or life-threatening experience associated with the use of the drug as soon as possible but in no event later than 7 calendar days after the Sponsor's initial receipt of the information; and should be followed to resolution, stabilization, or return to baseline regardless of conclusion of the study.

The previous stopping rules will also apply for the duration of the study. If a study subject has a SAE at any time during the study that the investigator determines may be related to the study vaccine (i.e., possibly, probably, or definitely related) then the study must stop until a full investigation of the event is conducted. At any time during the study, a SAE that was thought to be unrelated to the study vaccine would not warrant stopping the trial.

9. DATA COLLECTION AND MANAGEMENT

9.1 Data Collection, CRF Completion

The clinical sites will perform data collection, transfer tasks, and monitoring (if applicable). A Clinical Monitor designated by the CRO (and approved by the Sponsor) or the Sponsor will be responsible (or partly responsible) for monitoring the clinical study, in accordance with the current appropriate regulations.

The Investigator will maintain the individual subjects' files. The files will include visit dates of the subject, records of vital signs, medical history or examinations, concomitant medications, any AE's encountered, and any other notes, as appropriate. These documents constitute source data.

The study data will be entered into a 21 CFR Part 11 compliant CRF using electronic data capture (EDC). The data system will include password protection and internal quality checks such as automatic range checks to identify data that appears inconsistent, incomplete, or inaccurate. All entries on the CRF must be backed up by source data.

Clinical laboratory safety analyses will be performed by a safety laboratory (refer to Section 3 for details on different laboratory contacts) and then provided to site personnel for subjects' eligibility. There will be no direct entry into the CRF by the clinical site; the clinical laboratory data will be transferred from the laboratory to the personnel in charge of data management (refer to Section 3).

Queries will be generated for spurious data and clarification sought from the responsible Investigator or delegate at the clinical site. These data queries must be resolved in a timely manner by the clinical site personnel.

The study may be subject to an audit by an authorized representative(s) of Medicago and/or inspections by an authorized Regulatory Authority (e.g., Health Canada). Regulatory authorities may request access to all study documentation, including source data for inspection and copying, in keeping with local regulations. The Sponsor will immediately notify the Investigator of an upcoming audit/inspection.

In the event of an audit, all pertinent study-related documentation must be made available to the auditor(s). If an audit or inspection occurs, the Investigator will permit the auditor/inspector direct access to all relevant documents and allocate their time as well as the time of relevant staff to discuss the findings and any relevant recommendations.

Immunogenicity data obtained at Medicago will be transferred to the CRO who will take care of data entry in the SAS® database for statistical analysis.

None of the data to be collected during this trial will be estimated, if missing. Once the final study subject has completed the study evaluations and the CRF have been entered and queries resolved, the database can be locked following appropriate QC/QA procedures.

The Investigator must submit a completed CRF for each subject who receives the study vaccine. CRFs should be signed and dated by the Investigator to document his review of the data and acknowledgement that the data are accurate.

The most recent version of the MedDRA dictionary will be used for coding AEs. The most recent version of the WHO-Drug dictionary will be used for coding concomitant medications.

CRF completion guidelines will be issued separately.

9.2 Data Management

Data management will be performed by inVentiv Health.

10. STATISTICAL METHODS AND DETERMINATION OF SAMPLE SIZE

10.1 General Considerations

Data analysis will be performed by inVentiv Health. A complete description of the statistical analyses and methods will be reported in a Statistical Analysis Plan (SAP) to be finalized before database lock.

A clinical study report will be provided after Day 21. An addendum report containing all results analysis, including data from after Day 21 to Day 201, will be completed.

All descriptive and inferential statistical analyses will be performed using SAS® software (version 9.2 or higher). In general, continuous data will be summarized using descriptive statistics (i.e., mean or geometric mean, median, standard deviation, minimum, and maximum). Categorical data will be summarized using the number and percent of subjects in each category.

Descriptive statistics will be provided for the safety data. All inferential analyses of the safety data and immunogenicity endpoints will use a two-sided alpha level of 5%. No attempt will be made to control type I error rate inflation attributable to multiple comparisons unless otherwise stated.

Demographic and baseline characteristics will be summarized descriptively to assess the comparability between each vaccine group.

10.1.1 ANALYSIS OF PRIMARY SAFETY ENDPOINTS

All safety endpoints will be summarized by treatment group using descriptive statistics and appropriate statistical test when applicable.

The following safety endpoints will be assessed:

- Percentage, intensity, and relationship of immediate complaints, 30 minutes post-vaccination;
- Percentage, intensity, and relationship to vaccination of solicited local and systemic signs and symptoms 7 days following a single dose of study vaccine;
- Percentage, intensity, and relationship of unsolicited local and systemic signs and symptoms 21 days following a single dose of study vaccine;
- Occurrences of all AEs/SAEs;
- Occurrences of new onset of a chronic disease (NOCD);
- Number and percentage of subjects with normal and abnormal urine, haematological, and biochemical values at Screening, Days 3 and 201.

The counts and proportions of subjects in each vaccine group with immediate complaints and solicited symptoms will be tabulated by severity and also by relationship; local and systemic signs and symptoms will be tabulated separately and combined. The proportion of subjects with a non-zero grade, grade >2, grade >3 will be included. The number of subjects experiencing each solicited event and also experiencing immediate complaints will be compared between each vaccine dose level and also between vaccine dose levels and placebo group using Fisher's Exact or chi-squared tests.

The occurrence of unsolicited (coded) AEs will be tabulated and compared for the time period of 21 days after the vaccination. The occurrence of unsolicited (coded) AEs will also be tabulated by severity and relationship. Occurrence of unsolicited (coded) AEs will be compared (each vaccine dose level versus placebo) using Fisher's exact tests or Chi-Square test when appropriate, and also between vaccine dose groups.

Safety results will also be presented by age strata (50-64 and 65 and older, in a ratio of 1:1 (37 and 38 subjects in each treatment group respectively).

AE incidence rates will be compared between pairs of vaccine groups using Fisher's exact tests; statistical comparisons will only be made for all vaccine-emergent AEs. Given the multiplicity of comparisons contemplated, the safety analyses for both solicited reactions and

spontaneous AEs will feature markedly elevated type I error; however, no multiple comparisons corrections will be applied. Because of the increased probability of observing significant differences with multiple comparisons, patterns of AE rates and p-values will be examined rather than focusing on individual p-values. Additionally, since the study was not powered to detect differences in AE rates between treatment groups, it is understood that if significant differences are not apparent that this does not mean that they are non-existent.

Laboratory data and vital signs at each study timepoint will be summarized using descriptive statistics.

10.1.2 ANALYSIS OF PRIMARY IMMUNOGENICITY ENDPOINTS

Refer to Section 2.1.1 for a description of the primary immunogenicity endpoints.

The FDA's criteria for the population of ≥65 years of age will be used to analyze the primary immunogenicity endpoints achieved within each treatment groups of 75 subjects. Refer to Section 10.3 for determination of sample size.

Considering each age strata separately, the immunogenicity endpoints calculation (seroconversion rate, seroprotection rate and GMFR) will be performed for the H1N1, H3N2, and the B/Brisbane and B/Massachusetts virus strains for each treatment group.

For seroprotection and seroconversion rates on Day 21, the 95% confidence intervals will also be presented for each virus strains and treatment group per age strata (50-64 and 65 and older, in a ratio of 1:1 (37 and 38 subjects in each treatment group respectively).

The results will be compared to the EMEA (CPMP) immunogenicity criteria described in section 2.1.1. Moreover, the FDA (CBER) immunogenicity criteria described in section 2.1.1 will also be considered.

Considering the group size of each age strata per treatment group is too small to use the FDA criteria to analyze the immunogenicity endpoints, EMEA criteria for respective age strata will be used to analyze the seroconversion factor (or GMFR), seroconversion rate and seroprotection rate obtained in each age strata, for each treatment group.

10.1.3 ANALYSIS OF SECONDARY IMMUNOGENICITY ENDPOINTS

Refer to Section 2.2.1 for a description of the secondary immunogenicity endpoints.

For Day 21 and Day 201 separately, GMFR will be compared between active dose groups and also between each active dose group to placebo using analysis of variance (ANOVA) methods. A multiple comparison test (i.e., Tukey's range test) will be used to evaluate differences among the treatment groups.

For Day 21 and Day 201 separately, seroconversion rates and seroprotection rates (for HI only) will be compared (i.e., each vaccine dose level versus placebo and also between active dose groups) using Fisher's exact tests or Chi-Square tests. The difference and the 95% CI on the difference between the proportions of seroconverted or seroprotected subjects in each dose group compared to placebo will be calculated. Details for calculation will be provided in the SAP.

Number of subjects and proportion of subjects with a detectable HI antibody titer (≥LOQ) at Day 0, Day 21, and Day 201 will be tabulated. The number of subjects with a test result at each timepoint will be used as the denominator.

Results will be presented by age strata (50-64 and 65 and older, in a ratio of 1:1 (37 and 38 subjects in each treatment group respectively).

10.1.4 EXPLORATORY IMMUNOGENICITY ENDPOINTS

Refer to section 2.3 for the description of the exploratory endpoints.

Data from exploratory endpoints will not be included in the clinical study report. If required, ANOVA or Fisher's exact tests or Chi-square test when appropriate, will be performed on continuous or categorical endpoints respectively. Since the number of samples collected for exploratory endpoints may be small, nonparametric methods will be used in the event that the distribution of data does not meet the normality assumption. Results will also be presented by age strata (50-64 and 65 and older, in a ratio of 1:1 (37 and 38 subjects in each treatment group respectively). Note that analysis of these sera may be stratified by the interval between the 2 injections.

10.2 Analysis Populations

10.2.1 DEFINITION OF POPULATION

10.2.1.1 SAFETY ANALYSIS SET

The Safety Analysis Set is defined as all subjects who received any study treatment. All safety analyses will be performed using the Safety Analysis set.

10.2.1.2 FULL ANALYSIS SET

The Full Analysis Set (FAS) will consist of the subset of the Safety Set with a Day 0 and any post-vaccine immunogenicity assessment. Subjects who received an incorrect treatment will be analyzed as randomized. All primary and secondary immunogenicity endpoints will be analyzed using the FAS.

10.2.1.3 PER-PROTOCOL IMMUNOGENICITY POPULATION

The Per Protocol Set (PP) will consist of the subset of the FAS who completed the study without a major protocol violation.

For the Day 21 analysis, this should include the subjects who received the vaccine dose and had Day 0 and Day 21 immunogenicity measurements. Subjects who had blood samples for immunogenicity taken outside of the time window are to be excluded from the PP set for this specific visit. Subjects who received the wrong vaccine, but for whom the treatment received can be unequivocally confirmed, will be analyzed in the PP set as treated, provided they have no other violations that compromise their data.

For the Day 201 analysis, this should include the subjects who received the vaccine dose and had Day 0 and Day 201 immunogenicity measurements. The Day 201 PP Set is defined for data summaries of data collected at the Day 201 visit. Inclusion in this population has no impact on data summaries at other visit.

The immunogenicity endpoints of seroconversion factor, seroconversion rate, and seropositive rate will be performed using the PP set as sensitivity analyses.

10.3 Determination of Sample Size

The sample size needed to meet FDA's criteria for seroprotection in adults or in elderly: the lower bound of the 95% CI for the percentage of subjects achieving an HI reciprocal ≥40 should meet or exceed 70% or 60%, respectively.

Based on the HI titers results obtained for the quadrivalent vaccine tested in the previous clinical study in adults (CP-Q12VLP-004) and presented in Introduction, statistical analysis were performed to recommend a proper sample size. Statistical analysis was performed on the seroprotection criteria as this criterion is usually more difficult to achieve. Based on this analysis, in a treatment group of 75 subjects, seroprotection rates of at least 81% should have a lower bounded 95 % exact confidence limit that is above the desired percentage of 70% for the 4 vaccine strains. This study has not been powered to show differences between treatment groups, but it may be possible to show substantial differences between active treatments and placebo.

A study of this size is not powered to detect rare AEs; however, it will be possible to show substantial differences in rates of AEs. For AEs with an incidence of 4.5%, the probability of observing at least 1 event in a sample size of 75 is 97%.

10.4 Handling of Missing Data

Refer to the SAP for details regarding missing immunogenicity data.

11. ETHICAL AND LEGAL ISSUES AND PI/SPONSOR RESPONSIBILITIES

11.1 Ethical Conduct of the Trial / Good Clinical Practice (GCP)

The study will be conducted in accordance with the ethical principles that have their origins in the Declaration of Helsinki, International Conference on Harmonization (ICH) Guideline E6, GCP rules, and other applicable regulatory requirements.

The Investigator is responsible for obtaining written approval from the IRB/REB for the clinical study protocol (including all protocol amendments), the written ICF, informed consent updates, subject recruitment procedures (e.g., advertisements), and any other written information to be provided to the subject, which complies with local regulatory requirements.

The Investigator is responsible for maintaining a copy of the approval document in the study documentation files and for providing a copy to Medicago.

The Investigator should submit written reports of the clinical study status to the IRB/REB annually or more frequently, if requested by the IRB/REB. A final study notification should be forwarded to the IRB/REB after the study has been completed or in the event of premature termination of the study. A copy of all clinical study status reports (including termination) should

be provided to Medicago. The end of the trial is defined as the date of the last contact with any subject in the trial. The end of the trial must be notified to the IRB/REB within 90 days or within 15 days if the trial is halted or terminated early.

Following the reporting of serious adverse reactions and events to Medicago (Section 8.1.1), the Investigator must also promptly inform the IRB/REB of these; or other safety related information reported from Medicago.

11.2 Source Documents and Source Data

Source documents are original documents, data, and records of clinical findings, observations and other activities in a clinical study necessary for the reconstruction and evaluation of the study. Source documents will include: all logs and notes from the clinical site, laboratory records specific to this study, and relevant hospital records, clinical and office charts, laboratory notes, memoranda, evaluation checklists, pharmacy (or clinical site) dispensing records, recorded data from automated instruments, copies or transcriptions certified after verification as being accurate copies, microfiches, photographic negatives, microfilm or magnetic media, x-rays, subject files and records kept at the pharmacy (clinical site), at the laboratories and at medical/technical departments involved in the clinical study.

11.3 Confidentiality of Data and Access to Subject Records

Data collected during this study will be used to support the development, registration, and future marketing of VLP seasonal influenza vaccine. All data collected during the study will be controlled by the Sponsor or designee and the Sponsor will abide by all relevant data protection laws. After subjects have consented to take part in the study, their medical records and data collected during the study will be reviewed by representatives of the Sponsor and/or the company organizing the research on the Sponsor's behalf to confirm that the data collected is accurate and for the purpose of analyzing the results. These records and data may additionally be reviewed by auditors, interested commercial parties or by regulatory authorities. Outside of the clinical site, subjects will be referred to by a unique subject number rather than by name. The results of this study may be used in submissions to other countries throughout the world, which have ensured an adequate level of protection for personal data.

To ensure compliance with relevant regulations, data generated by this study must be available for inspection upon request by the Sponsor or an authorized representative of Medicago, regulatory authorities, and the IRB/REB.

11.4 Monitoring, Auditing and Archiving

11.4.1 MONITORING

The study will be monitored by representatives of Medicago throughout its duration. Moreover, representatives of the Sponsor will participate to the Site Initiation and Close-out visits as well as to one dosing day, at the Investigator's facilities. Other visits by Sponsor to the clinical site(s) are not excluded. Visits by Sponsor staff should be scheduled at mutually agreeable times periodically throughout the study. These visits will be conducted to evaluate the progress of the study, verify the rights and well-being of the subjects are protected, verify the reported clinical study data are accurate, complete and verifiable from source documents, and the conduct of the study is in compliance with the

approved protocol and amendments, GCP and applicable regulatory requirements. A monitoring visit should include a review of the essential clinical study documents (regulatory documents, CRFs, source documents, drug dispensing records, subject ICFs, etc.) as well as discussion on the conduct of the study with the Investigator and staff. Blinded and unblinded monitors will be assigned depending on the record to be reviewed.

The Study Monitor should conduct these visits as frequently as appropriate for the clinical study. The Investigator and staff should be available during these visits to facilitate the review of the clinical study records and resolve/document any discrepancies found during the visit.

11.4.2 AUDITS AND INSPECTIONS

In accordance with the principles of GCP, the study may be inspected by regulatory authorities, the Sponsor or assigned designee. The Sponsor is entitled to access information about the status of the study and to review the original study documents or site documents supporting the study.

During the course of the study, Quality Assurance (QA) auditors may audit the investigational site. As Medicago representatives, they are entitled to access all subject and study information, excluding the randomized treatment assignments.

11.4.3 ARCHIVING

The Investigator will retain the source documentation including: the original ICFs, study documentation (e.g. IRB/REB approval letters, etc.) and a copy of the completed CRFs for 25 years as per Canadian Food and Drug Regulations; Part C, Division 5, Section C.05.012 (3) (h). After 25 years, the Investigator must contact Medicago prior to the disposal of any study documents (Adopted from ICH guidelines, GCP, Section 4.9.5). If before the 25 year retention period is completed, the Investigator or institution can no longer maintain the study documentation, Medicago should be contacted to make other arrangements for storage.

11.5 Financial Contract and Insurance Coverage

Medicago has suitable insurance to cover clinical trials and product liability. The clinical sites must have suitable insurance to cover negligence and default liability.

11.6 Stipends for Participation

Subject stipends for clinical study participation must be clearly outlined in the ICF and approved by the IRB/REB.

12. REFERENCES

[1] Osterholm MT, Kelley NS, Sommer A, Belongia EA. Efficacy and effectiveness of influenza vaccines: a systematic review and meta-analysis. Lancet Infect Dis 2012;12(1):36-44.

[2] Palache A. Seasonal influenza vaccine provision in 157 countries (2004-2009) and the potential influence of national public health policies. Vaccine 2011;29:9459-9466.

[3] Molinari NA, Ortega-Sanchez IR, Messonnier ML, Thompson WW, Wortley PM, Weintraub E, Bridges CB. The annual impact of seasonal influenza in the US: measuring disease burden and costs. Vaccine

2007;25:5086-5096.

[4] DiazGranados CA, Denis M, Plotkin S. Seasonal influenza vaccine efficacy and its determinants in children and non-elderly adults: a systematic review with meta-analyses of controlled trials. Vaccine 2012;31:49-57.

[5] Chiu C, Wrammert J, Li GM, McCausland M, Wilson PC, Ahmed R. Cross-reactive humoral responses to influenza and their implications for a universal vaccine. Ann N Y Acad Sci 2013;1283:13-21.

[6] Gatherer D. Passage in egg culture is a major cause of apparent positive selection in influenza B hemagglutinin. J Med Virol 2010;82:123-127.

[7] Kim TS, Sun J, Braciale TJ. T cell responses during influenza infection: getting and keeping control. Trends Immunol 2011;32:225-231.

[8] Kreijtz JH, Fouchier RA, Rimmelzwaan GF. Immune responses to influenza virus infection. Virus Res 2011;162:19-30.

[9] McElhaney JE. Influenza vaccine responses in older adults. Ageing Res Rev 2011;10:379-388.

[10] Galli G, Medini D, Borgogni E, Zedda L, Bardelli M, Malzone C, Nuti S, Tavarini S, Sammicheli C, Hilbert AK, Brauer V, Banzhoff A, Rappuoli R, Del Giudice G, Castellino F. Adjuvanted H5N1 vaccine induces early CD4+ T cell response that predicts long-term persistence of protective antibody levels. Proc Natl Acad Sci U S A 2009;106:3877-3882.

[11] Appay V, van Lier RA, Sallusto F, Roederer M. Phenotype and function of human T lymphocyte subsets: consensus and issues. Cytometry A 2008;73:975-983.

[12] Farber DL, Yudanin NA, Restifo NP. Human memory T cells: generation, compartmentalization and homeostasis. Nat Rev Immunol 2014;14:24-35.

[13] Seder RA, Darrah PA, Roederer M. T-cell quality in memory and protection: implications for vaccine design. Nat Rev Immunol 2008;8:247-258.

[14] FDA Guidance for industry, Clinical data needed to support the licensure of seasonal inactivated influenza vaccines, May 2007. http://www.fda.gov/biologicsbloodvaccines/guidancecomplianceregulatoryinformation/guidances/vaccines/ucm074794.htm

[15] Foetisch K, Westphal S, Lauer I, Retzek M, Altmann F, Kolarich D, Scheurer S, Vieths S. Biological activity of IgE specific for cross-reactive carbohydrate determinants. J Allergy Clin Immunol 2003;111:889-896.

[16] Fotisch K, Altmann F, Haustein D, Vieths S. Involvement of carbohydrate epitopes in the IgE response of celery-allergic patients. Int Arch Allergy Immunol 1999;120:30-42.

[17] Westphal S, Kolarich D, Foetisch K, Lauer I, Altmann F, Conti A, Crespo JF, Rodriguez J, Enrique E, Vieths S, Scheurer S. Molecular characterization and allergenic activity of Lyc e 2 (beta-fructofuranosidase), a glycosylated allergen of tomato. Eur J Biochem 2003;270:1327-1337.

[18] FDA Guidance for industry, Toxicity grading scale for healthy adult and adolescent volunteers enrolled in preventive vaccine clinical trials, September 2007. http://www.fda.gov/biologicsbloodvaccines/guidancecomplianceregulatoryinformation/guidances/vaccines/ucm074775.htm

13. APPENDICES

SAMPLE-MEMORY AID DIARY (DAY 0 to DAY 21)	
**Day 0 to Day 21**	
**Study Name**	**Immunogenicity, Safety, and Tolerability of a Plant-Derived Seasonal VLP Quadrivalent Influenza Vaccine in Elderly**
**Protocol Number**	CP-Q13VLP-008
**Sponsor**	Medicago R&D Inc.<br>1020, route de l'Église, bureau 600<br>Sainte-Foy, Québec, Canada G1V 3V9
**Clinical site Address**	
**Principal Investigator Name**	
**Clinical Research Coordinator**	
**24-hour Emergency Number**	

SAMPLE MEMORY AID DIARY (DAY 0 to DAY 21)

Day 0 to Day 7

Instructions:

You should fill in your Memory Aid Diary at approximately the same time each evening.

You will need to refer to this Memory Aid Diary during the Telephone Calls.

Fill in the Day 0 to Day 7 tables EVERY DAY for the first 7 days after you receive your vaccine dose

You received the study vaccine on ________________ at ________________
yyyy-mm-dd hh:mm (24 hour format)

Oral Temperature:

Oral Temperature										
	**Scheduled date (yyyy-mm-dd)**									
	Timepoint since immunization	30 min, Post-dose	Evening, day of dose	1 Day after	2 Days after	3 Days after	4 Days after	5 Days after	6 Days after	7 Days after
	**DAILY TEMPERATURE (°C)**<br>Use the thermometer you were given at the clinic to measure the highest temperature obtained for each day.<br>Do not eat or drink food prior to take your temperature.									
	Comments:									

SAMPLE MEMORY AID DIARY (DAY 0 to DAY 21)

Day 0 to Day 7

Local Symptoms:

Fill in today's column by entering the WORST grade for each symptom that you have had in the last day (24 hours).

If you did not have the symptoms, enter "0"

**Symptoms**		**Grade** (see below)								
**Local**	Timepoint since immunization	30 min, Post-dose	Evening, day of dose	1 Day after	2 Days after	3 Days after	4 Days after	5 Days after	6 Days after	7 Days after
	**Redness where the injection was given** Use the ruler (given to you at the clinic) to estimate the size of the red area around the injection site.									
	**Swelling where the injection was given** Use the ruler (given to you at the clinic) to estimate the size of the swollen area around the injection site.									
	Grade:<br>0= Less than 25 mm<br>1= 25 mm or more but NOT more than 50 mm<br>(for swelling only: or does not interfere with activity)<br>2= More than 50 mm but NOT more than 100 mm<br>(for swelling only: or interfere with activity)<br>3= More than 100 mm<br>(for swelling only: or prevents daily activity)<br>4= Necrosis (death of tissue)<br>(for redness only: or exfoliative dermatitis)			Comments:						

Please list any other problems (not listed above) on page 5 and report them during your next visit at the clinic or contact.
Symptoms listed above which persist longer than 7 days after study vaccine administration should listed below and followed up until resolution.

medicaGO	**STUDY CP-Q13VLP-008**	Subject No.:
		Subject Initials:

SAMPLE MEMORY AID DIARY (DAY 0 to DAY 21)

Day 0 to Day 7

Systemic Symptoms:
Fill in today's column by entering the WORST grade for each symptom that you have had in the last day (24 hours).
If you did not have the symptoms, enter "0"

**Symptoms**		**Grade** (see below)								
**Systemic**	Timepoint since immunization	30 min, Post-dose	Evening, day of dose	1 Day after	2 Days after	3 Days after	4 Days after	5 Days after	6 Days after	7 Days after
	Pain at vaccine injection site									
	Headache									
	Muscle aches									
	Joint aches									
	Fatigue									
	Chills									
	Feelings of general discomfort or uneasiness									
	Feeling of swelling in the axilla									
	Feeling of swelling in the neck									
	Grade:<br>0= No symptom<br>1= Mild: Noticeable, but doesn't interfere with my work/normal activities.<br>2= Moderate: Bad enough to limit my work/normal activities.<br>3= Severe: Bad enough to prevent my work/normal activities<br>4= Results to a visit to an emergency room or hospitalization				Comments:					

Please list any other problems (not listed above) on page 5 and report them during your next visit at the clinic or contact.
Symptoms listed above which persist longer than 7 days after study vaccine administration should listed below and followed up until resolution.

SAMPLE MEMORY AID DIARY (DAY 0 to DAY 21)

Day 0 to Day 7

Other Symptoms or Side Effects:

Use the space below to record important information that you want to tell us about changes in your health. Feel free to make a note of any health problems you experience, worsening of old problems, or new medicines you take.

- It is especially important that you make a note of problems that require a visit to your doctor, a hospital stay, or a visit to emergency room.
- It is also important that you make a note of changes in your medications. Each medication must have a problem associated with it but each problem does not require a medication associated with it.

Other Symptoms or Side Effects:

Symptom/Side effect	Grade<br>See below	Date it started<br>(yyyy-mm-dd)	Do you still have it?<br>(yes/no)<br>If it is over, when did it end?<br>(yyyy-mm-dd)	Did you make a visit or call to a doctor about it?<br>(yes/no)	Did you go to an emergency room or hospital? Did you stay in hospital?<br>(yes/no, specify)	Put an "X" in this box once you have told us about this problem

Grade:
0= No symptom
1= Mild: Noticeable, but doesn't interfere with my work/normal activities.
2= Moderate: Bad enough to limit my work/normal activities.
3= Severe: Bad enough to prevent my work/normal activities
4= Results to a visit to an emergency room or hospitalization

Comments:

SAMPLE MEMORY AID DIARY (DAY 0 to DAY 21)

Day 0 to Day 7

Changes in your Medication:

Record any new medications you have taken since your last visit. Examples may include prescription drugs, over-the-counter drugs, herbal product and/or vitamins.

Please note that each medication must have a problem associated with it but each problem does not require a medication associated with it.

Changes in your Medications

Name and Dose of Medication used	How often are you taking this Medication?	Date and Time (yyyy-mm-dd) and (hh:mm)		Why was the medicine stopped, started, or changed?	Number of associated symptom or side effect (previous page)
		Started	Stopped		

Comments:

SAMPLE MEMORY AID DIARY (DAY 0 to DAY 21)

Day 8 to Day 21

Other Symptoms or Side Effects:

You do not need to complete this portion of your Memory Aid Diary every day. You should use these pages to record any side effects or symptoms you experience AND any changes in the medication you are taking.

Please complete this section neatly and carefully. You will review this section with the research clinic staff.

Use this table to record any important changes in your health that you want to tell us about. Please make a note of any health problems you experience, any old problems that get worse or any new medicine you take.

- It is especially important that you make a note of problems that require unplanned visits to your doctor, a hospital stay, or a trip to the emergency room.
- It is also important that you make a note of changes in your medications. Each medication must have a problem associated with it but each problem does not require a medication associated with it.

Other Symptoms or Side Effects:	**Symptom/Side effect**	**Grade** See below	**Date it started** (yyyy-mm-dd)	**Do you still have it?** (yes/no) **If it is over, when did it end?** (yyyy-mm-dd)	**Did you make a visit or call to a doctor about it?** (yes/no)	**Did you go to an emergency room or hospital? Did you stay in hospital?** (yes/no, specify)	**Put an "X" in this box once you have told us about this problem**

Grade:	Comments:
0= No symptom<br>1= Mild: Noticeable, but doesn't interfere with my work/normal activities.<br>2= Moderate: Bad enough to limit my work/normal activities.<br>3= Severe: Bad enough to prevent my work/normal activities<br>4= Results to a visit to an emergency room or hospitalization	

SAMPLE MEMORY AID DIARY (DAY 0 to DAY 21)

Day 8 to Day 21

Other Symptoms or Side Effects (continued):

Other Symptoms or Side Effects:	**Symptom/Side effect**	**Grade** See below	**Date it started** (yyyy-mm-dd)	**Do you still have it?** (yes/no) **If it is over, when did it end?** (yyyy-mm-dd)	**Did you make a visit or call to a doctor about it?** (yes/no)	**Did you go to an emergency room or hospital? Did you stay in hospital?** (yes/no, specify)	**Put an "X" in this box once you have told us about this problem**

Grade:
0= No symptom
1= Mild: Noticeable, but doesn't interfere with my work/normal activities.
2= Moderate: Bad enough to limit my work/normal activities.
3= Severe: Bad enough to prevent my work/normal activities
4= Results to a visit to an emergency room or hospitalization

Comments:

SAMPLE MEMORY AID DIARY (DAY 0 to DAY 21)

Day 8 to Day 21

Changes in your Medication:

Record any new medications you have taken since your last visit. Examples may include prescription drugs, over-the-counter drugs, herbal supplements and/or vitamins.

Please note that each medication must have a problem associated with it but each problem does not require a medication associated with it.

Changes in your Medications

Name and Dose of Medication used	How often are you taking this Medication?	Date and Time (yyyy-mm-dd) and (hh:mm)		Why was the medicine stopped, started, or changed?	Number of associated symptom or side effect (previous page)
		Started	Stopped		

Comments:

SAMPLE-MEMORY AID DIARY (DAY 22 to DAY 201)	
**Day 22 to Day 201**	
**Study Name**	**Immunogenicity, Safety, and Tolerability of a Plant-Derived Seasonal VLP Quadrivalent Influenza Vaccine in Elderly**
**Protocol Number**	CP-Q13VLP-008
**Sponsor**	Medicago R&D Inc.<br>1020, route de l'Église, bureau 600<br>Sainte-Foy, Québec, Canada G1V 3V9
**Clinical site Address**	
**Principal Investigator Name**	
**Clinical Research Coordinator**	
**24-hour Emergency Number**	

SAMPLE MEMORY AID DIARY (DAY 22 to DAY 201)

Day 22 to Day 201

Other Symptoms or Side Effects:

You do not need to complete this portion of your Memory Aid Diary every day. You should use these pages to record any side effects or symptoms you experience AND any changes in the medication you are taking.

Please complete this section neatly and carefully. You will review this section with the research clinic staff.

Use this table to record any important changes in your health that you want to tell us about. Please make a note of any health problems you experience, any old problems that get worse or any new medicine you take.

- It is especially important that you make a note of problems that require unplanned visits to your doctor, a hospital stay, or a trip to the emergency room.
- It is also important that you make a note of changes in your medications. Each medication must have a problem associated with it but each problem does not require a medication associated with it.

Other Symptoms or Side Effects:

**Symptom/Side effect**	**Grade** See below	**Date it started** (yyyy-mm-dd)	**Do you still have it?** (yes/no) **If it is over, when did it end?** (yyyy-mm-dd)	**Did you make a visit or call to a doctor about it?** (yes/no)	**Did you go to an emergency room or hospital? Did you stay in hospital?** (yes/no, specify)	**Put an "X" in this box once you have told us about this problem**

Grade:
1= Mild – Noticeable, but doesn't interfere with my work / normal activities.
2= Moderate – Bad enough to limit my work / normal activities.
3= Severe – Bad enough to prevent my work / normal activities
4= Results to a visit to an emergency room or hospitalization

Comments:

SAMPLE MEMORY AID DIARY (DAY 22 to DAY 201)

Day 22 to Day 201

Other Symptoms or Side Effects (continued):

Other Symptoms or Side Effects:

**Symptom/Side effect**	**Grade** See below	**Date it started** (yyyy-mm-dd)	**Do you still have it?** (yes/no) **If it is over, when did it end?** (yyyy-mm-dd)	**Did you make a visit or call to a doctor about it?** (yes/no)	**Did you go to an emergency room or hospital? Did you stay in hospital?** (yes/no, specify)	**Put an "X" in this box once you have told us about this problem**

Grade:
- 1= Mild – Noticeable, but doesn't interfere with my work / normal activities.
- 2= Moderate – Bad enough to limit my work / normal activities.
- 3= Severe – Bad enough to prevent my work / normal activities
- 4= Results to a visit to an emergency room or hospitalization

Comments:

SAMPLE MEMORY AID DIARY (DAY 22 to DAY 201)

Day 22 to Day 201

Other Symptoms or Side Effects (continued):

Other Symptoms or Side Effects:	**Symptom/Side effect**	**Grade** See below	**Date it started** (yyyy-mm-dd)	**Do you still have it?** (yes/no) **If it is over, when did it end?** (yyyy-mm-dd)	**Did you make a visit or call to a doctor about it?** (yes/no)	**Did you go to an emergency room or hospital? Did you stay in hospital?** (yes/no, specify)	**Put an "X" in this box once you have told us about this problem**

Grade:
1= Mild – Noticeable, but doesn't interfere with my work / normal activities.
2= Moderate – Bad enough to limit my work / normal activities.
3= Severe – Bad enough to prevent my work / normal activities
4= Results to a visit to an emergency room or hospitalization

Comments:

SAMPLE MEMORY AID DIARY (DAY 22 to DAY 201)

Day 22 to Day 201

Changes in your Medication:

Record any new medications you have taken since your last visit. Examples may include prescription drugs, over-the-counter drugs, herbal product and/or vitamins.

Please note that each medication must have a problem associated with it but each problem does not require a medication associated with it.

Changes in your Medications	Name and Dose of Medication used	How often are you taking this Medication?	Date and Time (yyyy-mm-dd) and (hh:mm)		Why was the medicine stopped, started, or changed?	Number of associated symptom or side effect (previous page)
			Started	Stopped		
	Comments:					

SAMPLE MEMORY AID DIARY (DAY 22 to DAY 201)

Day 22 to Day 201

Changes in your Medication (continued):

Changes in your Medications

Name and Dose of Medication used	How often are you taking this Medication?	Date and Time (yyyy-mm-dd) and (hh:mm)		Why was the medicine stopped, started, or changed?	Number of associated symptom or side effect (previous page)
		Started	Stopped		

Comments:
