# Supplementary material for: Immunogenicity and safety of a quadrivalent plant-derived virus like particle influenza vaccine candidate—Two randomized Phase II clinical trials in 18 to 49 and ≥50 years old adults
Source: PLoS One. 2019 Jun 5;14(6):e0216533. doi: 10.1371/journal.pone.0216533 (PMC6550445; doi:10.1371/journal.pone.0216533)
Supplement: S3 File — (DOC) [file pone.0216533.s009.doc]

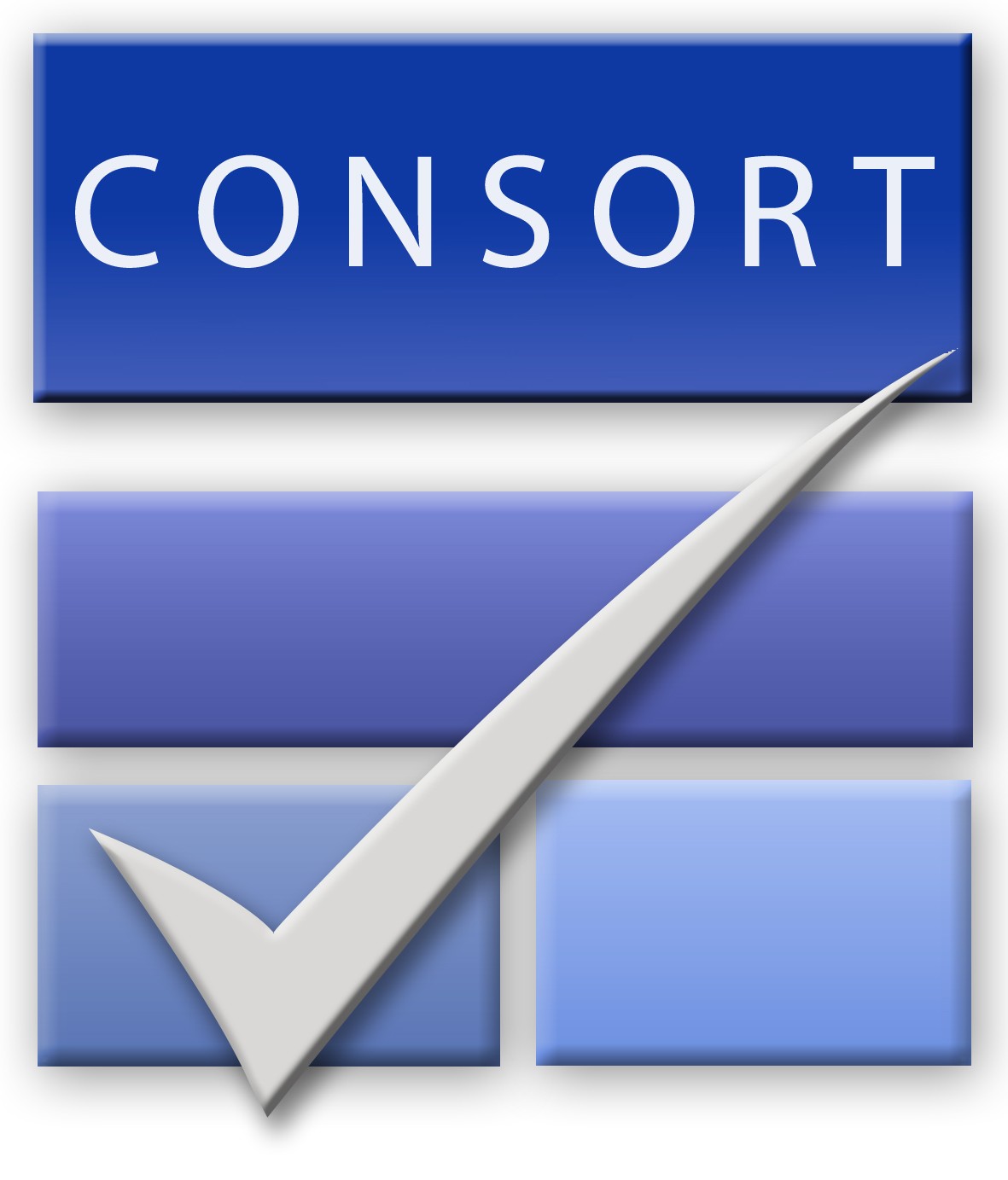
CONSORT 2010 checklist of information to include when reporting a randomised trial*

| Section/Topic | Item No | Checklist item | Reported on page No |
| --- | --- | --- | --- |
| Title and abstract | | | |
|  | 1a | Identification as a randomised trial in the title | Manuscript p1 |
|  | 1b | Structured summary of trial design, methods, results, and conclusions (for specific guidance see CONSORT for abstracts) | Manuscript p2 |
| Introduction | | | |
| Background and objectives | 2a | Scientific background and explanation of rationale | Manuscript pp3-4 |
|  | 2b | Specific objectives or hypotheses | Manuscript p4 & p19  Suppl Material (protocols) section 2 |
| Methods | | | |
| Trial design | 3a | Description of trial design (such as parallel, factorial) including allocation ratio | Manuscript pp6-7  Suppl Material (protocols) section 5.1 |
|  | 3b | Important changes to methods after trial commencement (such as eligibility criteria), with reasons | N/Ap |
| Participants | 4a | Eligibility criteria for participants | Manuscript pp5-7  Suppl Material (protocols) section 5.1.1 |
|  | 4b | Settings and locations where the data were collected | Manuscript pp5-7  Suppl Material (protocols) section 3 |
| Interventions | 5 | The interventions for each group with sufficient details to allow replication, including how and when they were actually administered | Manuscript pp6-7  Suppl Material (protocols) section 5.1 |
| Outcomes | 6a | Completely defined pre-specified primary and secondary outcome measures, including how and when they were assessed | Manuscript pp8-10  Suppl Material (protocols) section 2.1 and 2.2 |
|  | 6b | Any changes to trial outcomes after the trial commenced, with reasons | N/Ap |
| Sample size | 7a | How sample size was determined | Suppl Material (protocols) section 10 (18-49y) and 10.3 (>50y) |
|  | 7b | When applicable, explanation of any interim analyses and stopping guidelines | Suppl Material (protocols) section 8.1.8 |
| Randomisation: |  |  |  |
| Sequence generation | 8a | Method used to generate the random allocation sequence | Suppl Material (protocols) section 6.5 (18-49y) and 6.6 (>50y) |
|  | 8b | Type of randomisation; details of any restriction (such as blocking and block size) |  |
| Allocation concealment mechanism | 9 | Mechanism used to implement the random allocation sequence (such as sequentially numbered containers), describing any steps taken to conceal the sequence until interventions were assigned |  |
| Implementation | 10 | Who generated the random allocation sequence, who enrolled participants, and who assigned participants to interventions | Suppl Material (protocols) sections 5&6 |
| Blinding | 11a | If done, who was blinded after assignment to interventions (for example, participants, care providers, those assessing outcomes) and how |  |
|  | 11b | If relevant, description of the similarity of interventions |  |
| Statistical methods | 12a | Statistical methods used to compare groups for primary and secondary outcomes | Manuscript pp7-10  Suppl Material (protocols) section 10 |
|  | 12b | Methods for additional analyses, such as subgroup analyses and adjusted analyses |  |
| Results | | | |
| Participant flow (a diagram is strongly recommended) | 13a | For each group, the numbers of participants who were randomly assigned, received intended treatment, and were analysed for the primary outcome | Manuscript pp6-10; Fig. 1; figures' legends  Suppl Material (protocols) |
|  | 13b | For each group, losses and exclusions after randomisation, together with reasons | Manuscript Fig.1 (although not mentioning the reasons of exclusion) |
| Recruitment | 14a | Dates defining the periods of recruitment and follow-up | Manuscript pp6-7 and Suppl Material (protocols) |
|  | 14b | Why the trial ended or was stopped | N/Ap |
| Baseline data | 15 | A table showing baseline demographic and clinical characteristics for each group | Suppl tables 1&2 |
| Numbers analysed | 16 | For each group, number of participants (denominator) included in each analysis and whether the analysis was by original assigned groups | Manuscript p8-9, Fig. 1 |
| Outcomes and estimation | 17a | For each primary and secondary outcome, results for each group, and the estimated effect size and its precision (such as 95% confidence interval) | Manuscript Figs. 2, 3, 5 and suppl. Fig. 2 including figures' legends |
|  | 17b | For binary outcomes, presentation of both absolute and relative effect sizes is recommended | N/Ap |
| Ancillary analyses | 18 | Results of any other analyses performed, including subgroup analyses and adjusted analyses, distinguishing pre-specified from exploratory | Manuscript Figs 6-9 and Suppl Material (protocols) for definition of the exploratory endpoints |
| Harms | 19 | All important harms or unintended effects in each group (for specific guidance see CONSORT for harms) | Manuscript tables 1&2 and pp11-12 |
| Discussion | | | |
| Limitations | 20 | Trial limitations, addressing sources of potential bias, imprecision, and, if relevant, multiplicity of analyses | Manuscript 18-21 |
| Generalisability | 21 | Generalisability (external validity, applicability) of the trial findings | Manuscript 18-21 |
| Interpretation | 22 | Interpretation consistent with results, balancing benefits and harms, and considering other relevant evidence | Manuscript 18-21 |
| Other information | | |  |
| Registration | 23 | Registration number and name of trial registry | Manuscript p6 and p7 |
| Protocol | 24 | Where the full trial protocol can be accessed, if available | Available as Suppl Material |
| Funding | 25 | Sources of funding and other support (such as supply of drugs), role of funders | Manuscript p22 |

*We strongly recommend reading this statement in conjunction with the CONSORT 2010 Explanation and Elaboration for important clarifications on all the items. If relevant, we also recommend reading CONSORT extensions for cluster randomised trials, non-inferiority and equivalence trials, non-pharmacological treatments, herbal interventions, and pragmatic trials. Additional extensions are forthcoming: for those and for up to date references relevant to this checklist, see [www.consort-statement.org](http://www.consort-statement.org/).
